# Enantioselective Rhodium‐Catalyzed Coupling of Arylboronic Acids, 1,3‐Enynes, and Imines by Alkenyl‐to‐Allyl 1,4‐Rhodium(I) Migration

**DOI:** 10.1002/anie.201709334

**Published:** 2017-11-24

**Authors:** Michael Callingham, Benjamin M. Partridge, William Lewis, Hon Wai Lam

**Affiliations:** ^1^ School of Chemistry University of Nottingham University Park Nottingham NG7 2RD UK; ^2^ The GSK Carbon Neutral Laboratories for Sustainable Chemistry University of Nottingham Jubilee Campus, Triumph Road Nottingham NG7 2TU UK; ^3^ Department of Chemistry University of Sheffield Sheffield S3 7HF UK

**Keywords:** allylation, asymmetric catalysis, imines, isomerization, rhodium

## Abstract

A chiral rhodium complex catalyzes the highly enantioselective coupling of arylboronic acids, 1,3‐enynes, and imines to give homoallylic sulfamates. The key step is the generation of allylrhodium(I) species by alkenyl‐to‐allyl 1,4‐rhodium(I) migration.

Catalytic enantioselective nucleophilic allylations of aldehydes, ketones, and imines are valuable reactions for the preparation of chiral homoallylic alcohols and amines, which are useful building blocks for synthesis.^1^ Many of these processes utilize allyltin, allylboron, allylsilicon, or allyl halide compounds.1d,1f Although highly successful, one drawback is that preparation of reagents containing more complex allyl fragments can be non‐trivial. Of the methods that avoid such reagents,1a‐1c,1e one is generation of allylmetal species by the migratory insertion of an allene^2^ or a 1,3‐diene^3^ into a metal−element bond, followed by reaction with the electrophile (Scheme 1 A).^3^, ^4^, ^5^, ^6^, ^7^ Advantages of such three‐component reactions^3^, ^4^, ^5^ are the use of simpler reactants and the ability to rapidly increase structural complexity.^8^ Although highly enantioselective borylative three‐component nucleophilic allylations are known,^3^, ^4^, ^5^ the corresponding processes that form two carbon−carbon bonds have, to our knowledge, had limited success (up to 23 % *ee* has been obtained6c).^9^

**Scheme 1:**
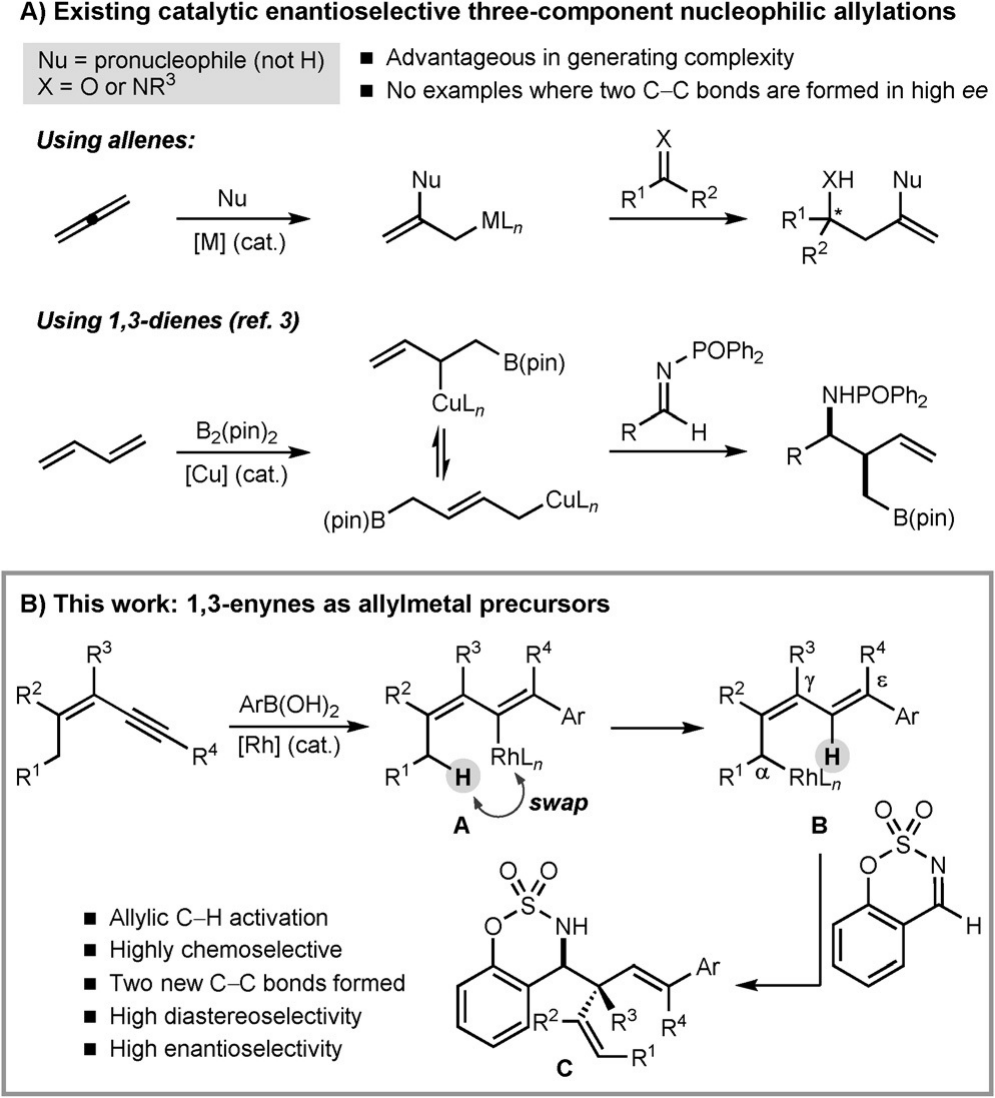
Catalytic enantioselective three‐component nucleophilic allylations.

Herein, we describe enantioselective three‐component nucleophilic allylations that involve an allylic C−H activation, an emerging strategy to generate nucleophilic allylmetal species.^10^, ^11^ This approach uses 1,3‐enynes, rather than allenes or 1,3‐dienes, and provides homoallylic sulfamates with high enantioselectivities.

Our reaction design is illustrated in Scheme 1 B. Rh^I^‐catalyzed addition of an arylboronic acid to the alkyne of a 1,3‐enyne would give alkenylrhodium(I) species **A**, which could undergo alkenyl‐to‐allyl 1,4‐rhodium(I) migration^12^, ^13^, ^14^, ^15^ to form allylrhodium(I) species **B**. Cyclic imines are excellent substrates for enantioselective Rh^I^‐catalyzed nucleophilic allylations^16^ and, therefore, we hoped that they could trap species **B** to give homoallylic sulfamates **C**. Cyclic sulfamates appear in a number of biologically active compounds.^17^

Although related to the two‐component arylative intramolecular allylations of ketones that we described recently,^10^ this three‐component coupling appeared to be significantly more challenging because numerous alternative pathways are possible. Firstly, chiral rhodium(I) complexes are known to promote the addition of arylboron reagents to cyclic imines.^18^ Secondly, addition of alkenylrhodium species **A** to the imine is possible.^19^ Thirdly, 1,4‐migration of rhodium(I) in species **A** to the *ortho* position of the aryl group derived from the arylboronic acid is known to be competitive.^10^ Finally, species **B** could potentially react with the imine in α‐ or ϵ‐selective allylations. Therefore, controlling the chemoselectivity was expected to be non‐trivial.

This study began with the reaction of imine **1 a** with 1,3‐enyne **2 a** (1.2 equiv) and PhB(OH)_2_ (1.5 equiv) in THF at 65 °C, in the presence of [{Rh(cod)Cl}_2_] (2.5 mol %), KF (1.5 equiv), and *t*AmOH (1.5 equiv) (Table 1, entry 1). Pleasingly, allylation product (±)‐**3 a** was formed as a single observable diastereomer (>19:1 d.r.) in 24 % NMR yield, along with several unidentified products. Using [{Ir(cod)Cl}_2_] increased the yield of (±)‐**3 a** to 53 %, although conjugated diene (±)‐**4** was also formed in 38 % yield (Table 1, entry 2).^20^ After screening additives, we found that ZnCl_2_ (1.0 equiv) increased the yield of (±)‐**3 a** to 81 %, and decreased the yield of (±)‐**4** (Table 1, entry 3). Next, chiral diene ligands^21^ were evaluated. An iridium complex of diene **L1** 22 returned only unchanged starting materials (Table 1, entry 4). However, the rhodium complex of **L1** gave *ent*‐**3 a** in 34 % yield and 99 % *ee*, with no trace of (±)‐**4** (Table 1, entry 5). The chiral tetrafluorobenzobarrelene **L2** 23 gave **3 a** in 83 % yield and 99 % *ee* (Table 1, entry 6). Repeating this reaction in the absence of ZnCl_2_ gave identical results (Table 1, entry 7). Surprisingly, the product of addition of PhB(OH)_2_ to imine **1 a** was not observed in the reactions described in Table 1, entries 2–7, while it was not clear whether this product was formed in the reaction described in Table 1, entry 1.

**Table 1:** Catalyst evaluation.^[a]^ 
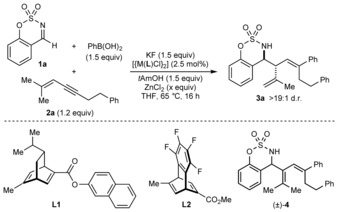

Entry	[M(**L**)Cl]_2_	ZnCl_2_ (*x* equiv)	Yield [%]^[b]^	*ee* [%]^[c]^
1	[{Rh(cod)Cl}_2_]	0	24	–
2	[{Ir(cod)Cl}_2_]	0	53 (38)^[d]^	–
3	[{Ir(cod)Cl}_2_]	1.0	81 (19)^[d]^	–
4	[{Ir(**L1**)Cl}_2_]^[e]^	1.0	n.r.	–
5	[{Rh(**L1**)Cl}_2_]^[e]^	1.0	34	−99^[f]^
6	[{Rh(**L2**)Cl}_2_]^[e]^	1.0	83	99
7	[{Rh(**L2**)Cl}_2_]^[e]^	0	83	99

[a] Reactions employed 0.05 mmol of **1 a**. Diastereomeric ratios were determined by ^1^H NMR analysis of the crude reactions. [b] Determined by ^1^H NMR analysis using 1,3,5‐trimethoxybenzene as an internal standard. [c] Determined by HPLC on a chiral stationary phase. [d] NMR yield of (±)‐**4**. [e] Formed by prior stirring 5.0 mol % of **L1** or **L2** with 2.5 mol % of [{Ir(coe)Cl}_2_] (coe=cyclooctene) or [{Rh(C_2_H_2_)_4_Cl_2_}_2_] in THF for 30 min. [f] The enantiomer of **3 a** was obtained. cod=1,5‐cyclooctadiene. *t*Am=*tert*‐amyl. n.r.=no reaction.

Variation of the imine was then explored by using [{Rh(**L2**)Cl}_2_] in the presence of ZnCl_2_ (1.0 equiv) (Scheme 2). Although ZnCl_2_ was unnecessary in the reaction of imine **1 a** (Table 1, compare entries 6 and 7), its inclusion gave more consistent results across a range of examples. Aldimines **1 a**–**1 g** reacted with 1,3‐enyne **2 a** and PhB(OH)_2_ to give products **3 a**–**3 g** in 52–75 % yield, and with the exception of **3 e**, all in >19:1 d.r. and 99 % *ee*.^24^ The reaction is tolerant of methyl (**3 b**), methoxy (**3 c** and **3 e**), halide (**3 d** and **3 e**), dioxole (**3 f**), and naphthyl groups (**3 g**) within the aldimine.

**Scheme 2:**
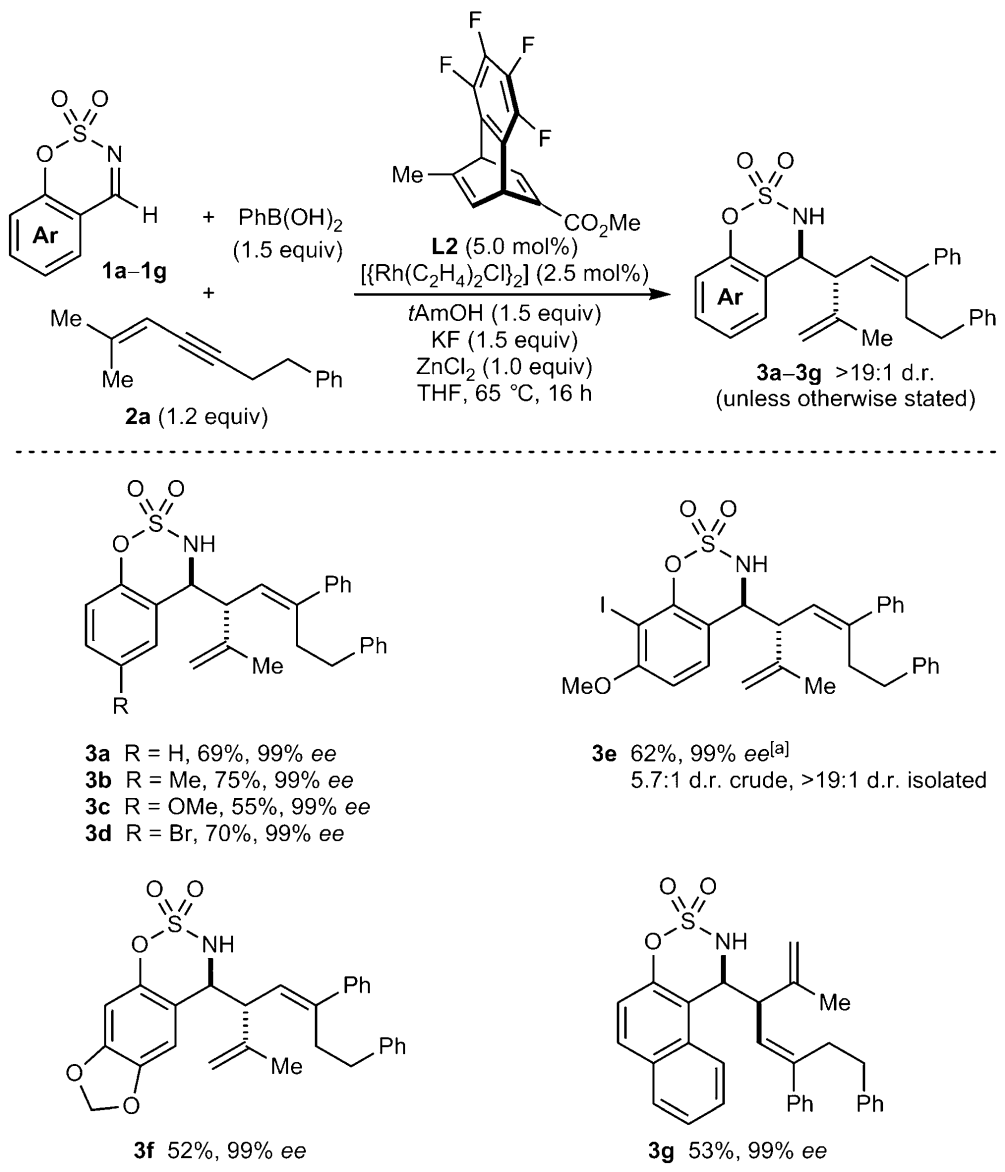
Variation of the imine. Reactions employed 0.30 mmol of the imine. Diastereomeric ratios were determined by ^1^H NMR analysis of the crude reactions. Yields are of isolated diastereomerically pure products. Enantiomeric excesses were determined by HPLC analysis on a chiral stationary phase. [a] Using 0.20 mmol of imine **1 e**.

Under the standard conditions, ketimine **5** reacted with 1,3‐enyne **2 a** and PhB(OH)_2_ to give a 1.7:1 mixture of diastereomers, in which the major diastereomer **6** [see Eq. 1 for the structure] has the opposite absolute configuration at the stereocenter bearing the 2‐propenyl group compared with the aldimine‐derived products **3** (Scheme 2). However, the diastereoselectivity was increased to 8:1 d.r. by using THF/MeCN (19:1) in place of THF only [Eq. (1)]. Initial purification of the mixture by chromatography gave **6** in approximately 50 % yield, 85 % purity, and 69 % *ee*. A second purification by trituration with pentane/toluene gave **6** with higher purity in 23 % yield and 93 % *ee*. This effect of nitrile co‐solvents altering the diastereochemical outcome was also observed in our study of arylative intramolecular allylations of ketones.^10^

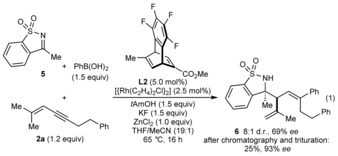



The reactions of imine **1 a**, PhB(OH)_2_, and various 1,3‐enynes **2 b**–**2 j** were then studied (Scheme 3). In most cases, the products were formed in >19:1 d.r. and the enantioselectivities were generally high. An alkyl chloride (**3 h**), silyl ether (**3 i**), or morpholine (**3 j**) in the 1,3‐enyne are tolerated, but **3 j** was formed in a modest 5:1 d.r. 1,3‐Enyne **2 e**, which contains a phenyl group *trans* to the alkyne, gave **3 k** in 49 % yield and 99 % *ee*, whereas 1,3‐enyne **2 f**, which contains a hydrogen atom at this site, returned only unchanged starting materials. However, using [{Ir(cod)Cl}_2_] (2.5 mol %) as the precatalyst gave racemic **3 l** in 90 % yield. 1,3‐Enyne **2 g** (a 5.8:1 *E*/*Z* mixture) gave **3 m** in 53 % yield and 99 % *ee*. In this case, no products that would be expected from reaction of the *Z* isomer of **2 g** were detected. 1,3‐Enyne **2 h** gave enol ether **3 n** in 66 % yield and 69 % *ee*. 1,3‐Enynes **2 i** and **2 j** gave products **3 o** and **3 p** containing an all‐carbon quaternary stereocenter, although **3 p** was almost racemic.

**Scheme 3:**
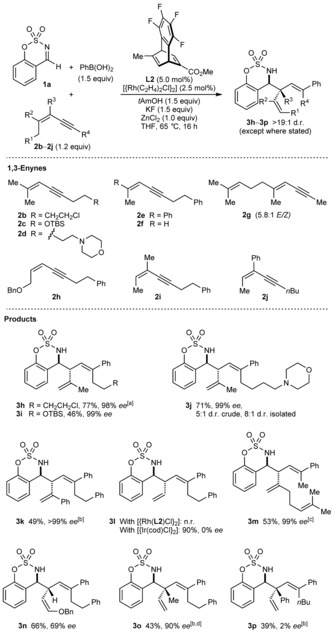
Variation of the 1,3‐enyne. See the footnote of Scheme 2 for general considerations. [a] Using 1.5 equiv of 1,3‐enyne **2 b**. [b] Using 3.0 equiv each of PhB(OH)_2_ and *t*AmOH. [c] Using 1.5 equiv of 1,3‐enyne **2 g** and 2.0 equiv each of PhB(OH)_2_, KF, and *t*AmOH. [d] An 8.2:1 inseparable mixture of **3 o** and the imine phenylation product was obtained (the yield of **3 o** has been adjusted accordingly).

Interestingly, 1,3‐enyne **2 k**, which contains a secondary alkyl group at the alkyne, reacted to give allylation product **3 q** as a mixture of *E*/*Z* isomers in a 1.7:1 ratio [Eq. 2]. The *E* isomer was obtained in 61 % yield and 98 % *ee*, whereas the *Z* isomer was obtained in 33 % yield and 87 % *ee*.^25^

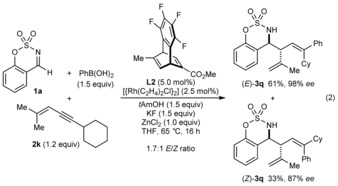



A range of arylboronic acids can be used in these reactions (Scheme 4). In all cases, the products were formed in >19:1 d.r. and with high enantioselectivities (96–99 % *ee*). For the reactions producing **3 y** and **3 z**, the products of direct arylation of the imine were observed in <15 % yield (by ^1^H NMR analysis) but were not isolated. The reaction is tolerant of aryl halides (**3 r**, **3 v**, and **3 x**), methoxy groups (**3 s** and **3 z**), alkenes (**3 t**), methyl groups (**3 u** and **3 y**), and esters (**3 w**).

**Scheme 4:**
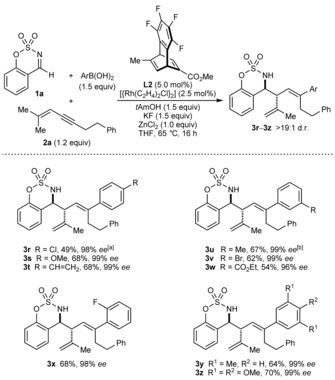
Variation of the arylboronic acid. See the footnote of Scheme 2 for general considerations. [a] Isolated in approximately 91 % purity (the yield has been adjusted accordingly). [b] Using 1.5 equiv of 1,3‐enyne **2 a**.

The reaction of imine **1 a** with PhB(OH)_2_ and the hexadeuterated 1,3‐enyne [D]_6_‐**2 a**, using the rhodium complex derived from racemic **L2**, gave [D]_6_‐**3 a**, in which there was >95 % deuterium transfer to the trisubstituted alkene [Eq. 3]. This result suggests 1,4‐rhodium(I) migration occurs by C−H oxidative addition to give a rhodium(III) hydride, followed by C−H reductive elimination.^10^, 13a, 14b, 26

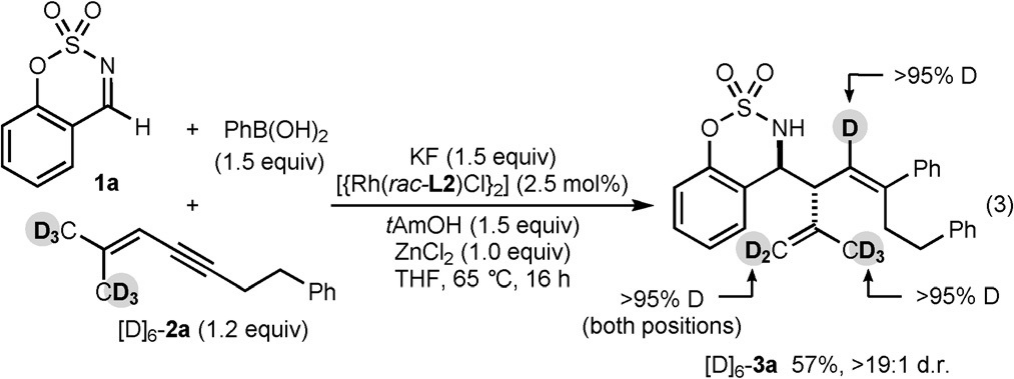



A possible catalytic cycle to give product **3 a** begins with formation of rhodium complex **7** from [{Rh(C_2_H_4_)_2_Cl}_2_], chiral diene **L2**, KF, and possibly *t*AmOH (Scheme 5). Transmetalation of the arylboronic acid with **7** gives arylrhodium species **8**, which could react with imine **1 a** to give **9**.^18^ However, we assume that the greater π‐Lewis basicity of alkynes compared to imine **1 a** leads to preferential coordination of **8** to 1,3‐enyne **2 a**, which gives, after migratory insertion, alkenylrhodium species **10**. In a previous study, we established that alkenyl‐to‐aryl 1,4‐rhodium(I) migration of intermediates similar to **10** to give arylrhodium species such as **11** is a significant pathway.^10^ The fact that products such as **12** are not observed suggests that **11** is too sterically hindered to react with imine **1 a**. Instead, **11** can undergo the reverse 1,4‐rhodium(I) migration to regenerate **10**, which, after alkenyl‐to‐allyl 1,4‐rhodium(I) migration, gives allylrhodium species **13**. Reaction of **13** with imine **1 a** through conformation **14**, in which the sulfonyl group of the imine and the methyl group of the allyl ligand project towards the less hindered quadrants defined by the ligand, gives **15**. Protonolysis of **15** with HX (X=Cl, F, or O*t*Am) releases product **3 a** and regenerates rhodium complex **7**. At present, the reason behind the beneficial effect of ZnCl_2_ is not known, although possibilities include acceleration of the allylation by Lewis acid activation, or improvement of catalyst turnover.

**Scheme 5:**
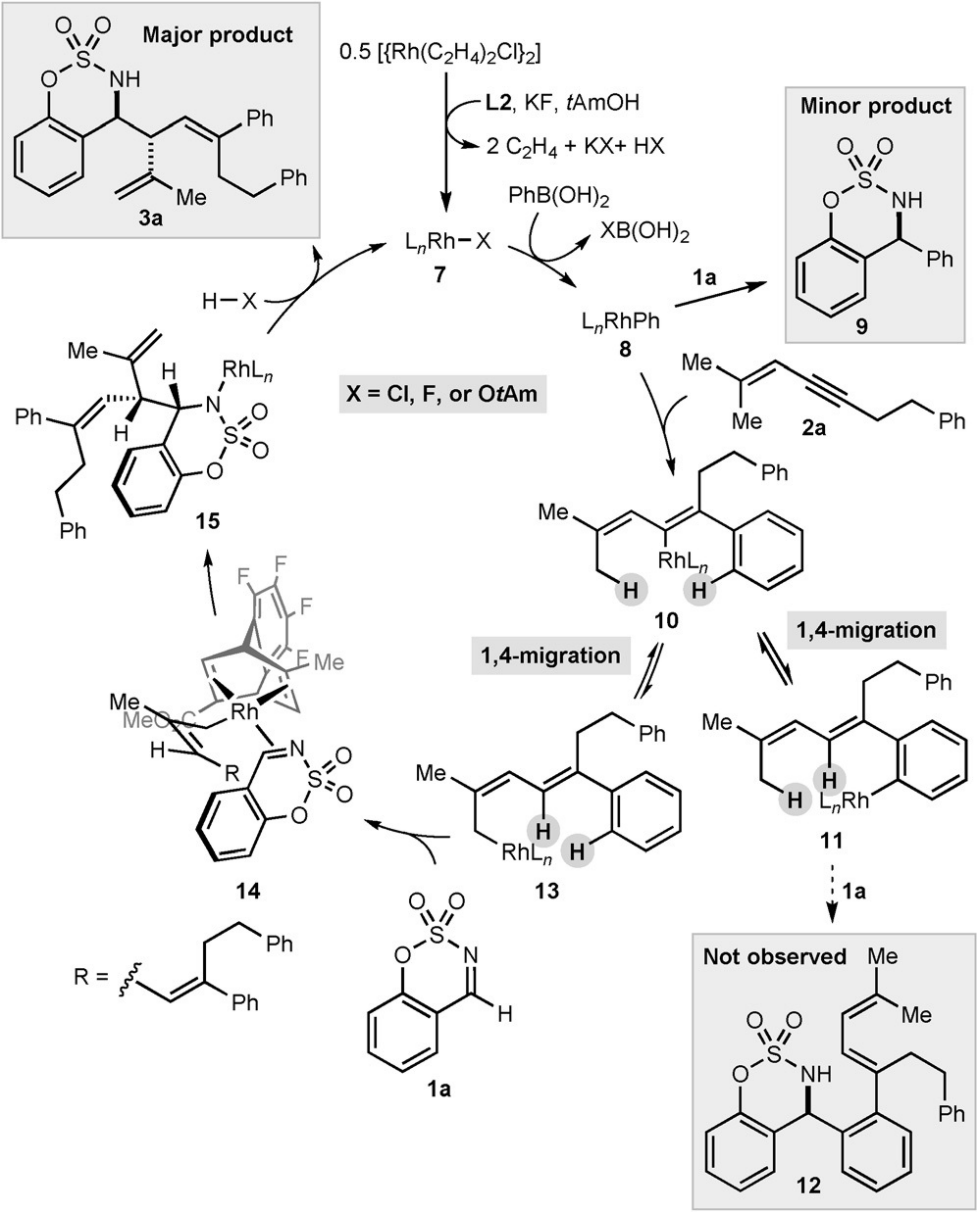
Proposed catalytic cycle.

In conclusion, we have developed highly stereoselective couplings of arylboronic acids, 1,3‐enynes, and cyclic imines. These reactions rely upon alkenyl‐to‐allyl 1,4‐metal migrations to generate nucleophilic allylmetal species, and proceed under iridium(I) catalysis to produce racemic products, or under rhodium(I) catalysis to produce highly enantioenriched products when a chiral tetrafluorobenzobarrelene ligand is used. Given the number of other products that could arise from alternative pathways, the chemoselectivity of this process is notable.^27^

## Conflict of interest

The authors declare no conflict of interest.

## Supporting information

As a service to our authors and readers, this journal provides supporting information supplied by the authors. Such materials are peer reviewed and may be re‐organized for online delivery, but are not copy‐edited or typeset. Technical support issues arising from supporting information (other than missing files) should be addressed to the authors.

Supplementary
