# Supplementary material for: Enantioselective Rhodium‐Catalyzed Coupling of Arylboronic Acids, 1,3‐Enynes, and Imines by Alkenyl‐to‐Allyl 1,4‐Rhodium(I) Migration
Source: Angew Chem Int Ed Engl. 2017 Nov 24;56(51):16352–6. doi: 10.1002/anie.201709334 (PMC5765452; doi:10.1002/anie.201709334)
Supplement: Supplementary file 1 — Supplementary [file ANIE-56-16352-s001.pdf]

Angewandte Chemie
Eine Zeitschrift der Gesellschaft Deutscher Chemiker

Supporting Information

Enantioselective Rhodium-Catalyzed Coupling of Arylboronic Acids, 1,3-Enynes, and Imines by Alkenyl-to-Allyl 1,4-Rhodium(I) Migration

*Michael Callingham, Benjamin M. Partridge, William Lewis, and Hon Wai Lam**

anie_201709334_sm_miscellaneous_information.pdf

Author Contributions

M.C. Conceptualization: Equal; Data curation: Equal; Formal analysis: Equal; Investigation: Lead; Methodology: Lead; Validation: Equal; Writing – original draft: Equal; Writing – review & editing: Equal
B.P. Data curation: Equal; Formal analysis: Equal; Investigation: Supporting; Methodology: Supporting; Validation: Equal; Writing – original draft: Equal; Writing – review & editing: Equal
W.L. Data curation: Supporting; Formal analysis: Supporting; Validation: Supporting
H.L. Conceptualization: Equal; Formal analysis: Supporting; Funding acquisition: Lead; Supervision: Lead; Validation: Supporting; Writing – original draft: Equal; Writing – review & editing: Lead.

Supporting Information

1. General Information 2
2. Substrate Synthesis 3
3. Enantioselective Rh-Catalyzed Arylative Allylation 11
4. Investigation of Deuterium Transfer 31
5. Measurement of Kinetic Isotope Effect in an Intermolecular Competition Experiment 32
6. NMR Spectra 33
7. HPLC Traces 74

1. General Information

All air-sensitive reactions were carried out under a nitrogen atmosphere using oven-dried apparatus. Anhydrous THF was dried and purified by passage through activated alumina columns using a solvent purification system. Anhydrous MeCN was purchased and used as received from Acros Organics. All commercially available reagents were used as received unless otherwise stated. Arylboronic acids were used as received unless the sample contained >10% boroxine as determined by ^{1}H NMR analysis. In this case, the boronic acid was stirred in a mixture of Et_2O and water for 30 min. The organic phase was separated, dried (Na_2SO_4), filtered and concentrated under reduced pressure to give the corresponding boronic acid which was used without further purification. All petroleum ether used was 40–60 °C petroleum ether. Thin layer chromatography (TLC) was performed on Merck DF-Alufoilien $60F_{254}$ 0.2 mm precoated plates. Compounds were visualized by exposure to UV light or by dipping the plates into solutions of potassium permanganate or vanillin followed by gentle heating. Flash column chromatography was carried out using silica gel (Fisher Scientific 60 Å particle size 35-70 micron). Melting points were recorded on a Gallenkamp melting point apparatus and are uncorrected. The solvent of recrystallization is reported in parentheses. Infra-red (IR) spectra were recorded on a Nicolet Avatar 360 FT instrument on the neat compound. NMR spectra were acquired on Bruker AV500, Bruker AV400, Bruker AV(III)400HD, Bruker DPX400, or Bruker DPX300 spectrometers. ^{1}H and ^{13}C NMR spectra were referenced to external tetramethylsilane *via* the residual protonated solvent (^{1}H) or the solvent itself (^{13}C). All chemical shifts are reported in parts per million (ppm). For $CDCl_3$, the shifts are referenced to 7.27 ppm for ^{1}H NMR spectroscopy and 77.0 ppm for ^{13}C NMR spectroscopy. Abbreviations used in the description of resonances are: s (singlet), d (doublet), t (triplet), q (quartet), app (apparent), br (broad) and m (multiplet). Coupling constants (*J*) are quoted to the nearest 0.1 Hz. HSQC and HMBC experiments were used to assist ^{1}H NMR assignments where required. ^{13}C NMR assignments were made using the DEPT sequence with secondary pulses at 90° and 135°. ^{19}F NMR spectra were not proton-decoupled, and were referenced through the solvent lock (^{2}H) signal according to IUPAC recommended secondary referencing method the Bruker protocols. High-resolution mass spectra were recorded using electrospray ionization (ESI) or electron impact ionization (EI) techniques. X-ray diffraction data were collected at 120 K on an Agilent SuperNova diffractometer using CuKα radiation. Chiral HPLC analysis was performed on an Agilent 1290 series or Agilent 1260 series instrument using 4.6 × 250 mm columns. Authentic racemic samples were prepared using $[Ir(cod)Cl]_2$ or [Rh(*rac*-**L2**)Cl]$_2$. [Rh(*rac*-**L2**)Cl]$_2$ was prepared by stirring $[Rh(C_2H_4)Cl]_2$ and **L2** (1.8 equiv) in CH_2Cl_2 at room temperature for 16 h. The solution was filtered through silica, and concentrated *in vacuo* to afford the complex as an orange solid.

2. Substrate Synthesis

Preparation of Ligands

Ligands **L1**[1] and **L2**[2] were prepared according to the literature.

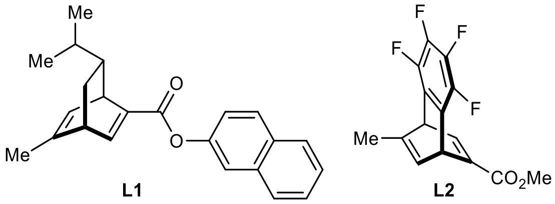

L1 **L2**

L2: $[\alpha]_D^{25.0}$ +24.2 (*c* 1.49, $CHCl_3$)

Preparation of Imines

Benzoxathiazine-2-2-dioxides **1a-c,**[3] **1d**,[4] and **1f-g**[3] were prepared according to the literature.

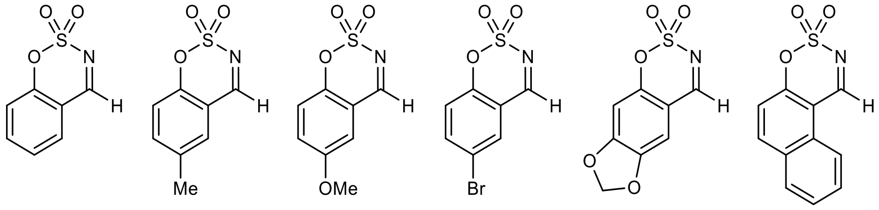

1a **1b** **1c** **1d** **1f** **1g**

Benzoisothiazole-1,1-dioxide **5** was prepared according to the literature.[5]

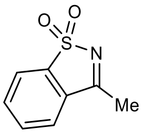

5

1. Okamoto, K.; Hayashi, T.; Rawal, V. H. *Chem. Commun.* **2009**, *32*, 4815–4817.
2. M. Hatano, T. Nishimura, *Angew. Chem., Int. Ed.* **2015**, *54,* 10949–10952.
3. Luo, Y.; Carnell, A. J.; Lam, H. W. *Angew. Chem. Int. Ed.* **2012**, *51*, 6762–6766.
4. Luo, Y.; Hepburn, H. B.; Chotsaeng, N.; Lam, H. W. *Angew. Chem., Int. Ed.* **2012**, *51*, 8309–8313.
5. Yang, Q.; Shang, G.; Gao, W.; Deng, J.; Zhang, X. *Angew. Chem., Int. Ed*. **2006**, *45*, 3832–3835.

8-Iodo-7-methoxy-1,2λ^6,3-benzoxathiazine-2,2-dione (1e)

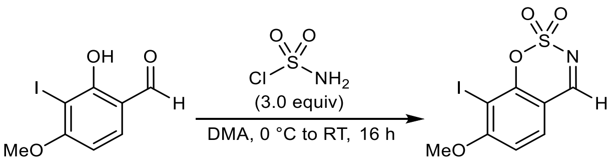

To a solution of aldehyde **S1**[6] (500 mg, 1.80 mmol) in DMA (15 mL) at room temperature was carefully added freshly prepared $ClSO_2NH_2$[7] (5.40 mmol) in small portions and the resulting solution was stirred for 18 h. The reaction was quenched carefully with ice-cold H_2O (20 mL) and the mixture was transferred to a separating funnel containing CH_2Cl_2 (20 mL). The aqueous layer was separated and extracted with CH_2Cl_2 (3 x 20 mL), and the combined organic layers were washed with saturated aqueous $NaHCO_3$ solution (30 mL), dried (Na_2SO_4), filtered, and concentrated *in vacuo*. The mixture was purified by column chromatography (50% petroleum ether/EtOAc) to give imine **1e** (102 mg, 17%) as a white solid. R_f = 0.12 (50% EtOAc/petroleum ether); m.p. 162-163 °C (Et_2O); IR 2991, 1580, 1387, 1133, 1065, 739 cm^{-1}; ^{1}H NMR (400 MHz, $CDCl_3$) δ 8.43 (1H, s, N=C**H**), 7.65 (1H, d, *J* = 8.6 Hz, Ar**H**), 6.87 (1H, d, *J* = 8.6 Hz, Ar**H**), 4.07 (3H, s, C**H$_3$**); ^{13}C NMR (126 MHz, $CDCl_3$) δ 166.4 (CH), 165.8 (C), 156.1 (C), 132.9 (CH), 110.7 (C), 108.2 (CH), 78.2 (C), 57.5 (CH_3); HRMS (ESI) Exact mass calcd for $[C_8H_6INNaO_4S]^+$ $[M+Na]^+$: 361.8954, found: 361.8970.

Preparation of 1,3-Enynes:

1,3-Enynes **2a**, $[D]_6$-**2a**, **2c**, **2e**, **2f**, and **2i** were prepared according to the literature.[8]

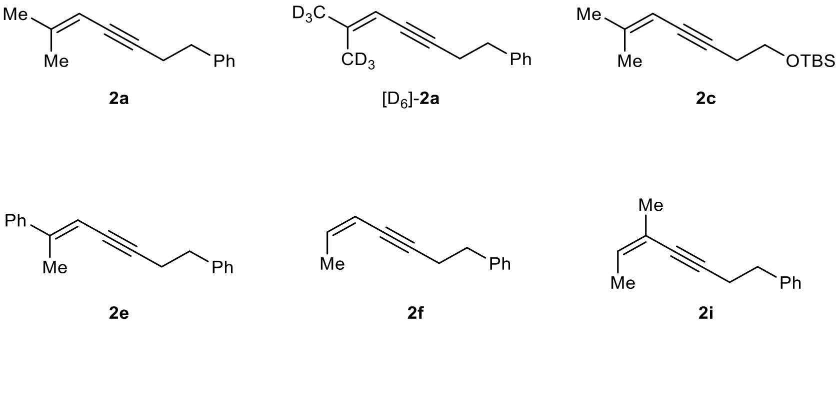

6. Schmidt, B.; Krehl, S.; Kelling, A.; Schilde, U. *J. Org. Chem.* **2012**, *77*, 2360–2367.
7. Wang, Y. Q.; Yu, C. B.; Wang, D. W.; Wang, X. B.; Zhou, Y. G. *Org. Lett.* **2008**, *10*, 2071–2074.
8. Burns, D. J.; Lam, H. W. *Angew. Chem., Int. Ed.* **2014**, *53*, 9931–9935.

9-Chloro-2-methylnon-2-en-4-yne (2b)

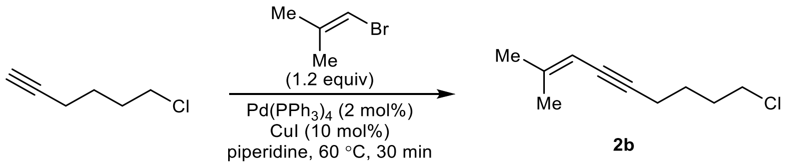

1-Bromo-2-methyl-prop-1-ene (1.4 mL, 14 mmol) was added to a solution of $Pd(PPh_3)_4$ (286 mg, 0.247 mmol) and CuI (236 mg, 1.24 mmol) in piperidine (20 mL). The solution was stirred for 1 min, 6-chlorohex-1-yne (1.5 mL, 12 mmol) was added, and the mixture was stirred at 60 °C for 30 min. The mixture was cooled to room temperature, and saturated aqueous NH_4Cl solution (50 mL) was added. The mixture was extracted with petroleum ether (3 × 20 mL) and the combined organic phases washed with brine (20 mL), dried ($MgSO_4$), filtered, and concentrated *in vacuo*. The mixture was purified by column chromatography (100% petroleum ether) to give *alkyne* **2b** (1.11 g, 53%) as a colorless oil. R_f = 0.46 (100% petroleum ether); IR 2910, 1434, 1335, 1049, 821, 651 cm^{-1}; ^{1}H NMR (400 MHz, $CDCl_3$) δ 5.25-5.20 (1H, m, C=C**H)**, 3.57 (2H, t, *J* = 6.6 Hz, **CH**$_2$Cl), 2.39 (2H, td, *J* = 6.8, 2.0 Hz, C≡CC**H**$_2$), 1.96-1.88 (2H, m, C**H**$_2$CH$_2$Cl), 1.86 (3H, s, C**H**$_3$), 1.78 (3H, s, C**H**$_3$), 1.73-1.65 (2H, m, C≡CCH$_2$C**H**$_2$); ^{13}C NMR (101 MHz, $CDCl_3$) δ 147.0 (C), 105.2 (CH), 91.0 (C), 79.1 (C), 44.6 (CH_2), 31.6 (CH_2), 26.2 (CH_2), 24.6 (CH_3), 20.8 (CH_3), 18.8 (CH_2); HRMS (GC-EIMS) Exact mass calcd for $[C_{10}H_{15}{}^{35}Cl]^+$ $[M]^+$: 170.0857, found: 170.0856.

4-(Hex-5-yn-1-yl)morpholine (S2)

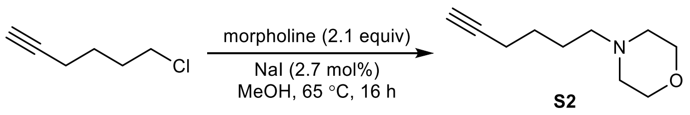

Morpholine (2.2 mL, 25 mmol) was added to a solution of 6-chlorohex-1-yne (1.5 mL, 12 mmol) and NaI (50 mg, 0.33 mmol) in MeOH (20 mL). The mixture was heated at 65 °C for 16 h. The mixture was concentrated *in vacuo*, and the residue was taken up in Et_2O (20 mL), acidified with 1 M aqueous HCl solution, and the aqueous phase separated. The mixture was basified with saturated aqueous K_2CO_3 solution and extracted with CH_2Cl_2 (3 × 10 mL). The combined organic phases were dried ($MgSO_4$), filtered, and concentrated *in vacuo* to give *alkyne* **S2** (463 mg, 22%) as a colorless oil. The data were consistent with those reported in the literature.[9]

9. Mizuno, A.; Kusama, H.; Iwasawa, N. *Angew. Chem., Int. Ed.* **2009**, *48*, 8318–8320.

4-(8-Methylnon-7-en-5-yn-1-yl)morpholine (2d)

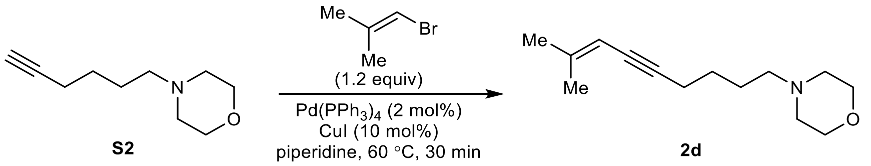

1-Bromo-2-methyl-prop-1-ene (0.31 mL, 3.1 mmol) was added to a solution of $Pd(PPh_3)_4$ (64.0 mg, 0.0554 mmol) and CuI (52.8 mg, 0.227 mmol) in piperidine (10 ml). The solution was stirred for 1 min, alkyne **S2** (0.464 g, 2.77 mmol) was added, and the mixture stirred at 60 °C for 30 min. The mixture was cooled to room temperature and water (5 mL) was added. The volatiles were removed *in vacuo*, water (10 mL) was added, and the mixture was extracted with EtOAc (3 × 20 mL). The combined organic phases were washed with brine (20 mL), dried ($MgSO_4$), filtered, and concentrated *in vacuo*. The mixture was purified by column chromatography (99% EtOAc, 1% Et_3N). The fractions containing the product were concentrated *in vacuo* and taken up in Et_2O (20 mL). 10% Aqueous HCl solution (10 mL) was added and the aqueous phase separated. The mixture was basified with saturated aqueous K_2CO_3 and extracted with CH_2Cl_2 (3 × 10 mL). The combined organic phases were dried ($MgSO_4$), filtered, and concentrated *in vacuo* to give *alkyne* **2d** (231 mg, 38%) as a yellow oil. R_f = 0.20 (100% EtOAc); IR 2933, 1445, 1117, 864 cm^{-1}; ^{1}H NMR (400 MHz, $CDCl_3$) δ 5.22 (1H, br s, C=C**H**), 3.72 (4H t, *J* = 4.7 Hz, 2 × OC**H**$_2$), 2.44 (4H, br s, 2 × OCH_2C**H**$_2$N), 2.39-2.31 (4H, m, NC**H**$_2$$CH_2CH_2$C**H**$_2$), 1.86 (3H, s, C**H**$_3$), 1.77 (3H, s, C**H**$_3$), 1.67-1.50 (4H, m, NCH_2C**H**$_2$C**H**$_2$$CH_2$); ^{13}C NMR (101 MHz, $CDCl_3$) δ 146.8 (C), 105.4 (CH), 91.6 (C), 78.7 (C), 67.0 (2 × CH_2), 58.5 (CH_2), 53.7 (2 × CH_2), 26.9 (CH_2), 25.7 (CH_2), 24.6 (CH_3), 20.8 (CH_3), 19.4 (CH_2); HRMS (ESI) Exact mass calcd for $[C_{14}H_{24}NO]^+$ $[M+H]^+$: 222.1852, found: 222.1852.

(*E*)-5,9-Dimethyldeca-4,8-dien-2-yne (*E*-2g)

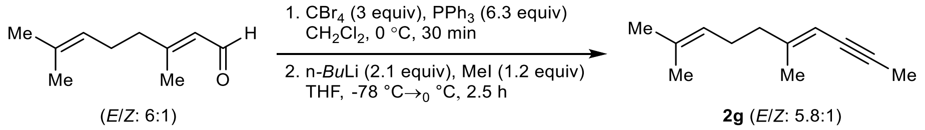

Carbon tetrabromide (6.50 g, 19.7 mmol) was added portion-wise to a solution of geranial[10] (*E*/*Z* ratio = 6:1, 1.00 g, 6.57 mmol) and PPh_3 (10.9 g, 41.4 mmol) in CH_2Cl_2 (50 mL) at 0 °C. The mixture was stirred for 30 min at 0 °C before saturated aqueous $NaHCO_3$ (50 mL) was added, and the mixture was extracted with CH_2Cl_2 (2 × 20 mL). The combined organic phases were dried ($MgSO_4$), filtered, and concentrated *in vacuo*. The mixture was subjected to column chromatography (100% petroleum ether). The residue was taken up in THF (25 mL), cooled to –78 °C, and *n*-BuLi (1.6 M, 8.7 mL, 13.8 mmol) was added dropwise. The mixture was stirred at –78 °C for 30 min, and then stirred at 0 °C

10. Prepared according to: Le, H.; Batten, A.; Morken, J. P. *Org. Lett.* **2014**, *16*, 2096–2099.

for 1 h. MeI (0.49 mL, 7.88 mmol) was added and the mixture stirred at 0 °C for 1 h. The reaction was quenched with water (25 mL), and the mixture was extracted with Et_2O (3 × 20 mL). The combined organic phases were dried ($MgSO_4$), filtered, and concentrated *in vacuo*. The mixture was purified by column chromatography (100% petroleum ether) to give *alkyne* **2g** in an *E/Z* ratio of 5.8:1 (825 mg, 77%) as a colorless oil. R_f = 0.28 (100% petrol); IR 2915, 1539, 1377, 1105, 830 cm^{-1}; ^{1}H NMR (400 MHz, $CDCl_3$) δ 5.25-5.20 (1H, m, C=C**H**C≡C), 5.10-5.04 (1H, m, $(CH_3)_2$C=C**H**), 2.15-2.03 (4H, m, C$\mathbf{H_2}$C$\mathbf{H_2}$), 1.98 (3H, d, *J* = 2.3 Hz, C≡C$\mathbf{H_3}$), 1.86 (3H, s, C$\mathbf{H_3}$C=CHC≡C), 1.68 (3H, s, C$\mathbf{H_3}$C(CH_3)=CH), 1.60 (3H, s, C$\mathbf{H_3}$C(CH_3)=CH); ^{13}C NMR (101 MHz, $CDCl_3$) δ 150.3 (C), 131.9 (C), 123.6 (CH), 105.0 (CH), 87.8 (C), 77.6 (C), 38.6 (CH_2), 26.3 (CH_2), 25.7 (CH_3), 19.0 (CH_3), 17.7 (CH_3), 4.4 (CH_3); HRMS (GC-EIMS) Exact mass calcd for $[C_{12}H_{18}]^+$ $[M]^+$: 162.1403, found: 162.1400.

Characteristic NMR signals of **(*Z*)-5,9-dimethyldeca-4,8-dien-2-yne (*Z*-2g)**:
^{1}H NMR (400 MHz, $CDCl_3$) δ 5.15 (1H, dddd, *J* = 7.0, 5.6, 2.9, 1.5 Hz, $(CH_3)_2$C=C**H**), 2.32-2.25 (2H, m, C$\mathbf{H_2}$), 1.96 (3H, d, *J* = 2.4 Hz, C≡CC$\mathbf{H_3}$), 1.76 (3H, s, C$\mathbf{H_3}$C=CHC≡C), 1.70 (3H, d, *J* = 1.4 Hz, C$\mathbf{H_3}$C(CH_3)=CH)), 1.63 (3H, s, C$\mathbf{H_3}$C(CH_3)=CH)); ^{13}C NMR (101 MHz, $CDCl_3$) δ 150.4 (C), 132.8 (C), 124.0 (CH), 105.6 (CH), 87.4 (C), 34.6 (CH_2), 25.7 (CH_2), 22.4 (CH_3), 17.6 (CH_3).

Methyl (*Z*)-7-phenylhept-2-en-4-ynoate (S3)

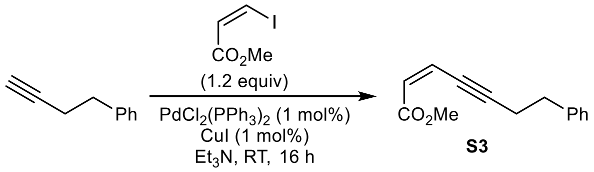

(*Z*)-3-Iodopropenoic acid methyl ester[11] (980 mg, 4.61 mmol) was added to a solution of $PdCl_2(PPh_3)_2$ (27 mg, 0.038 mmol), and CuI (7.2 mg, 0.038 mmol) in Et_3N (10 mL). The solution was stirred for 5 min, 4-phenyl-1-butyne (0.54 mL, 3.8 mmol) was added, and the mixture was stirred at room temperature for 16 h. The reaction mixture was diluted with Et_2O (20 mL), washed with 1 M aqueous HCl solution (20 mL), and the aqueous layer was further extracted with Et_2O (2 × 20 mL). The organic layers were combined, dried (Na_2SO_4), filtered, and concentrated *in vacuo*. The mixture was purified by column chromatography (5% EtOAc/petroleum ether) to give *alkyne* **S3** (637 mg, 77%) as an orange oil. R_f = 0.24 (5% EtOAc/petroleum ether); IR 2949, 2205, 1726 (C=O), 1193, 1172, 698 cm^{-1}; ^{1}H NMR (400 MHz, $CDCl_3$) δ 7.34-7.29 (2H, m, Ar**H**) 7.28-7.21 (3H, m, Ar**H**), 6.16 (1H, dt, *J* = 11.3, 2.3 Hz, **H**C=CHCO$_2$Me), 6.07 (1H, d, *J* = 11.3 Hz, C**H**CO$_2$Me), 3.76 (3H, s, OC$\mathbf{H_3}$), 2.94

11. Burns, D. J.; Best, D.; Wieczysty, M. D.; Lam, H. W. *Angew. Chem., Int. Ed.* **2015**, *54*, 9958–9962.

(2H, t, J = 7.6 Hz, PhC**H**$_2$), 2.76 (2H, td, J = 7.6, 2.3 Hz, PhCH$_2$C**H**$_2$); ^{13}C NMR (101 MHz, CDCl$_3$) δ 165.2 (C), 140.4 (C), 128.4 (4 × CH), 127.2 (CH), 126.3 (CH), 124.0 (CH), 103.2 (C), 78.1 (C), 51.4 (CH$_3$), 34.7 (CH$_2$), 22.2 (CH$_2$); HRMS (ESI) Exact mass calcd for $[C_{14}H_{14}NaO_2]^+$ $[M+Na]^+$: 237.0886, found: 237.0895.

(*Z*)-7-Phenylhept-2-en-4-yn-1-ol (S4)

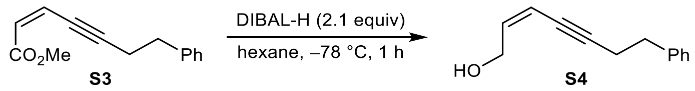

Alkyne **S3** (500 mg, 2.33 mmol) was added dropwise to a solution of DIBAL-H (4.9 mL of a 1.0 M solution in hexane, 4.9 mmol) at −78 °C. The mixture was stirred at −78 °C for 1 h. The reaction was warmed to 0 °C and 1 M aqueous HCl solution was added dropwise until effervescence ceased. Then, Et_2O (15 mL) and 1 M aqueous HCl solution (15 mL) were added, the layers were separated, and the aqueous layer was further extracted with Et_2O (2 × 15 mL). The organic layers were combined, washed with brine (15 mL) dried, (Na_2SO_4), filtered and concentrated *in vacuo*. The mixture was purified by column chromatography (50% Et_2O/petroleum ether) to give *alcohol* **S4** (297 mg, 68%) as a yellow oil. R_f = 0.46 (66% Et_2O/petroleum ether); IR 3316 (O-H), 2925, 1453, 1015, 742, 698 cm^{-1}; ^{1}H NMR (400 MHz, CDCl$_3$) δ 7.36-7.29 (2H, m, Ar**H**), 7.27-7.21 (3H, m, Ar**H**), 6.01 (1H, dt, J = 10.8, 6.4 Hz, =C**H**CH$_2$), 5.61-5.54 (1H, m, C**H**=CHCH$_2$OH), 4.31 (2H, dd, J = 6.4, 1.4 Hz, C**H**$_2$OH), 2.87 (2H, t, J = 7.5 Hz, PhC**H**$_2$), 2.66 (2H, td, J = 7.5, 2.2 Hz, PhCH$_2$C**H**$_2$), 1.65 (1H, br s, O**H**); ^{13}C NMR (101 MHz, CDCl$_3$) δ 140.4 (C), 140.2 (CH), 128.41 (2 × CH), 128.37 (2 × CH), 126.3 (CH), 111.0 (CH), 109.9 (C), 95.6 (C), 60.8 (CH$_2$), 34.9 (CH$_2$), 21.6 (CH$_2$); HRMS (ESI) Exact mass calcd for $[C_{13}H_{14}NaO]^+$ $[M+Na]^+$: 209.0937, found: 209.0922.

[(*Z*)-7-(Benzyloxy)hept-5-en-3-yn-1-yl]benzene (2h)

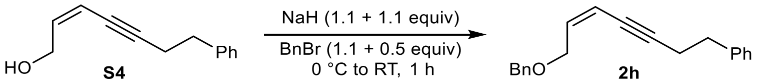

NaH (60% wt in mineral oil, 101 mg, 2.50 mmol) was added to a stirred solution of alcohol **S4** (391 mg, 2.10 mmol) in THF (10 mL) at 0 °C, and the mixture was stirred at 0 °C for 30 min. Benzyl bromide (0.30 mL, 2.5 mmol) was added, the reaction was warmed to room temperature, and stirred for a further 1 h. Additional benzyl bromide (0.15 mL, 1.3 mmol) and NaH (60% wt in mineral oil, 101 mg, 2.50 mmol) were then added, and the reaction mixture stirred for 16 h at room temperature After this time 1 M aqueous HCl solution (10 mL) was added, the layers were separated, and the aqueous layer was extracted with Et_2O (2 × 15 mL). The organic layers were combined, washed with brine (15 mL), dried (Na_2SO_4), and concentrated *in vacuo*. The mixture was purified by column chromatography (5% Et_2O/petroleum ether) to give *1,3-enyne* **2h** (343 mg, 59%) as a pale yellow oil.

R_f = 0.40 (5% EtOAc/petroleum ether); IR 2923, 1453, 1093, 1075, 734, 696 cm^{-1}; ^{1}H NMR (400 MHz, $CDCl_3$) δ 7.39-7.27 (7H, m, Ar**H**), 7.26-7.19 (3H, m, Ar**H**), 6.00 (1H, dt, *J* = 10.8, 6.4 Hz, HC=C**H**CH_2), 5.66-5.59 (1H, m, CC**H**=CH), 4.49 (2H, s, PhC**H**$_2$O), 4.22 (2H, dd, *J* = 6.4, 1.5 Hz, HC=CHC**H$_2$**), 2.84 (2H, t, *J* = 7.4 Hz, PhC**H$_2$**), 2.63 (2H, td, *J* = 7.4, 2.1 Hz, PhCH_2C**H$_2$**); ^{13}C NMR (101 MHz, $CDCl_3$) δ 140.5 (C), 138.3 (C), 138.2 (CH), 128.40 (2 × CH), 128.36 (2 × CH), 128.3 (2 × CH), 127.8 (2 × CH), 127.6 (CH), 126.3 (CH), 112.0 (CH), 95.5 (C), 77.1, (C), 72.2 (CH_2), 67.8 (CH_2), 35.0 (CH_2), 21.6 (CH_2); HRMS (ESI) Exact mass calcd for $[C_{20}H_{20}NaO]^+$ $[M+Na]^+$: 299.1406, found: 299.1417.

(*Z*)-Non-2-en-4-yn-3-ylbenzene (2j)

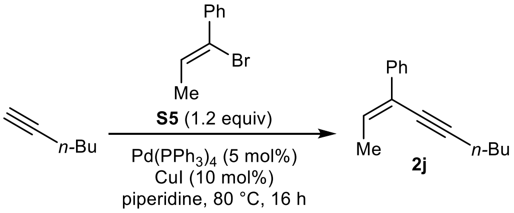

Vinyl bromide **S5**[12] (113 mg, 0.573 mmol) in piperidine (5 mL) was added to a vial containing $Pd(PPh_3)_4$ (30.1 mg, 0.026 mmol), and CuI (9.9 mg, 0.052 mmol). The solution was stirred for 5 min and 1-hexyne (60 µL, 0.52 mmol) was then added and the mixture was heated at 80 °C for 16 h. The reaction mixture was cooled to room temperature, diluted with Et_2O (10 mL), washed with 1 M aqueous HCl solution (10 mL), and the aqueous layer was further extracted with Et_2O (2 × 10 mL). The organic layers were combined, dried (Na_2SO_4), and concentrated *in vacuo*. The mixture was purified by column chromatography (100% petroleum ether) to give *1,3-enyne* **2j** (65 mg, 63%) as an orange oil. R_f = 0.39 (100% petroleum ether); IR 2930, 1493, 1447, 757, 693 cm^{-1}; ^{1}H NMR (400 MHz, $CDCl_3$) δ 7.61-7.56 (2H, m, Ar**H**), 7.35-7.29 (2H, m, Ar**H**), 7.28-7.21 (1H, m, Ar**H**), 6.40 (1H, q, *J* = 6.9 Hz, C=C**H**), 2.49 (2H, t, *J* = 7.0 Hz, C≡CC**H$_2$**), 2.05 (3H, d, *J* = 6.9 Hz, C=CHC**H$_3$**), 1.67-1.58 (2H, m, C≡CCH_2C**H$_2$**), 1.57-1.46 (2H, m, C**H$_2$**CH_3), 0.97 (3H, t, *J* = 7.3 Hz, CH_2C**H$_3$**); ^{13}C NMR (101 MHz, $CDCl_3$) δ 138.9 (C), 131.5 (CH), 128.2 (2 × CH), 127.1 (CH), 125.8 (2 × CH), 124.7 (C), 96.7 (C), 77.6 (C), 31.0 (CH_2), 22.0 (CH_2), 19.3 (CH_2), 16.7 (CH_3), 13.6 (CH_3); HRMS (GC-EIMS) Exact mass calcd for $[C_{15}H_{18}Na]^+$ $[M+Na]^+$: 198.1400, found: 198.1403.

12. Spaggiari, A.; Vaccari, D.; Davoli, P.; Torre, G.; Prati, F. *J. Org. Chem.* **2007**, *72*, 2216–2219.

(4-Methylpent-3-en-1-yn-1-yl)cyclohexane (2k)

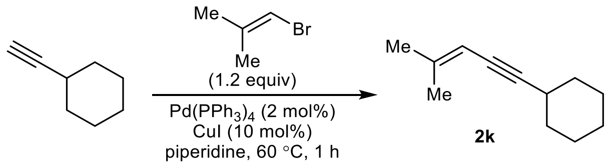

Cyclohexylacetylene (300 mg, 2.77 mmol) was added to a solution of 1-bromo-2-methyl-prop-1-ene (450 mg, 3.33 mmol), $Pd(PPh_3)_4$ (64.0 mg, 0.055 mmol) and CuI (53.0 mg, 0.028 mmol) in pyrrolidine (5 ml). The mixture was stirred at 65 °C for 1 h, and then cooled to room temperature. Saturated aqueous NH_4Cl solution (10 ml) was added and the mixture extracted with Et_2O (3 × 10 mL). The combined organic phases were washed with brine (10 mL), dried (Na_2SO_4), filtered, and concentrated in vacuo. The mixture was purified by column chromatography (100% petroleum ether) to give *1,3-enyne* **2k** (404 mg, 90%) as a colorless oil. R_f = 0.60 (100% petroleum ether); IR 2910, 1434, 1335, 1049, 821, 651 cm^{-1}; ^1H NMR (400 MHz, $CDCl_3$) δ 5.28-5.25 (1H, m, C=C**H**), 2.57-2.46 (1H, m, C**H**CH_2), 1.89-1.87 (3H, m, C**H3**), 1.87-1.79 (2H, m, (C**H2**)5) 1.79-1.77 (3H, m, C**H3**), 1.76-1.67 (2H, m, (C**H2**)5), 1.55-1.41 (2H, m, (C**H2**)5), 1.39-1.25 (4H, m, (C**H2**)5); ^{13}C NMR (101 MHz, $CDCl_3$) δ 146.6 (C), 105.5 (CH), 96.4 (C), 78.3 (C), 33.0 (2 × CH_2), 29.8 (CH), 26.0 (CH_2), 24.9 (2 × CH_2), 24.6 (CH_3), 20.7 (CH_3); HRMS (GC-EIMS) Exact mass calcd for $[C_{12}H_{18}]^+$ $[M]^+$: 162.1403, found: 162.1406.

3. Enantioselective Rh-Catalyzed Arylative Allylation

General Procedure A: Enantioselective Arylative Allylation Reaction

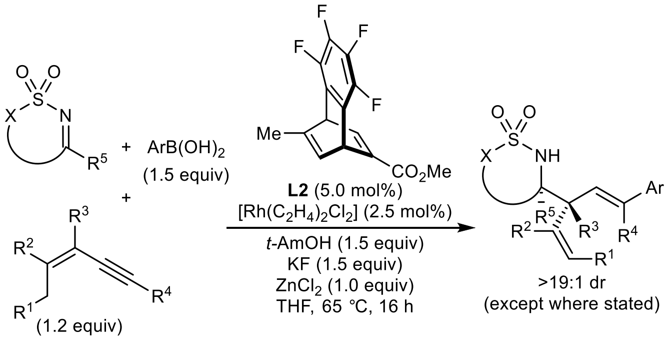

$[Rh(C_2H_4)_2Cl]_2$ (2.9 mg, 0.0075 mmol, 2.5 mol%) and **L2** (4.5 mg, 0.015 mmol, 5 mol%) were added to an oven-dried microwave vial and purged with nitrogen for 1 h. Degassed anhydrous THF (1.5 mL) was added and the mixture stirred for 30 min at room temperature. Imine (0.30 mmol), 1,3-enyne (0.36 mmol), arylboronic acid (0.45 mmol), and KF (26.1 mg, 0.450 mmol) were added to a separate oven-dried microwave vial. The vial was purged with nitrogen for 1 h, and the catalyst solution was then added to the vial containing the imine. *t*-Amyl alcohol (0.05 mL, 0.45 mmol) and $ZnCl_2$ solution (0.7 M in THF, 0.43 mL, 0.30 mmol) were added and the reaction mixture was stirred at 65 °C for 16 h. The mixture was cooled to room temperature, diluted with saturated aqueous NH_4Cl solution (5 mL) and water (5 mL), and extracted with EtOAc (3 × 10 mL). The combined organic phases were washed with brine, dried (Na_2SO_4), filtered, and concentrated *in vacuo*. The crude residue was purified by column chromatography to give the allylation product.

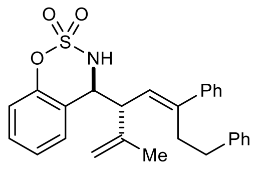

(4*S*)-4-[(3*R*,*E*)-2-Methyl-5,7-diphenylhepta-1,4-dien-3-yl]-3,4-dihydro-1,2λ^6,3-benzoxathiazine-2,2-dione (3a). General Procedure A was followed using imine **1a** (55.0 mg, 0.300 mmol), 1,3-enyne **2a** (66.3 mg, 0.360 mmol), and phenylboronic acid (54.9 mg, 0.450 mmol). The mixture was purified by column chromatography (10% EtOAc/petroleum ether) to give *allylation product* **3a** (92.0 mg, 69%) as a colorless oil (*ca.* 95% purity; small quantities of solvents and silicone grease remained). R_f = 0.21 (10% EtOAc/petroleum ether); $[\alpha]_D^{25.2}$ –26.2 (c 0.32, $CHCl_3$); IR 3268 (N-H), 2925, 1367, 1192, 1168, 754, 697 cm^{-1}; ^{1}H NMR (400 MHz, $CDCl_3$) δ 7.38-7.27 (4H, m, Ar**H**), 7.26-7.18 (5H, m, Ar**H**), 7.17-7.12 (1H, m, Ar**H**), 7.11-7.01 (4H, m, Ar**H**), 5.51 (1H, d, *J* = 9.3 Hz, C=C**H**), 5.07 (1H, s, C=C**H**$_A$H$_B$), 5.03-4.96 (2H, m, C=CH$_A$**H**$_B$ and C**H**NH), 4.07 (1H, d, *J* = 7.3 Hz, N**H**), 3.57 (1H, dd, *J* = 9.3, 6.4 Hz, C=CC**H**), 2.83-2.71 (2H, m, PhC**H**$_2$), 2.69-2.59 (1H, m, PhCH$_2$C**H**$_A$H$_B$), 2.42 (1H, dt, *J* = 13.6, 8.1 Hz, PhCH$_2$CH$_A$**H**$_B$), 1.92 (3H, s, C**H**$_3$); ^{13}C NMR (101 MHz, $CDCl_3$) δ 151.4 (C), 143.4 (C), 143.2 (C), 141.7 (C), 141.5 (C), 129.3 (CH), 128.47 (2 × CH), 128.45 (2 × CH), 128.4 (2

× CH), 127.5 (CH), 126.7 (CH), 126.6 (2 × CH), 126.3 (CH), 125.2 (CH), 124.8 (CH), 122.1 (C), 118.7 (CH), 115.3 (CH_2), 56.9 (CH), 49.1 (CH), 33.7 (CH_2), 31.9 (CH_2), 21.4 (CH_3); HRMS (ESI) Exact mass calcd for $[C_{27}H_{27}NNaO_3S]^+$ $[M+Na]^+$: 468.1604, found: 468.1609; Enantiomeric excess was determined by HPLC using a Chiralcel OD-H column (90:10 *iso*-hexane:*i*-PrOH, 1.0 mL/min, 254 nm): t_r (major) = 7.3 min, t_r (minor) = 14.7 min, 99% ee.

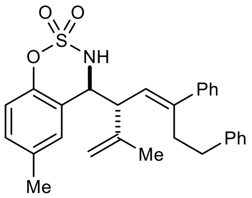

(4*S*)-6-Methyl-4-[(3*R*,*E*)-2-methyl-5,7-diphenylhepta-1,4-dien-3-yl]-3,4-dihydro-1,2λ⁶,3-benzoxathiazine-2,2-dione (3b). General Procedure A was followed using imine **1b** (59.2 mg, 0.300 mmol), 1,3-enyne **2a** (66.3 mg, 0.360 mmol), and phenylboronic acid (54.9 mg, 0.450 mmol). The mixture was purified by column chromatography (15% Et_2O/*iso*-hexane) to give *allylation product* **3b** (103 mg, 75%) as a pale brown solid (*ca.* 95% purity; small quantities of unidentified impurities and silicone grease remained). R_f = 0.23 (15% Et_2O/*iso*-hexane); m.p. 118-120 °C (Et_2O); $[\alpha]_D^{24.1}$ –114.4 (*c* 0.37, $CHCl_3$); IR 3310 (N-H), 2935, 1433, 852, 748, 694 cm^{-1}; ^1H NMR (400 MHz, $CDCl_3$) δ 7.39-7.21 (8H, m, Ar**H)**, 7.15-7.10 (4H, m, Ar**H**), 6.99-6.91 (1H, m, Ar**H**), 5.60 (1H, d, *J* = 9.6 Hz, C=C**H**), 5.10 (1H, m, C=C$\mathbf{H_A}H_B$), 5.06-4.97 (2H, m, C=C$H_A\mathbf{H_B}$ and C**H**NH), 4.23 (1H, d, *J* = 6.8 Hz, N**H**), 3.66 (1H, dd, *J* = 9.7, 6.0 Hz, C=CC**H**), 2.83-2.72 (2H, m, PhC$\mathbf{H_2}$), 2.69-2.56 (1H, m, PhCH_2C$\mathbf{H_A}H_B$), 2.52-2.40 (1H, m Ph$CH_2CH_A\mathbf{H_B}$), 2.35 (3H, s, ArC$\mathbf{H_3}$), 1.96 (3H, C=CC$\mathbf{H_3}$); ^{13}C NMR (101 MHz, $CDCl_3$) δ 149.2 (C), 143.4 (C), 143.2 (C), 141.7 (C), 141.5 (C), 134.9 (C), 129.9 (CH), 128.4 (2 × CH), 128.4 (2 × CH), 128.2 (2 × CH), 127.4 (CH), 127.0 (CH), 126.6 (2 × CH), 126.2 (CH), 124.5 (CH), 121.7 (C), 118.5 (CH), 115.3 (CH_2), 57.1 (CH), 49.4 (CH), 33.9 (CH_2), 31.9 (CH_2), 21.4 (CH_3), 20.9 (CH_3); HRMS (ESI) Exact mass calcd for $[C_{28}H_{29}NNaO_3S]^+$ $[M+Na]^+$: 482.1760, found: 482.1777; Enantiomeric excess was determined by HPLC using a Chiralcel OD-H column (90:10 *iso*-hexane:*i*-PrOH, 1.0 mL/min, 210 nm): t_r (major) = 6.9 min, t_r (minor) = 10.4 min, 99% ee.

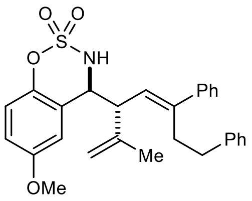

(4*S*)-6-Methoxy-4-[(3*R*,*E*)-2-methyl-5,7-diphenylhepta-1,4-dien-3-yl]-3,4-dihydro-1,2λ⁶,3-benzoxathiazine-2,2-dione (3c). General Procedure A was followed using imine **1c** (64.0 mg, 0.300 mmol), 1,3-enyne **2a** (66.3 mg, 0.360 mmol), and phenylboronic acid (54.9 mg, 0.450 mmol). The mixture was purified by column chromatography (10% EtOAc/petroleum ether) to give *allylation product* **3c** (79.0 mg, 55%) as a yellow oil (*ca.* 95% purity; small quantities of unidentified impurities and silicone grease remained). R_f = 0.21 (15% EtOAc/petroleum ether); $[\alpha]_D^{24.9}$ –59.6 (*c* 0.34, $CHCl_3$); IR 3268 (N-H), 2936, 1490, 1444, 1169, 848, 698 cm^{-1}; ^1H NMR (400 MHz, $CDCl_3$) δ 7.38-7.14 (8H, m, Ar**H**), 7.09-7.05 (2H, m, Ar**H**), 6.98 (1H, d, *J* = 8.8 Hz, Ar**H**), 6.87-6.80 (2H, m, Ar**H**), 5.57 (1H,

d, J = 9.7 Hz, C=C**H**), 5.05 (1H, m, C=C**H$_A$**H$_B$) 5.00 (1H, m, C=CH$_A$**H$_B$**), 4.94 (1H, dd, J = 7.4, 6.6 Hz, C**H**NH), 3.92 (1H, d, J = 7.4 Hz, N**H**), 3.75 (3H, s, OC**H$_3$**), 3.53 (1H, dd, J = 9.7, 6.6 Hz, C=CC**H**), 2.82-2.74 (2H, m, PhC**H$_2$**), 2.67-2.57 (1H, m, PhCH$_2$C**H$_A$**H$_B$), 2.41 (1H, dt, J = 13.7, 8.2 Hz, PhCH$_2$CH$_A$**H$_B$**), 1.90 (3H, s, CC**H$_3$**); ^{13}C NMR (101 MHz, CDCl$_3$) δ 156.5 (C), 145.1 (C), 143.7 (C), 142.9 (C), 141.7 (C), 141.5 (C), 128.5 (4 × CH), 128.3 (2 × CH), 127.5 (CH), 126.7 (2 × CH), 126.3 (CH), 124.8 (CH), 123.2 (C), 119.6 (CH), 115.2 (CH$_2$), 114.4 (CH), 112.0 (CH), 57.1 (CH), 55.6 (CH$_3$), 49.6 (CH), 33.9 (CH$_2$), 31.8 (CH$_2$), 21.2 (CH$_3$); HRMS (ESI) Exact mass calcd for [$C_{28}H_{29}NNaO_4S$]$^+$ [M+Na]$^+$: 498.1710, found: 498.1704; Enantiomeric excess was determined by HPLC using a Chiralcel OD-H column (95:5 *iso*-hexane:*i*-PrOH, 0.8 mL/min, 210 nm): t_r (major) = 22.7 min, t_r (minor) = 28.5 min, 99% ee.

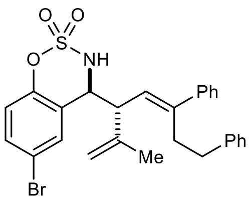

(4*S*)-6-Bromo-4-[(3*R*,*E*)-2-methyl-5,7-diphenylhepta-1,4-dien-3-yl]-3,4-dihydro-1,2λ^6,3-benzoxathiazine-2,2-dione (3d). General Procedure A was followed using imine **1d** (78.6 mg, 0.300 mmol), 1,3-enyne **2a** (66.3 mg, 0.360 mmol), and phenylboronic acid (54.9 mg, 0.450 mmol). The mixture was purified by column chromatography (5% EtOAc/*iso*-hexane) to give *allylation product* **3d** (110 mg, 70%) as a pale yellow solid. R$_f$ = 0.27 (15% Et$_2$O/ *iso*-hexane); m.p. 105-106 °C (Et$_2$O); $[\alpha]_D^{24.7}$ −119.2 (*c* 0.39, CHCl$_3$); IR 3297 (N-H), 2919, 1448, 1163, 825, 693 cm^{-1}; ^{1}H NMR (400 MHz, CDCl$_3$) δ 7.46-7.42 (2H, m, Ar**H**) 7.38-7.28 (3H, m, Ar**H**), 7.26-7.14 (5H, m, Ar**H**) 7.11-7.06 (2H, m, Ar**H**), 6.95-6.91 (1H, m, Ar**H**), 5.48 (1H, d, J = 9.8 Hz, C=C**H**), 5.06 (1H, s, C=C**H$_A$**H$_B$), 4.99-4.97 (1H, m, C=CH$_A$**H$_B$**), 4.94 (1H, d, J = 6.8 Hz, C**H**NH), 3.87 (1H, br s, N**H**), 3.49 (1H, dd, J = 9.8, 6.8 Hz, C=CC**H**), 2.79 (2H, dd, J = 8.1, 6.7 Hz, PhC**H$_2$**), 2.65 (1H, dt, J = 13.7, 6.7 Hz, PhCH$_2$C**H$_A$**H$_B$), 2.46 (1H dt, J = 13.7, 8.1 Hz, PhCH$_2$CH$_A$**H$_B$**), 1.89 (3H, s, C**H$_3$**); ^{13}C NMR (101 MHz, CDCl$_3$) δ 150.4 (C), 143.3 (C), 143.2 (C), 141.6 (C), 141.4 (C), 132.3 (CH), 129.7 (CH), 128.54 (2 × CH), 128.51 (2 × CH), 128.4 (2 × CH), 127.7 (CH), 126.7 (2 × CH), 126.4 (CH), 124.4 (CH), 124.3 (C), 120.5 (CH), 117.8 (C), 115.5 (CH$_2$), 56.6 (CH), 49.2 (CH), 33.7 (CH$_2$), 31.8 (CH$_2$), 21.2 (CH$_3$); HRMS (ESI) Exact mass calcd for [$C_{27}H_{26}{}^{79}BrNNaO_3S$]$^+$ [M+Na]$^+$: 546.0709, found: 546.0699; Enantiomeric excess was determined by HPLC using a Chiralcel OD-H column (95:5 *iso*-hexane:*i*-PrOH, 0.8 mL/min, 254 nm): t_r (major) = 16.3 min, t_r (minor) = 24.5 min, 99% ee.

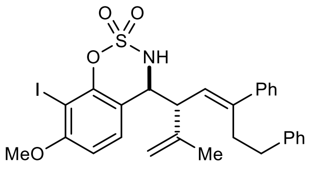

(4*S*)-8-Iodo-7-methoxy-4-[(3*R*,*E*)-2-methyl-5,7-diphenylhepta-1,4-dien-3-yl]-3,4-dihydro-1,2λ^6,3-benzoxathiazine-2,2-dione (3e). A modification of General Procedure A was followed using imine **1e** (67.8 mg, 0.200 mmol), 1,3-enyne **2a** (44.2 mg, 0.240 mmol),

phenylboronic acid (36.6 mg, 0.300 mmol), KF (17.4 mg, 0.300 mmol), *t*-amyl alcohol (0.03 mL, 0.30 mmol), $[Rh(C_2H_4)_2Cl]_2$ (1.9 mg, 0.0050 mmol) **L2** (4.0 mg, 0.010 mmol), $ZnCl_2$ solution (0.7 M in THF, 0.29 mL, 0.20 mmol), and THF (1.0 mL). The mixture was purified by column chromatography (10% EtOAc/*iso*-hexane) to give *allylation product* **3e** (74 mg, 62%) as an off-white solid. R_f = 0.24 (20% EtOAc/petroleum ether); m.p. 151-153 °C (Et_2O); $[\alpha]_D^{25.0}$ –18.6 (*c* 0.30, $CHCl_3$); IR 3306 (N-H), 2923, 1451, 1186, 1170, 697 cm^{-1}; ^1H NMR (400 MHz, $CDCl_3$) δ 7.39-7.28 (3H, m, Ar**H**), 7.26-7.21 (2H, m, Ar**H**), 7.21-7.14 (3H, m, Ar**H**) 7.08 (1H, dd, *J* = 8.7, 0.9 Hz, Ar**H**), 7.05-7.01 (2H, m, Ar**H**) 6.61 (1H, d, *J* = 8.7 Hz, Ar**H**), 5.49 (1H, d, *J* = 9.5 Hz, C=C**H**), 5.04 (1H, s, C=C$\mathbf{H_A}H_B$), 4.96 (1H, s, C=C$H_A\mathbf{H_B}$), 4.90 (1H, dd, *J* = 7.8, 6.9 Hz, C**H**NH), 3.92 (3H, s, OC$\mathbf{H_3}$), 3.81 (1H, d, *J* = 7.8 Hz, N**H**), 3.44 (1H, dd, *J* = 9.5, 6.9 Hz, C=CC**H**), 2.83-2.74 (2H, m, PhC$\mathbf{H_2}$), 2.71-2.61 (1H, m, PhCH$_2$C$\mathbf{H_A}H_B$), 2.41 (1H, dt, *J* = 13.6, 8.1 Hz, PhCH$_2$C$H_A\mathbf{H_B}$), 1.89 (3H, s, CC$\mathbf{H_3}$); ^{13}C NMR (101 MHz, $CDCl_3$) δ 159.2 (C), 151.5 (C), 143.5 (C), 142.8 (C), 141.5 (C), 141.4 (C), 128.5 (4 × CH), 128.4 (2 × CH), 127.6 (CH), 127.4 (CH), 126.7 (2 × CH), 126.4 (CH), 125.1 (CH), 116.0 (C), 115.1 (CH_2), 107.3 (CH), 78.5 (C), 56.7 (CH_3), 56.6 (CH), 49.4 (CH), 33.7 (CH_2), 31.7 (CH_2), 21.3 (CH_3); HRMS (ESI) Exact mass calcd for $[C_{28}H_{28}INNaO_4S]^+$ $[M+Na]^+$: 624.0676, found: 624.0660; Enantiomeric excess was determined by HPLC using a Chiralcel OD-H column (90:10 *iso*-hexane:*i*-PrOH, 1.0 mL/min, 254 nm): t_r (major) = 12.6 min, t_r (minor) = 18.5 min, 99% ee.

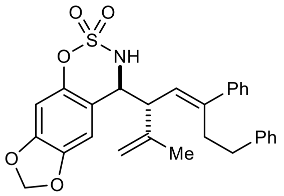

(13*S*)-13-[(3*R*,*E*)-2-Methyl-5,7-diphenylhepta-1,4-dien-3-yl]-4,6,10-trioxa-11λ^6-thia-12-azatricyclo[7.4.0.0^{3,7}]trideca-1,3(7),8-triene-11,11-dione (3f). General Procedure A was followed using imine **1f** (68.2 mg, 0.300 mmol), 1,3-enyne **2a** (66.3 mg, 0.360 mmol), and phenylboronic acid (54.9 mg, 0.450 mmol). The mixture was purified by column chromatography (10% EtOAc/petroleum ether) to give *allylation product* **3f** (76.0 mg, 52%) as a dark orange solid (small quantities of unidentified impurities and silicone grease remained). R_f = 0.43 (20% EtOAc/petroleum ether); m.p. 108-109 °C (Et_2O); $[\alpha]_D^{25.2}$ –26.2 (*c* 0.48, $CHCl_3$); IR 3291 (N-H), 2916, 1482, 1200, 1135, 1032, 697 cm^{-1}; ^1H NMR (400 MHz, $CDCl_3$) δ 7.40-7.15 (8H, m, Ar**H**), 7.10-7.04 (2H, m, Ar**H**), 6.62-6.59 (1H, m, Ar**H**), 6.55 (1H, s, Ar**H**), 6.00 (1H, d, *J* = 1.3 Hz, C$\mathbf{H_A}H_B$O), 5.98 (1H, d, *J* = 1.3 Hz, C$H_A\mathbf{H_B}$O), 5.54 (1H, d, *J* = 9.5 Hz, C=C**H**), 5.06-5.03 (1H, m, C=C$\mathbf{H_A}H_B$), 4.98-4.95 (1H, m, C=C$H_A\mathbf{H_B}$), 4.84 (1H, dd, *J* = 7.2, 6.4 Hz, C**H**NH), 3.94 (1H, d, *J* = 7.2 Hz, N**H**), 3.44 (1H, dd, *J* = 9.5, 6.4 Hz, C=CC**H**), 2.80-2.73 (2H, m, PhC$\mathbf{H_2}$), 2.69-2.59 (1H, m, PhCH$_2$C$\mathbf{H_A}H_B$), 2.41 (1H, dt, *J* = 13.7, 8.2 Hz PhCH$_2$C$H_A\mathbf{H_B}$), 1.88 (3H, s, CC$\mathbf{H_3}$); ^{13}C NMR (126 MHz, $CDCl_3$) δ 147.8 (C), 145.8 (C), 145.1 (C), 143.5 (C), 143.0 (C) , 141.6 (C), 141.5 (C), 128.5 (4 × CH), 128.3 (2 × CH), 127.6 (CH), 126.6 (2 × CH), 126.4 (CH), 124.7 (CH), 115.3 (CH_2), 114.6 (C), 105.3 (CH), 102.0 (CH_2),

100.5 (CH), 56.9 (CH), 49.4 (CH), 33.8 (CH_2), 31.9 (CH_2), 21.4 (CH_3); HRMS (ESI) Exact mass calcd for $[C_{28}H_{27}NNaO_5S]^+$ $[M+Na]^+$: 512.1502, found: 512.1509; Enantiomeric excess was determined by HPLC using a Chiralcel OD-H column (90:10 *iso*-hexane:*i*-PrOH, 1.0 mL/min, 254 nm): t_r (major) = 12.6 min, t_r (minor) = 16.6 min, 99% ee.

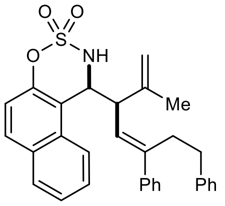

(1*S*)-1-[(3*R*,*E*)-2-Methyl-5,7-diphenylhepta-1,4-dien-3-yl]-1H,2H-4,3λ^6,2-naphtho[1,2-e][1,2λ^6,3]oxathiazine-3,3-dione (3g). General Procedure A was followed using imine **1g** (70.0 mg, 0.300 mmol), 1,3-enyne **2a** (66.3 mg, 0.360 mmol), and phenylboronic acid (54.9 mg, 0.450 mmol). The mixture was purified by column chromatography (10% EtOAc/petroleum ether) to give *allylation product* **3g** (79.0 mg, 53%) as an orange oil (*ca*. 95% purity; small quantities of unidentified impurities and silicone grease remained). R_f = 0.34 (15% EtOAc/petroleum ether); $[\alpha]_D^{24.8}$ –170.3 (*c* 0.47, $CHCl_3$); IR 3306 (N-H), 2926, 1451, 1184, 1170, 696 cm^{-1}; ^{1}H NMR (400 MHz, $CDCl_3$) δ 7.97 (1H, d, *J* = 8.6 Hz, Ar**H**), 7.89 (1H, d, *J* = 8.2 Hz, Ar**H**), 7.85 (1H, d, *J* = 8.9 Hz, Ar**H**), 7.70 (1H, t, *J* = 7.8 Hz, Ar**H**), 7.55 (1H, t, *J* = 7.5 Hz, Ar**H**), 7.36-7.22 (4H, m, Ar**H**), 7.20-7.01 (5H, m, Ar**H**), 6.65-6.60 (2H, m, Ar**H**), 5.84 (1H, dd, *J* = 3.6, 2.8 Hz, C**H**NH), 5.72 (1H, d, *J* = 10.0 Hz, C=C**H**), 5.14 (1H, d, *J* = 1.5 Hz, C=C$\mathbf{H_A}$$H_B$), 5.10-5.04 (2H, m, C=C$H_A$$\mathbf{H_B}$ and N**H**), 4.09-4.00 (1H, m, C=CC**H**), 2.10-1.88 (6H, m, PhC$\mathbf{H_2}$C$\mathbf{H_A}$$H_B$ and C$\mathbf{H_3}$), 1.23-1.13 (1H, m, PhCH_2C$H_A$$\mathbf{H_B}$); ^{13}C NMR (101 MHz, $CDCl_3$) δ 149.5 (C), 145.2 (C), 142.9 (C), 141.8 (C), 141.3 (2 × C), 131.8 (C), 130.7 (CH), 129.7 (CH), 128.4 (2 × CH), 128.1 (2 × CH), 127.9 (CH), 127.8 (2 × CH), 127.4 (CH), 126.4 (2 × CH), 125.9 (CH), 125.7 (CH), 121.9 (CH), 121.4 (CH), 118.6 (CH), 116.4 (C), 116.2 (CH_2), 56.2 (CH), 48.8 (CH), 33.9 (CH_2), 32.0 (CH_2), 21.9 (CH_3); HRMS (ESI) Exact mass calcd for $[C_{31}H_{29}NNaO_3S]^+$ $[M+Na]^+$: 518.1760, found: 518.1757; Enantiomeric excess was determined by HPLC using a Chiralcel OD-H column (90:10 *iso*-hexane:*i*-PrOH, 1.0 mL/min, 210 nm): t_r (major) = 12.2 min, t_r (minor) = 28.9 min, 99% ee.

3-Methyl-3-[(3*S*,*E*)-2-methyl-5,7-diphenylhepta-1,4-dien-3-yl]-2,3-dihydro-1λ^6,2-benzothiazole-1,1-dione (6)

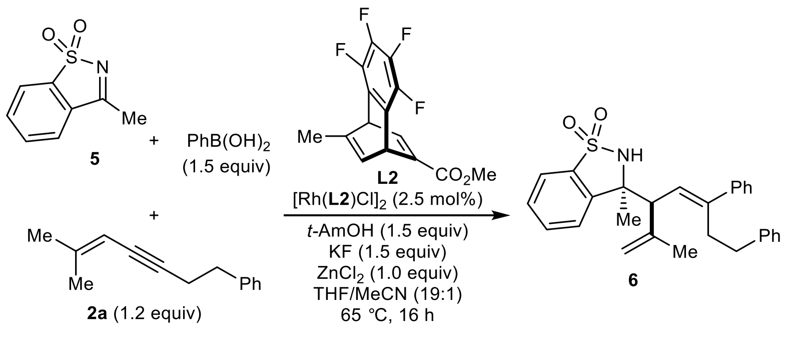

$[Rh(C_2H_4)_2Cl]_2$ (2.9 mg, 0.0075 mmol, 2.5 mol%) and **L2** (4.5 mg, 0.015 mmol, 5 mol%) were added to an oven-dried microwave vial and purged with nitrogen for 1 h. Degassed anhydrous THF (1.4 mL) and degassed anhydrous MeCN (0.1 mL) were added and the mixture stirred for 30 min at room temperature. Imine **5** (54.3 mg, 0.300 mmol), 1,3-enyne **2a** (66.3 mg, 0.360 mmol), phenylboronic acid (54.9 mg, 0.450 mmol), and KF (26.1 mg, 0.450 mmol) were added to a separate oven-dried microwave vial. The vial was purged with nitrogen for 1 h, and the catalyst solution was then added to the vial containing the imine. *t*-Amyl alcohol (0.05 mL, 0.45 mmol) and $ZnCl_2$ solution (0.7 M in THF, 0.43 mL, 0.30 mmol) were added and the reaction mixture was stirred for at 65 °C for 16 h. The mixture was cooled to room temperature, diluted with saturated aqueous NH_4Cl solution (5 mL) and water (5 mL), and extracted with EtOAc (3 × 10 mL). The combined organic phases were washed with brine, dried (Na_2SO_4), filtered, and concentrated *in vacuo*. The mixture was purified by column chromatography (10% EtOAc/petrol) to give *allylation product* **6** (80.0 mg) as an orange solid in *ca.* 85% purity as determined by ^{1}H NMR analysis, and in 69% ee. This material was further purified by the following procedure: (i) suspending it in hot pentane (*ca.* 3 mL), followed by the addition of toluene (*ca.* 0.1 mL); (ii) agitation of the mixture; (iii) removal of the solvent with a pipette, and (iv) washing the residue with additional pentane. This sequence gave *allylation product* **6** (33 mg, 25%) as an orange solid in 93% ee. Combining all of the washes from this trituration procedure and concentration *in vacuo* gave a second batch of material (45 mg) in *ca.* 60% purity as a yellow solid, which was not purified further. R_f = 0.37 (20% EtOAc/petroleum ether); m.p. 172-174 °C (Et_2O); $[\alpha]^{25.0}_{D}$ –13.8 (*c* 0.58, $CHCl_3$); IR 3206 (N-H), 2923, 1445, 1274, 1131, 767, 700 cm^{-1}; ^{1}H NMR (400 MHz, $CDCl_3$) δ 7.77-7.73 (1H, m, Ar**H**), 7.61-7.55 (1H, td, *J* = 7.6, 1.3 Hz, Ar**H**), 7.54-7.48 (1H, td, *J* = 7.6, 1.1 Hz, Ar**H**), 7.43-7.38 (3H, m, Ar**H**), 7.37-7.31 (2H, m, Ar**H**), 7.29-7.23 (3H, m, Ar**H**), 7.21-7.15 (1H, m, Ar**H**), 7.09-7.03 (2H, m, Ar**H**), 5.85 (1H, d, *J* = 10.3 Hz, C=C**H**), 4.96-4.93 (1H, m, C=C$\mathbf{H_A}$$H_B$), 4.87 (1H, br s, C=C$H_A$$\mathbf{H_B}$), 4.63 (1H, s, N**H**), 3.52 (1H, d, *J* = 10.3 Hz, C=CC**H**), 2.64-2.48 (3H, m, PhC$\mathbf{H_2}$C$\mathbf{H_A}$$H_B$), 2.35 (1H, dt, *J* = 13.2, 7.7 Hz, PhCH_2C$H_A$$\mathbf{H_B}$), 1.71 (3H, s, CH_2=CC$\mathbf{H_3}$), 1.53 (3H, s, NHCC$\mathbf{H_3}$); 13C NMR (126 MHz, CDCl3) δ 144.1 (C), 143.4 (C), 143.1 (C), 142.3 (C), 141.5 (C), 134.9 (C), 132.4 (CH), 129.3 (CH), 128.6 (2 × CH), 128.4 (4 × CH), 127.5 (CH), 126.6 (2 × CH), 126.1 (CH), 125.9 (CH), 124.5 (CH), 121.3 (CH), 117.1 (CH_2), 65.7 (C), 54.0 (CH), 34.1 (CH_2), 32.2 (CH_2), 26.4 (CH_3), 22.6 (CH_3); HRMS (ESI) Exact mass calcd for $[C_{28}H_{29}NNaO_2S]^+$ $[M+Na]^+$: 466.1811, found: 466.1811;

Enantiomeric excess was determined by HPLC using a Chiralcel AD-H column (90:10 *iso*-hexane:*i*-PrOH, 0.8 mL/min, 254 nm).

First batch (before trituration): t_r (minor) = 14.3 min, t_r (major) = 21.5 min, 69% ee.

Second batch (after trituration): t_r (minor) = 14.2 min, t_r (major) = 21.0, 93% ee.

Crystals suitable for X-ray analysis were prepared by slow diffusion of petroleum ether into a solution of **6** in toluene.

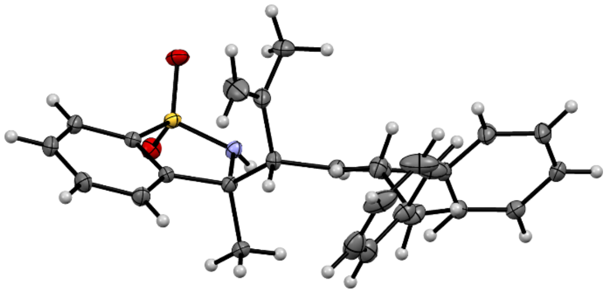

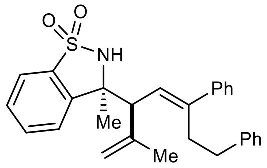

6
CCDC 1552183
Flack parameter = 0.03(3)

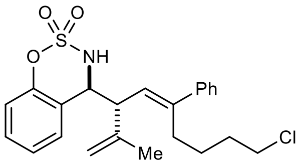

(4*S*)-4-[(3*R*,*E*)-9-Chloro-2-methyl-5-phenylnona-1,4-dien-3-yl]-3,4-dihydro-1,2λ^6,3-benzoxathiazine-2,2-dione (3h). A modification of General Procedure A was followed using imine **1a** (55.3 mg, 0.302 mmol), 1,3-enyne **2b** (76.8 mg, 0.450 mmol), and phenylboronic acid (54.9 mg, 0.450 mmol). The mixture was purified by column chromatography (10% EtOAc/petrol) to give *allylation product* **3h** (97.0 mg, 77%) as a pale yellow oil (*ca*. 95% purity; small quantities of unidentified impurities and silicone grease remained). R_f = 0.24 (15% EtOAc/petrol); $[\alpha]_D^{23.7}$ −48.4 (*c* 0.91, $CHCl_3$); IR 3275 (N-H), 2948, 1369, 1167, 756 cm^{-1}; ^1H NMR (400 MHz, $CDCl_3$) δ 7.36-7.27 (1H, m, Ar**H**), 7.28-7.18 (5H, m, Ar**H**), 7.07-7.02 (3H, m, Ar**H**), 5.50 (1H, d, *J* = 9.5 Hz, C=C**H**), 5.15-5.08 (2H, m, C**H**NH and C=C**H_A**H_B), 4.99 (1H, s, C=CH_A**H_B**), 4.76 (1H, d, *J* = 5.6 Hz, N**H**), 3.81 (1H, dd, *J* = 9.5, 4.9 Hz, C=CC**H**), 3.45 (1H, ddd, *J* = 14.1, 10.8, 6.7 Hz, C**H_A**H_BCl), 3.44 (1H, ddd, *J* = 14.1, 10.8, 6.6 Hz, CH_A**H_B**Cl), 2.40 (1H, ddd, *J* = 14.1, 9.5, 6.1 Hz, C=CC**H_A**H_B), 2.32 (1H, ddd, *J* = 14.1, 9.3, 6.3 Hz, C=CCH_A**H_B**) 1.97 (3H, s, C**H_3**), 1.73-1.60 (2H, m, C**H_2**CH_2Cl), 1.40-1.23 (2H, m, C=CCH_2C**H_2**); ^{13}C NMR (101 MHz, $CDCl_3$) δ 151.3 (C), 144.8 (C), 143.0 (C), 141.8 (C), 129.4 (CH), 128.3 (2 × CH), 127.4 (CH), 126.9 (CH), 126.4 (2 × CH), 125.4 (CH), 123.2 (CH), 121.9 (C), 119.1 (CH), 116.1 (CH_2), 57.5 (CH), 49.3 (CH), 44.6 (CH_2), 32.3 (CH_2), 29.5 (CH_2), 25.1 (CH_2), 21.6 (CH_3); HRMS (ESI) Exact mass calcd for $[C_{23}H_{26}{}^{35}ClNNaO_3S]^+$ $[M+Na]^+$: 454.1214, found: 454.1219; Enantiomeric excess was determined by HPLC using a Chiralcel OD-H column (90:10 *iso*-hexane:*i*-PrOH, 1.5 mL/min, 254 nm): t_r (major) = 5.2 min, t_r (minor) = 11.8 min, 98% ee.

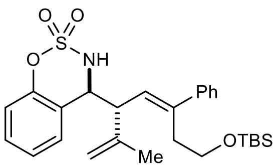

(4*S*)-4-[(3*R*,*E*)-7-[(*tert*-Butyldimethylsilyl)oxy]-2-methyl-5-phenylhepta-1,4-dien-3-yl]-3,4-dihydro-1,2λ^6,3-benzoxathiazine-2,2-dione (3i). General Procedure A was followed using imine **1a** (54.8 mg, 0.299 mmol), 1,3-enyne **2c** (85.8 mg, 0.360 mmol), and phenylboronic acid (54.9 mg, 0.450 mmol). The mixture was purified by column chromatography (15% EtOAc/petrol) to give *allylation product* **3i** (68.8 mg, 46%) as a pale yellow oil. R_f = 0.13 (10% EtOAc/petrol); $[\alpha]_D^{23.8}$ −23.6

(*c* 0.67, $CHCl_3$); IR 3281 (N-H), 2928, 1370, 1170, 832, 755 cm^{-1}; ^{1}H NMR (400 MHz, $CDCl_3$) δ 7.37-7.21 (5H, m, Ar**H**), 7.20-7.11 (3H, m, Ar**H**), 7.04 (1H, dd, *J* = 8.1, 1.2 Hz, Ar**H**), 5.59 (1H, d, *J* = 9.8 Hz, C=C**H**), 5.13 (1H, d, *J* = 6.8, 6.2 Hz, C**H**NH), 5.09 (1H, s, C=C**H$_A$**H$_B$), 5.01 (1H, s, C=CH$_A$**H$_B$**), 5.00 (1H, d, *J* = 6.8 Hz, N**H**) 3.85 (1H, dd, *J* = 9.8, 6.2 Hz, C**H**C=C), 3.53 (1H, dt, *J* = 10.1, 6.6 Hz, OC**H$_A$**H$_B$), 3.43 (1H, dt, *J* = 10.1, 7.2 Hz, OCH$_A$**H$_B$**), 2.65 (2H, dd, *J* = 7.2, 6.6 Hz, OCH$_2$C**H$_2$**), 1.96 (3H, s, C=CC**H$_3$**), 0.81 (9H, s, C(C**H$_3$**)$_3$), –0.05 (3H, s, SiC**H$_3$**), –0.07 (3H, s, SiC**H$_3$**); ^{13}C NMR (101 MHz, $CDCl_3$) δ 151.5 (C), 143.4 (C), 141.9 (C), 141.2 (C), 129.4 (CH), 128.3 (2 × CH), 127.4 (CH), 126.8 (CH), 126.5 (2 × CH), 125.6 (CH), 125.2 (CH), 122.3 (C), 118.9 (CH), 115.5 (CH_2), 61.8 (CH_2), 57.1 (CH), 49.2 (CH), 34.1 (CH_2), 26.0 (3 × CH_3), 21.6 (CH_3), 18.5 (C), −5.4 (CH_3), −5.5 (CH_3); HRMS (ESI) Exact mass calcd for $[C_{27}H_{37}NNaO_4SSi]^+$ $[M+Na]^+$: 522.2105 found: 522.2104; Enantiomeric excess was determined by HPLC using a Chiralcel OD-H column (90:10 *iso*-hexane:*i*-PrOH, 1.5 mL/min, 254 nm): t_r (major) = 3.2 min, t_r (minor) = 5.7 min, 99% ee.

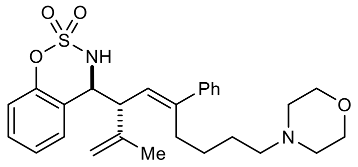

(4*S*)-4-[(3*R*,*E*)-2-Methyl-9-(morpholin-4-yl)-5-phenylnona-1,4-dien-3-yl]-3,4-dihydro-1,2λ^6,3-benzoxathiazine-2,2-dione (3j). General Procedure A was followed using imine **1a** (54.9 mg, 0.300 mmol), 1,3-enyne **2d** (79.7 mg, 0.360 mmol), and phenylboronic acid (54.9 mg, 0.450 mmol). The mixture was purified by column chromatography (1% Et_3N/EtOAc) to give *allylation product* **3j** (103 mg, 71%) as a yellow oil as an 8:1 ratio of diastereomers. R_f = 0.26 (99% EtOAc/1% Et_3N); $[\alpha]_D^{25.1}$ –75.3 (*c* 0.48, $CHCl_3$); IR 3199 (N-H), 2957, 1363, 1051, 817, 745 cm^{-1}; ^{1}H NMR (400 MHz, $CDCl_3$) δ 7.34-7.22 (5H, m, Ar**H**), 7.15 (3H, tt, *J* = 7.8, 1.4 Hz, Ar**H**), 7.08-7.04 (1H, m, Ar**H**), 5.54 (1H, d, *J* = 9.6 Hz, C=C**H**), 5.08 (1H, d, *J* = 6.4 Hz, C**H**NH) 5.06 (1H, br s, C=C**H$_A$**H$_B$), 4.99 (1H, br s, C=CH$_A$**H$_B$**), 3.76 (1H, dd, *J* = 9.6, 6.4 Hz, C=CC**H**), 3.62 (2H, ddd, *J* = 11.4, 6.3, 3.1 Hz, OC**H$_2$**), 3.55 (2H, ddd, *J* = 11.4, 6.3, 3.1 Hz, OC**H$_2$**), 2.45 (2H, dd, *J* = 8.4, 6.6 Hz, C=CC**H$_2$**), 2.40-2.08 (6H, m, 3 × NC**H$_2$**), 1.94 (3H, s, C**H$_3$**), 1.45-1.34 (2H, m, C=CCH$_2$CH$_2$C**H$_2$**), 1.29-1.17 (2H, m, C=CCH$_2$C**H$_2$**), the N**H** proton was not observed; ^{13}C NMR (101 MHz, $CDCl_3$) δ 151.8 (C), 144.0 (C), 143.3 (C), 141.9 (C), 129.4 (CH), 128.3 (2 × CH), 127.3 (CH), 126.6 (CH), 126.5 (2 × CH), 125.1 (CH), 124.5 (CH), 123.0 (C), 118.9 (CH), 115.2 (CH_2), 66.6 (2 × CH_2), 58.3 (CH_2), 57.2 (CH), 53.7 (2 × CH_2), 48.9 (CH), 29.2 (CH_2), 25.4 (CH_2), 25.0 (CH_2), 21.5 (CH_3); HRMS (ESI) Exact mass calcd for $[C_{27}H_{35}N_2O_4S]^+$ $[M+H]^+$: 483.2312, found: 483.2315; Enantiomeric excess was determined by HPLC using a Chiralcel AD-H column (90:10 *iso*-hexane:*i*-PrOH, 1.5 mL/min, 210 nm): t_r (major) = 6.2 min, t_r (major) = 7.1 min, 99% ee.

Characteristic ^{1}H NMR signals of minor diastereomer:

^{1}H NMR (400 MHz, $CDCl_3$) δ 6.62 (1H, dd, *J* = 7.9, 1.4 Hz, Ar**H**), 5.39 (1H, d, *J* = 10.4 Hz, C=C**H**), 5.14 (1H, q, *J* = 1.4 Hz, C=C**H**$_2$), 4.92 (1H, d, *J* = 4.4 Hz, C**H**NH), 3.40 (1H, dd, *J* = 10.4, 4.4 Hz, C=CC**H**).

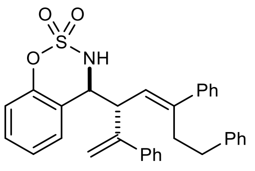

(4*S*)-4-[(3*R*,*E*)-2,5,7-Triphenylhepta-1,4-dien-3-yl]-3,4-dihydro-1,2λ^6,3-benzoxathiazine-2,2-dione (3k). A modification of General Procedure A was followed using imine **1a** (55.0 mg, 0.300 mmol), 1,3-enyne **2e** (88.6 mg, 0.360 mmol), phenylboronic acid (110 mg, 0.900 mmol) and *t*-amyl alcohol (0.10 mL, 0.90 mmol). The mixture was purified by column chromatography (10% EtOAc/petrol, followed by 70:28:2 petroleum ether/CH_2Cl_2/EtOAc), to give *allylation product* **3k** (75.0 mg, 49%) as a pale brown solid. R_f = 0.23 (10% EtOAc/petroleum ether); m.p. 113-114 °C (Et_2O); $[\alpha]_D^{25.4}$ +86.2 (*c* 0.39, $CHCl_3$); IR 3307 (N-H), 3024, 1422, 1198, 1169, 697 cm^{-1}; ^{1}H NMR (400 MHz, $CDCl_3$) δ 7.48-7.36 (5H, m, Ar**H**), 7.36-7.21 (7H, m, Ar**H**), 7.19-7.06 (5H, m, Ar**H**), 7.03 (1H, dd, *J* = 8.3, 1.2 Hz, Ar**H**), 6.95 (1H, d, *J* = 7.8 Hz, Ar**H**), 5.60 (1H, d, *J* = 8.6 Hz, C=C**H**), 5.49 (1H, s, C=C**H$_A$**H$_B$), 5.32 (1H, s, C=CH$_A$**H$_B$**), 4.81 (1H, dd, *J* = 6.3, 4.7 Hz, C**H**NH), 4.52 (1H, d, *J* = 6.3 Hz, N**H**) 4.28 (1H, dd, *J* = 8.6, 4.7 Hz, C=CC**H**), 2.82-2.69 (2H, m, PhC**H**$_2$), 2.65-2.54 (1H, m, PhCH$_2$C**H$_A$**H$_B$) 2.43 (1H, dt, *J* = 13.7, 8.2 Hz, PhCH$_2$CH$_A$**H$_B$**); ^{13}C NMR (101 MHz, $CDCl_3$) δ 151.3 (C), 147.5 (C), 144.5 (C), 141.59 (C), 141.56 (C), 140.1 (C), 129.2 (CH), 128.9 (2 × CH), 128.51 (2 × CH), 128.45 (2 × CH), 128.4 (CH), 128.3 (2 × CH), 127.6 (CH), 126.62 (3 × CH), 126.56 (2 × CH), 126.2 (CH), 125.5 (CH), 123.2 (CH), 121.8 (C), 118.9 (CH), 117.7 (CH_2), 57.4 (CH), 47.1 (CH), 33.8 (CH_2), 32.5 (CH_2); HRMS (ESI) Exact mass calcd for $[C_{32}H_{29}NNaO_3S]^+$ $[M+Na]^+$: 530.1760, found: 530.1757; Enantiomeric excess was determined by HPLC using a Chiralcel OD-H column (90:10 *iso*-hexane:*i*-PrOH, 1.0 mL/min, 254 nm): t_r (major) = 11.8 min, t_r (minor) = 14.6 min, >99% ee.

±(4*S*)-4-[(3*S*,*E*)-5,7-Diphenylhepta-1,4-dien-3-yl]-3,4-dihydro-1,2λ^6,3-benzoxathiazine-2,2-dione (3l)

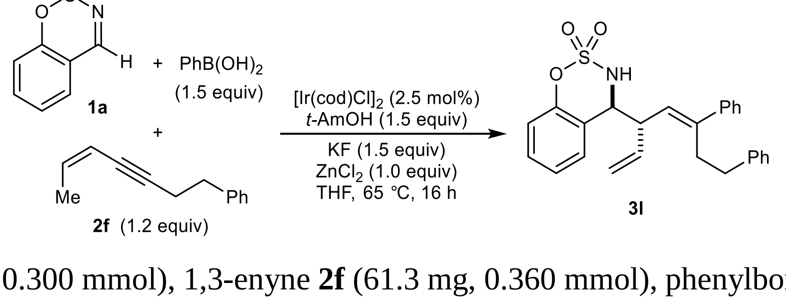

Imine **1a** (55.0 mg, 0.300 mmol), 1,3-enyne **2f** (61.3 mg, 0.360 mmol), phenylboronic acid (110 mg, 0.900 mmol), KF (26.1 mg, 0.450 mmol), and [Ir(cod)Cl]$_2$ (5.0 mg, 0.0075 mmol, 2.5 mol%) were added to an oven-dried microwave vial. The vial was purged with nitrogen for 1 h, and degassed

anhydrous THF (1.5 mL) was added, followed by *t*-amyl alcohol (0.05 mL, 0.45 mmol) and $ZnCl_2$ solution (0.7 M in THF, 0.43 mL, 0.30 mmol). The mixture was stirred at 65 °C for 16 h and subsequently cooled to room temperature. The mixture was diluted with saturated aqueous NH_4Cl solution (5 mL) and water (5 mL), and extracted with EtOAc (3 × 10 mL). The combined organic phases were washed with brine, dried (Na_2SO_4), filtered, and concentrated *in vacuo*. The crude residue was purified by column chromatography (10% EtOAc/petroleum ether) to give *allylation product* **3l** (117 mg, 90%) as an orange oil (*ca.* 93% purity; small quantities of solvents, unidentified impurities, and silicone grease remained). R_f = 0.19 (10% EtOAc/petroleum ether); IR 3272 (N-H), 2926, 1485, 1451, 1416, 1168, 755, 698 cm^{-1}; ^1H NMR (400 MHz, $CDCl_3$) δ 7.38-7.11 (11H, m, Ar**H**) 7.09-7.05 (2H, m, Ar**H**), 7.02 (1H, d, *J* = 8.2, 1.2, Ar**H**) 5.86 (1H, ddd, *J* = 17.0, 10.3, 6.4 Hz, CH_2=C**H**), 5.48 (1H, d, *J* = 9.2 Hz, C=C**H**), 5.26 (1H, dt, *J* = 10.3, 1.3 Hz, C=C**H**$_A$H$_B$), 5.18 (1H, dt, *J* = 17.0, 1.3 Hz, C=CH$_A$**H**$_B$), 4.83 (1H, dd, *J* = 7.7, 5.7 Hz, C**H**NH), 4.23 (1H, d, *J* = 7.7 Hz, N**H**), 3.73-3.65 (1H, m, C=CC**H**), 2.88-2.73 (2H, m, PhC**H**$_2$), 2.68-2.58 (1H, m, PhCH$_2$C**H**$_A$H$_B$), 2.47 (1H, dt, *J* = 13.7, 8.0 Hz, PhCH$_2$CH$_A$**H**$_B$); ^{13}C NMR (101 MHz, $CDCl_3$) δ 151.5 (C), 143.6 (C), 141.6 (C), 141.4 (C), 136.2 (CH), 129.4 (CH), 128.5 (2 × CH), 128.43 (2 × CH), 128.42 (2 × CH) 127.6 (CH), 126.9 (CH), 126.7 (2 × CH), 126.2 (CH), 125.1 (CH), 123.9 (CH), 121.6 (C), 118.9 (CH), 118.6 (CH_2), 59.4 (CH), 46.1 (CH), 33.8 (CH_2), 32.0 (CH_2); HRMS (ESI) Exact mass calcd for $[C_{26}H_{25}NNaO_3S]^+$ $[M+Na]^+$: 454.1447, found: 454.1444.

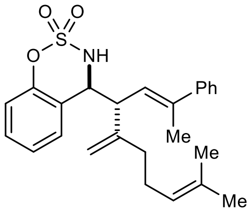

(4*S*)-4-[(*E*,4*R*)-9-Methyl-5-methylidene-2-phenyldeca-2,8-dien-4-yl]-3,4-dihydro-1,2λ^6,3-benzoxathiazine-2,2-dione (3m). A modification of General Procedure A was followed using imine **1a** (54.9 mg, 0.300 mmol), 1,3-enyne **2g** (73.0 mg, 0.450 mmol, *E*/*Z* = 5.8:1), phenylboronic acid (73.2 mg, 0.600 mmol), KF (34.9 mg, 0.600 mmol), and *t*-amyl alcohol (0.07 mL, 0.60 mmol). The mixture was purified by column chromatography (10% EtOAc/petrol) to give *allylation product* **3m** (67.2 mg, 53%) as a yellow oil (*ca.* 95% purity; small quantities of solvents, unidentified impurities, and silicone grease remained). R_f = 0.17 (10% EtOAc/petrol); $[\alpha]_D^{23.7}$ –23.6 (*c* 0.51, $CHCl_3$); IR 3274 (N-H), 2970, 1370, 1169, 755 cm^{-1}; ^1H NMR (400 MHz, $CDCl_3$) δ 7.31-7.10 (8H, m, Ar**H**), 7.03 (1H, dd, *J* = 8.2, 1.2 Hz, Ar**H**), 5.61 (1H, dq, *J* = 9.3, 1.4 Hz, PhC=C**H**), 5.21-5.15 (1H, m, $(CH_3)_2$C=C**H**), 5.14 (1H, dd, *J* = 5.6, 4.7 Hz, C**H**NH), 5.13 (1H, s, C=C**H**$_A$H$_B$), 5.06 (1H, br s, C=CH$_A$**H**$_B$), 4.78 (1H, d, *J* = 5.6 Hz, N**H**), 3.88 (1H, dd, *J* = 9.3, 4.7 Hz, C=CC**H**), 2.34-2.19 (4H, m, C**H**$_2$C**H**$_2$), 1.90 (3H, d, *J* = 1.4 Hz, C=C(Ph)C**H**$_3$), 1.73 (3H, s, (C**H**$_3$)$_2$C=C), 1.67 (3H, s, (C**H**$_3$)$_2$C=C); ^{13}C NMR (101 MHz, $CDCl_3$) δ 151.3 (C), 146.5 (C), 142.9 (C), 139.9 (C), 132.6 (C), 129.3 (CH), 128.2 (2 × CH), 127.3 (CH), 126.7 (CH), 125.8 (2 × CH), 125.4 (CH), 123.4 (CH), 122.5 (CH), 122.0 (C), 119.0

(CH), 114.6 (CH_2), 57.6 (CH), 48.0 (CH), 34.8 (CH_2), 26.4 (CH_2), 25.7 (CH_3), 17.9 (CH_3), 16.6 (CH_3); HRMS (ESI) Exact mass calcd for $[C_{25}H_{29}NNaO_3S]^+$ $[M+Na]^+$: 446.1760, found: 446.1762; Enantiomeric excess was determined by HPLC using a Chiralcel OD-H column (90:10 *iso*-hexane:*i*-PrOH, 1.5 mL/min, 254 nm): t_r (major) = 3.7 min, t_r (minor) = 7.6 min, 99% ee.

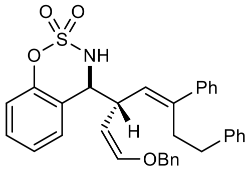

(4*S*)-4-[(1*Z*,3*R*,4*E*)-1-(Benzyloxy)-5,7-diphenylhepta-1,4-dien-3-yl]-3,4-dihydro-1,2λ^6,3-benzoxathiazine-2,2-dione (3n). A modification of General Procedure A was followed using imine **1a** (55.0 mg, 0.300 mmol), 1,3-enyne **2h** (99.5 mg, 0.360 mmol), phenylboronic acid (110 mg, 0.900 mmol), and *t*-amyl alcohol (0.10 mL, 0.90 mmol). The mixture was purified twice by column chromatography (5% EtOAc/petroleum ether; second column with 60:39:1 pentane/$CHCl_3$/EtOAc) to give *allylation product* **3n** (107 mg, 66%) as an orange oil (*ca.* 95% purity; small quantities of solvents, unidentified impurities, and silicone grease remained). R_f = 0.14 (10% EtOAc/petroleum ether); $[\alpha]_D^{25.4}$ –24.8 (*c* 0.30, $CHCl_3$); IR 3271 (N-H), 3027, 1366, 1191, 1169, 751, 696 cm^{-1}; ^1H NMR (400 MHz, $CDCl_3$) δ 7.45-7.30 (8H, m, Ar**H**), 7.29-7.17 (6H, m, Ar**H**), 7.17-7.05 (4H, m, Ar**H**), 7.00 (1H, dd, *J* = 8.2, 1.3 Hz, Ar**H**), 6.09 (1H, d, *J* = 6.2 Hz, OC**H**=CH), 5.89 (1H, d, *J* = 9.3 Hz, C=C**H**), 4.98 (1H, d, *J* = 8.7 Hz, N**H**), 4.77 (1H, d, *J* = 12.2 Hz, OC**H_A**H_B), 4.70 (1H, d, *J* = 12.2 Hz, OCH_A**H_B**), 4.53 (1H, dd, *J* = 8.7, 4.6 Hz, C**H**NH), 4.23 (1H, dd, *J* = 9.3, 6.2 Hz, OCH=C**H**), 4.12 (1H, td, *J* = 9.3, 4.6 Hz, C=CC**H**), 2.83 (2H, t, *J* = 7.6 Hz, PhC**H_2**), 2.73-2.63 (1H, m, PhCH_2C**H_A**H_B), 2.56-2.45 (1H, m, PhCH_2CH_A**H_B**); ^{13}C NMR (101 MHz, $CDCl_3$) δ 151.7 (C), 146.8 (CH), 141.8 (C), 141.6 (C), 141.5 (C), 136.4 (C), 129.2 (CH), 128.6 (2 × CH), 128.5 (4 × CH), 128.4 (2 × CH), 128.3 (CH), 127.6 (2 × CH), 127.4 (CH), 127.2 (CH), 126.7 (2 × CH), 126.5 (CH), 126.1 (CH), 124.7 (CH), 121.6 (C), 118.7 (CH), 102.5 (CH), 74.4 (CH_2), 60.9 (CH), 37.9 (CH), 34.3 (CH_2), 32.2 (CH_2); HRMS (ESI) Exact mass calcd for $[C_{33}H_{31}NNaO_4S]^+$ $[M+Na]^+$: 560.1866, found: 560.1870; Enantiomeric excess was determined by HPLC using a Chiralcel OD-H column (90:10 *iso*-hexane:*i*-PrOH, 1.0 mL/in, 254 nm): t_r (major) = 17.7 min, t_r (minor) = 28.9 min, 69% ee.

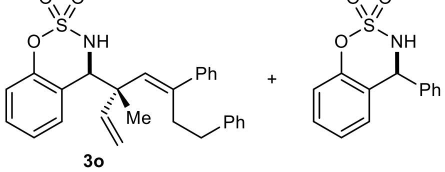

3o
(8.2:1 inseparable mixture)

(4*S*)-4-[(3*S*,*E*)-3-Methyl-5,7-diphenylhepta-1,4-dien-3-yl]-3,4-dihydro-1,2λ^6,3-benzoxathiazine-2,2-dione (3o). A modification of General Procedure A was followed using imine **1a** (55.0 mg, 0.300 mmol), 1,3-enyne **2i** (66.3 mg, 0.360 mmol), phenylboronic acid (110 mg, 0.900 mmol), and *t*-amyl alcohol (0.10 mL, 0.90 mmol). The mixture was purified by column chromatography (80:19:1 petroleum ether/CH_2Cl_2/EtOAc, to give an 8.2:1 inseparable mixture of the product of addition of phenylboronic acid to the imine, and *allylation*

product **3o**, respectively (67.0 mg, 43%, adjusted yield of **3o**) as a brown oil (*ca.* 95% purity; small quantities of silicone grease remained). R_f = 0.23 (10% EtOAc/petroleum ether); $[\alpha]_D^{25.3}$ +13.2 (*c* 0.22, $CHCl_3$); IR 3289 (N-H), 2927, 1364, 1169, 730, 697 cm^{-1}; ^{1}H NMR (400 MHz, $CDCl_3$) δ 7.47-7.31 (8H, m, Ar**H**), 7.30-7.24 (1H, m, Ar**H**) 7.21-7.14 (2H, m, Ar**H**), 7.11-7.05 (3H, m, Ar**H**), 6.06 (1H, dd, *J* = 17.4, 10.7 Hz, **H**C=CH_2), 5.66 (1H, s, C=C**H**), 5.31 (1H, d, *J* = 10.7 Hz, HC=C**H**$_A$H$_B$), 5.20 (1H, d, *J* = 17.4 Hz, HC=CH$_A$**H**$_B$), 4.82-4.75 (2H, m, N**H** and C**H**NH), 2.91 (2H, m, PhC**H**$_2$), 2.56 (2H, t, *J* = 8.0 Hz, PhCH$_2$C**H**$_2$), 1.34 (3H, s, C**H**$_3$); ^{13}C NMR (101 MHz, $CDCl_3$) δ 151.9 (C), 144.8 (C), 143.0 (C), 141.3 (C), 140.9 (CH), 131.4 (CH), 129.7 (CH), 128.7 (CH), 128.6 (2 × CH), 128.4 (2 × CH), 128.3 (2 × CH), 127.5 (CH), 126.7 (2 × CH), 126.1 (CH), 125.0 (CH), 121.4 (C), 119.5 (CH), 116.4 (CH_2), 65.0 (CH), 48.2 (C), 34.0 (CH_2), 33.0 (CH_2), 21.6 (CH_3); HRMS (ESI) Exact mass calcd for $C_{27}H_{27}NNaO_3S$ $[M+Na]^+$: 468.1604, found: 468.1603; Enantiomeric excess was determined by HPLC using a Chiralcel OD-H column (90:10 *iso*-hexane:*i*-PrOH, 1.0 mL/min, 254 nm): t_r (major) = 8.5 min, t_r (minor) = 12.7 min, 90% ee.

Characteristic signals of the product of addition of phenylboronic acid to the imine[13] were observed at: ^{1}H NMR (400 MHz, $CDCl_3$) δ 6.84 (1H, d, *J* = 8.1 Hz, Ar**H**), 5.92 (1H, d, *J* = 8.4 Hz, C**H**NH).

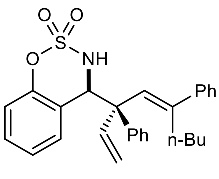

(4*S*)-4-[(3*S*,*E*)-3,5-Diphenylnona-1,4-dien-3-yl]-3,4-dihydro-1,2λ^6,3-benzoxathiazine-2,2-dione (3p). A modification of General Procedure A was followed using imine **1a** (55.0 mg, 0.300 mmol), 1,3-enyne **2j** (71.4 mg, 0.360 mmol), phenylboronic acid (110 mg, 0.900 mmol) and *t*-amyl alcohol (0.10 mL, 0.90 mmol). The mixture was purified by column chromatography (10% EtOAc/petroleum ether, followed by 75:20:5 petroleum ether/CH_2Cl_2/Et_2O), to give *allylation product* **3p** (54.0 mg, 39%) as an off-white solid (*ca.*95% purity; small quantities of silicone grease remained). R_f = 0.23 (10% EtOAc/petroleum ether); m.p. 127-129 °C (Et_2O); $[\alpha]_D^{25.6}$ +6.2 (*c* 0.38, $CHCl_3$); IR 3268 (N-H), 2954, 1417, 1166, 715, 696 cm^{-1}; ^{1}H NMR (400 MHz, $CDCl_3$) δ 7.40-7.24 (11H, m, Ar**H**), 7.04 (1H, dd, *J* = 8.3 and 1.3 Hz, Ar**H**), 6.92 (1H, td, *J* = 7.7 and 1.3 Hz, Ar**H**), 6.60-6.49 (2H, m, CH_2=C**H** and Ar**H**), 5.99 (1H, s, C=C**H**), 5.53 (1H, dd, *J* = 10.6, 0.9 Hz, C=C**H**$_A$H$_B$), 5.38 (1H, dd, *J* = 17.3, 0.9 Hz, C=CH$_A$**H**$_B$), 5.11 (1H, d, *J* = 4.8 Hz, C**H**NH), 4.92 (1H, d, *J* = 4.8 Hz, N**H**), 2.23-2.06 (2H, m, C**H**$_2$), 1.05-0.80 (4H, m, C**H**$_2$C**H**$_2$CH$_3$), 0.62 (3H, t, *J* = 6.8 Hz, C**H**$_3$); ^{13}C NMR (101 MHz, $CDCl_3$) δ 152.0 (C), 148.6 (C), 142.6 (C), 140.4 (C), 138.9 (CH), 129.7 (CH), 129.4 (CH), 128.7 (4 × CH), 128.4 (2 × CH), 127.8 (CH), 127.4 (CH), 126.6 (2 × CH), 126.4 (CH), 124.7 (CH), 120.7 (C), 119.3

13. (a) C. Jiang, Y. Lu, T. Hayashi, *Angew. Chem., Int. Ed.* **2014**, *53*, 9936-9939. (b) M. Quan, L. Tang, J. Shen, G. Yang, W. Zhang, *Chem. Commun.* **2017**, *53*, 609-612.

(CH), 118.8 (CH_2), 64.8 (CH), 57.4 (C), 32.0 (CH_2), 28.6 (CH_2), 22.8 (CH_2), 13.6 (CH_3); HRMS (ESI) Exact mass calcd for $[C_{28}H_{29}NNaO_3S]^+$ $[M+Na]^+$: 482.1760, found: 482.1753; Enantiomeric excess was determined by HPLC using a Chiralcel OD-H column (90:10 *iso*-hexane:*i*-PrOH, 1.0 mL/min, 210 nm): t_r (major) = 6.3 min, t_r (minor) = 9.8 min, 2% ee.

Crystals suitable for X-ray analysis were prepared by slow diffusion of petroleum ether into a solution of **3p** in EtOAc. The resulting crystals are of the racemic compound.

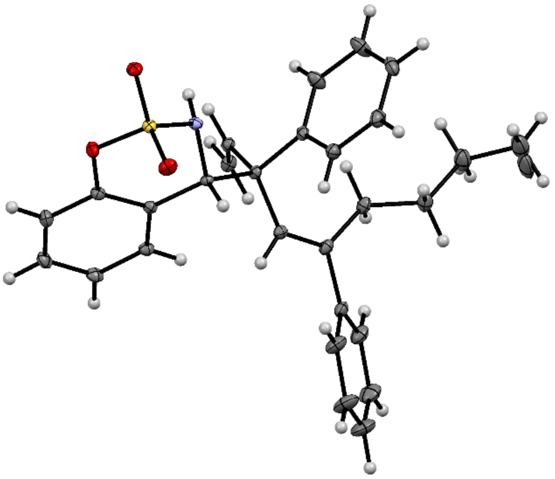

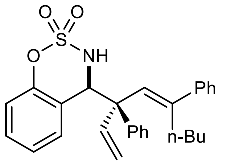

3p
CCDC 1552181

Note: In the butyl chain, the two carbon atoms at the terminal end exhibit disorder. This disorder is not shown above, for clarity.

(4*S*)-4-[(3*R*,*E*)-5-Cyclohexyl-2-methyl-5-phenylpenta-1,4-dien-3-yl]-3,4-dihydro-1,2λ^6,3-benzoxathiazine-2,2-dione [(*E*)-3q] and (4*S*)-4-[(3*R*,*Z*)-5-cyclohexyl-2-methyl-5-phenylpenta-1,4-dien-3-yl]-3,4-dihydro-1,2λ^6,3-benzoxathiazine-2,2-dione [(*Z*)-3q]

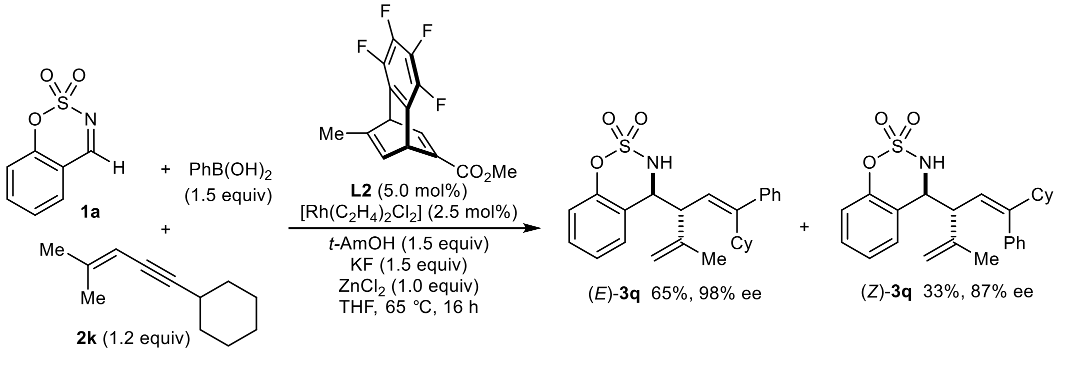

General Procedure A was followed using imine **1a** (55.0 mg, 0.300 mmol), 1,3-enyne **2k** (58.0 mg, 0.360 mmol), and phenylboronic acid (54.9 mg, 0.450 mmol). The mixture was purified by column chromatography (10% EtOAc/*iso*-hexane) to give *allylation product* (*Z*)-**3q** (43.0 mg, 33%) as an off-white solid (*ca.* 97% purity; small quantities of solvents, unidentified impurities, and silicone grease remained). Additional column chromatography (70:29:1 *iso*-hexane/CH_2Cl_2/EtOAc) gave *allylation product* (*E*)-**3q** (85.0 mg, 65%) as a white solid (*ca.* 95% purity; small quantities of

solvents, unidentified impurities, and silicone grease remained). The stereochemistry of the alkenes of (*E*)-**3q** and (*Z*)-**3q** were assigned on the basis of NOESY NMR spectra (see pages 60 and 62).

Data for (*E*)-**3q**: R_f = 0.35 (10% EtOAc/*iso*-hexane); m.p. 128-129 °C (Et_2O); $[\alpha]_D^{25.0}$ –50.0 (c 0.56, $CHCl_3$); IR 3280 (N-H), 2925, 1485, 1415 1192, 1168, 755, 729 cm^{-1}; ^{1}H NMR (400 MHz, $CDCl_3$) 7.37-7.32 (1H, m, Ar**H**), 7.31-7.19 (5H, m, Ar**H**), 7.06 (1H, dd, *J* = 8.2, 1.3 Hz, Ar**H**), 6.83-6.79 (2H, m, Ar**H**), 5.15 (1H, dd, *J* = 6.1, 4.7 Hz, C**H**NH), 5.12-5.10 (1H, m, C=C$\mathbf{H_A}$$H_B$), 5.10-5.05 (2H, m, C=C$H_A$$\mathbf{H_B}$ and C=C**H**), 4.64 (1H, d, *J* = 6.1 Hz, N**H**), 3.91 (1H, dd, *J* = 9.9, 4.7 Hz, C=CHC**H**), 2.38 (1H, tt, *J* = 11.3, 2.9 Hz, C**H**CH_2), 1.99-1.96 (3H, m, C$\mathbf{H_3}$), 1.78-1.53 (4H, m, (C$\mathbf{H_2}$)$_5$), 1.32-1.11 (4H, m, (C$\mathbf{H_2}$)$_5$), 1.05-0.85 (2H, m, (C$\mathbf{H_2}$)$_5$); ^{13}C NMR (101 MHz, $CDCl_3$) δ 151.5 (C), 151.4 (C), 143.3 (C), 142.3 (C), 129.3 (CH), 128.5 (2 × CH), 127.5 (2 × CH), 126.8 (CH), 126.6 (CH), 125.3 (CH), 122.5 (CH), 122.1 (C), 119.1 (CH), 115.6 (CH_2), 57.6 (CH), 47.9 (CH), 41.0 (CH), 31.5 (CH_2), 31.0 (CH_2), 26.6 (CH_2), 26.4 (CH_2), 25.8 (CH_2), 21.7 (CH_3); HRMS (ESI) Exact mass calcd for $[C_{25}H_{29}NNaO_3S]^+$ $[M+Na]^+$ 446.1760, found: 466.1745; Enantiomeric excess was determined by HPLC using a Chiralcel OD-H column (90:10 *iso*-hexane:*i*-PrOH, 1.0 mL/min, 254 nm): t_r (major) = 5.4 min, t_r (minor) = 8.4 min, 98% ee.

Data for (*Z*)-**3q**: R_f = 0.46 (10% EtOAc/*iso*-hexane); m.p. 151-153 °C (Et_2O); $[\alpha]_D^{25.0}$ –21.3 (c 0.94, $CHCl_3$); IR 3260 (N-H), 2923, 1486 1433, 1196, 1171, 704 cm^{-1}; ^{1}H NMR (400 MHz, $CDCl_3$) δ 7.35-7.25 (4H, m, Ar**H**), 7.09 (1H, td, *J* = 7.6, 1.3 Hz, Ar**H**), 7.00 (1H, dd, *J* = 8.2, 1.2 Hz, Ar**H**), 6.90-6.85 (2H, m, Ar**H**), 6.70 (1H, dt, *J* = 7.8, 1.1 Hz, Ar**H**), 5.34 (1H, dd, *J* = 10.4, 1.1 Hz, C=C**H**), 5.14-5.11 (1H, m, C=C$\mathbf{H_A}$$H_B$), 5.06-5.03 (1H, m, C=C$H_A$$\mathbf{H_B}$), 4.94 (1H, dd, *J* = 5.1, 4.3 Hz, C**H**NH), 4.78 (1H, d, *J* = 5.1 Hz, N**H**), 3.26 (1H, dd, *J* = 10.3, 4.3 Hz, C=CHC**H**), 1.99 (1H, tt, *J* = 11.7, 4.6 Hz, C**H**CH_2), 1.83-1.80 (3H, m, C$\mathbf{H_3}$), 1.74-1.55 (3H, m, (C$\mathbf{H_2}$)$_5$), 1.54-1.43 (2H, m, (C$\mathbf{H_2}$)$_5$), 1.23-0.89 (5H, m, (C$\mathbf{H_2}$)$_5$); ^{13}C NMR (101 MHz, $CDCl_3$) δ 151.9 (C), 151.2 (C), 144.3 (C), 140.6 (C), 128.9 (CH), 127.92 (2 × CH), 127.86 (2 × CH), 127.2 (CH), 126.9 (CH), 125.2 (CH), 121.8 (C), 118.6 (CH), 118.5 (CH), 115.8 (CH_2), 57.1 (CH), 48.9 (CH), 46.2 (CH), 32.9 (CH_2), 31.8 (CH_2), 26.6 (CH_2), 26.4 (CH_2), 26.0 (CH_2), 21.5 (CH_3); HRMS (ESI) Exact mass calcd for $[C_{25}H_{29}NNaO_3S]^+$ $[M+Na]^+$ 446.1760, found: 466.1749; Enantiomeric excess was determined by HPLC using a Chiralcel IC column (90:10 *iso*-hexane:*i*-PrOH, 0.8 mL/min, 254 nm): t_r (minor) = 8.7 min, t_r (major) = 9.6 min, 87% ee.

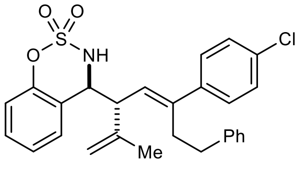

(4*S*)-4-[(3*R*,*E*)-5-(4-Chlorophenyl)-2-methyl-7-phenylhepta-1,4-dien-3-yl]-3,4-dihydro-1,2λ^6,3-benzoxathiazine-2,2-dione (3r). General Procedure A was followed using imine **1a** (55.1 mg, 0.301 mmol), 1,3-enyne **2a** (65.3 mg, 0.360 mmol), and 4-chlorophenylboronic acid (70.4 mg,

0.45 mmol). The mixture was purified twice by column chromatography (15% EtOAc/petroleum ether; second column with 60:38:2 pentane/$CHCl_3$/EtOAc) to give *allylation product* **3r** (78 mg, 91% purity as determined by use of an internal standard, 49% adjusted yield) as a pale yellow oil (unidentified impurities remained, which could not be separated). R_f = 0.34 (15% EtOAc/petroleum ether); $[\alpha]_D^{25.0}$ –49.8 (*c* 0.48, $CHCl_3$); IR 3268 (N-H), 2966, 1487, 1368, 1168, 756 cm^{-1}; ^1H NMR (400 MHz, $CDCl_3$) δ 7.34-7.27 (3H, m, Ar**H**), 7.23-7.15 (3H, m, Ar**H**), 7.16-7.10 (3H, m, Ar**H**), 7.09-7.01 (4H, m, Ar**H**), 5.48 (1H, d, *J* = 9.3 Hz, C=C**H**), 5.05 (1H, s, C=C**H**$_{\mathbf{A}}$H$_B$), 4.97 (1H, dd, *J* = 7.3, 6.5 Hz, C**H**NH), 4.94 (1H, s, C=CH$_A$**H**$_{\mathbf{B}}$), 3.97 (1H, d, *J* = 7.3 Hz, N**H**), 3.52 (1H, dd, *J* = 9.3, 6.5 Hz, C=CC**H**), 2.73 (2H, dd, *J* = 8.1, 6.5 Hz, PhC**H**$_{\mathbf{2}}$), 2.62 (1H, dt, *J* = 13.6, 6.5 Hz, PhCH$_2$C**H**$_{\mathbf{A}}$H$_B$), 2.38 (1H, dt, *J* = 13.6, 8.1 Hz, PhCH$_2$CH$_A$**H**$_{\mathbf{B}}$), 1.90 (3H, s, C**H**$_{\mathbf{3}}$); ^{13}C NMR (101 MHz, $CDCl_3$) δ 151.3 (C), 143.2 (C), 141.9 (C), 141.2 (C), 140.1 (C), 133.3 (C), 129.4 (CH), 128.6 (2 × CH), 128.5 (2 × CH), 128.4 (2 × CH), 127.9 (2 × CH), 126.7 (CH), 126.4 (CH), 125.6 (CH), 125.2 (CH), 122.0 (C), 118.7 (CH), 115.4 (CH_2), 56.8 (CH), 49.1 (CH), 33.6 (CH_2), 31.8 (CH_2), 21.4 (CH_3); HRMS (ESI) Exact mass calcd for $[C_{27}H_{26}{}^{35}ClNNaO_3S]^+$ $[M+Na]^+$: 502.1214, found: 502.1192; Enantiomeric excess was determined by HPLC using a Chiralcel OD-H column (90:10 *iso*-hexane:*i*-PrOH, 1.5 mL/min, 210 nm): t_r (major) = 4.9 min, t_r (minor) = 10.9 min, 98% ee.

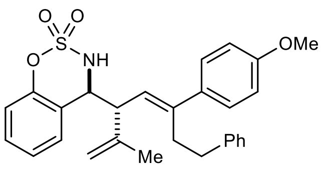

(4*S*)-4-[(3*R*,*E*)-5-(4-Methoxyphenyl)-2-methyl-7-phenylhepta-1,4-dien-3-yl]-3,4-dihydro-1,2λ^6,3-benzoxathiazine-2,2-dione (3s). General Procedure A was followed using imine **1a** (55.0 mg, 0.300 mmol), 1,3-enyne **2a** (65.3 mg, 0.360 mmol), and 4-methoxyphenylboronic acid (63.9 mg, 0.420 mmol). The mixture was purified by column chromatography (20% EtOAc/petroleum ether) to give *allylation product* **3s** (96.5 mg, 68%) as a pale yellow oil (*ca.* 95% purity; small quantities of unidentified impurities remained). R_f = 0.15 (20% EtOAc/*iso*-hexane); $[\alpha]_D^{25.5}$ –32.0 (*c* 0.25, $CHCl_3$); IR 3278 (N-H), 2933, 1510, 1364, 1166, 1032, 757 cm^{-1}; ^1H NMR (500 MHz, $CDCl_3$) δ 7.33-7.27 (1H, m, Ar**H**), 7.24-6.97 (10H, m, Ar**H**), 6.86 (2H, d, *J* = 8.8 Hz, Ar**H**), 5.42 (1H, d, *J* = 9.4 Hz, C=C**H**), 5.04 (1H, t, *J* = 1.5 Hz, C=C**H**$_{\mathbf{A}}$H$_B$), 4.98 (1H, dd, *J* = 7.3, 6.4 Hz, C**H**NH), 4.96 (1H, s, C=CH$_A$**H**$_{\mathbf{B}}$), 4.02 (1H, d, *J* = 7.3 Hz, N**H**), 3.82 (3H, s, OC**H**$_{\mathbf{3}}$), 3.52 (1H, dd, *J* = 9.4, 6.4 Hz, C=CC**H**), 2.72 (2H, dd, *J* = 8.1, 6.6 Hz, PhC**H**$_{\mathbf{2}}$), 2.62 (1H, dt, *J* = 13.4, 6.6 Hz, PhCH$_2$C**H**$_{\mathbf{A}}$H$_B$); 2.41 (1H, dt, *J* = 13.4, 8.1 Hz, PhCH$_2$CH$_A$**H**$_{\mathbf{B}}$), 1.90 (3H, s, CC**H**$_{\mathbf{3}}$); ^{13}C NMR (126 MHz, $CDCl_3$) δ 159.1 (C), 151.4 (C), 143.5 (C), 142.5 (C), 141.6 (C), 134.0 (C), 129.2 (CH), 128.5 (2 × CH), 128.4 (2 × CH), 127.7 (2 × CH), 126.8 (CH), 126.3 (CH), 125.2 (CH), 123.5 (CH), 122.2 (C), 118.7 (CH), 115.2 (CH_2), 113.8 (2 × CH), 56.9 (CH), 55.3 (CH_3), 49.2 (CH), 33.8 (CH_2), 31.9 (CH_2), 21.4 (CH_3); HRMS (ESI) Exact mass calcd for $[C_{28}H_{29}NNaO_4S]^+$

$[M+Na]^+$: 498.1710, found: 498.1705; Enantiomeric excess was determined by HPLC using a Chiralcel OD-H column (90:10 *iso*-hexane:*i*-PrOH, 1.5 mL/min, 210 nm); t_r (major) = 6.4 min, t_r (minor) = 11.4 min, 99% ee.

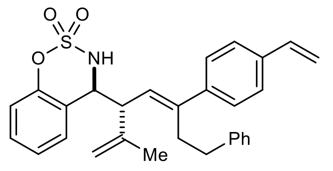

(4*S*)-4-[(3*R*,*E*)-5-(4-Ethenylphenyl)-2-methyl-7-phenylhepta-1,4-dien-3-yl]-3,4-dihydro-1,2λ^6,3-benzoxathiazine-2,2-dione (3t). General Procedure A was followed using imine **1a** (55.3 mg, 0.302 mmol), 1,3-enyne **2a** (65.3 mg, 0.360 mmol), and 4-vinylphenylboronic acid (66.2 mg, 0.450 mmol). The mixture was purified by column chromatography (15% EtOAc/*iso*-hexane) to give *allylation product* **3t** (96 mg, 68%) as a yellow oil (*ca.* 95% purity; small quantities of unidentified impurities and silicone grease remained). R_f = 0.14 (15% EtOAc/petroleum ether); $[\alpha]_D^{25.2}$ –44.6 (*c* 0.25, $CHCl_3$); IR 3269 (N-H), 2935, 1368, 1168, 752 cm^{-1}; ^{1}H NMR (400 MHz, $CDCl_3$) δ 7.40-7.35 (2H, m, Ar**H**), 7.34-7.29 (1H, m, Ar**H**), 7.24-7.11 (6H, m, Ar**H**), 7.10-7.01 (4H, m, Ar**H**), 6.73 (1H, dd, *J* = 17.6, 10.9 Hz, C**H**=CH_2), 5.77 (1H, dd, *J* = 17.6, 0.9 Hz, CH=C**H_A**H_B), 5.53 (1H, d, *J* = 9.3 Hz, C=C**H**), 5.27 (1H, dd, *J* = 10.9, 0.9 Hz, CH=C**H_A**H_B), 5.05 (1H, br s, C=CH_A**H_B**), 4.99 (1H, dd, J = 7.3, 6.4 Hz, C**H**NH), 4.96 (1H, br s, C=CH_A**H_B**), 4.05 (1H, br s, N**H**), 3.55 (1H, dd, *J* = 9.3, 6.4 Hz, C=CC**H**), 2.80-2.72 (2H, m, PhC**H_2**), 2.64 (1H, dt, *J* = 13.5, 6.5 Hz, $PhCH_2C$**H_A**H_B), 2.42 (1H, dt, J = 13.5, 8.1 Hz, $PhCH_2CH_A$**H_B**), 1.91 (3H, s, C**H_3**); ^{13}C NMR (101 MHz, $CDCl_3$) δ 151.4 (C), 143.4 (C), 142.7 (C), 141.5 (C), 141.0 (C), 136.9 (CH), 136.3 (C), 129.3 (CH), 128.5 (2 × CH), 128.4 (2 × CH), 126.8 (2 × CH), 126.7 (CH), 126.3 (3 × CH), 125.2 (CH), 124.8 (CH), 122.1 (C), 118.7 (CH), 115.3 (CH_2), 113.9 (CH_2), 56.9 (CH), 49.2 (CH), 33.7 (CH_2), 31.7 (CH_2), 21.4 (CH_3); HRMS (ESI) Exact mass calcd for $[C_{29}H_{29}NNaO_3S]^+$ $[M+Na]^+$: 494.1760, found: 494.1750; Enantiomeric excess was determined by HPLC using a Chiralcel OD-H column (90:10 *iso*-hexane:*i*-PrOH, 1.5 mL/min, 210 nm): t_r (major) = 4.8 min, t_r (minor) = 8.8 min, 99% ee.

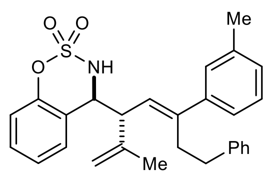

(4*S*)-4-[(3*R*,*E*)-2-Methyl-5-(3-methylphenyl)-7-phenylhepta-1,4-dien-3-yl]-3,4-dihydro-1,2λ^6,3-benzoxathiazine-2,2-dione (3u). General Procedure A was followed using imine **1a** (54.9 mg, 0.300 mmol), 1,3-enyne **2a** (81.6 mg, 0.450 mmol), and 3-methylphenylboronic acid (61.2 mg, 0.450 mmol). The mixture was purified by column chromatography (10% EtOAc/petroleum ether) to give *allylation product* **3u** (92.7 mg, 67%) as a pale yellow oil (*ca.* 95% purity; small quantities of unidentified impurities remained). R_f = 0.27 (20% EtOAc/petroleum ether); $[\alpha]_D^{25.5}$ –30.3 (*c* 0.40, $CHCl_3$); IR 3269 (N-H), 2922, 1370, 1168, 753 cm^{-1}; ^{1}H NMR (400 MHz, $CDCl_3$) δ 7.34-7.28 (1H, m, Ar**H**), 7.24-7.00 (10H, m, Ar**H**), 7.00-6.95 (2H, m, Ar**H**), 5.46 (1H, d, *J* = 9.4 Hz, C=C**H**), 5.05

(1H, s, C=C**H$_A$**H$_B$), 4.99 (1H, dd, *J* = 7.3, 6.4 Hz, C**H**NH), 4.97 (1H, s, C=CH$_A$**H$_B$**), 4.06 (1H, d, *J* = 7.3 Hz, N**H**), 3.55 (1H, dd, *J* = 9.4, 6.4 Hz, C=CC**H**), 2.80-2.67 (2H, m, PhC**H$_2$**), 2.62 (1H, dt, *J* = 13.6, 6.7 Hz, PhCH$_2$C**H$_A$**H$_B$), 2.41 (1H, dt, *J* = 13.6, 8.1 Hz, PhCH$_2$CH$_A$**H$_B$**), 2.35 (3H, s, ArC**H$_3$**), 1.90 (3H, s, C=CC**H$_3$**); ^{13}C NMR (101 MHz, CDCl$_3$) δ 151.4 (C), 143.42 (C), 143.35 (C), 141.7 (C), 141.6 (C), 138.0 (C), 129.3 (CH), 128.5 (2 × CH), 128.4 (2 × CH), 128.33 (CH), 128.25 (CH), 127.3 (CH), 126.8 (CH), 126.3 (CH), 125.2 (CH), 124.5 (CH), 123.8 (CH), 122.1 (C), 118.7 (CH), 115.3 (CH$_2$), 57.0 (CH), 49.1 (CH), 33.8 (CH$_2$), 31.9 (CH$_2$), 21.5 (CH$_3$), 21.4 (CH$_3$); HRMS (ESI) Exact mass calcd for $[C_{28}H_{30}NO_3S]^+$ $[M+H]^+$: 460.1941, found: 460.1938; Enantiomeric excess was determined by HPLC using a Chiralcel OD-H column (90:10 *iso*-hexane:*i*-PrOH, 1.5 mL/min, 210 nm); t$_r$ (major) = 4.2 min, t$_r$ (minor) = 8.0 min, 99% ee.

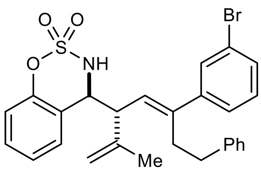

(4*S*)-4-[(3*R*,*E*)-5-(3-Bromophenyl)-2-methyl-7-phenylhepta-1,4-dien-3-yl]-3,4-dihydro-1,2λ^6,3-benzoxathiazine-2,2-dione (**3v**). General Procedure A was followed using imine **1a** (55.2 mg, 0.301 mmol), 1,3-enyne **2a** (65.3 mg, 0.360 mmol), and 3-bromophenylboronic acid (90.4 mg, 0.450 mmol). The mixture was purified by column chromatography (20% EtOAc/petroleum ether) to give *allylation product* **3v** (97.3 mg, 62%) as a pale yellow oil (*ca.* 93% purity; small quantities of solvents, unidentified impurities, and silicone grease remained). R$_f$ = 0.22 (20% EtOAc/*iso*-hexane); $[\alpha]_D^{23.8}$ – 143.6 (*c* 0.36, CHCl$_3$); IR 3273 (N-H), 2922, 1369, 1168, 756 cm^{-1}; ^{1}H NMR (500 MHz, CDCl$_3$) δ 7.41 (1H, ddd, *J* = 7.9, 2.0, 1.0 Hz, Ar**H**), 7.35-7.28 (2H, m, Ar**H**), 7.25-7.16 (4H, m, Ar**H**), 7.13 (1H, td, *J* = 7.6, 1.3 Hz, Ar**H**), 7.09 (1H, dt, *J* = 7.8, 1.4 Hz, Ar**H**), 7.07-7.01 (4H, m, Ar**H**), 5.49 (1H, d, *J* = 9.4 Hz, C=C**H**), 5.06 (1H, s, C=C**H$_A$**H$_B$), 4.97 (1H, dd, *J* = 7.3, 6.5 Hz, C**H**NH), 4.95 (1H, s, C=CH$_A$**H$_B$**), 4.05 (1H, d, *J* = 7.3 Hz, N**H**), 3.54 (1H, dd, *J* = 9.4, 6.5 Hz, C=CC**H**), 2.72 (2H, dd, *J* = 8.2, 6.6 Hz, PhC**H$_2$**), 2.62 (1H, dt, *J* = 13.6, 6.6 Hz, PhCH$_2$C**H$_A$**H$_B$), 2.41 (1H, dt, *J* = 13.6, 8.2 Hz, PhCH$_2$CH$_A$**H$_B$**), 1.90 (3H, s, C**H$_3$**); ^{13}C NMR (101 MHz, CDCl$_3$) δ 151.3 (C), 143.9 (C), 143.1 (C), 141.9 (C), 141.2 (C), 130.4 (CH), 130.0 (CH), 129.5 (CH), 129.4 (CH), 128.5 (2 × CH), 128.4 (2 × CH), 126.7 (CH), 126.4 (CH), 126.2 (CH), 125.5 (CH), 125.2 (CH), 122.6 (C), 121.9 (C), 118.8 (CH), 115.5 (CH$_2$), 56.9 (CH), 49.1 (CH), 33.6 (CH$_2$), 31.8 (CH$_2$), 21.4 (CH$_3$); HRMS (ESI) Exact mass calcd for $[C_{27}H_{26}{}^{79}BrNNaO_3S]^+$ $[M+Na]^+$: 546.0709, found: 546.0700; Enantiomeric excess was determined by HPLC using a Chiralcel OD-H column (90:10 *iso*-hexane:*i*-PrOH, 1.5 mL/min, 210 nm); t$_r$ (major) = 4.9 min, t$_r$ (minor) = 10.7 min, 99% ee.

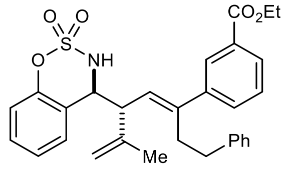

3-[(*E*,5*R*)-5-[(4*S*)-2,2-Dioxo-3,4-dihydro-1,2λ^6,3-benzoxathiazin-4-yl]-6-methyl-1-phenylhepta-3,6-dien-3-yl]benzoate (3w). General Procedure A was followed using imine **1a** (54.8 mg, 0.299 mmol), 1,3-enyne **2a** (65.3 mg, 0.360 mmol), and 3-(ethoxycarbonyl)phenylboronic acid (87.3 mg, 0.450 mmol). The mixture was purified twice by column chromatography (first column with 20% EtOAc/petroleum ether; second column with 60:38:2 pentane/$CHCl_3$/EtOAc) to give *allylation product* **3w** (84 mg, 54%) as a pale yellow oil. R_f = 0.19 (20% EtOAc/petroleum ether); $[\alpha]_D^{25.3}$ –32.6 (*c* 0.37, $CHCl_3$); IR 3263 (N-H), 2929, 1699, 1367, 1168, 754 cm^{-1}; ^{1}H NMR (500 MHz, $CDCl_3$) δ 7.95 (1H, dt, *J* = 7.7, 1.5 Hz, Ar**H**), 7.87 (1H, t, *J* = 1.8 Hz, Ar**H**), 7.38 (1H, t, *J* = 7.7 Hz, Ar**H**), 7.35-7.29 (2H, m, Ar**H**), 7.24-7.16 (3H, m, Ar**H**), 7.14 (1H, td, *J* = 7.6, 1.3 Hz, Ar**H**), 7.09-7.01 (4H, m, Ar**H**), 5.54 (1H, d, *J* = 9.3 Hz, C=C**H**), 5.06 (1H, t, *J* = 1.6 Hz, C=C**H**$_A$H$_B$), 4.99 (1H, dd, *J* = 7.3, 6.4 Hz, C**H**NH), 4.97-4.95 (1H, m, C=CH$_A$**H**$_B$), 4.43-4.34 (2H, m, OC**H**$_2$), 4.17 (1H, d, *J* = 7.3 Hz, N**H**), 3.59 (1H, dd, *J* = 9.3, 6.4 Hz, C=CC**H**), 2.81 (1H, ddd, *J* = 14.3, 8.2, 6.1 Hz, PhC**H**$_A$CH$_B$), 2.77 (1H, dt, *J* = 14.3, 7.9, 7.4 Hz, PhCH$_A$C**H**$_B$), 2.62 (1H, ddd, *J* = 13.7, 7.4, 6.1 Hz, PhCH$_2$C**H**$_A$H$_B$), 2.39 (1H, ddd, *J* = 13.7, 8.2, 7.9 Hz, PhCH$_2$CH$_A$**H**$_B$), 1.91 (3H, s, CC**H**$_3$), 1.41 (3H, t, *J* = 7.1 Hz, CH$_2$C**H**$_3$); ^{13}C NMR (126 MHz, $CDCl_3$) δ 166.5 (C), 151.4 (C), 143.2 (C), 142.4 (C), 142.0 (C), 141.3 (C), 131.2 (CH), 130.6 (C), 129.3 (CH), 128.6 (CH), 128.54 (CH), 128.50 (2 × CH), 128.3 (2 × CH), 127.5 (CH), 126.7 (CH), 126.3 (CH), 125.9 (CH), 125.2 (CH), 122.0 (C), 118.7 (CH), 115.5 (CH_2), 61.1 (CH_2), 56.9 (CH), 49.1 (CH), 33.7 (CH_2), 31.9 (CH_2), 21.4 (CH_3), 14.3 (CH_3); HRMS (ESI) Exact mass calcd for $[C_{30}H_{31}NNaO_5S]^+$ $[M+Na]^+$: 540.1815, found: 540.1801; Enantiomeric excess was determined by HPLC using a Chiralcel OD-H column (90:10 *iso*-hexane:*i*-PrOH, 1.5 mL/min, 210 nm): t_r (major) = 5.8 min, t_r (minor) = 13.2 min, 96% ee.

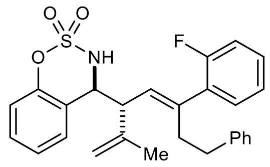

(4*S*)-4-[(3*R*,*E*)-5-(2-Fluorophenyl)-2-methyl-7-phenylhepta-1,4-dien-3-yl]-3,4-dihydro-1,2λ^6,3-benzoxathiazine-2,2-dione (3x). General Procedure A was followed using imine **1a** (55.0 mg, 0.300 mmol), 1,3-enyne **2a** (66.3 mg, 0.360 mmol), and 2-fluorophenylboronic acid (63.0 mg, 0.450 mmol). The mixture was purified by column chromatography (10% EtOAc/*iso*-hexane) to give *allylation product* **3x** (95.0 mg, 68%) as a yellow oil (*ca.* 95% purity; small quantities of solvents and silicone grease remained). R_f = 0.27 (10% EtOAc/*iso*-hexane); $[\alpha]_D^{25.0}$ –37.0 (c 0.54, $CHCl_3$); IR 3273 (N-H), 2923, 1485, 1450 1192, 1168, 753, 699 cm^{-1}; ^{1}H NMR (400 MHz, $CDCl_3$) δ 7.36-7.30 (1H, m, Ar**H**), 7.30-7.15 (5H, m, Ar**H**), 7.14-7.06 (4H, m, Ar**H**), 7.05-6.99 (2H, m, Ar**H**), 6.95 (1H, td, *J* = 7.6, 1.8 Hz, Ar**H**), 5.43 (1H, d, *J* = 9.5 Hz, C=C**H**), 5.12-5.10 (1H, m, C=C**H**$_A$H$_B$), 5.07-5.02 (2H, m, C=CH$_A$**H**$_B$ and C**H**NH), 4.31 (1H, d, *J* = 6.6 Hz, N**H**), 3.69 (1H, dd, *J* = 9.5, 5.9 Hz, C=CC**H**), 2.83-2.67 (2H,

m, PhC**H**$_2$), 2.59 (1H, ddd, *J* = 13.8, 8.1, 5.8 Hz, PhCH$_2$C**H**$_A$H$_B$), 2.40 (1H, dt, *J* = 13.8, 8.1 Hz, PhCH$_2$CH$_A$**H**$_B$), 1.95 (3H, s, C**H**$_3$); ^{13}C NMR (101 MHz, CDCl$_3$) δ 159.7 (d, $^1J_{C-F}$ = 246.6 Hz, C), 151.4 (C), 142.8 (C), 141.4 (C), 139.5 (C), 130.4 (d, $^3J_{C-F}$= 4.3 Hz, CH), 129.8 (d, $^2J_{C-F}$ = 14.6 Hz, C), 129.2 (CH), 128.9 (d, $^3J_{C-F}$= 8.1 Hz, CH), 128.4 (2 × CH), 128.3 (2 × CH), 127.2 (CH), 126.7 (CH), 126.2 (CH), 125.2 (CH), 124.1 (d, $^4J_{C-F}$ = 3.6 Hz, CH), 121.8 (C), 118.7 (CH), 115.6 (d, $^2J_{C-F}$ = 22.7 Hz, CH), 115.5 (CH$_2$), 56.9 (CH), 48.6 (CH), 33.7 (CH$_2$), 32.7 (d, $^4J_{C-F}$= 3.3 Hz, CH$_2$), 21.5 (CH$_3$); ^{19}F NMR (376 MHz, CDCl$_3$) δ –115.3 (m); HRMS (ESI) Exact mass calcd for $[C_{27}H_{26}FNNaO_3S]^+$ $[M+Na]^+$ 486.1510, found: 486.1503; Enantiomeric excess was determined by HPLC using a Chiralcel OD-H column (90:10 *iso*-hexane:*i*-PrOH, 1.0 mL/min, 210 nm): t_r (major) = 6.3 min, t_r (minor) = 11.8 min, 98% ee.

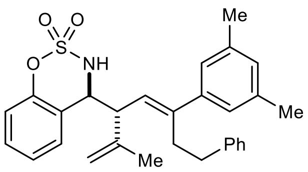

(4*S*)-4-[(3*R*,*E*)-5-(3,5-Dimethylphenyl)-2-methyl-7-phenylhepta-1,4-dien-3-yl]-3,4-dihydro-1,2λ^6,3-benzoxathiazine-2,2-dione (3y). General Procedure A was followed using imine **1a** (55.3 mg, 0.302 mmol), 1,3-enyne **2a** (65.3 mg, 0.360 mmol), and 3,5-dimethylphenylboronic acid (67.5 mg, 0.450 mmol). The mixture was purified by column chromatography (15% EtOAc/petroleum ether) to give *allylation product* **3y** (91.2 mg, 64%) as a pale brown solid (*ca.* 97% purity, small quantities of unidentified impurities remain). R_f = 0.41 (15% EtOAc/petroleum ether); m.p. 146-147 °C (toluene/petroleum ether); $[\alpha]_D^{25.2}$ –41.7 (*c* 0.38, CHCl$_3$); IR 3274 (N-H), 2947, 1370, 1166, 757 cm^{-1}; ^{1}H NMR (400 MHz, CDCl$_3$) δ 7.33-7.27 (1H, m, Ar**H**), 7.25-7.00 (8H, m, Ar**H**), 6.92 (1H, br s, Ar**H**), 6.77 (2H, br s, Ar**H**), 5.44 (1H, d, *J* = 9.4 Hz, C=C**H**), 5.04 (1H, s, C=C**H**$_A$H$_B$), 4.99 (1H, dd, *J* = 7.3, 6.3 Hz, C**H**NH), 4.96 (1H, q, *J* = 1.0 Hz, C=CH$_A$**H**$_B$), 4.07 (1H, d, *J* = 7.3 Hz, N**H**), 3.55 (1H, dd, *J* = 9.4, 6.3 Hz, C=CC**H**), 2.77-2.67 (2H, m, PhC**H**$_2$), 2.62 (1H, dt, *J* = 13.6, 6.8 Hz, PhCH$_2$C**H**$_A$H$_B$), 2.42 (1H, dt, *J* = 13.6, 8.1 Hz, PhCH$_2$CH$_A$**H**$_B$), 2.31 (6H, s, 2 × ArC**H**$_3$), 1.90 (3H, s, C=CC**H**$_3$); ^{13}C NMR (101 MHz, CDCl$_3$) δ 151.4 (C), 143.5 (C), 143.5 (C), 141.72 (C), 141.66 (C), 137.9 (2 × C), 129.3 (CH), 129.2 (CH), 128.5 (2 × CH), 128.4 (2 × CH), 126.8 (CH), 126.3 (CH), 125.2 (CH), 124.5 (2 × CH), 124.2 (CH), 122.1 (C), 118.7 (CH), 115.3 (CH$_2$), 57.0 (CH), 49.1 (CH), 33.8 (CH$_2$), 32.0 (CH$_2$), 21.42 (CH$_3$), 21.39 (2 × CH$_3$); HRMS (ESI) Exact mass calcd for $[C_{29}H_{35}N_2O_3S]^+$ $[M+NH_4]^+$: 491.2363, found: 491.2358; Enantiomeric excess was determined by HPLC using a Chiralcel OD-H column (90:10 *iso*-hexane:*i*-PrOH, 1.5 mL/min, 210 nm); t_r (major) = 3.7 min, t_r (minor) = 6.4 min, 99% ee.

Crystals suitable for X-ray analysis were prepared by slow evaporation of a solution of allylation product **3y** in toluene/petroleum ether.

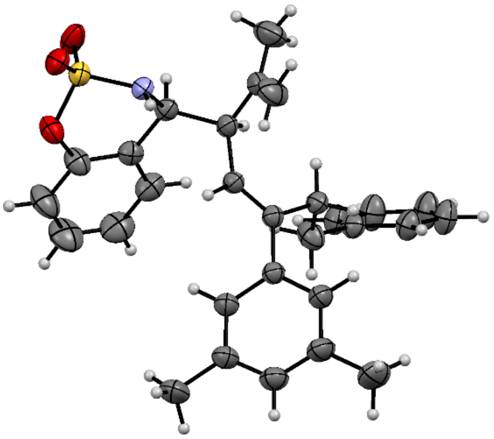

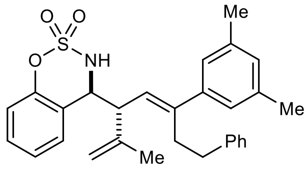

3y
CCDC 1552182
Flack parameter = –0.003(14)

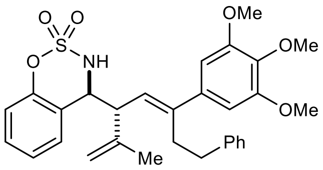

(4*S*)-4-[(3*R*,*E*)-2-Methyl-7-phenyl-5-(3,4,5-trimethoxyphenyl)hepta-1,4-dien-3-yl]-3,4-dihydro-1,2λ^{6},3-benzoxathiazine-2,2-dione (3z). General Procedure A was followed using imine **1a** (55.0 mg, 0.300 mmol), 1,3-enyne **2a** (65.3 mg, 0.360 mmol), and 3,4,5-trimethoxyphenylboronic acid (95.4 mg, 0.450 mmol). The mixture was purified by column chromatography (25% EtOAc/petroleum ether) to give *allylation product* **3z** (113 mg, 70%) as a yellow oil (*ca.* 95% puritiy; small quantities of unidentified products remained). R_f = 0.17 (25% EtOAc/petroleum ether); $[\alpha]_D^{25.3}$ –86.5 (*c* 0.37, $CHCl_3$); IR 3255 (N-H), 2935, 1580, 1411, 1123, 758 cm^{-1}; ^{1}H NMR (400 MHz, $CDCl_3$) δ 7.35-7.30 (1H, m, Ar**H**), 7.27-7.20 (3H, m, Ar**H**), 7.20-7.13 (1H, m, Ar**H**), 7.10-7.02 (4H, m, Ar**H**), 6.30 (2H, s, Ar**H**), 5.45 (1H, d, *J* = 9.2 Hz, C=C**H**), 5.11 (1H, s, C=C**H$_A$**H$_B$), 5.04 (1H, dd, *J* = 6.7, 5.9 Hz, C**H**NH), 5.00 (1H, s, C=CH$_A$**H$_B$**), 4.16 (1H, d, *J* = 6.7 Hz, N**H**), 3.87 (3H, s, OC**H$_3$**), 3.83 (6H, s, 2 × OC**H$_3$**), 3.56 (1H, dd, *J* = 9.2, 5.9 Hz, C=CC**H**), 2.74-2.58 (3H, m, PhC**H$_2$** and PhCH$_2$C**H$_A$**H$_B$), 2.40 (1H, dt, *J* = 13.2, 7.7 Hz, PhCH$_2$CH$_A$**H$_B$**), 1.95 (3H, s, CC**H$_3$**); ^{13}C NMR (101 MHz, $CDCl_3$) δ 153.0 (2 × C), 151.5 (C), 143.5 (C), 143.1 (C), 141.5 (C), 137.63 (C), 137.61 (C), 129.2 (CH), 128.5 (2 × CH), 128.4 (2 × CH), 126.9 (CH), 126.3 (CH), 125.4 (CH), 124.3 (CH), 122.3 (C), 118.7 (CH), 115.6 (CH_2), 104.0 (2 × CH), 60.9 (CH), 56.9 (CH_3), 56.1 (2 × CH_3), 49.3 (CH), 33.8 (CH_2), 32.1 (CH_2), 21.5 (CH_3); HRMS (ESI) Exact mass calcd for $[C_{30}H_{33}NNaO_6S]^+$ $[M+Na]^+$: 558.1921, found: 558.1917; Enantiomeric excess was determined by HPLC using a Chiralcel OD-H column (85:15 *iso*-hexane:*i*-PrOH, 1.5 mL/min, 210 nm): t_r (major) = 6.6 min, t_r (major) = 14.6 min, 99% ee.

4. Investigation of Deuterium Transfer

(±)-(4*S*)-4-[(3*R*,*E*)-2-(Methyl-d$_3$)-5,7-diphenylhepta-1,4-dien-3-yl-1,1,4-d$_3$]-3,4-dihydrobenzo[*e*][1,2,3]oxathiazine 2,2-dioxide ([D]$_6$-3a)

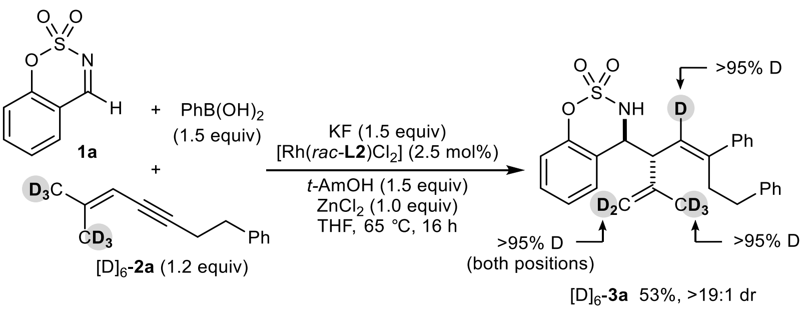

Imine **1a** (36.6 mg, 0.200 mmol), 1,3-enyne [D]$_6$-**2a** (45.6 mg, 0.240 mmol), phenylboronic acid (36.6 mg, 0.150 mmol), KF (17.4 mg, 0.300 mmol), and [Rh(*rac*-**L2**)Cl]$_2$ (4.4 mg, 0.0056 mmol) were added to an oven-dried microwave vial. The vial was purged with nitrogen for 1 h and degassed THF (1 mL), *t*-amyl alcohol (32 μL, 0.30 mmol) and $ZnCl_2$ solution (0.7 M in THF, 0.28 mL, 0.20 mmol) were added. The reaction mixture was stirred for 16 h at 65 °C. The mixture was cooled to room temperature, diluted with saturated aqueous NH_4Cl solution (4 mL) and water (4 mL), and extracted with EtOAc (3 × 5 mL). The combined organic phases were washed with brine, dried (Na_2SO_4), filtered, and concentrated *in vacuo*. The mixture was purified twice by column chromatography (first column with 5% EtOAc/*iso*-hexane; second column with 60:39:1 *iso*-hexane/CH_2Cl_2/EtOAc) to give *allylation product* [D]$_6$-**3a** (48 mg, 53%) as a pale yellow oil (*ca.* 97% purity; small quantities of solvents and silicone grease remained). R_f = 0.38 (15% EtOAc/petroleum ether); IR 3267 (N-H), 3025, 1418, 1193, 1169, 753, 697 cm^{-1}; ^{1}H NMR (500 MHz, $CDCl_3$) δ 7.36-7.26 (4H, m, Ar**H**), 7.24-7.16 (5H, m, Ar**H**), 7.14 (1H, td, *J* = 7.6, 1.2 Hz, Ar**H**), 7.10-7.07 (1H, m, Ar**H**), 7.06-7.02 (3H, m, Ar**H**), 4.98 (1H, dd, *J* = 7.3, 6.3 Hz, C**H**NH), 4.03 (1H, d, *J* = 7.3 Hz, N**H**), 3.53 (1H, d, *J* = 6.3 Hz, C=CC**H**), 2.83-2.68 (2H, m, PhC**H$_2$**), 2.62 (1H, dt, *J* = 13.6, 6.6 Hz, PhCH$_2$**H$_A$**H$_B$), 2.40 (1H, dt, *J* = 13.6, 8.1 Hz, PhCH$_2$H$_A$**H$_B$**); ^{13}C NMR (126 MHz, $CDCl_3$) δ 151.4 (C), 143.12 (C), 143.05 (C), 141.6 (C), 141.5 (C), 129.3 (CH), 128.5 (2 × CH), 128.5 (2 × CH), 128.4 (2 × CH), 127.5 (CH), 126.7 (CH), 126.7 (2 × CH), 126.3 (CH), 125.2 (CH), 122.1 (C), 118.8 (CH_2), 56.9 (CH), 49.0 (CH), 33.7 (CH_2), 31.9 (CH_2); HRMS (ESI) Exact mass calcd for $[C_{27}H_{21}D_6NNaO_3S]^+$ $[M+Na]^+$: 474.1980, found: 474.1983.

5. Measurement of Kinetic Isotope Effect in an Intermolecular Competition Experiment

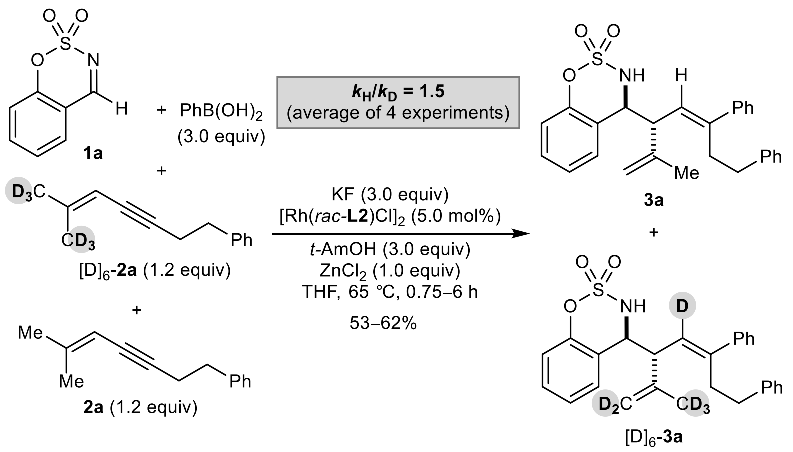

Imine **1a** (9.1 mg, 0.050 mmol), 1,3-enyne **2a** (11.1 mg, 0.0602 mmol), 1,3-enyne [D_6]-**2a** (11.4 mg, 0.0599 mmol), phenylboronic acid (18.3 mg, 0.150 mmol), KF (8.7 mg, 0.15 mmol), and [Rh(*rac*-**L2**)Cl]$_2$ (2.3 mg, 0.0023 mmol) were added to an oven-dried microwave vial. The vial was purged with nitrogen for 1 h and degassed THF (1 mL), *t*-amyl alcohol (18 µL, 0.15 mmol) and $ZnCl_2$ solution (0.7 M in THF, 0.07 mL, 0.05 mmol) were added. The reaction mixture was stirred at 65 °C for 0.75–6 h. The mixture was cooled to room temperature, and the mixture was diluted with saturated aqueous NH_4Cl solution (1 mL) and EtOAc (2 mL). 1,3,5-Trimethoxybenzene was added as an internal standard, and the organic phase was separated and concentrated *in vacuo*. The crude material was analyzed by ^{1}H NMR spectroscopy.

Run 1: time: 3 h; combined yield of **3a** and [D_6]-**3a** = 62% (20% of **1a** remaining). k_H/k_D = 1.35
Run 2: time: 3 h; combined yield of **3a** and [D_6]-**3a** = 60% (35% of **1a** remaining). k_H/k_D = 1.47
Run 3: time: 6 h; combined yield of **3a** and [D_6]-**3a** = 53% (37% of **1a** remaining). k_H/k_D = 1.55
Run 4: time: 45 min; combined yield of **3a** and [D_6]-**3a** = 37% (52% of **1a** remaining). k_H/k_D = 1.45

The heterogeneity of the reactions prevented the reliable determination of the rate constants of separate experiments containing either **2a** or [D]$_6$-**2a** only. Therefore, it was not possible to conclude whether alkenyl-to-allyl 1,4-rhodium(I) migration is the turnover-limiting step.

6. NMR Spectra

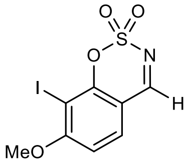

1e

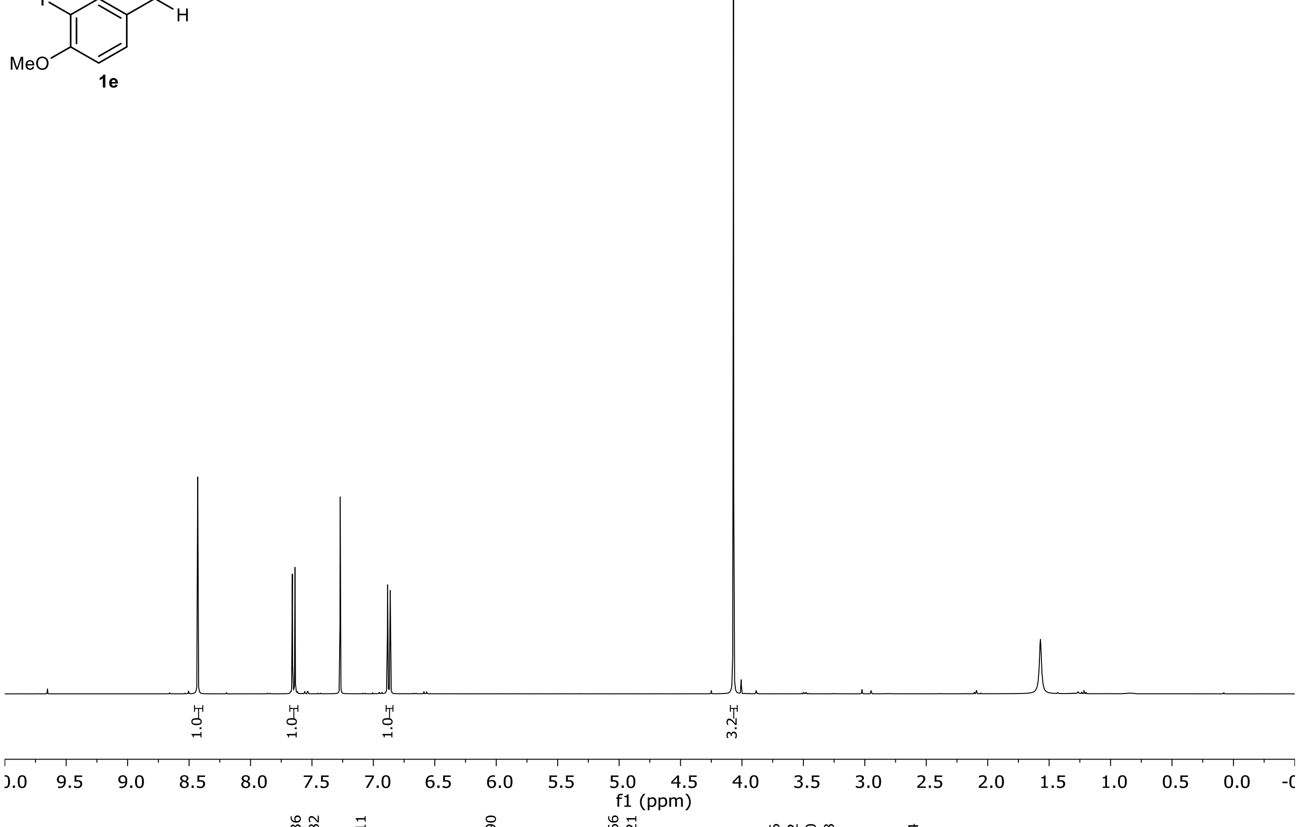

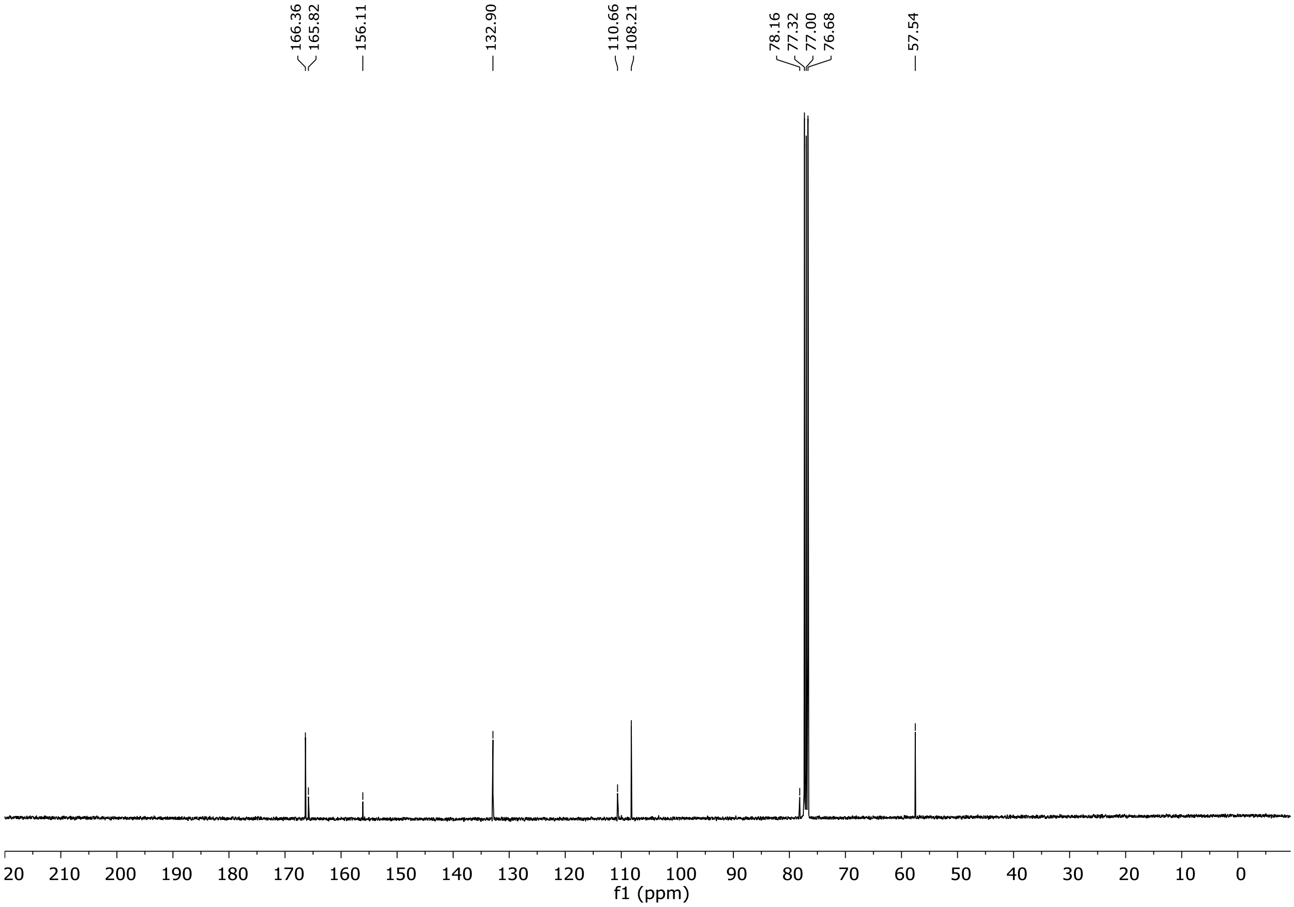

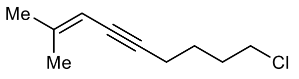
Me
Me
Cl

2b

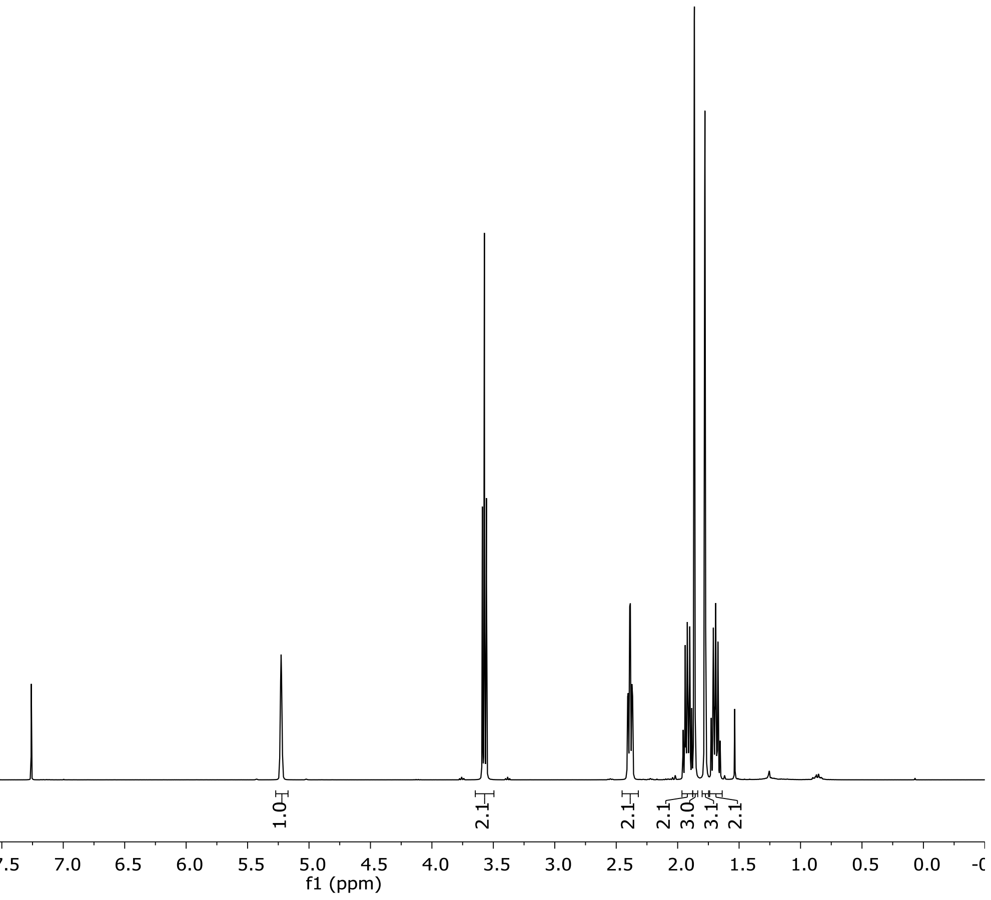
1.0
2.1
2.1
2.1
3.0
3.1
2.1
f1 (ppm)

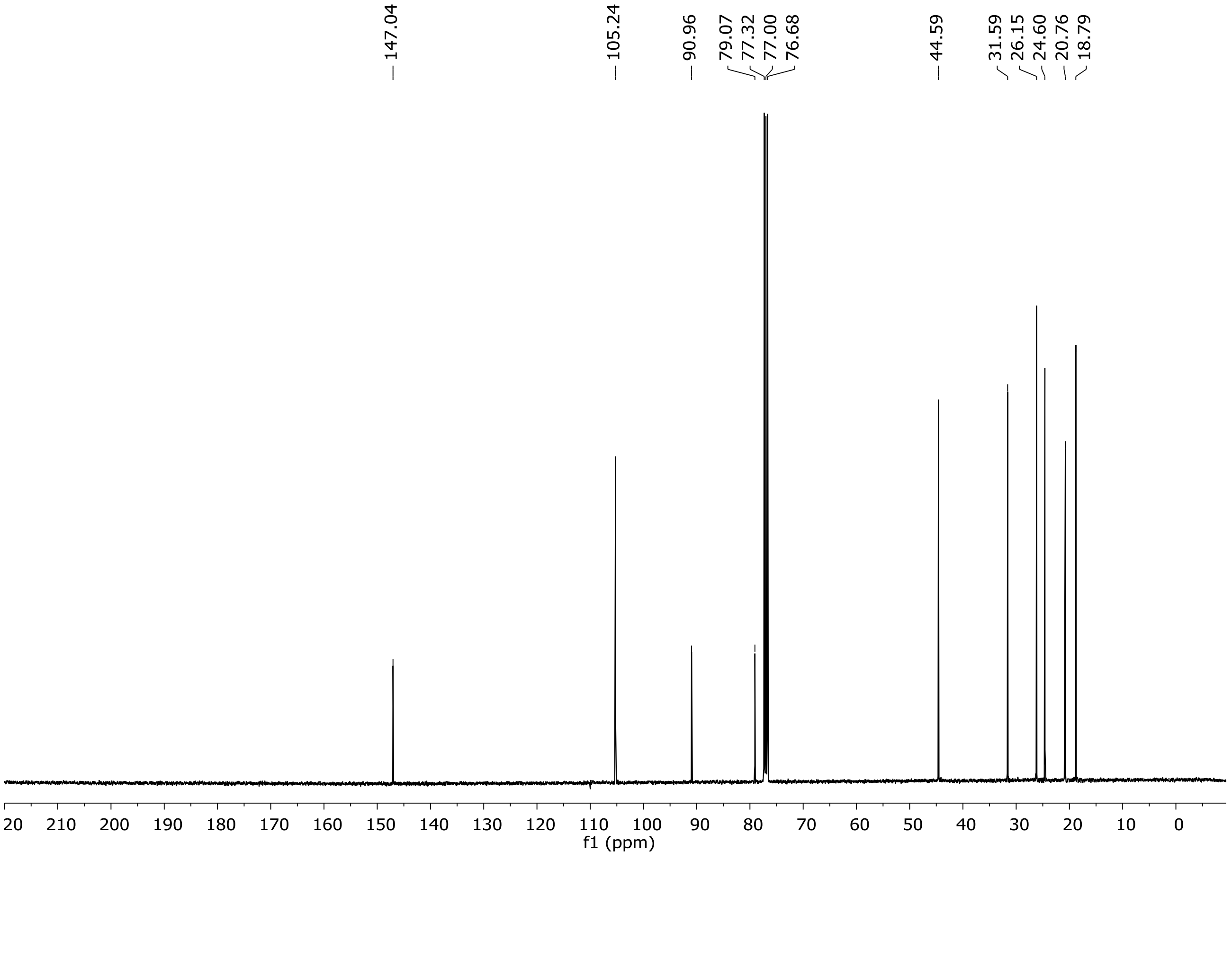
147.04
105.24
90.96
79.07
77.32
77.00
76.68
44.59
31.59
26.15
24.60
20.76
18.79
f1 (ppm)

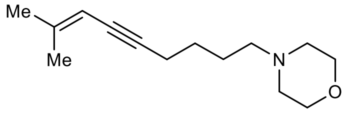
Me
Me
N
O

2d

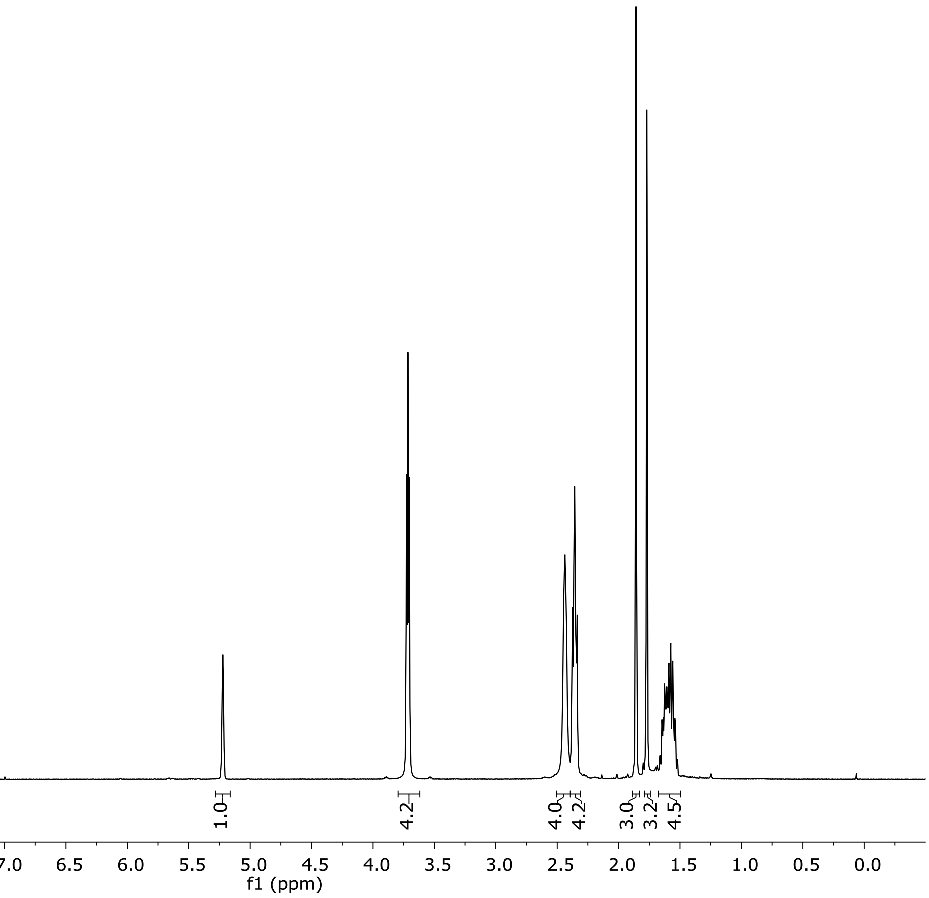
1.0
4.2
4.0
4.2
3.0
3.2
4.5
).0 9.5 9.0 8.5 8.0 7.5 7.0 6.5 6.0 5.5 5.0 4.5 4.0 3.5 3.0 2.5 2.0 1.5 1.0 0.5 0.0
f1 (ppm)

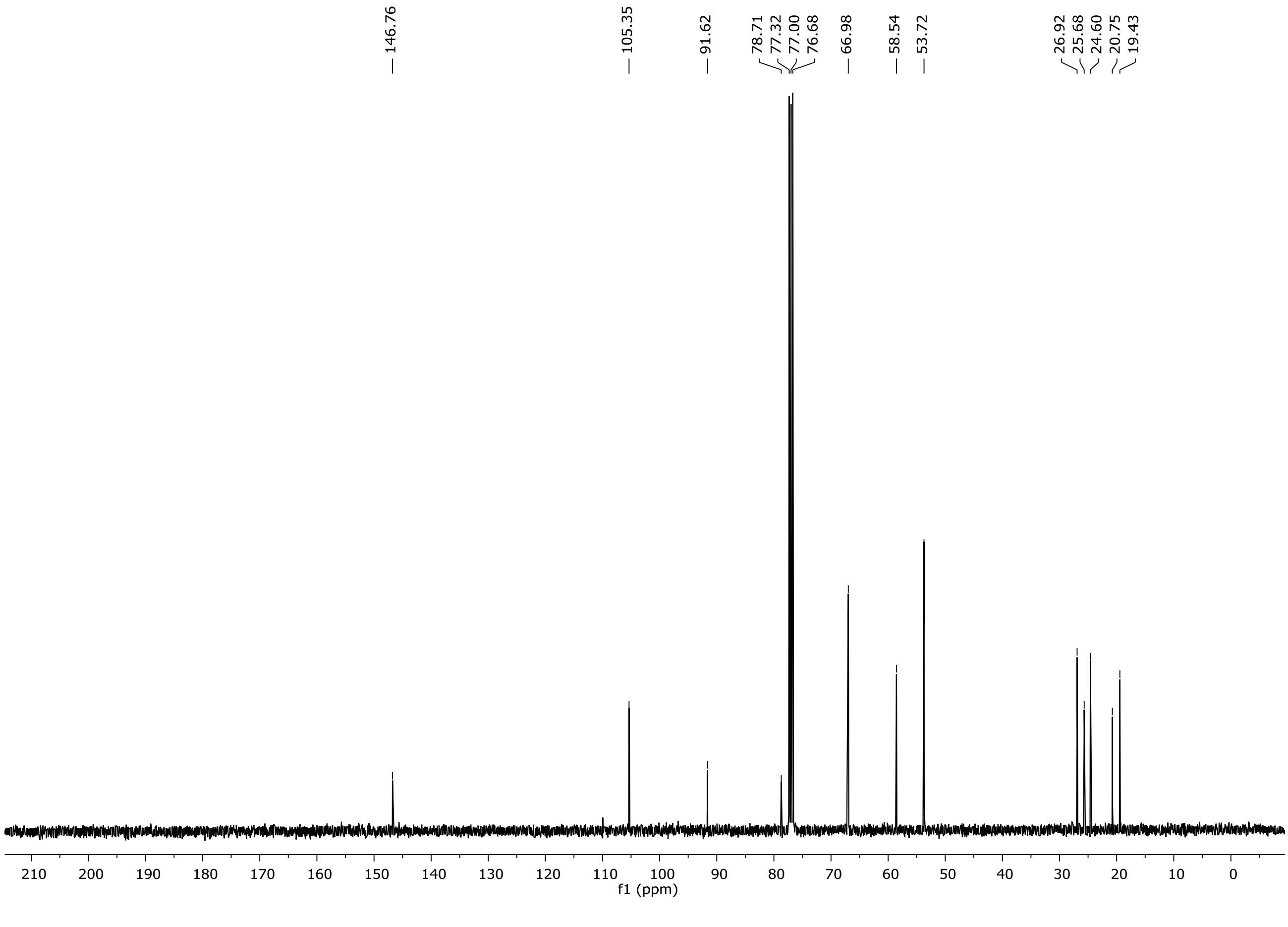
146.76
105.35
91.62
78.71
77.32
77.00
76.68
66.98
58.54
53.72
26.92
25.68
24.60
20.75
19.43
210 200 190 180 170 160 150 140 130 120 110 100 90 80 70 60 50 40 30 20 10 0
f1 (ppm)

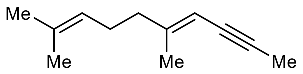
Me
Me
Me
Me

2g (*E*/*Z*: 5.8:1)

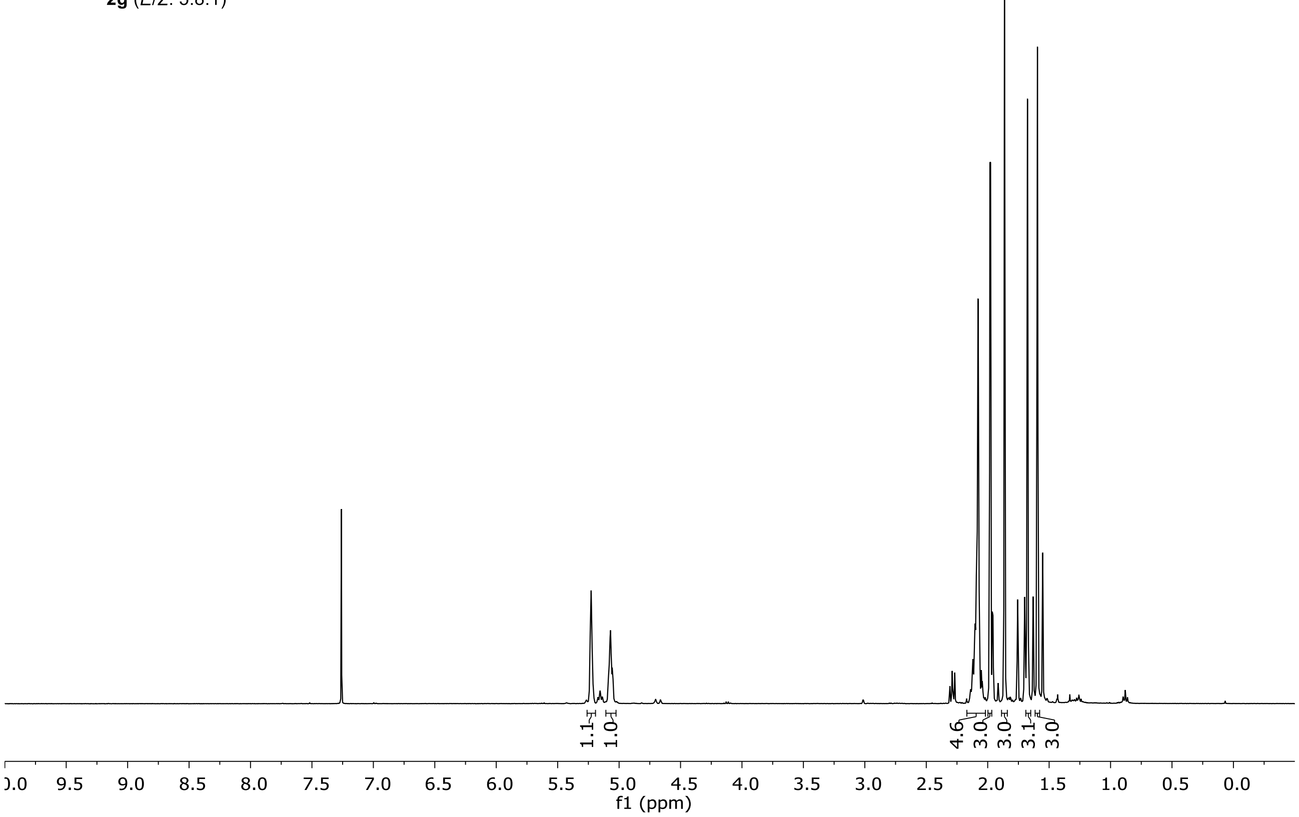
1.1
1.0
4.6
3.0
3.0
3.1
3.0
f1 (ppm)

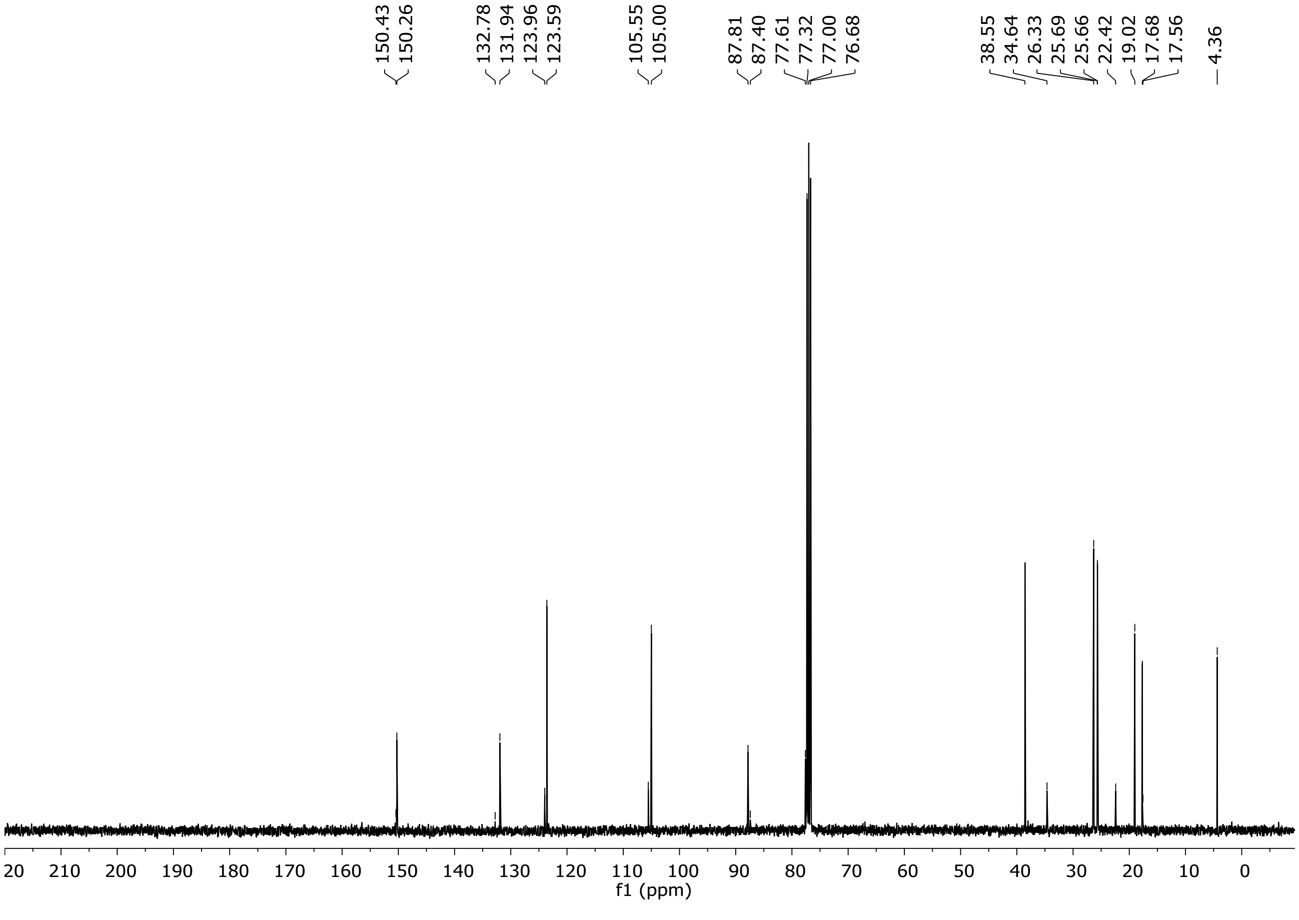
150.43
150.26
132.78
131.94
123.96
123.59
105.55
105.00
87.81
87.40
77.61
77.32
77.00
76.68
38.55
34.64
26.33
25.69
25.66
22.42
19.02
17.68
17.56
4.36
f1 (ppm)

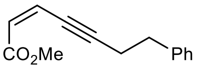
CO2Me
Ph

S3

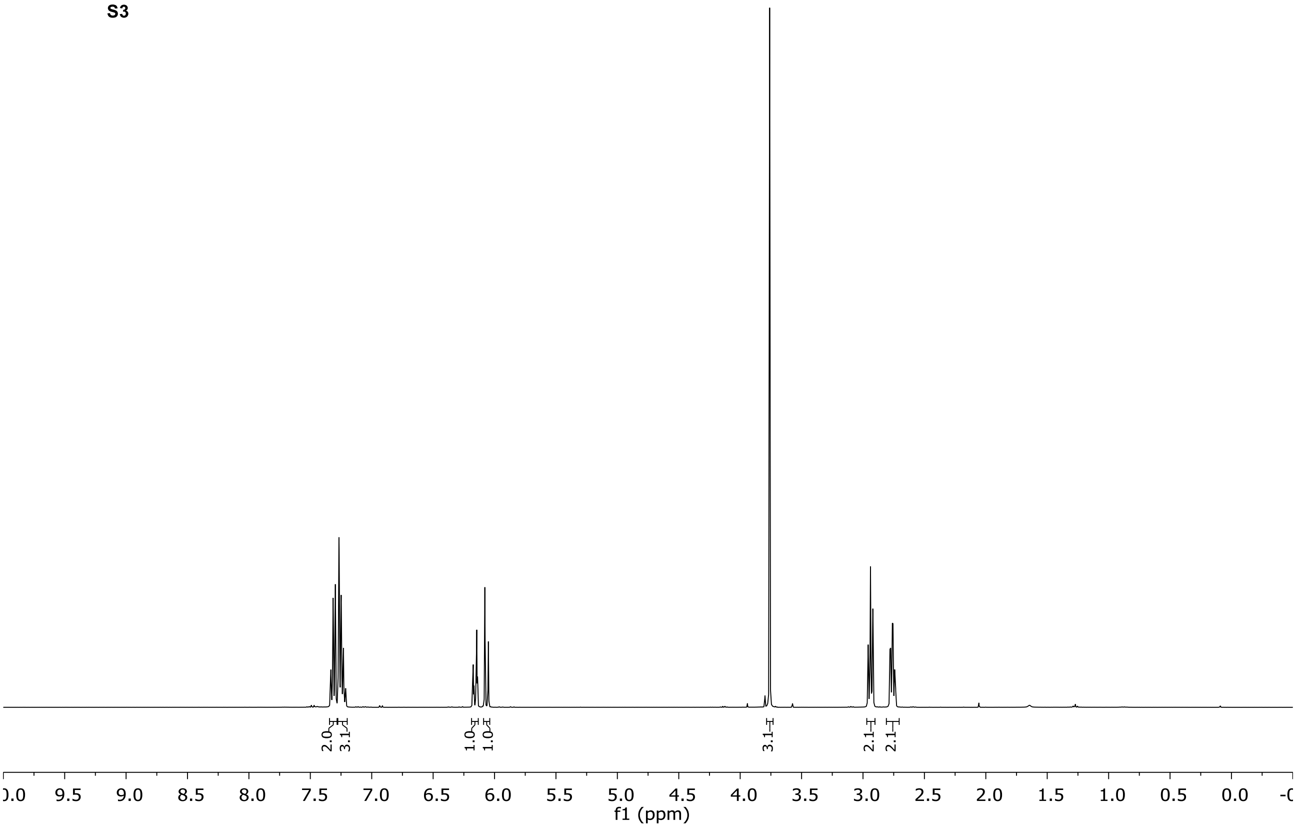
2.0
3.1
1.0
1.0
3.1
2.1
2.1
f1 (ppm)

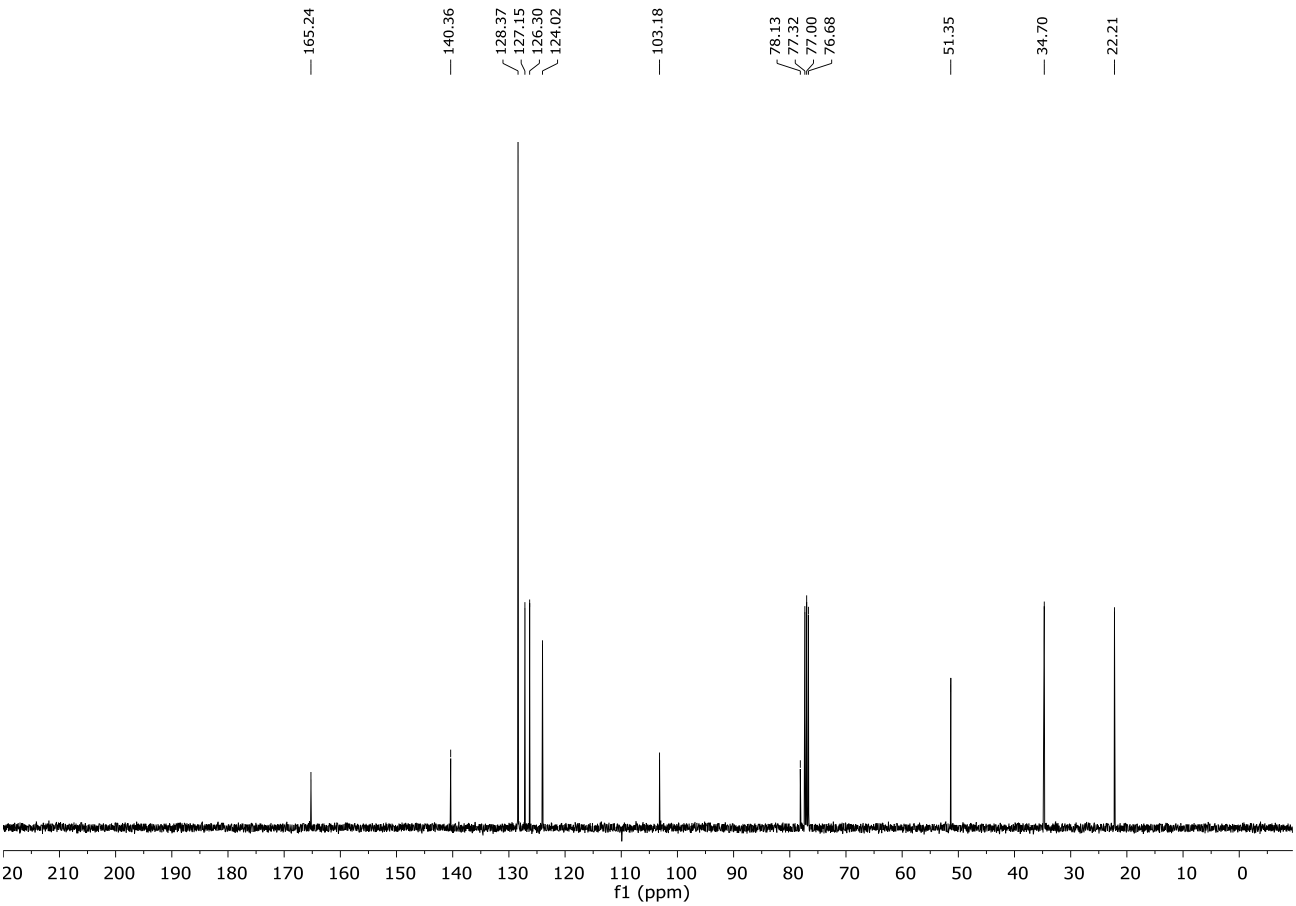
165.24
140.36
128.37
127.15
126.30
124.02
103.18
78.13
77.32
77.00
76.68
51.35
34.70
22.21
f1 (ppm)

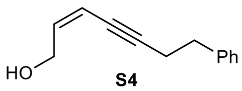
HO
Ph

S4

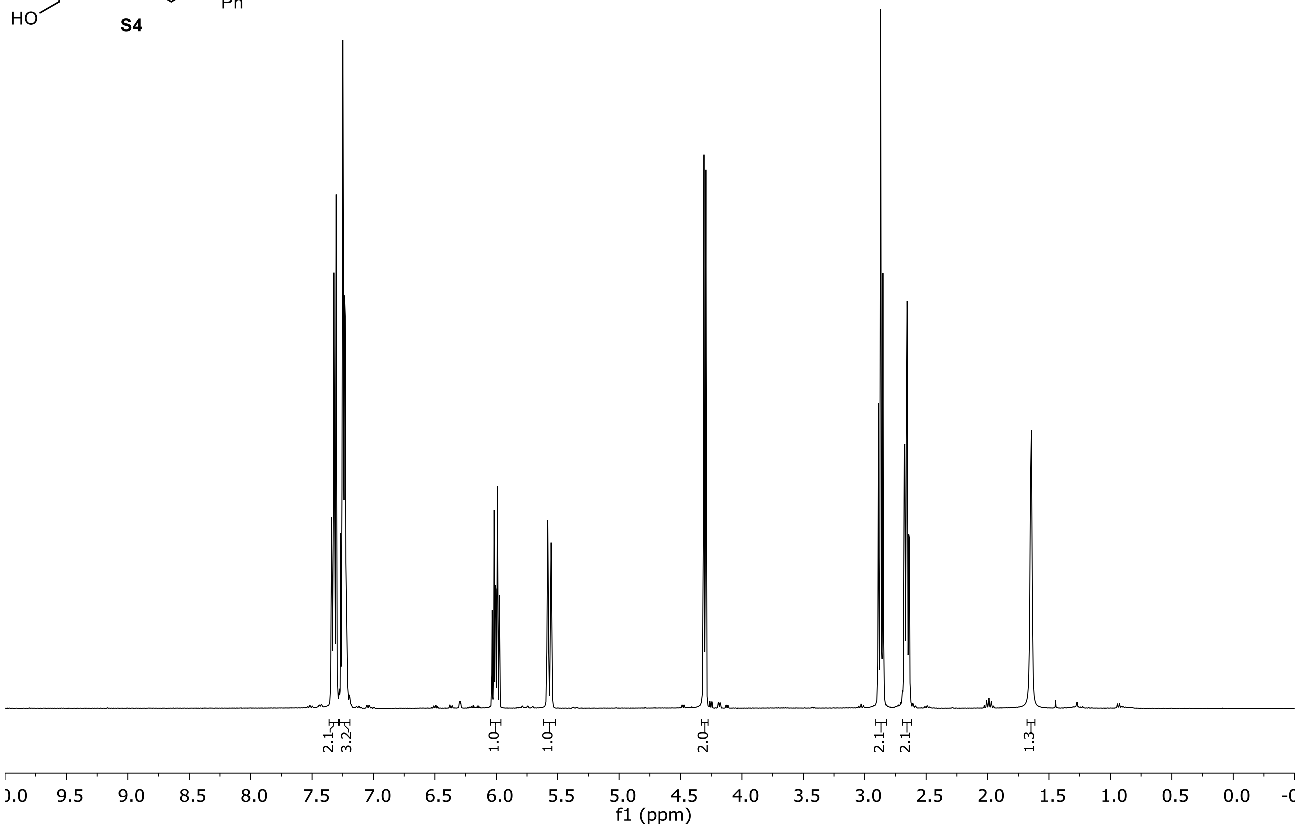
2.1
3.2
1.0
1.0
2.0
2.1
2.1
1.3
f1 (ppm)

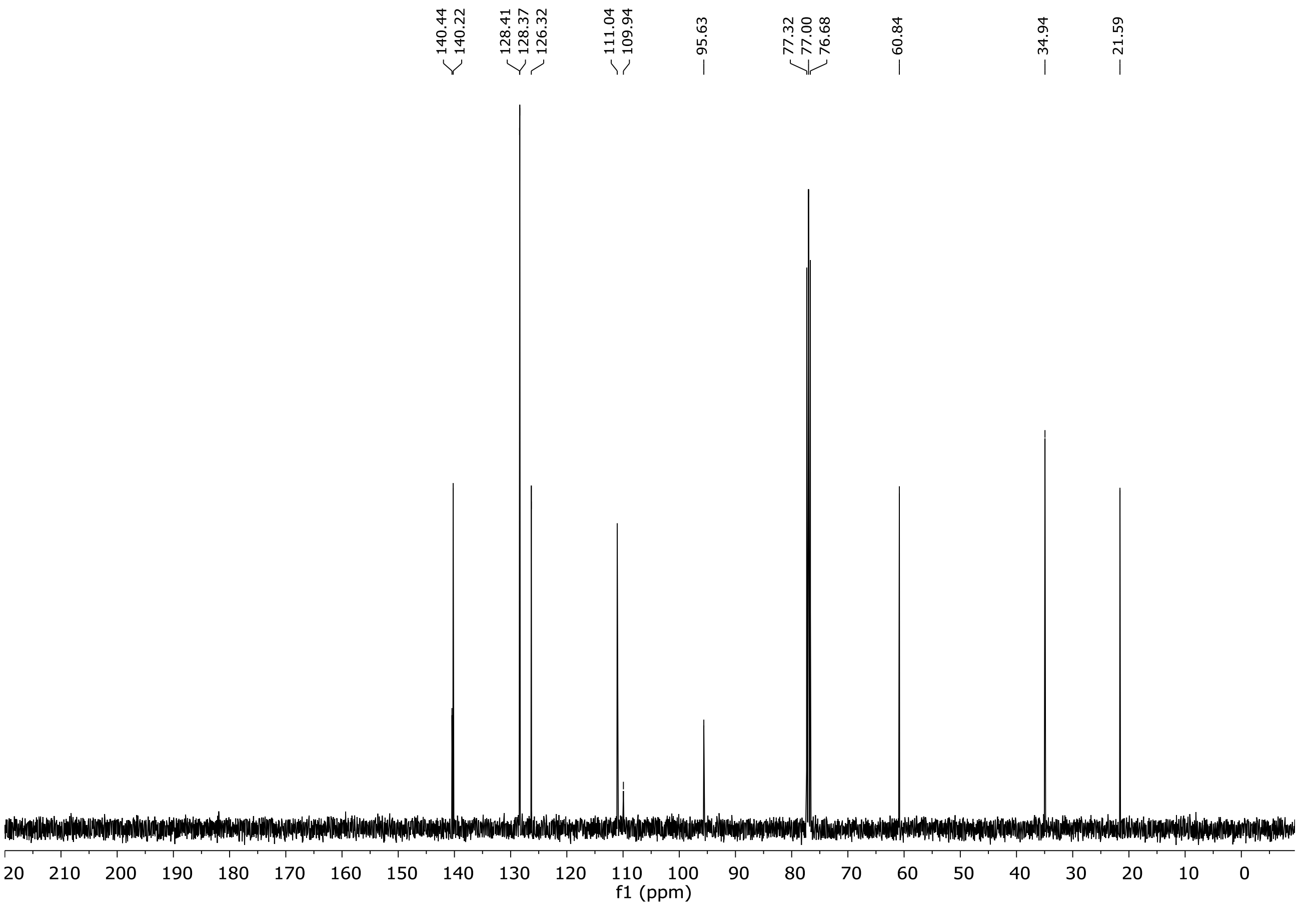
140.44
140.22
128.41
128.37
126.32
111.04
109.94
95.63
77.32
77.00
76.68
60.84
34.94
21.59
f1 (ppm)

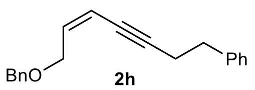
BnO
Ph

2h

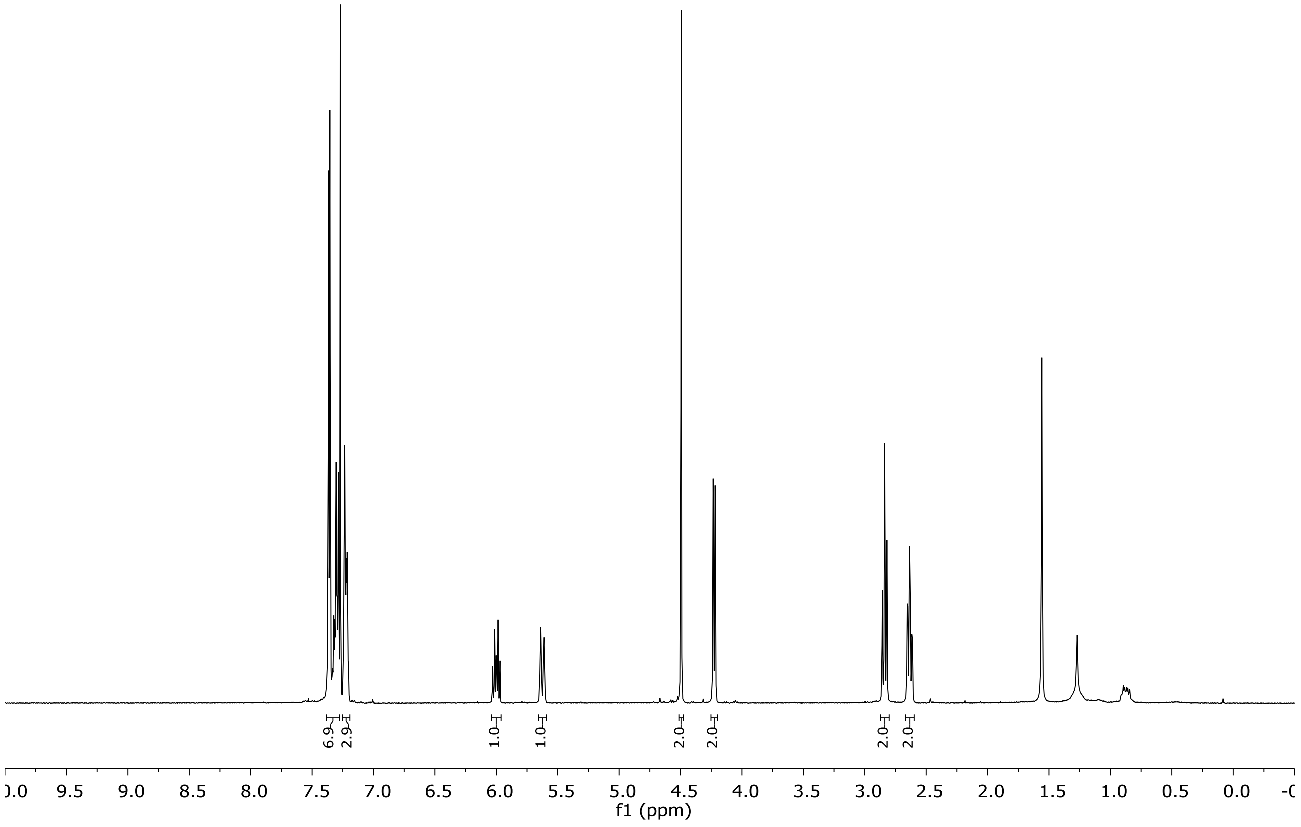
6.9
2.9
1.0
1.0
2.0
2.0
2.0
2.0
f1 (ppm)

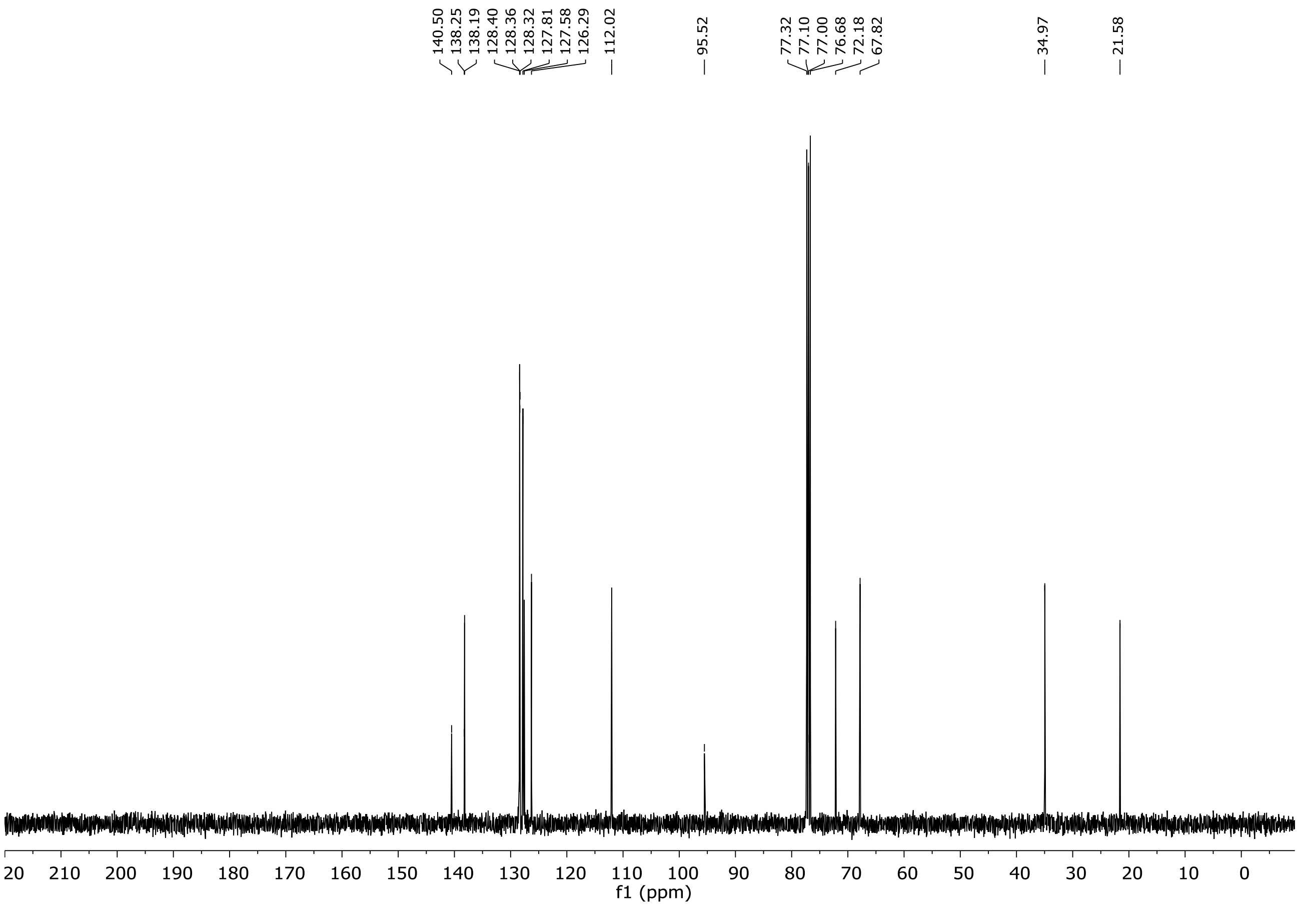
140.50
138.25
138.19
128.40
128.36
128.32
127.81
127.58
126.29
112.02
95.52
77.32
77.10
77.00
76.68
72.18
67.82
34.97
21.58
f1 (ppm)

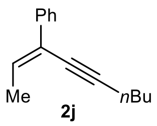
Ph
Me
nBu

2j

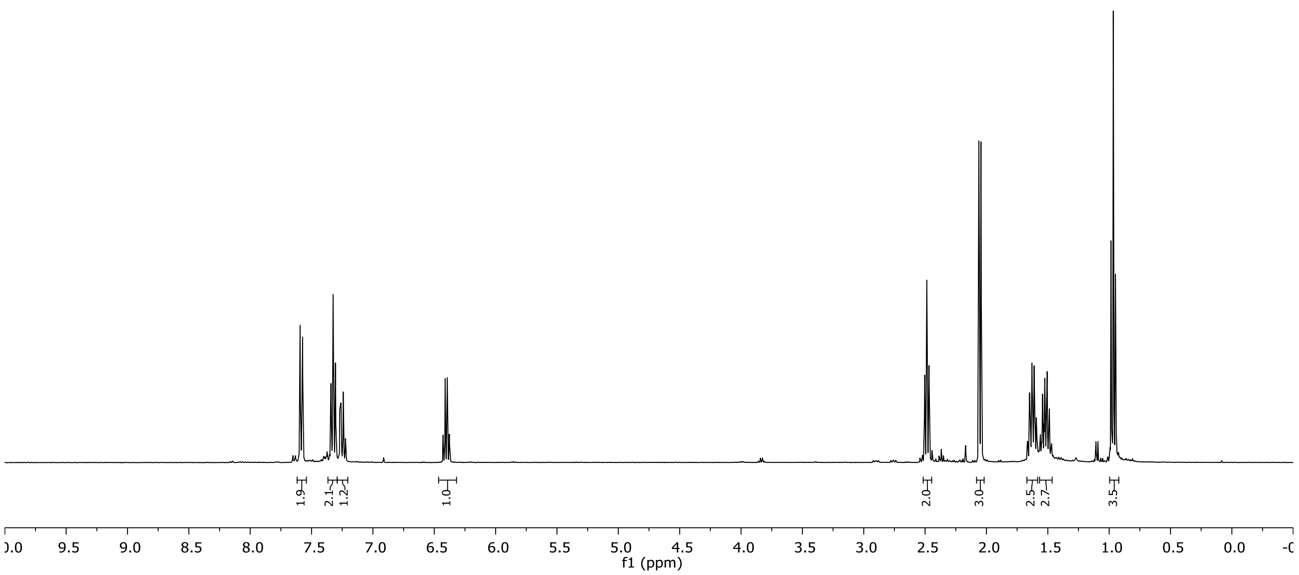
1.9
2.1
1.2
1.0
2.0
3.0
2.5
2.7
3.5
f1 (ppm)

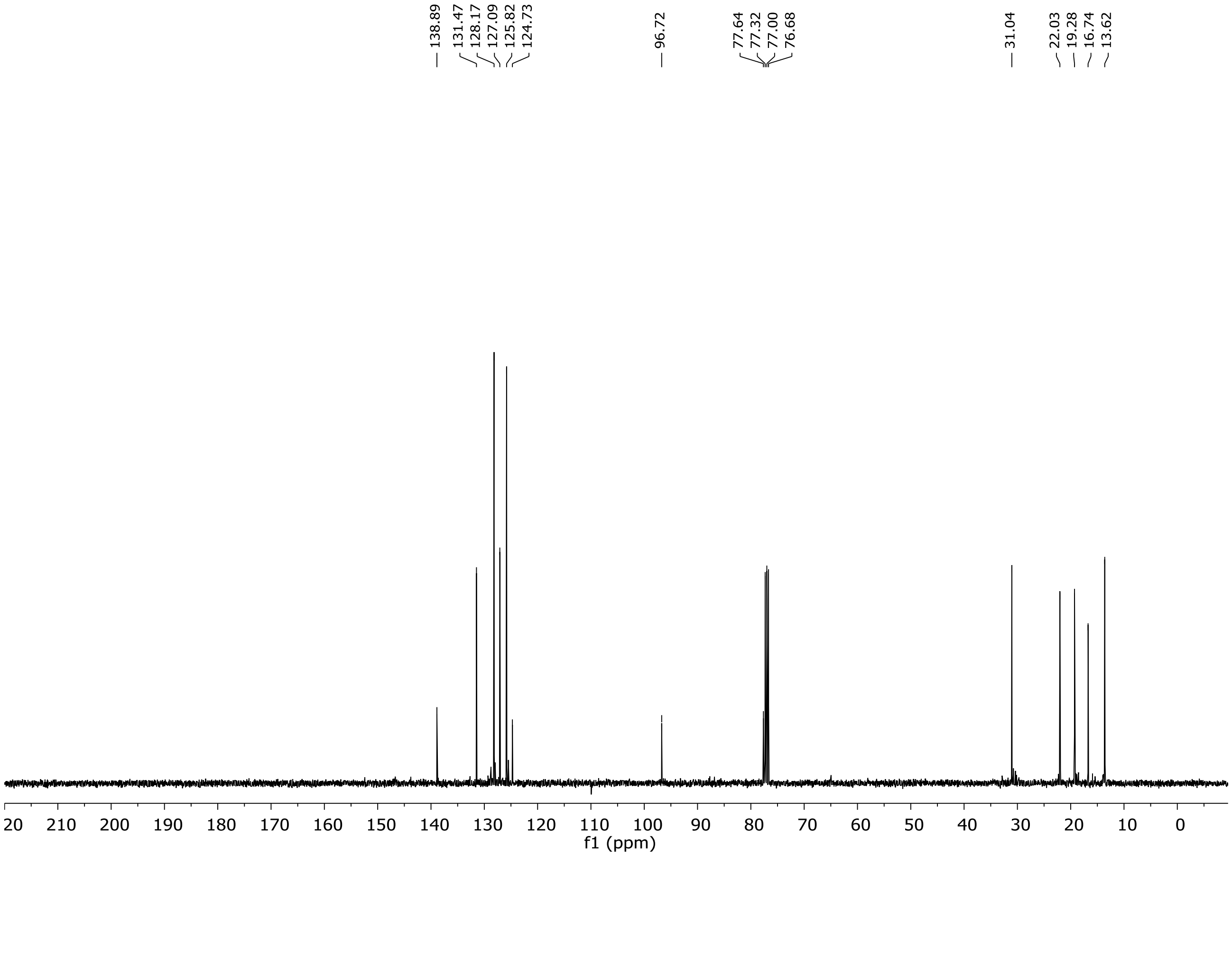
138.89
131.47
128.17
127.09
125.82
124.73
96.72
77.64
77.32
77.00
76.68
31.04
22.03
19.28
16.74
13.62
f1 (ppm)

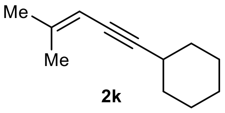
Me
Me

2k

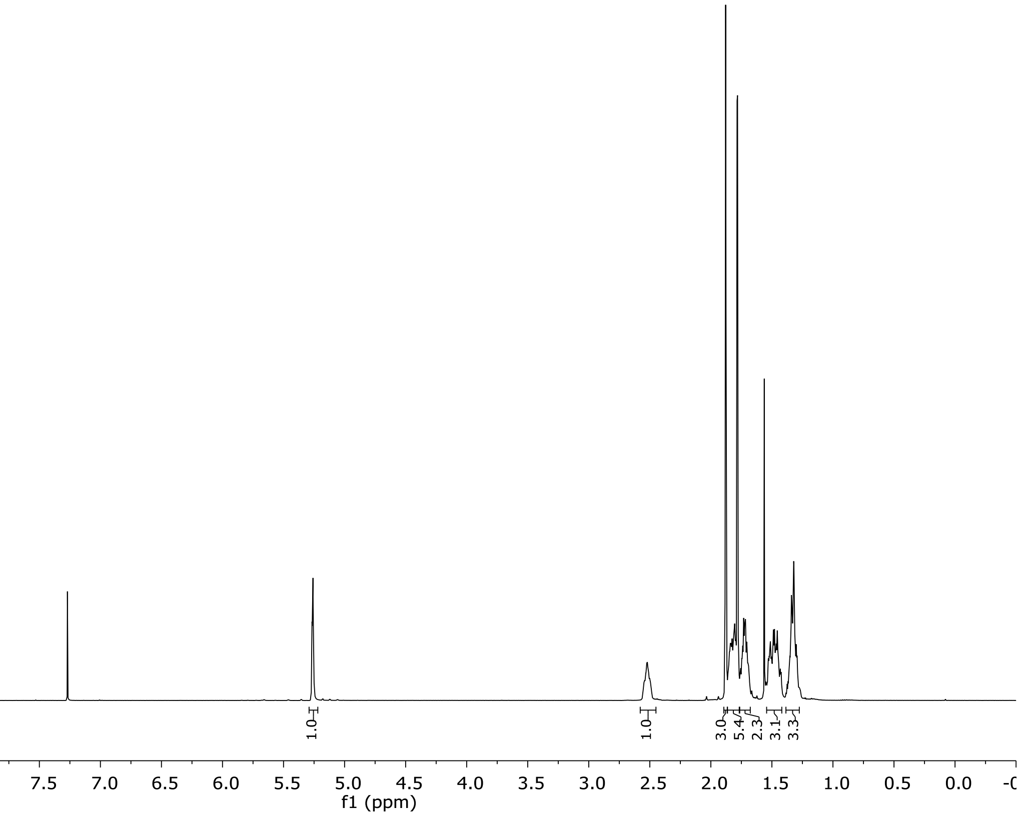
1.0
1.0
3.0
5.4
2.3
3.1
3.3
f1 (ppm)

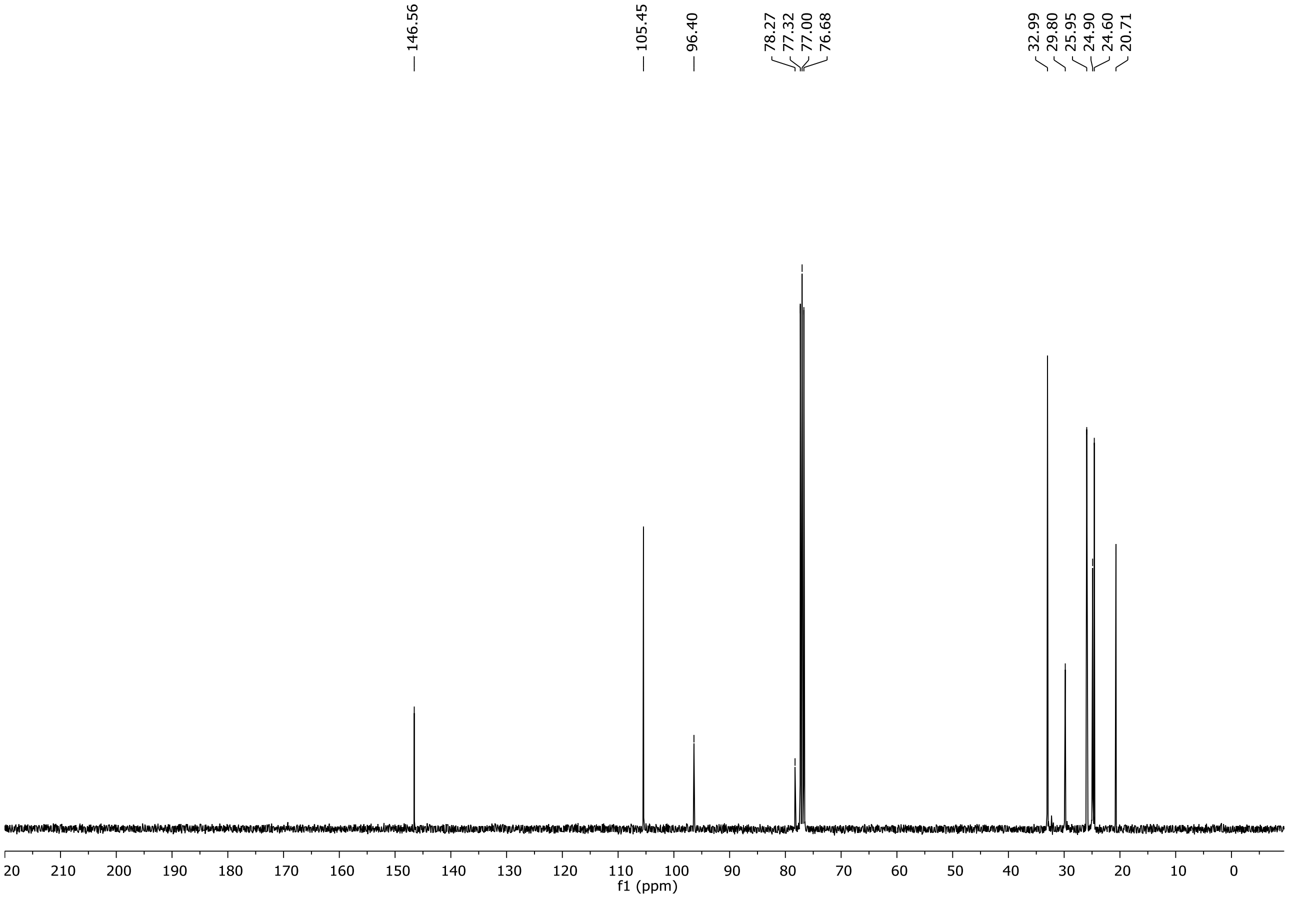
146.56
105.45
96.40
78.27
77.32
77.00
76.68
32.99
29.80
25.95
24.90
24.60
20.71
f1 (ppm)

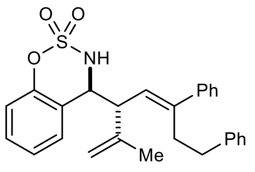

3a

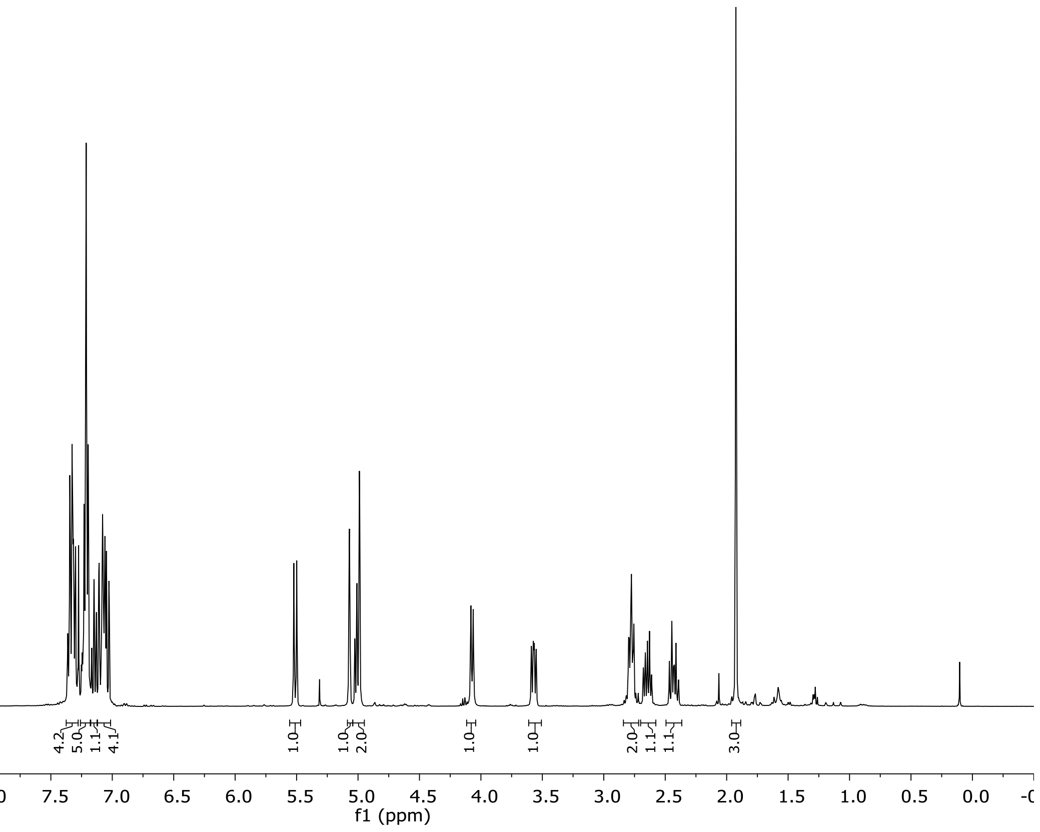
4.2
5.0
1.1
1.1
4.1
1.0
1.0
2.0
1.0
1.0
2.0
1.1
1.1
3.0
f1 (ppm)

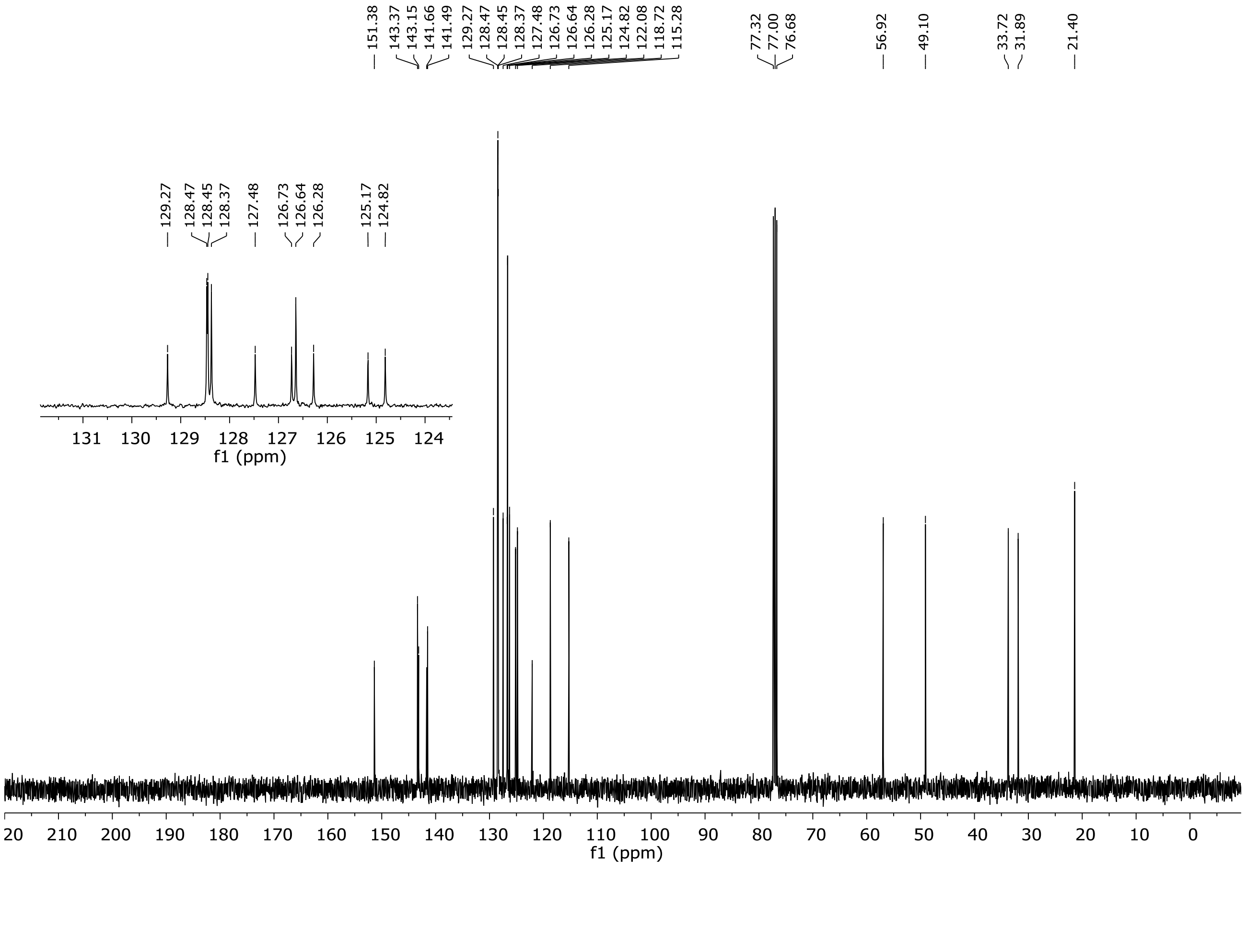
151.38
143.37
143.15
141.66
141.49
129.27
128.47
128.45
128.37
127.48
126.73
126.64
126.28
125.17
124.82
122.08
118.72
115.28
77.32
77.00
76.68
56.92
49.10
33.72
31.89
21.40
f1 (ppm)

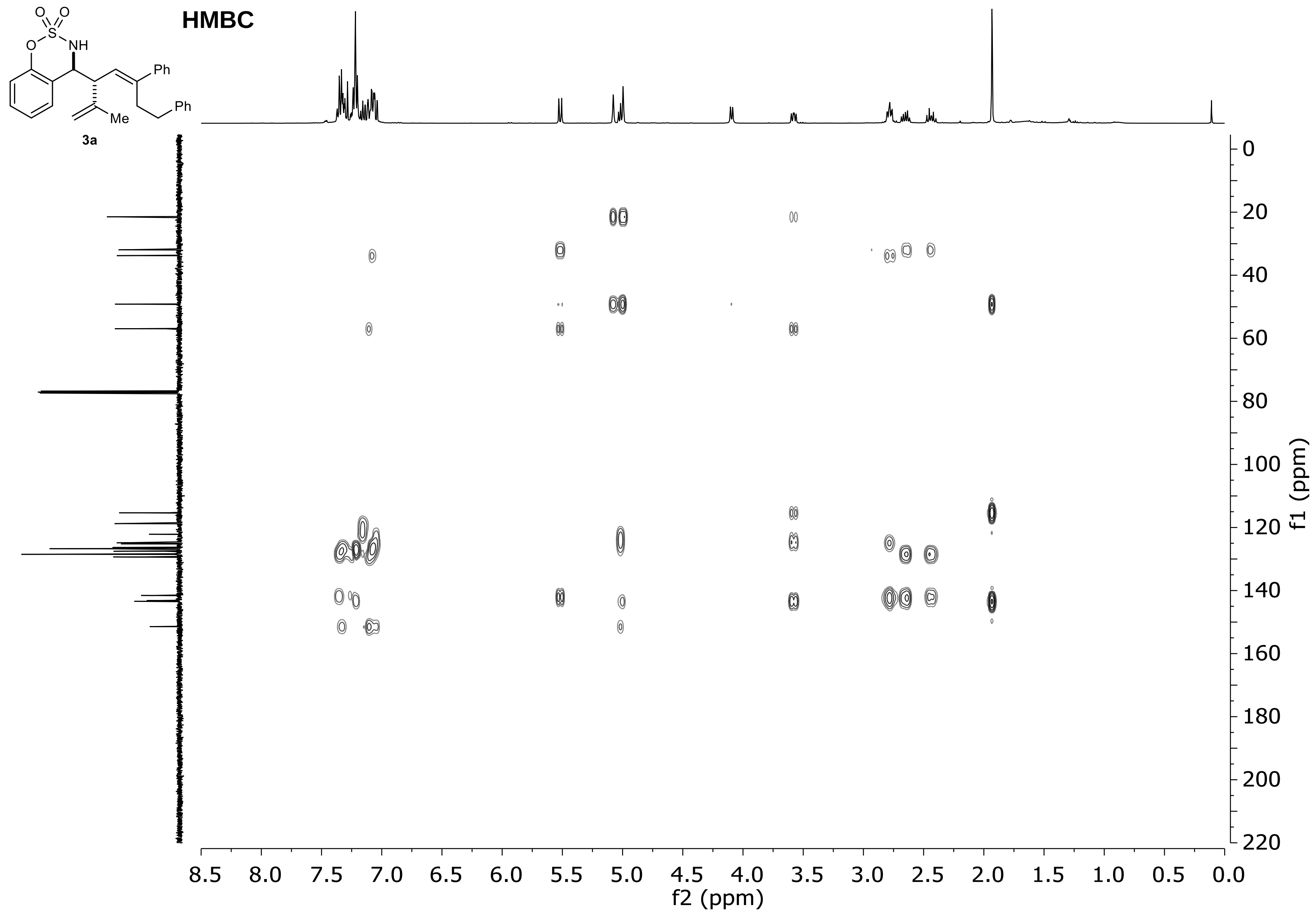
O O
S
O NH
Ph
Me
Ph
3a
HMBC
f2 (ppm)
f1 (ppm)
8.5 8.0 7.5 7.0 6.5 6.0 5.5 5.0 4.5 4.0 3.5 3.0 2.5 2.0 1.5 1.0 0.5 0.0
0 20 40 60 80 100 120 140 160 180 200 220

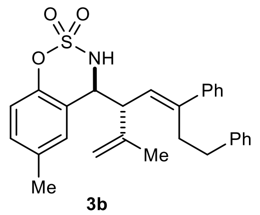
O
O
S
O
NH
Ph
Ph
Me
Me

3b

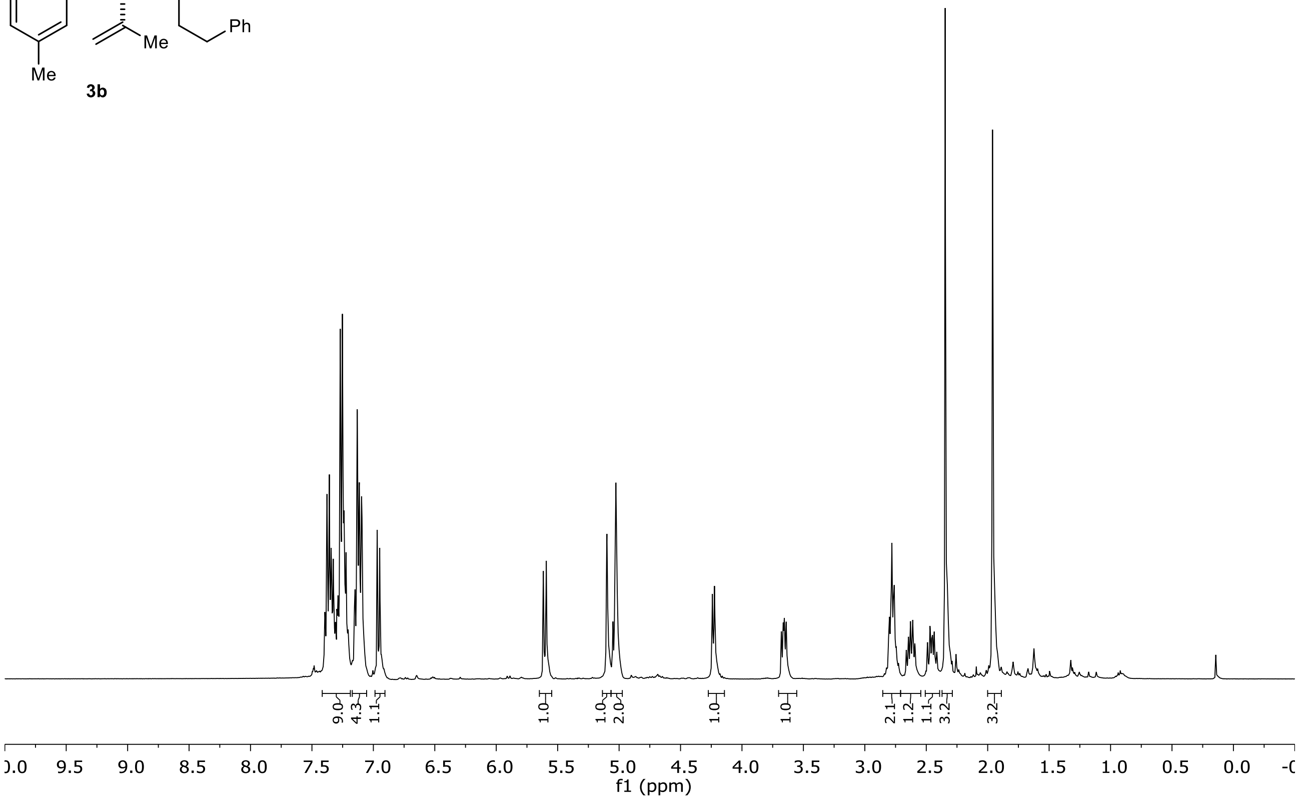
9.0
4.3
1.1
1.0
1.0
2.0
1.0
1.0
2.1
1.2
1.1
3.2
3.2
f1 (ppm)

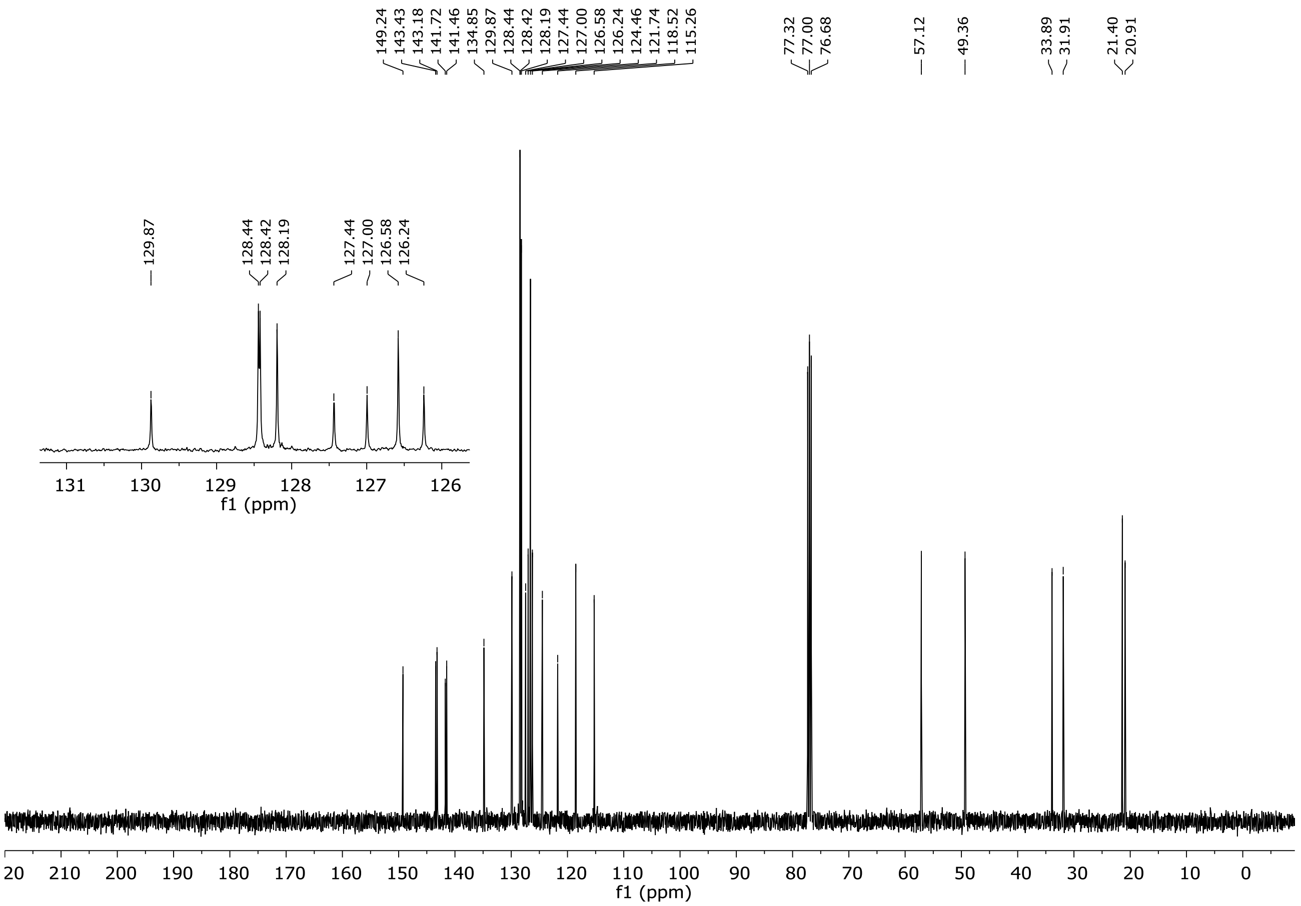
149.24
143.43
143.18
141.72
141.46
134.85
129.87
128.44
128.42
128.19
127.44
127.00
126.58
126.24
124.46
121.74
118.52
115.26
77.32
77.00
76.68
57.12
49.36
33.89
31.91
21.40
20.91
f1 (ppm)

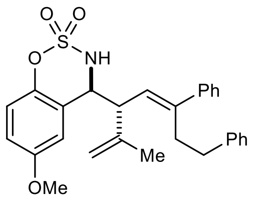
O
O
S
O
NH
Ph
Ph
Me
OMe

3c

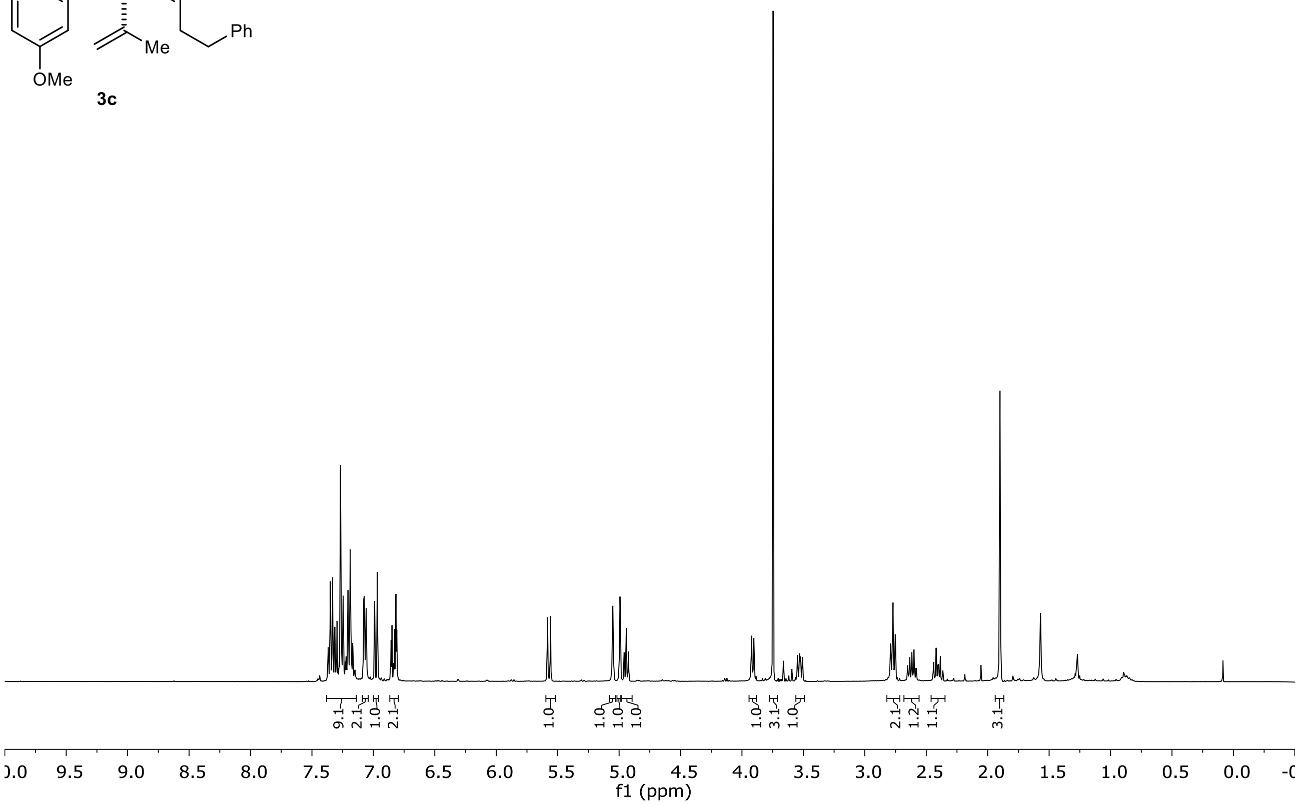
9.1
2.1
1.0
2.1
1.0
1.0
1.0
1.0
1.0
3.1
1.0
2.1
1.2
1.1
3.1
f1 (ppm)

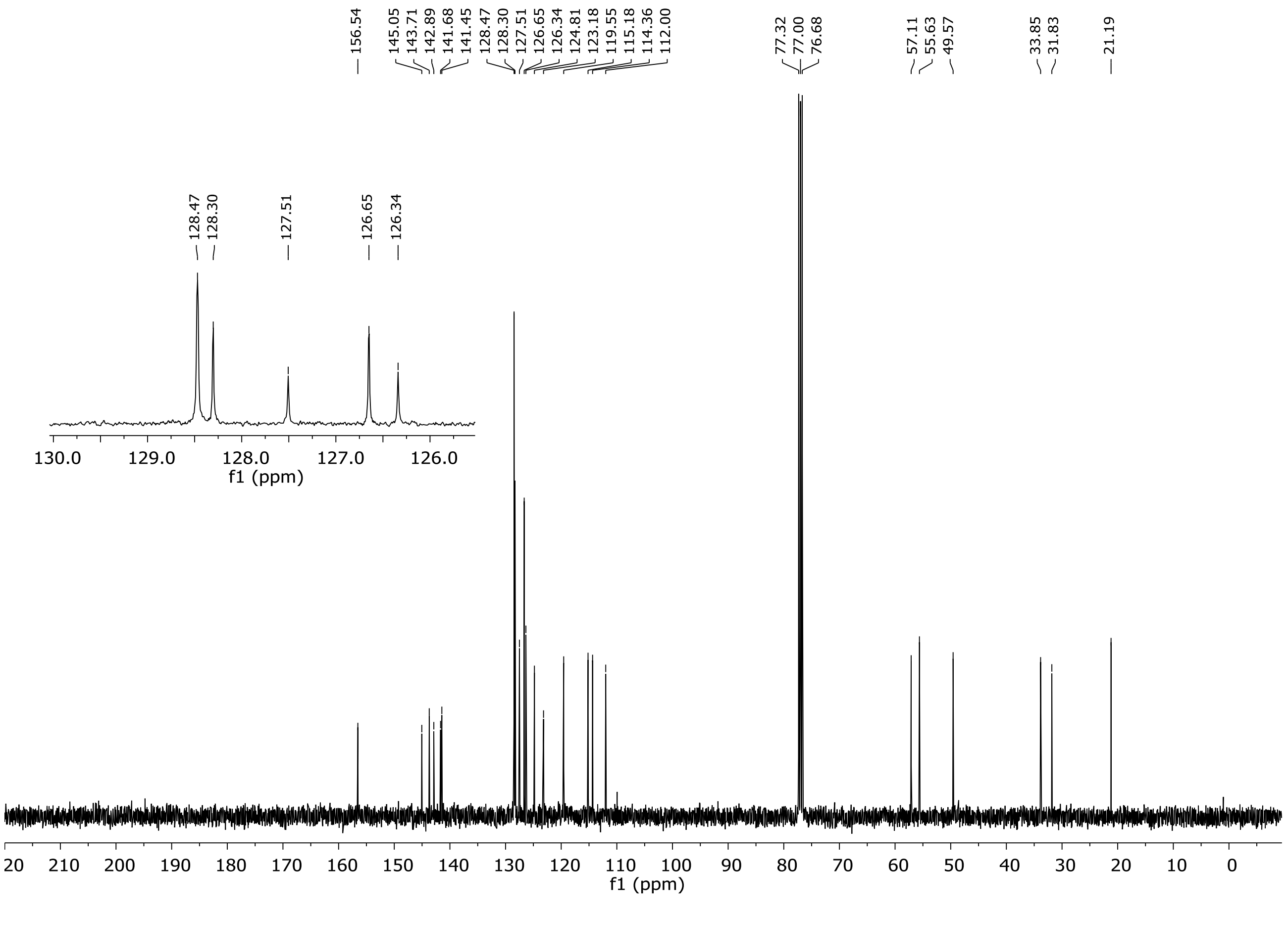
156.54
145.05
143.71
142.89
141.68
141.45
128.47
128.30
127.51
126.65
126.34
124.81
123.18
119.55
115.18
114.36
112.00
77.32
77.00
76.68
57.11
55.63
49.57
33.85
31.83
21.19
128.47
128.30
127.51
126.65
126.34
f1 (ppm)
f1 (ppm)

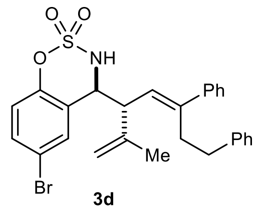
O
O
S
NH
O
Ph
Ph
Me
Br

3d

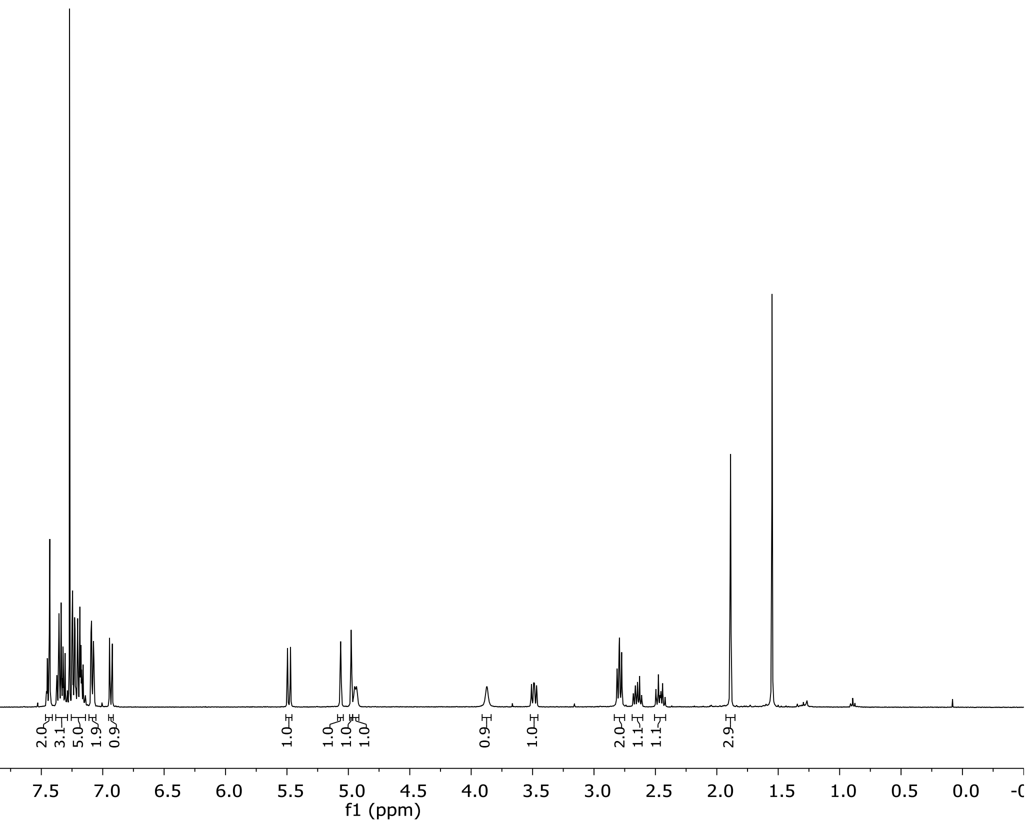
2.0
3.1
5.0
1.9
0.9
1.0
1.0
1.0
1.0
0.9
1.0
2.0
1.1
1.1
2.9
f1 (ppm)

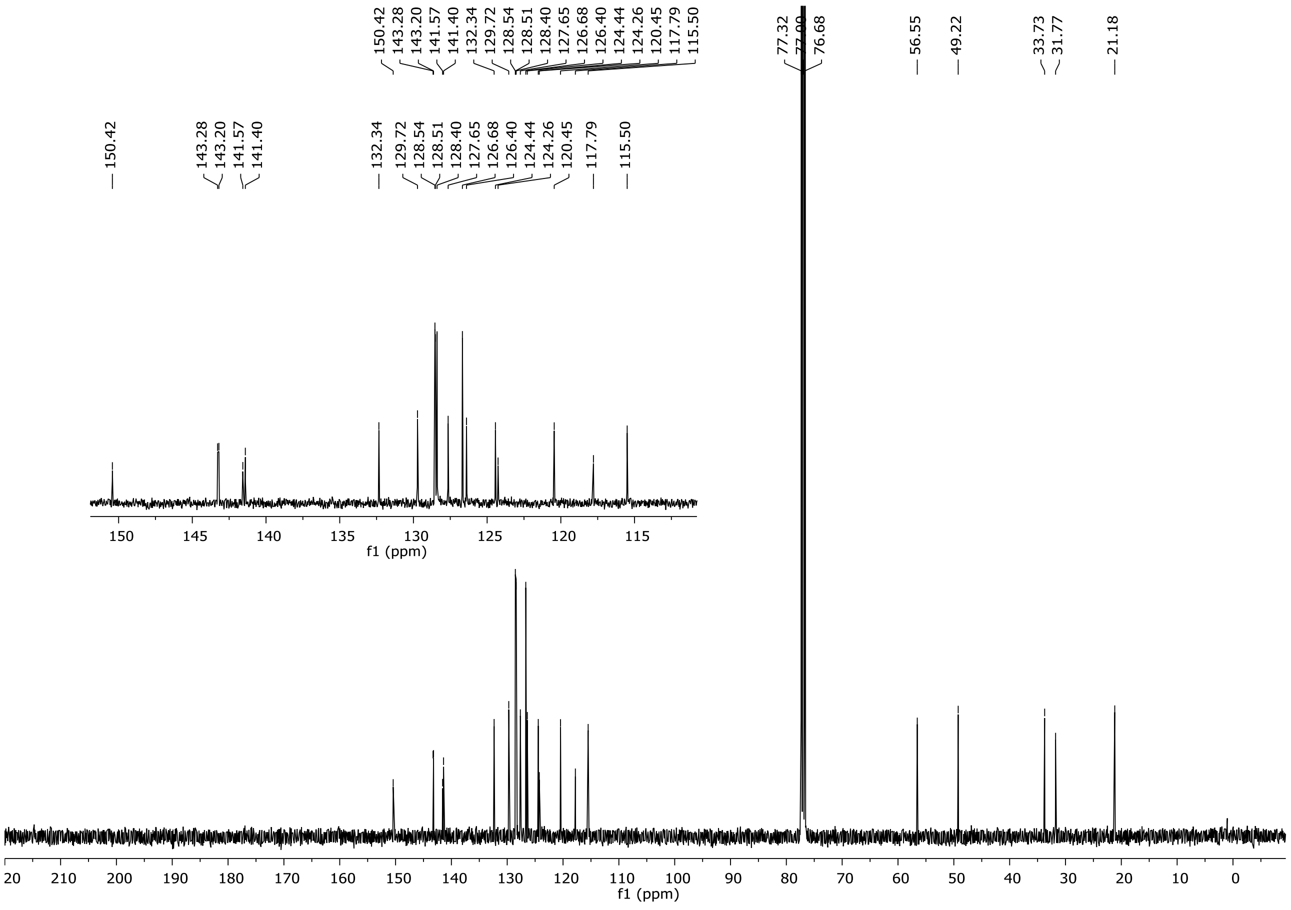
150.42
143.28
143.20
141.57
141.40
132.34
129.72
128.54
128.51
128.40
127.65
126.68
126.40
124.44
124.26
120.45
117.79
115.50
77.32
76.68
56.55
49.22
33.73
31.77
21.18
f1 (ppm)

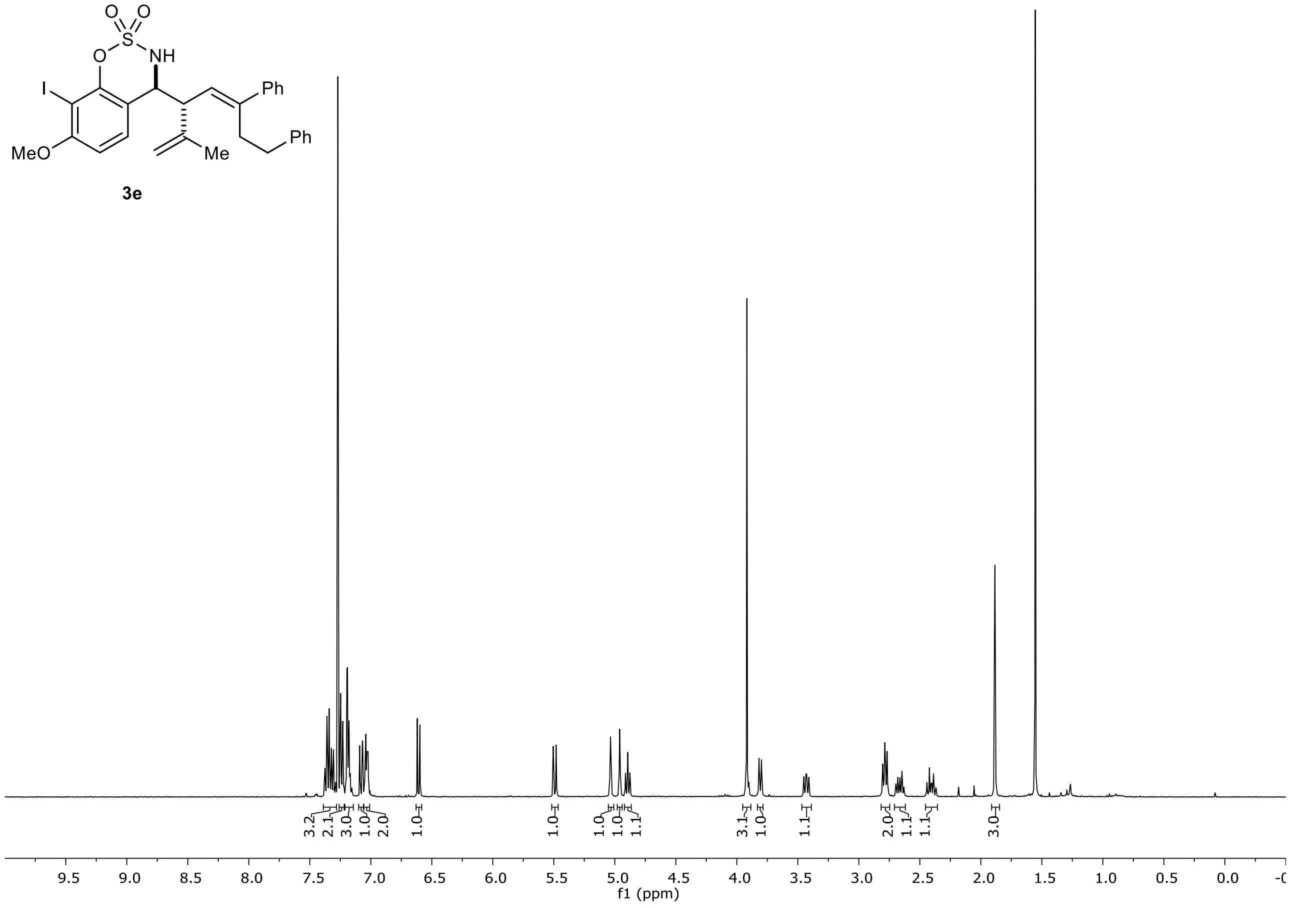
O
O
S
O
NH
I
Ph
MeO
Me
Ph
3e
3.2
2.1
3.0
1.0
2.0
1.0
1.0
1.0
1.0
1.1
3.1
1.0
1.1
2.0
1.1
1.1
3.0
f1 (ppm)

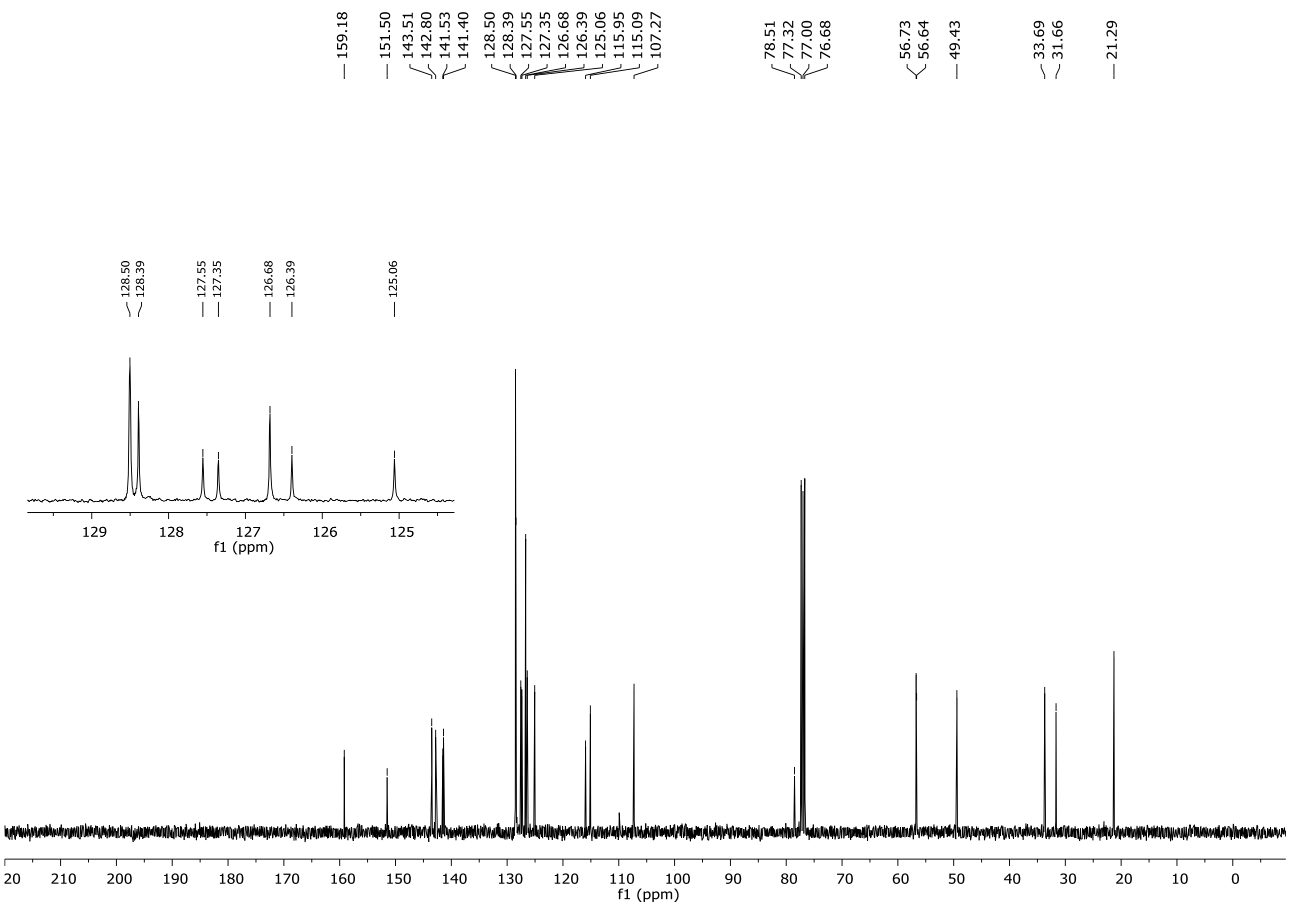
159.18
151.50
143.51
142.80
141.53
141.40
128.50
128.39
127.55
127.35
126.68
126.39
125.06
115.95
115.09
107.27
78.51
77.32
77.00
76.68
56.73
56.64
49.43
33.69
31.66
21.29
f1 (ppm)

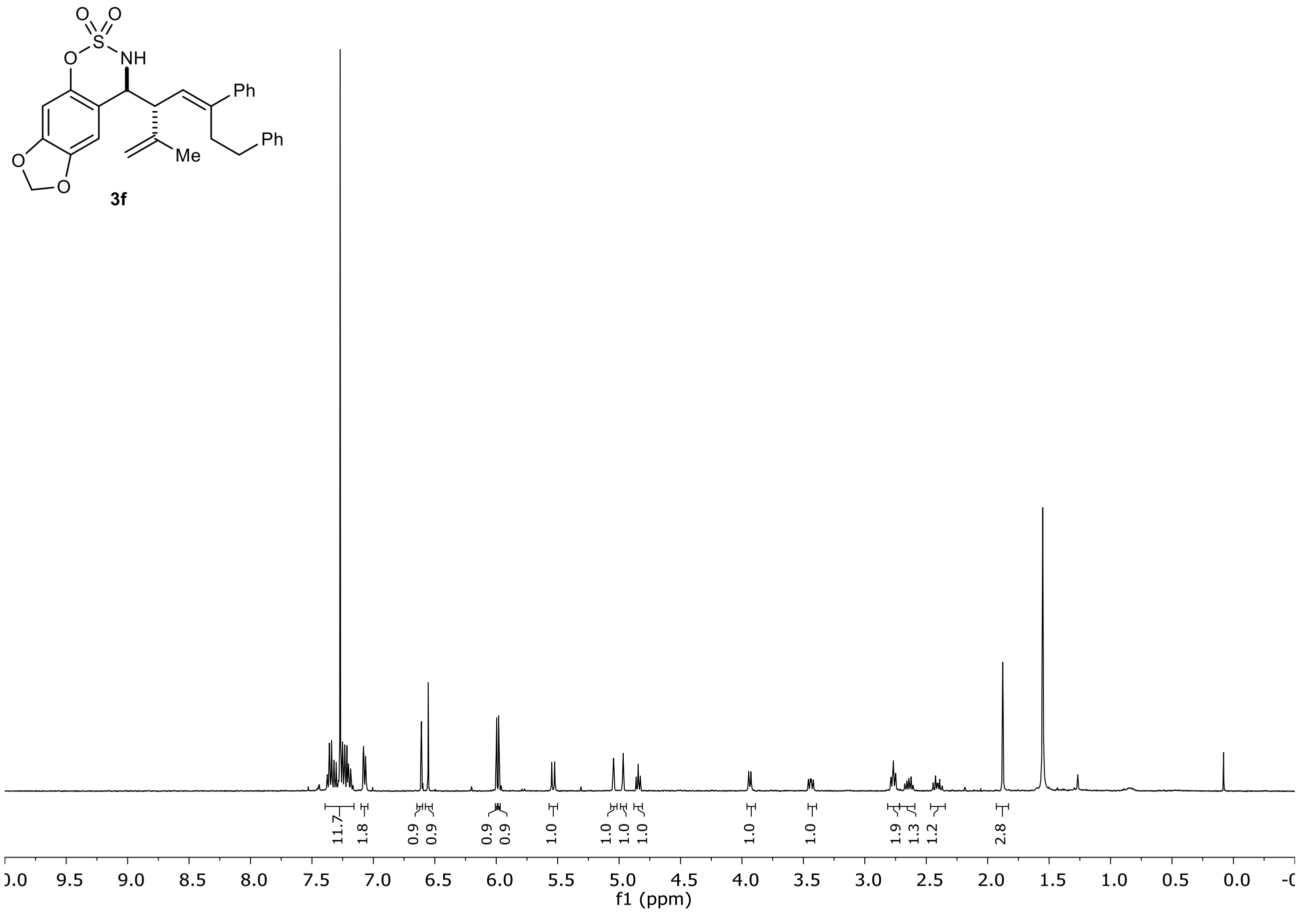
O
O
S
NH
O
Ph
Ph
Me
O
O
3f
11.7
1.8
0.9
0.9
0.9
0.9
1.0
1.0
1.0
1.0
1.0
1.0
1.9
1.3
1.2
2.8
f1 (ppm)

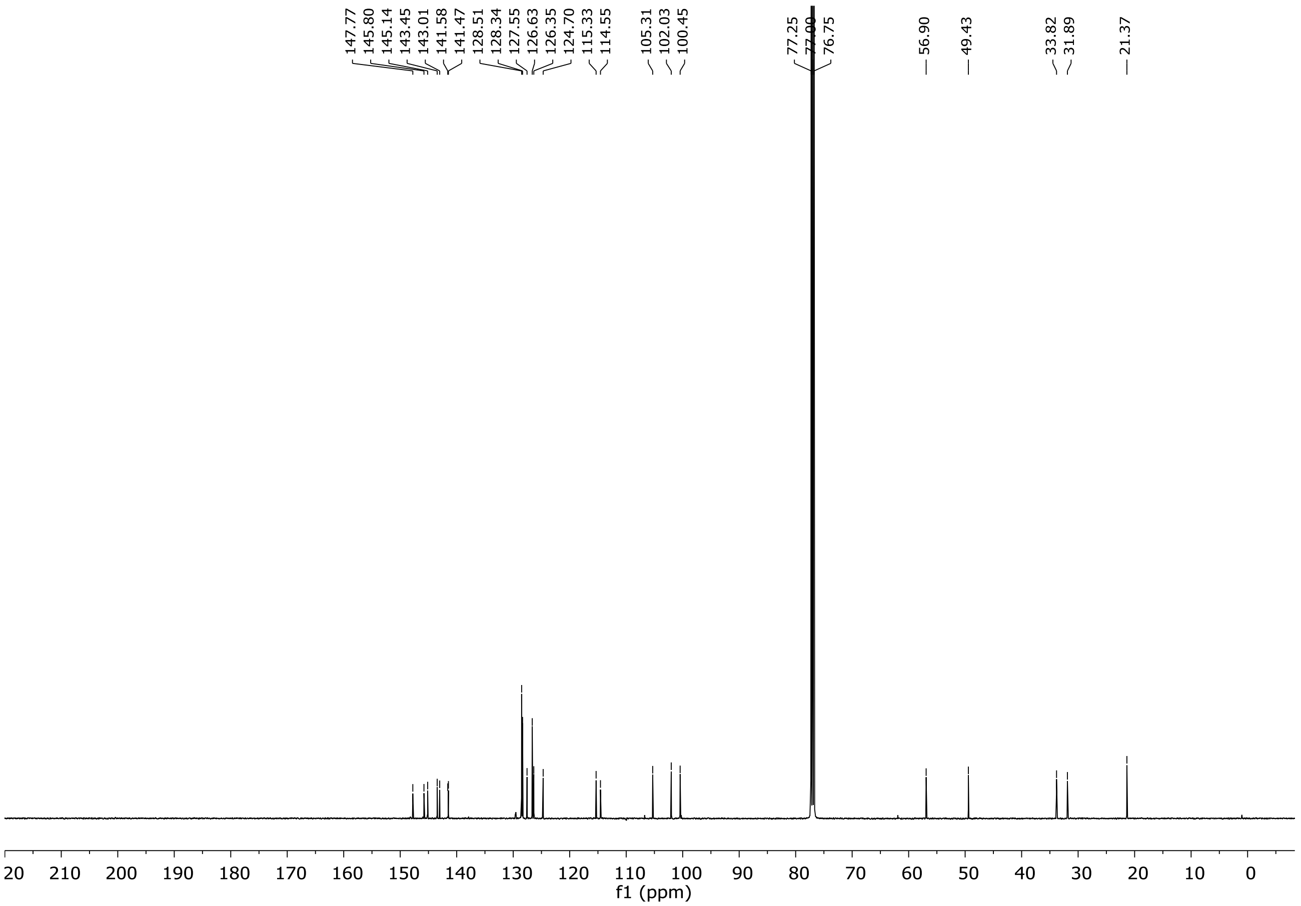
147.77
145.80
145.14
143.45
143.01
141.58
141.47
128.51
128.34
127.55
126.63
126.35
124.70
115.33
114.55
105.31
102.03
100.45
77.25
77.00
76.75
56.90
49.43
33.82
31.89
21.37
f1 (ppm)

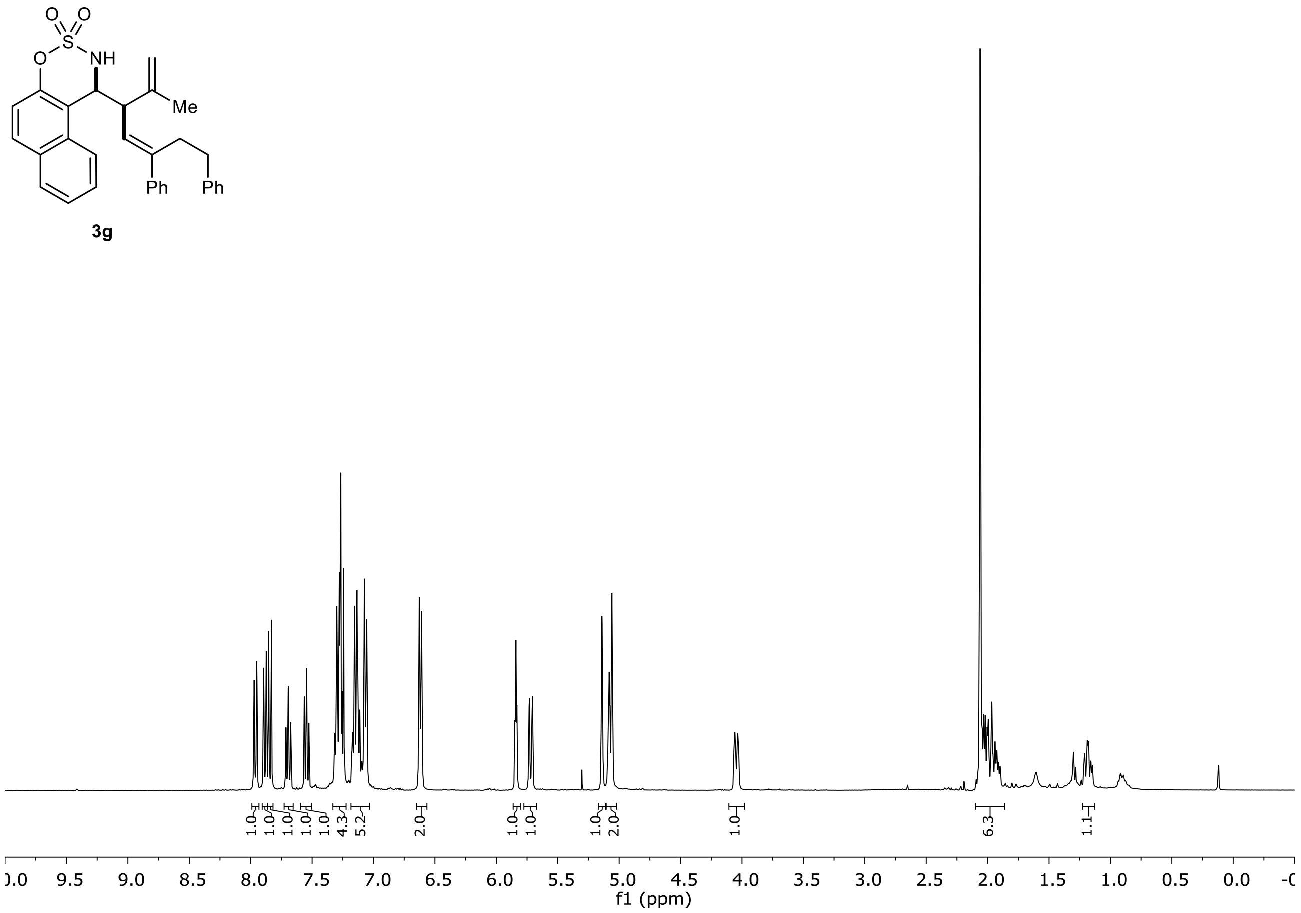
O
O
S
O
NH
Me
Ph
Ph

3g

1.0
1.0
1.0
1.0
1.0
4.3
5.2
2.0
1.0
1.0
1.0
2.0
1.0
6.3
1.1
f1 (ppm)

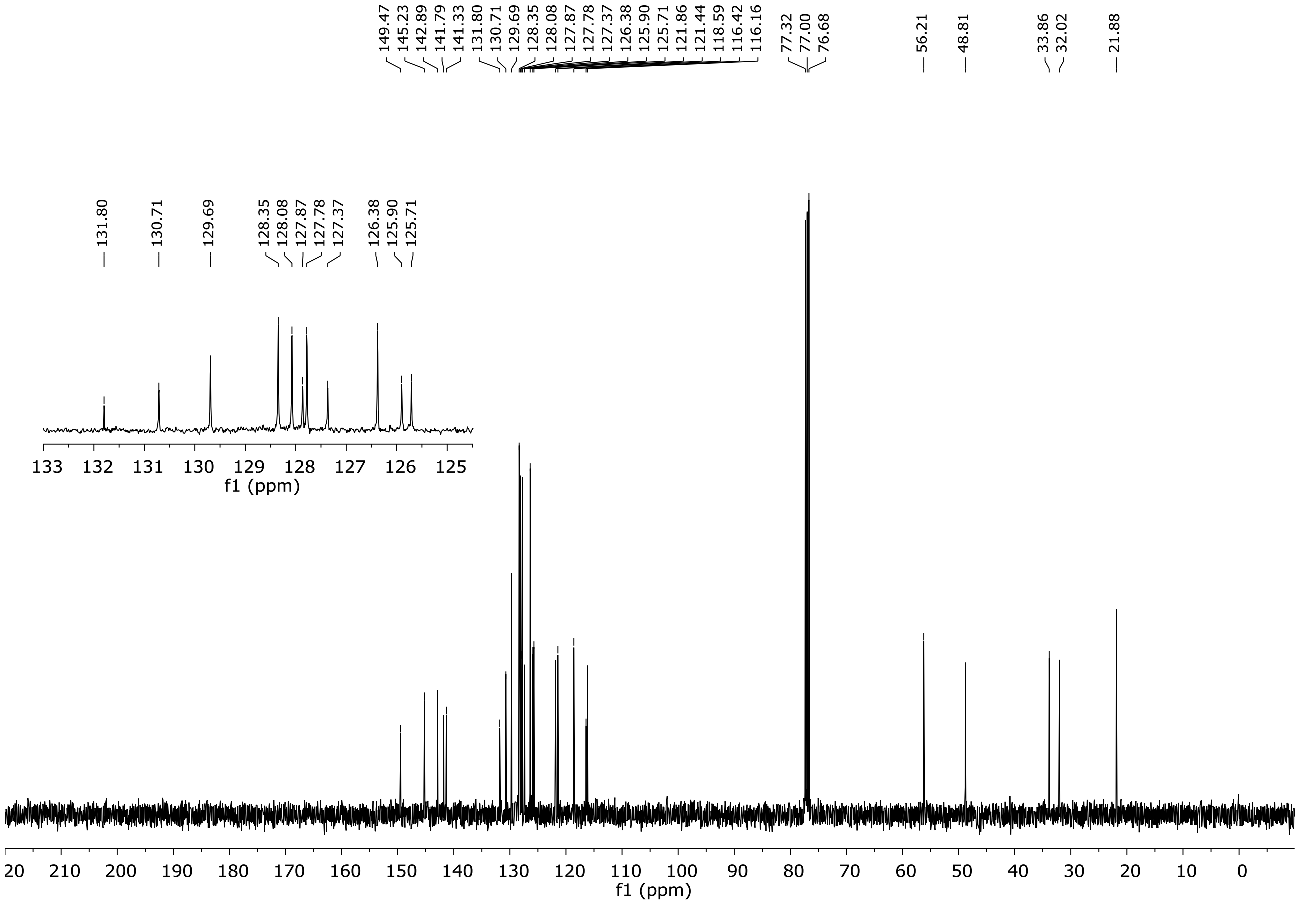
149.47
145.23
142.89
141.79
141.33
131.80
130.71
129.69
128.35
128.08
127.87
127.78
127.37
126.38
125.90
125.71
121.86
121.44
118.59
116.42
116.16
77.32
77.00
76.68
56.21
48.81
33.86
32.02
21.88
f1 (ppm)

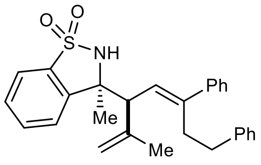
O
O
S
NH
Ph
Me
Me
Ph

6

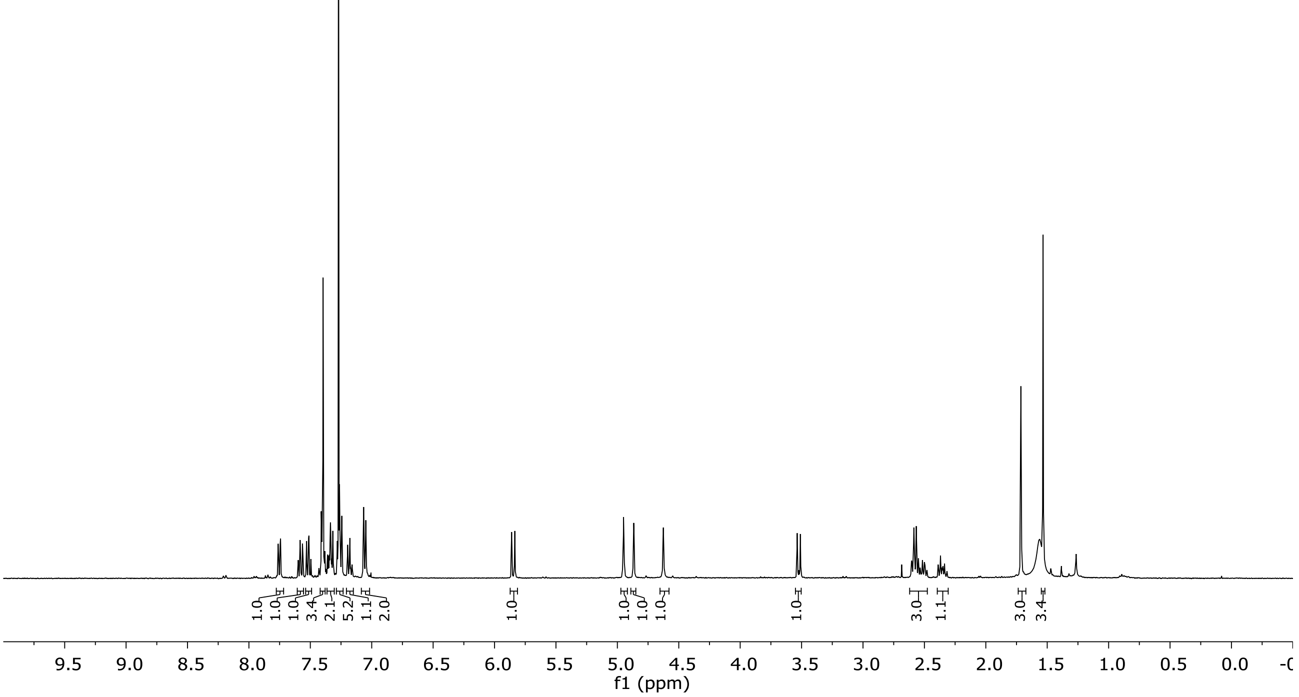
1.0
1.0
1.0
3.4
2.1
5.2
1.1
2.0
1.0
1.0
1.0
1.0
1.0
3.0
1.1
3.0
3.4
9.5 9.0 8.5 8.0 7.5 7.0 6.5 6.0 5.5 5.0 4.5 4.0 3.5 3.0 2.5 2.0 1.5 1.0 0.5 0.0
f1 (ppm)

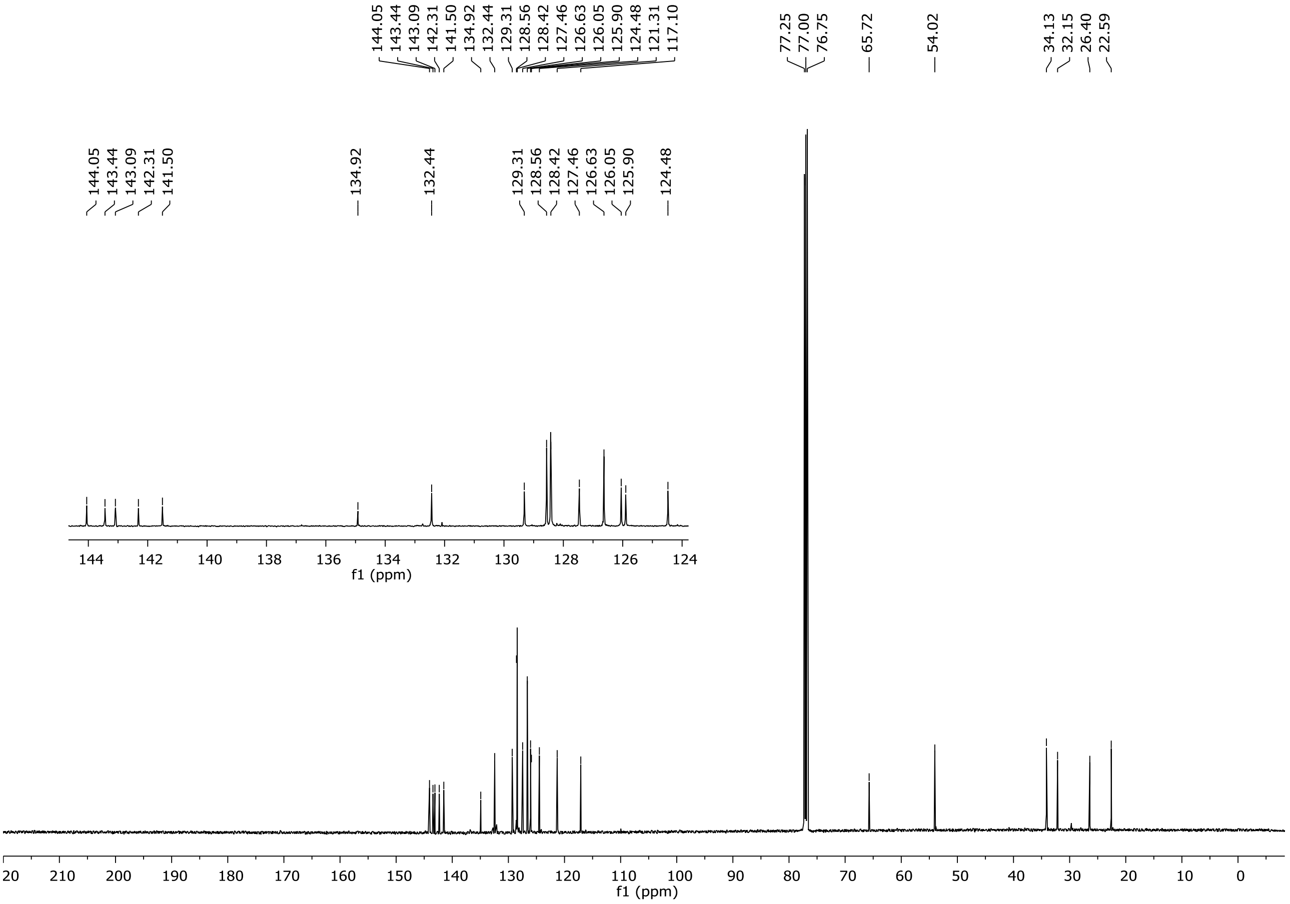
144.05
143.44
143.09
142.31
141.50
134.92
132.44
129.31
128.56
128.42
127.46
126.63
126.05
125.90
124.48
121.31
117.10
77.25
77.00
76.75
65.72
54.02
34.13
32.15
26.40
22.59
144 142 140 138 136 134 132 130 128 126 124
f1 (ppm)
210 200 190 180 170 160 150 140 130 120 110 100 90 80 70 60 50 40 30 20 10 0
f1 (ppm)

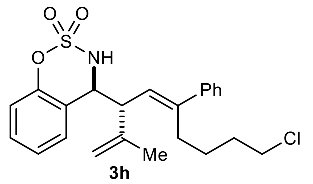
O O
S
O NH
Ph
Me
Cl
3h

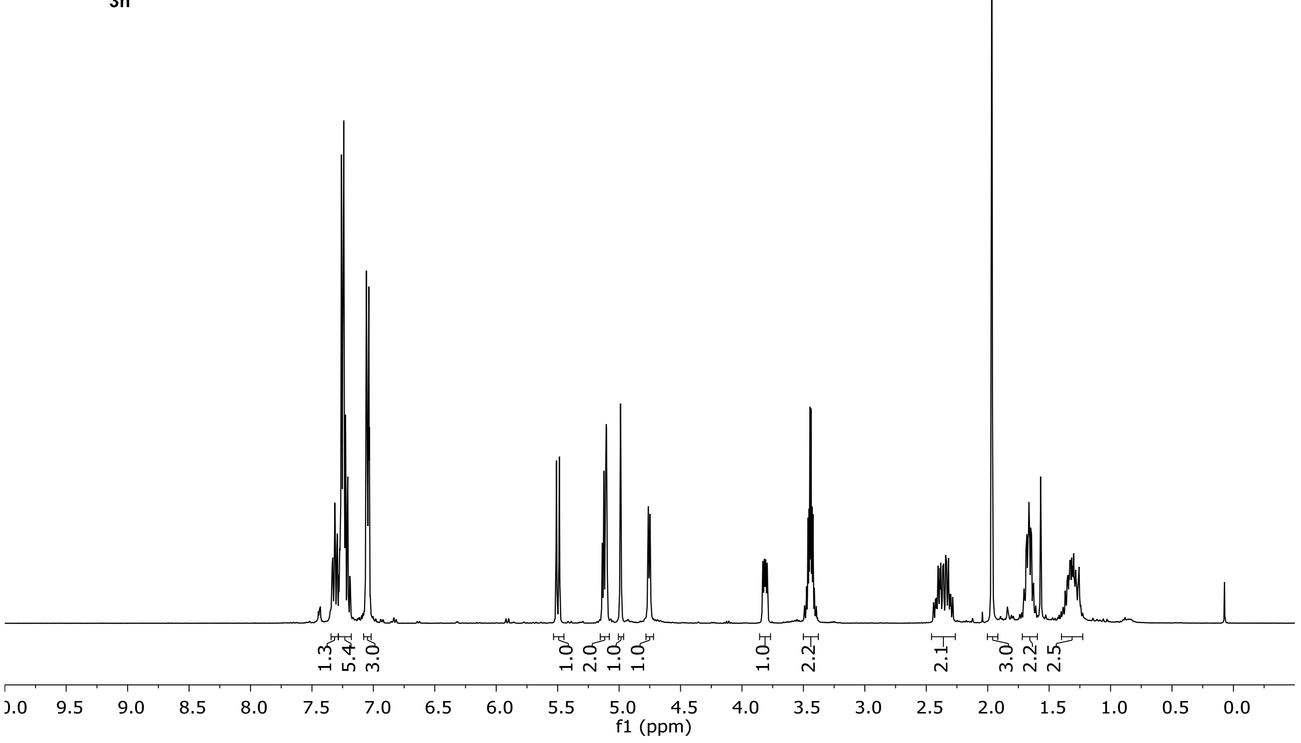
1.3
5.4
3.0
1.0
2.0
1.0
1.0
1.0
2.2
2.1
3.0
2.2
2.5
f1 (ppm)

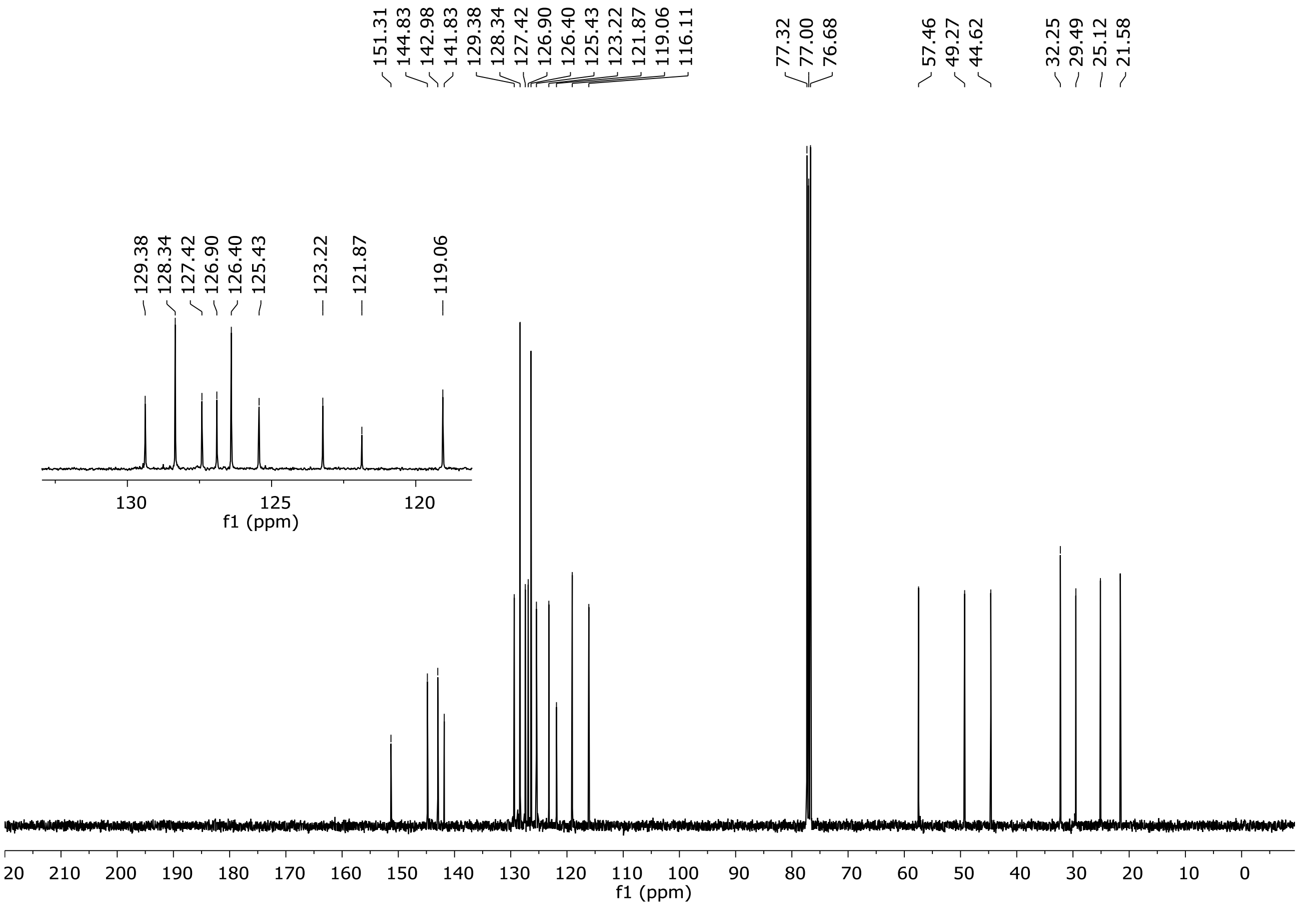
151.31
144.83
142.98
141.83
129.38
128.34
127.42
126.90
126.40
125.43
123.22
121.87
119.06
116.11
77.32
77.00
76.68
57.46
49.27
44.62
32.25
29.49
25.12
21.58
f1 (ppm)

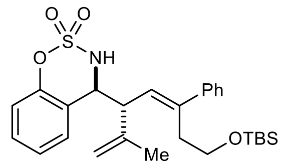
O
O
S
O
NH
Ph
OTBS
Me

3i

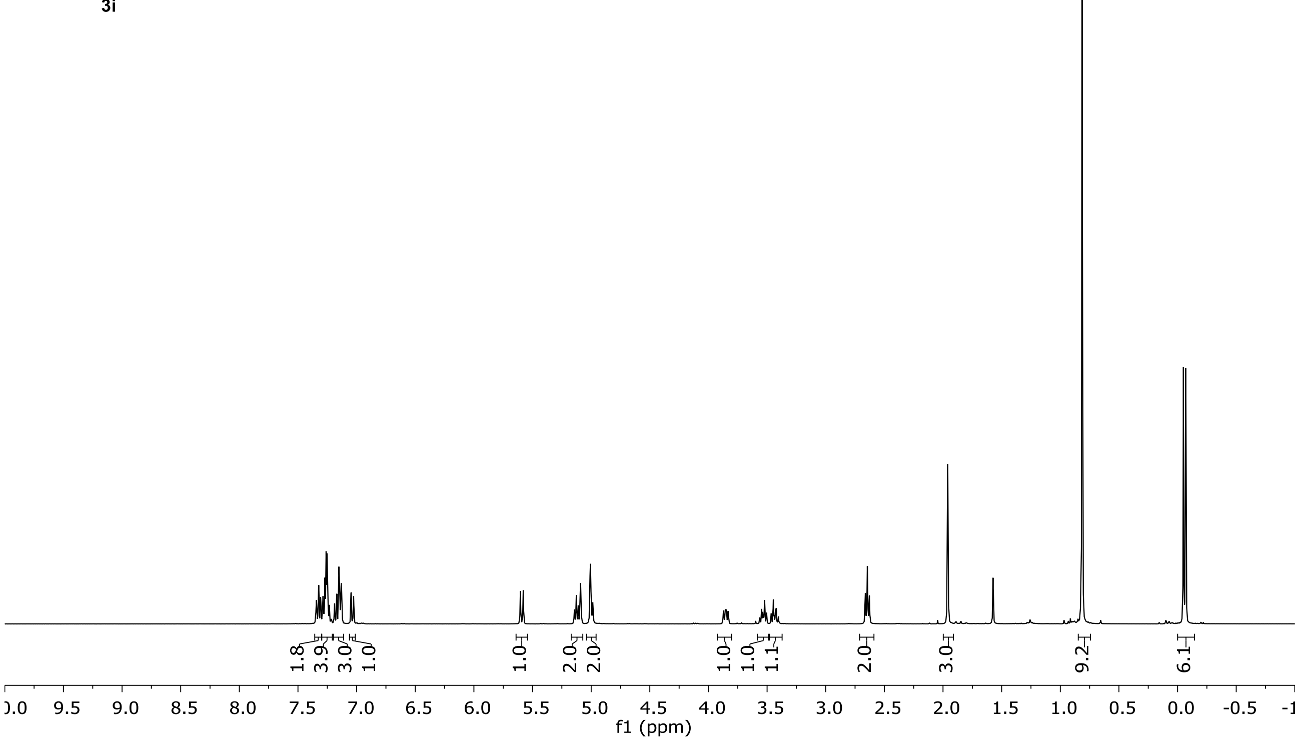
1.8
3.9
3.0
1.0
1.0
2.0
2.0
1.0
1.0
1.1
2.0
3.0
9.2
6.1
f1 (ppm)

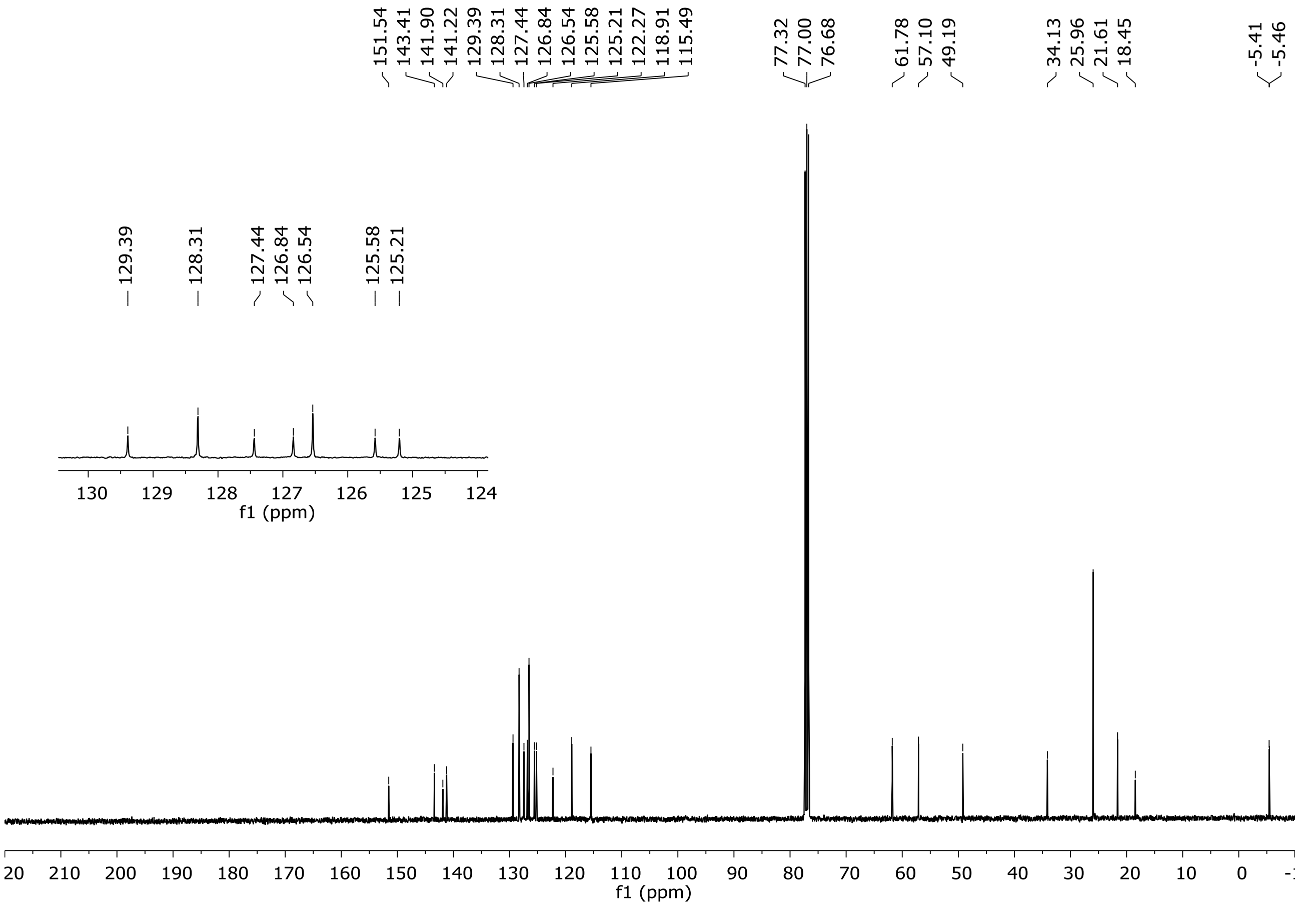
151.54
143.41
141.90
141.22
129.39
128.31
127.44
126.84
126.54
125.58
125.21
122.27
118.91
115.49
77.32
77.00
76.68
61.78
57.10
49.19
34.13
25.96
21.61
18.45
-5.41
-5.46
129.39
128.31
127.44
126.84
126.54
125.58
125.21
f1 (ppm)
f1 (ppm)

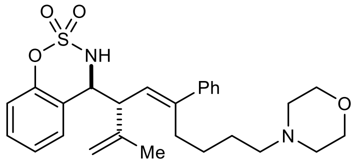

3j
8:1 mixture of diastereomers

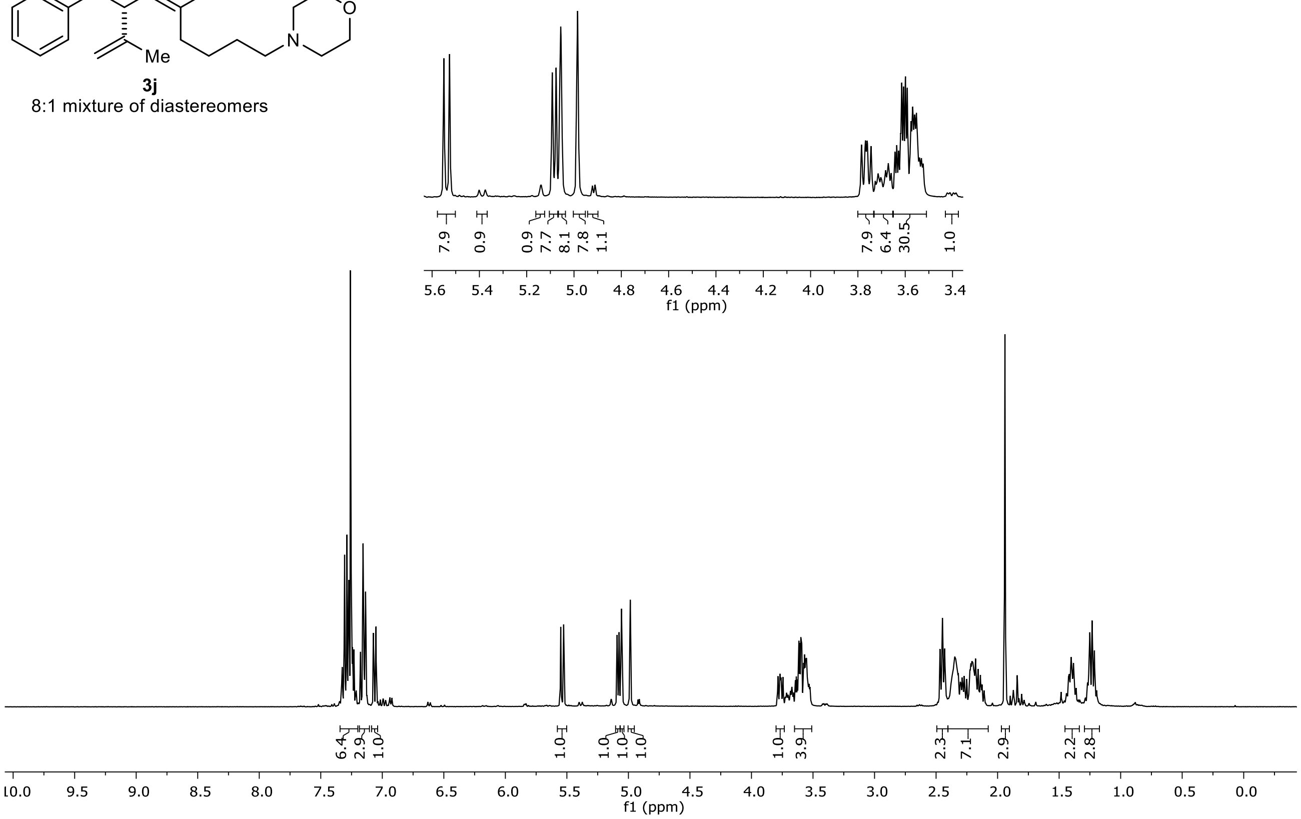

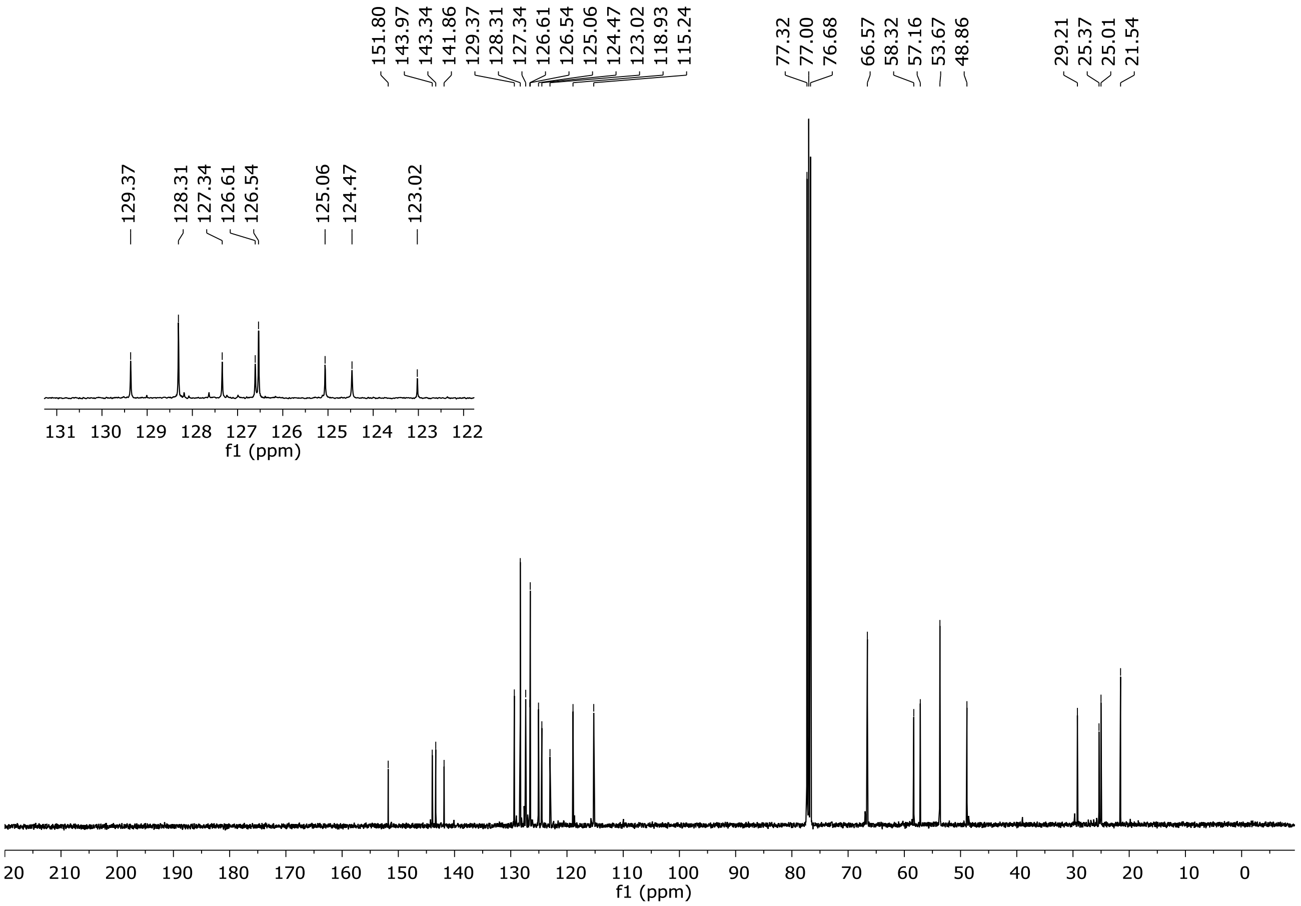

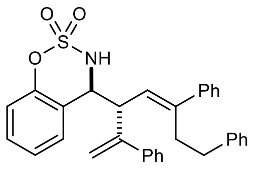

3k

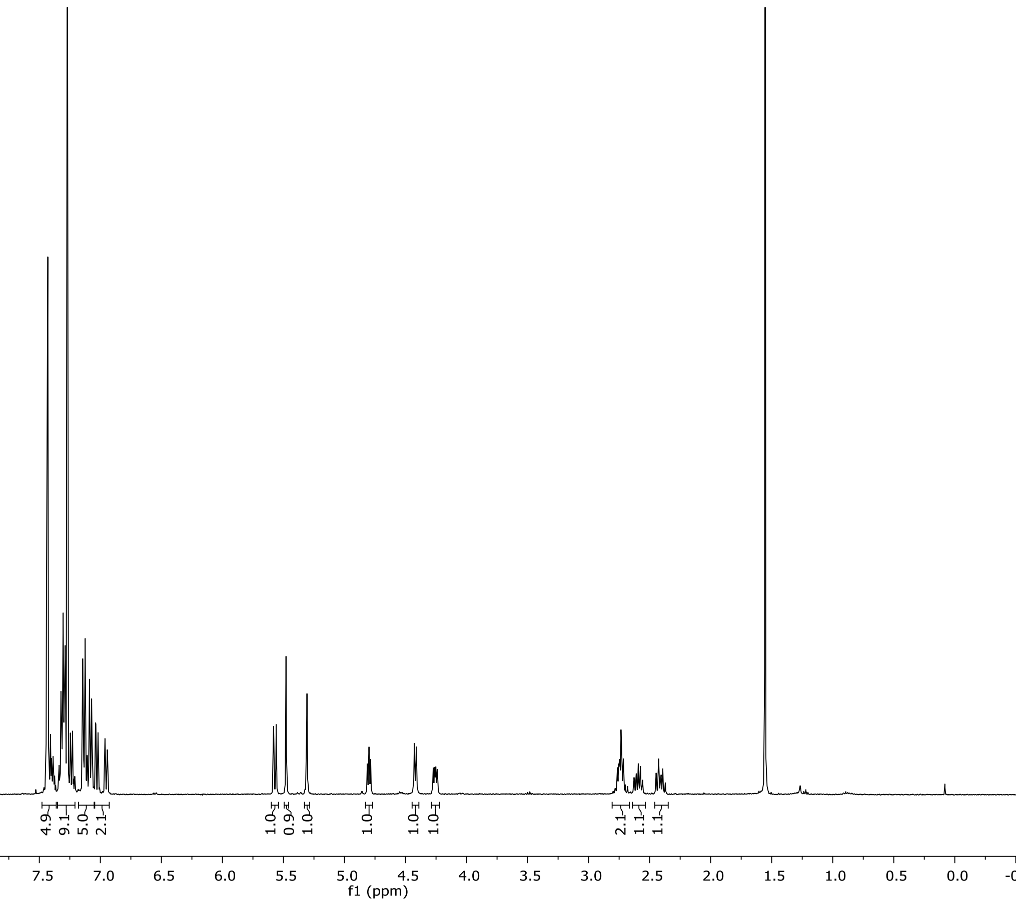

4.9
9.1
5.0
2.1
1.0
0.9
1.0
1.0
1.0
1.0
2.1
1.1
1.1
f1 (ppm)

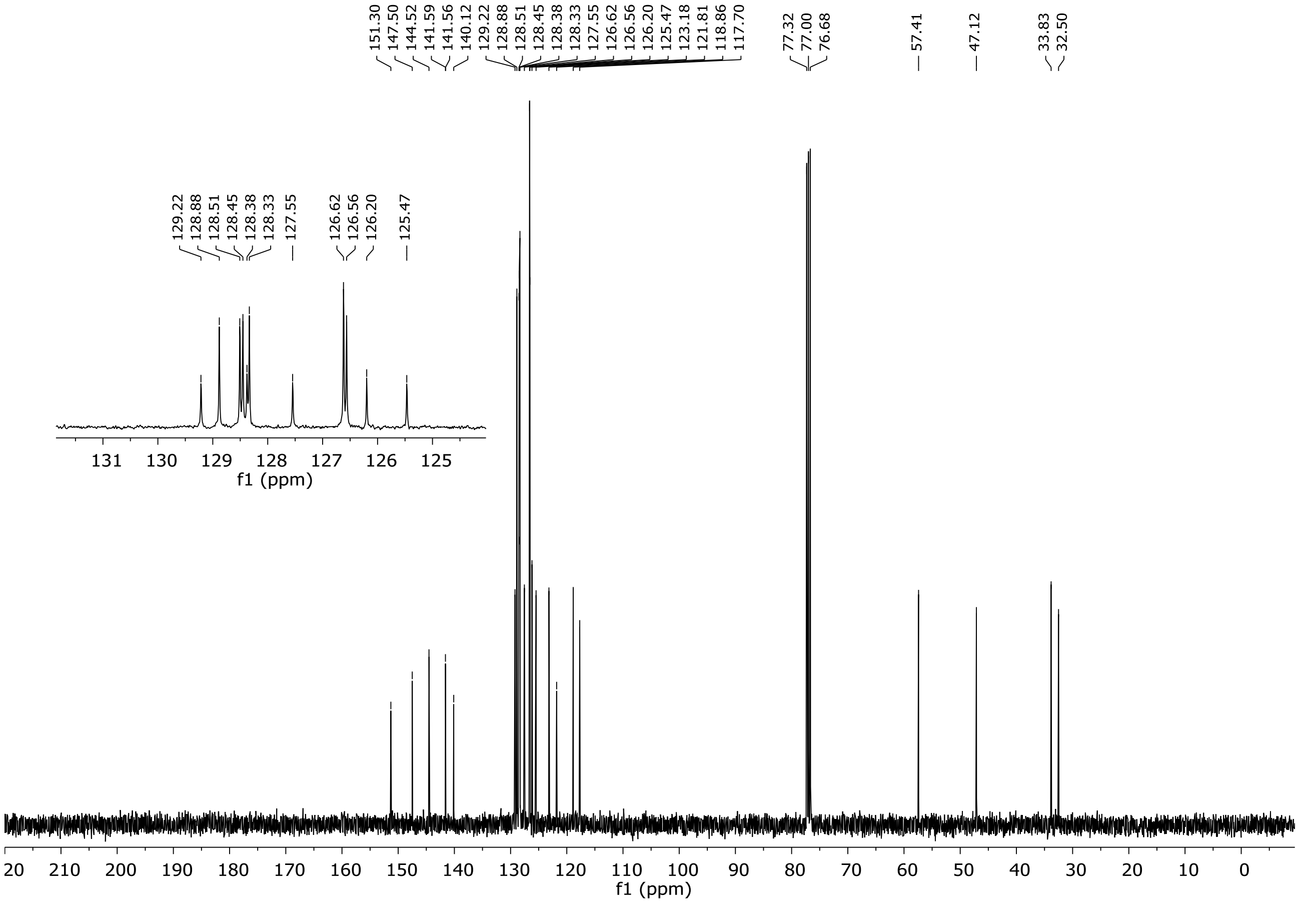

151.30
147.50
144.52
141.59
141.56
140.12
129.22
128.88
128.51
128.45
128.38
128.33
127.55
126.62
126.56
126.20
125.47
123.18
121.81
118.86
117.70
77.32
77.00
76.68
57.41
47.12
33.83
32.50
f1 (ppm)

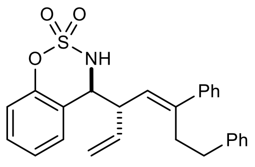

3l

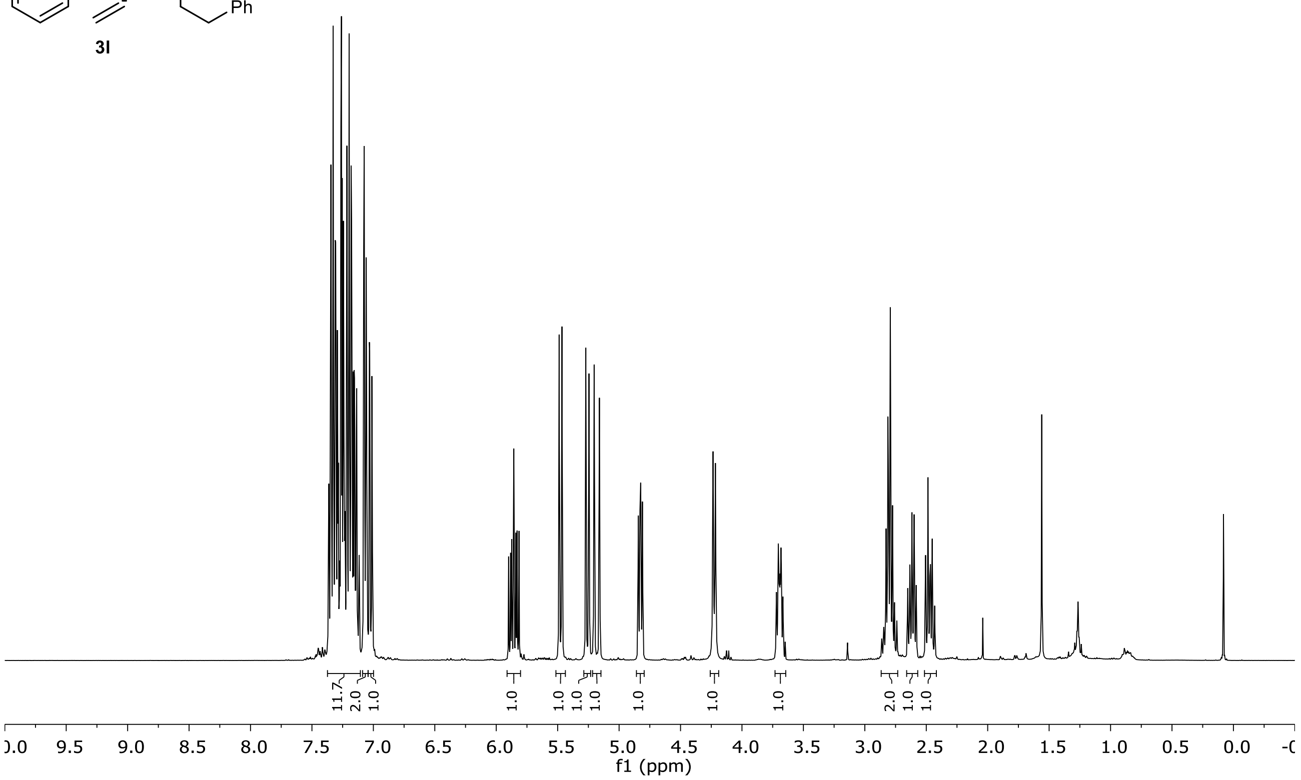
11.7
2.0
1.0
1.0
1.0
1.0
1.0
1.0
1.0
1.0
2.0
1.0
1.0
f1 (ppm)

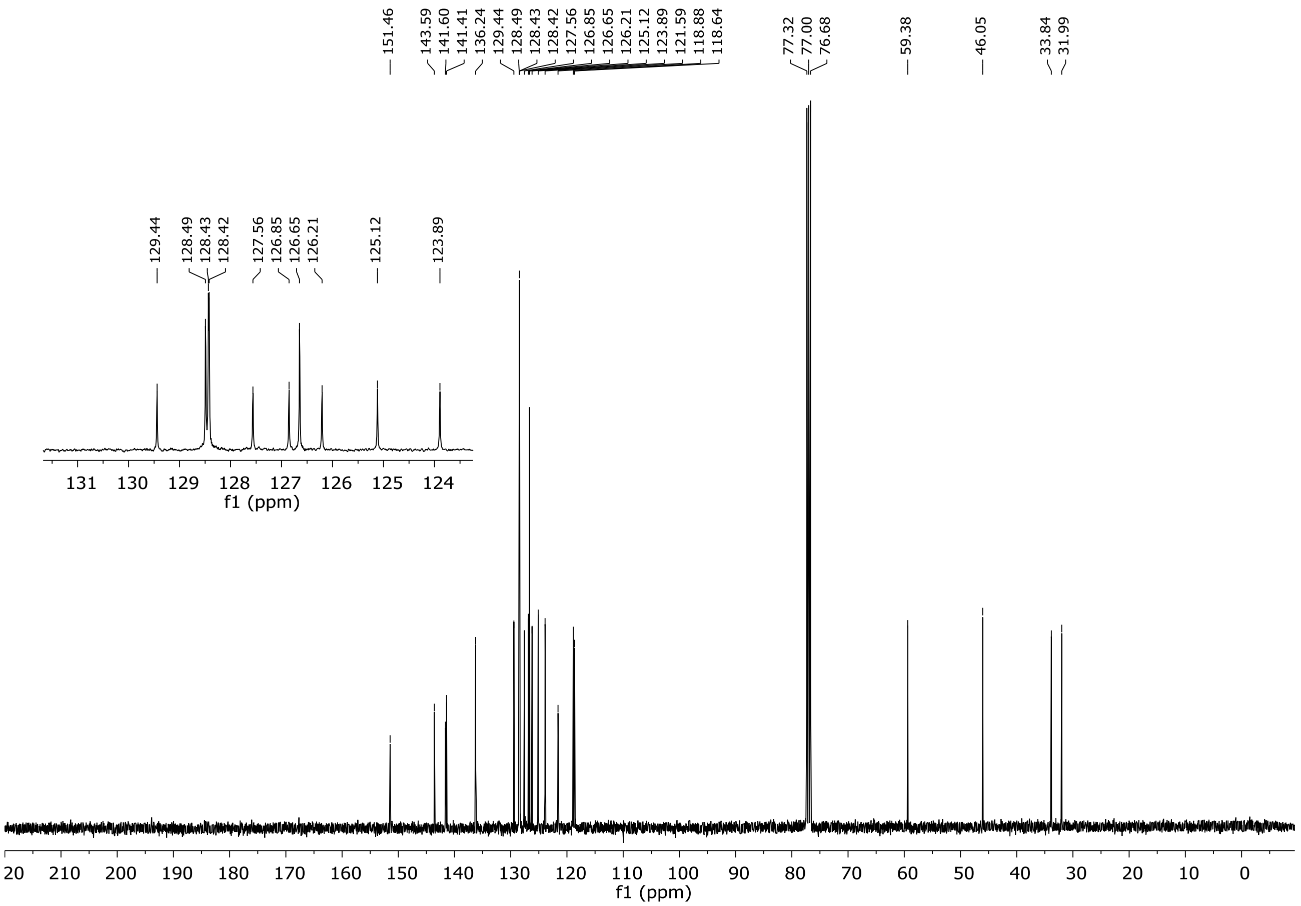
151.46
143.59
141.60
141.41
136.24
129.44
128.49
128.43
128.42
127.56
126.85
126.65
126.21
125.12
123.89
121.59
118.88
118.64
77.32
77.00
76.68
59.38
46.05
33.84
31.99
f1 (ppm)

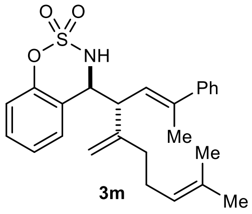
O
O
S
O
NH
Ph
Me
Me
Me

3m

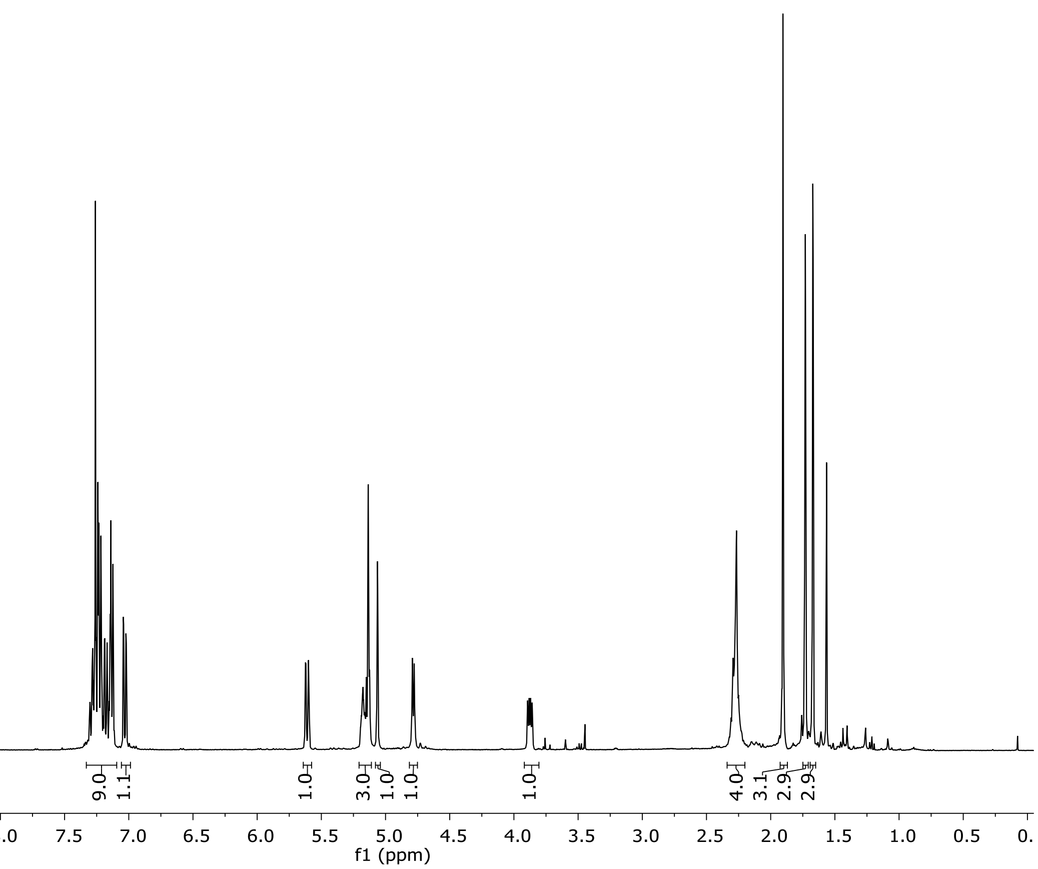
9.0
1.1
1.0
3.0
1.0
1.0
1.0
4.0
3.1
2.9
2.9
f1 (ppm)

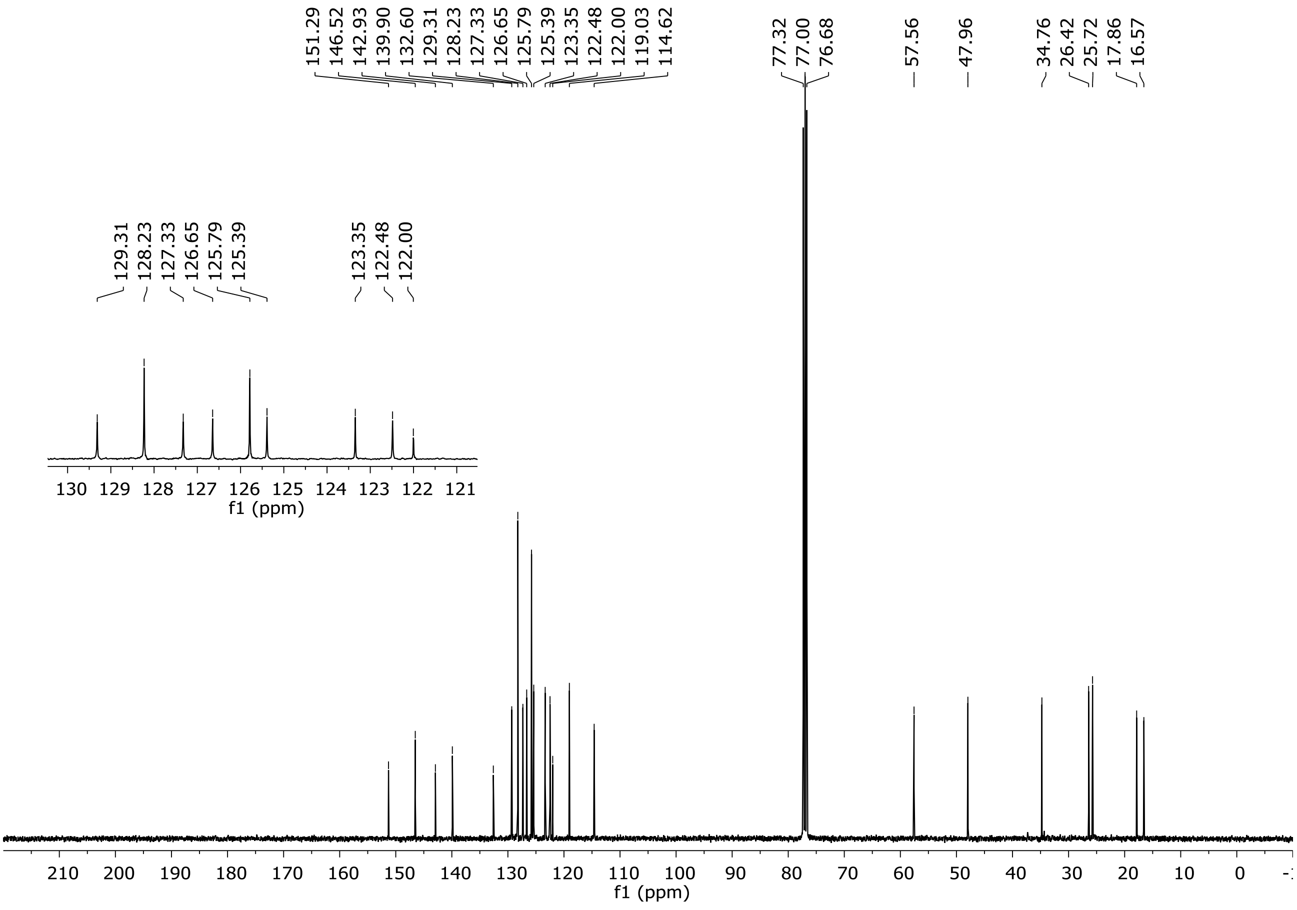
151.29
146.52
142.93
139.90
132.60
129.31
128.23
127.33
126.65
125.79
125.39
123.35
122.48
122.00
119.03
114.62
77.32
77.00
76.68
57.56
47.96
34.76
26.42
25.72
17.86
16.57
f1 (ppm)

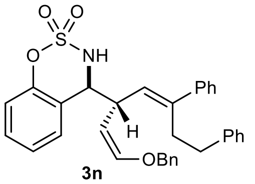

3n

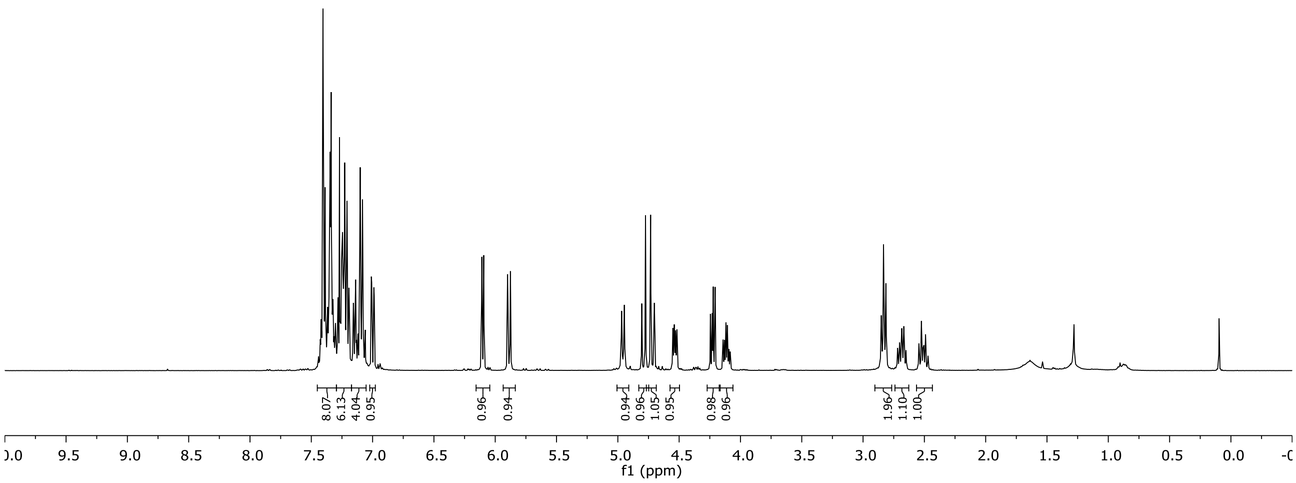

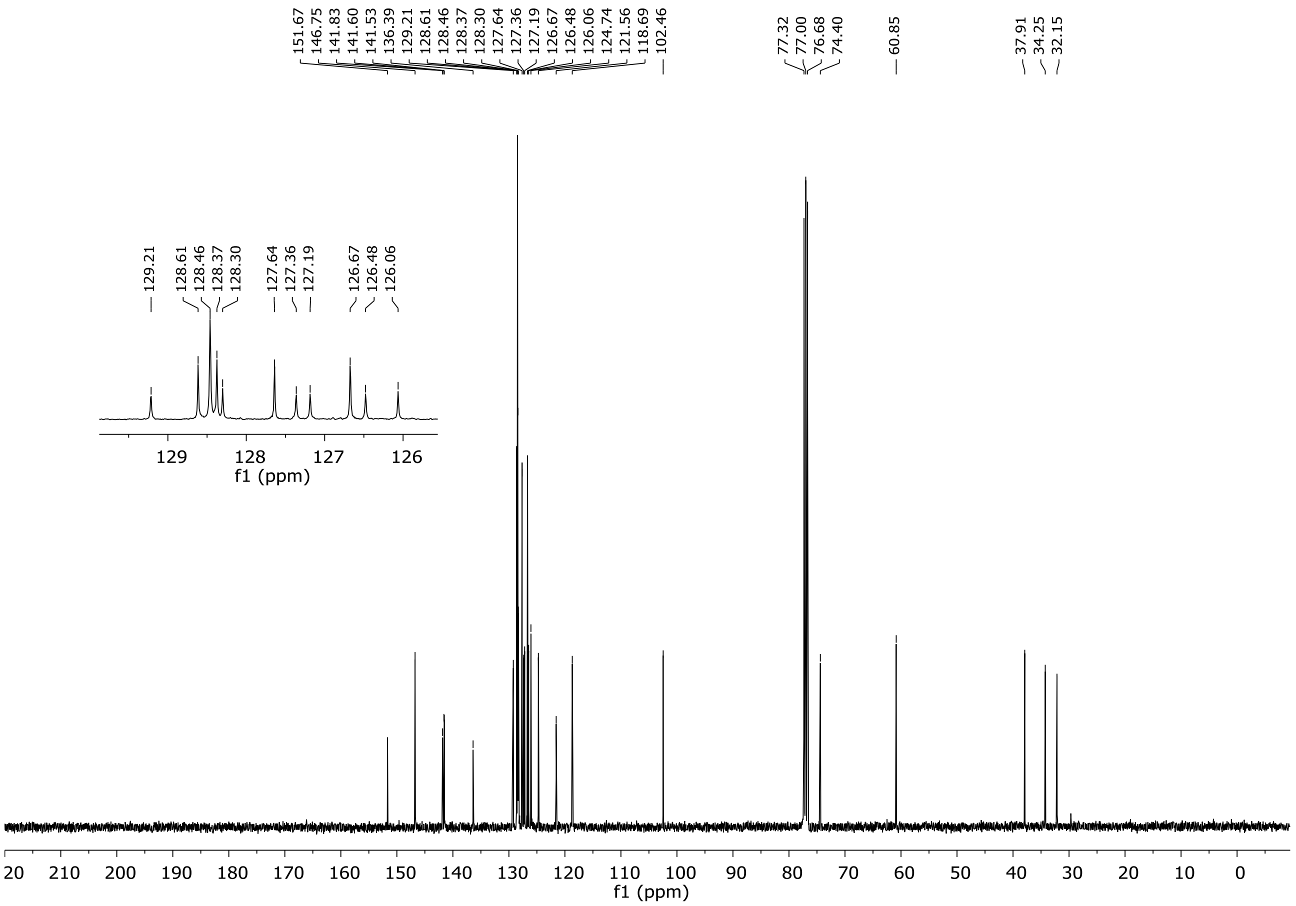

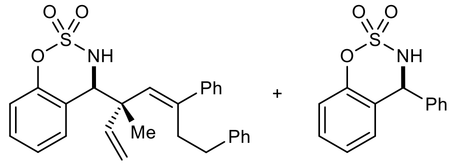

3o

(8.2:1 inseparable mixture)

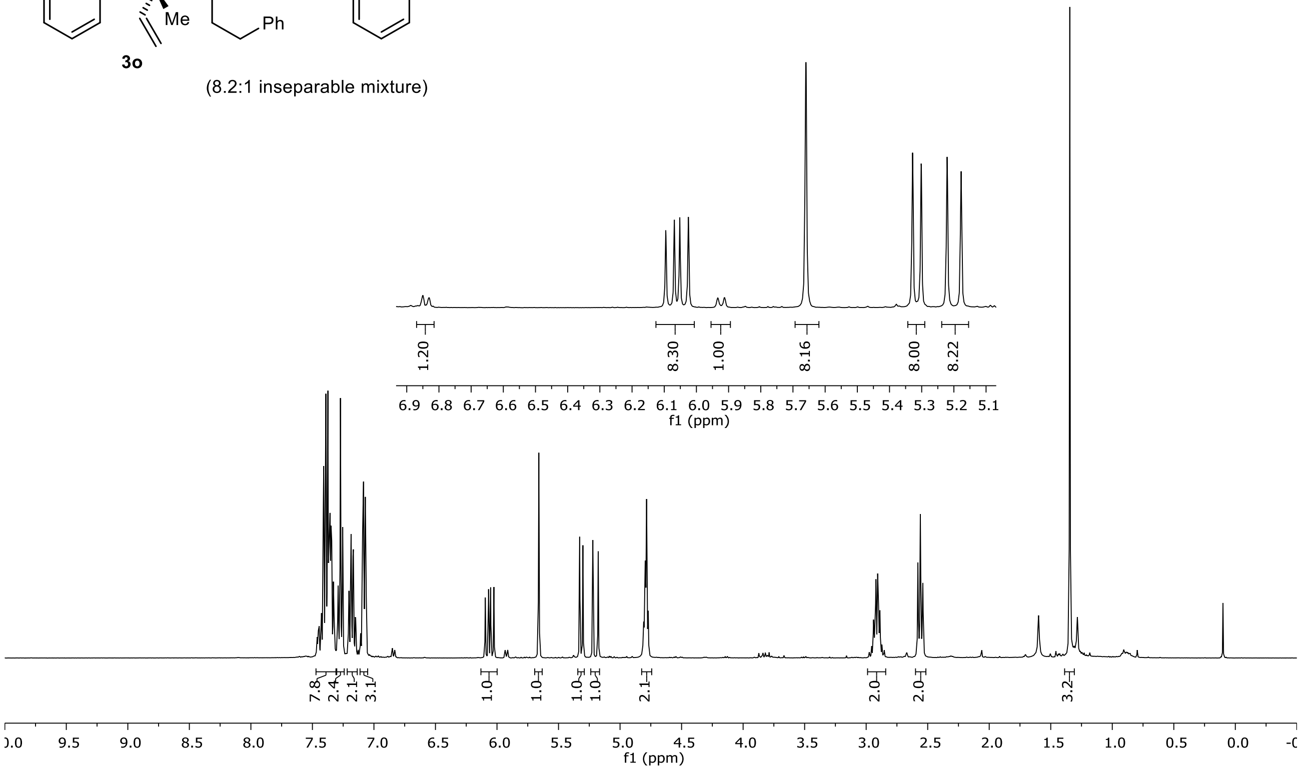

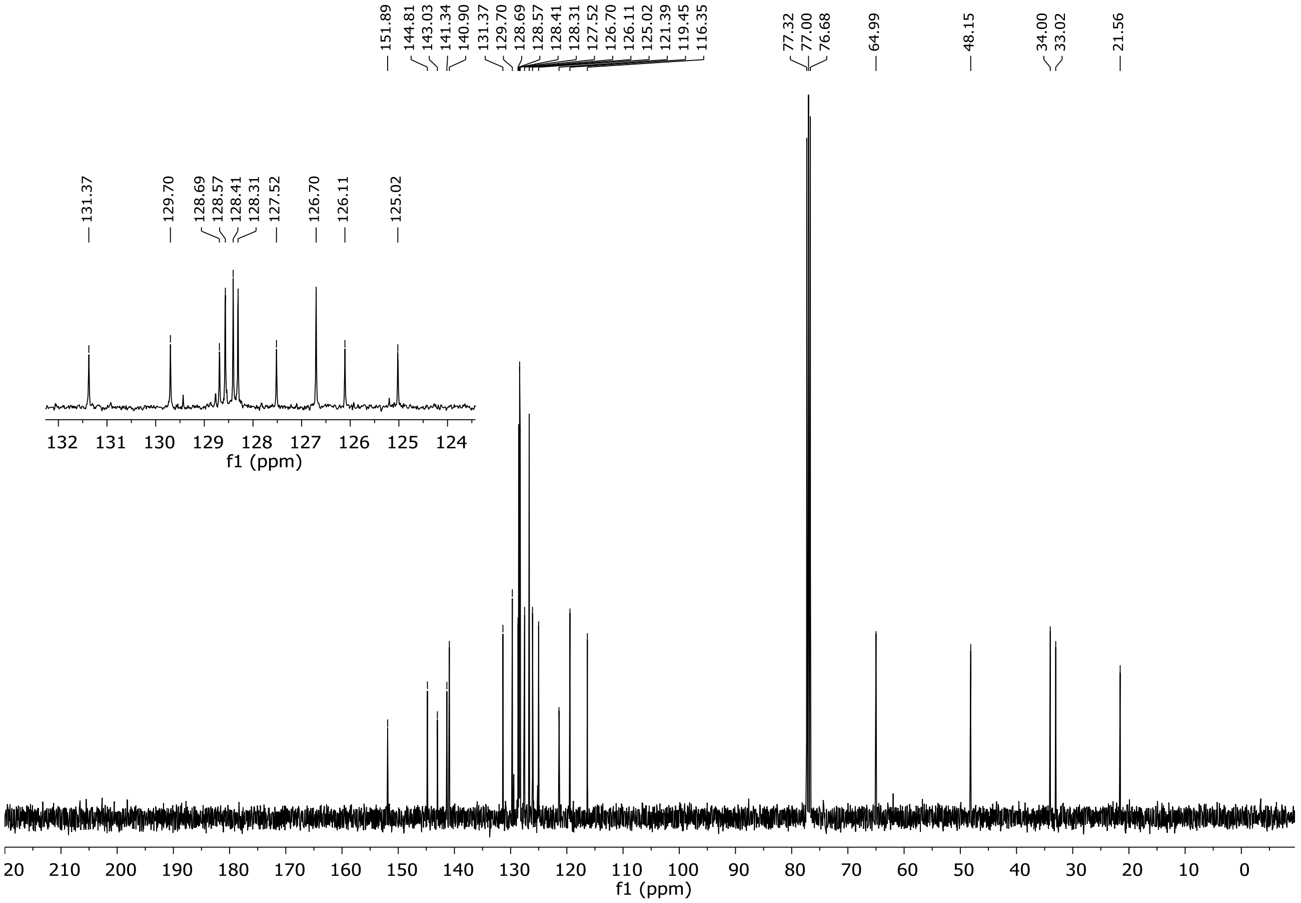

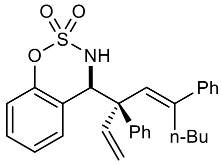
O
O
S
NH
O
Ph
Ph
n-Bu

3p

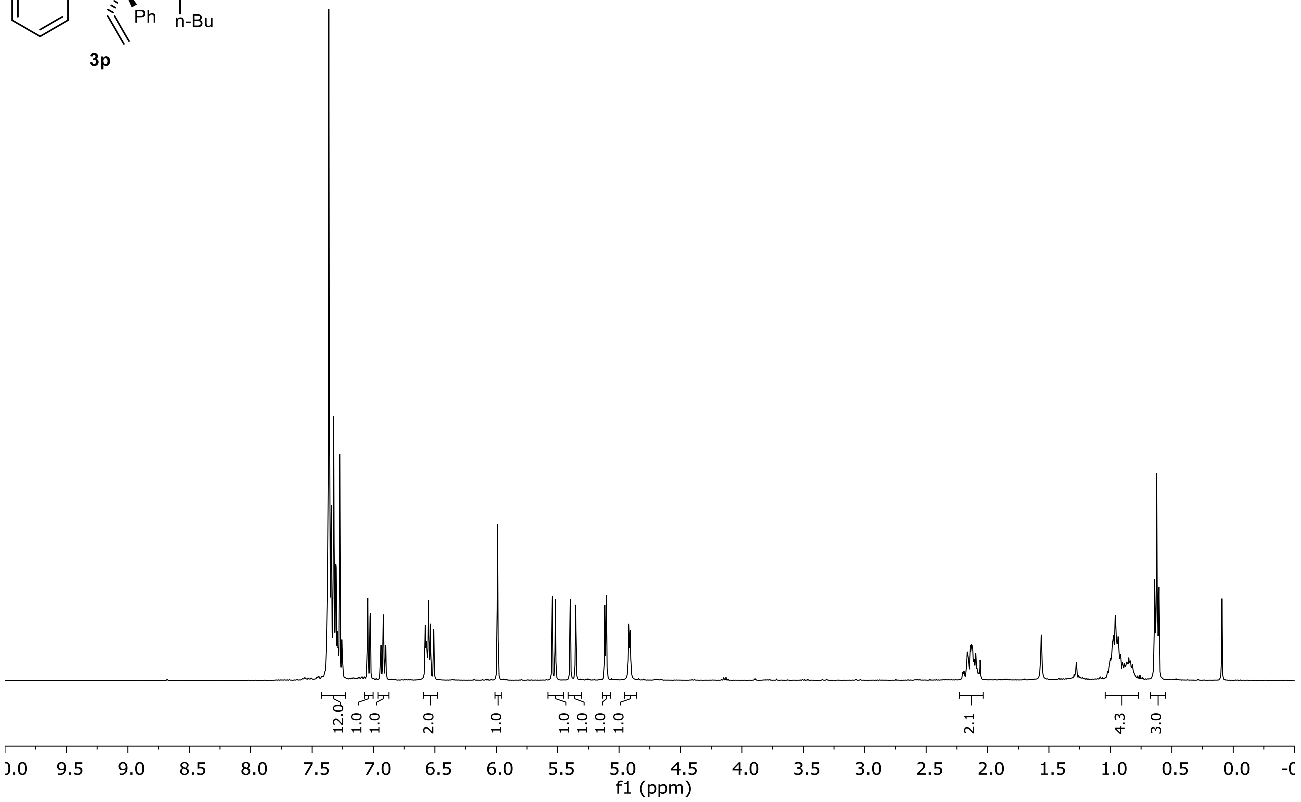
12.0
1.0
1.0
2.0
1.0
1.0
1.0
1.0
1.0
2.1
4.3
3.0
f1 (ppm)

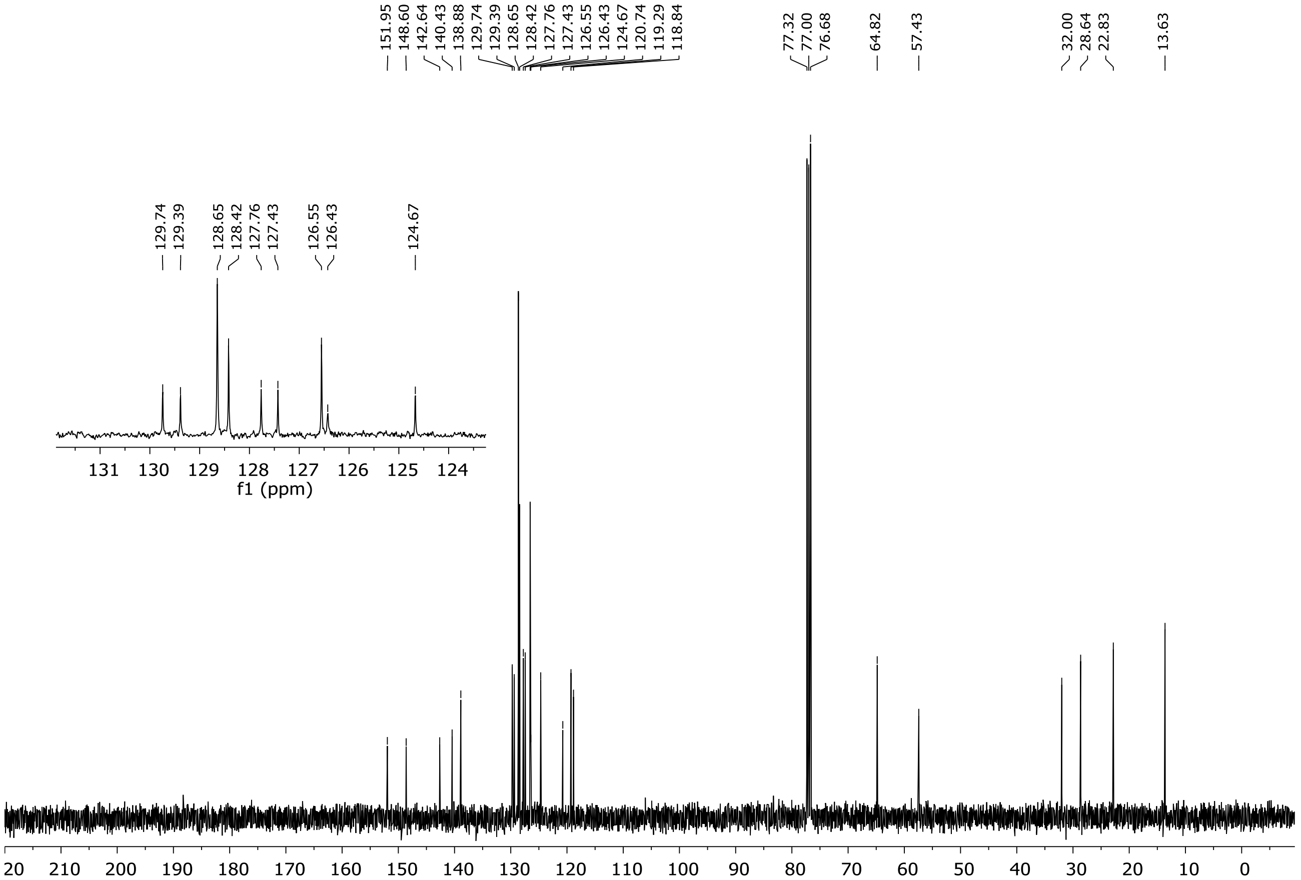
151.95
148.60
142.64
140.43
138.88
129.74
129.39
128.65
128.42
127.76
127.43
126.55
126.43
124.67
120.74
119.29
118.84
77.32
77.00
76.68
64.82
57.43
32.00
28.64
22.83
13.63
f1 (ppm)

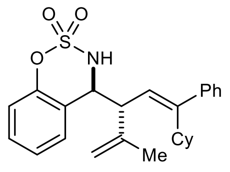

(*E*)-**3q**

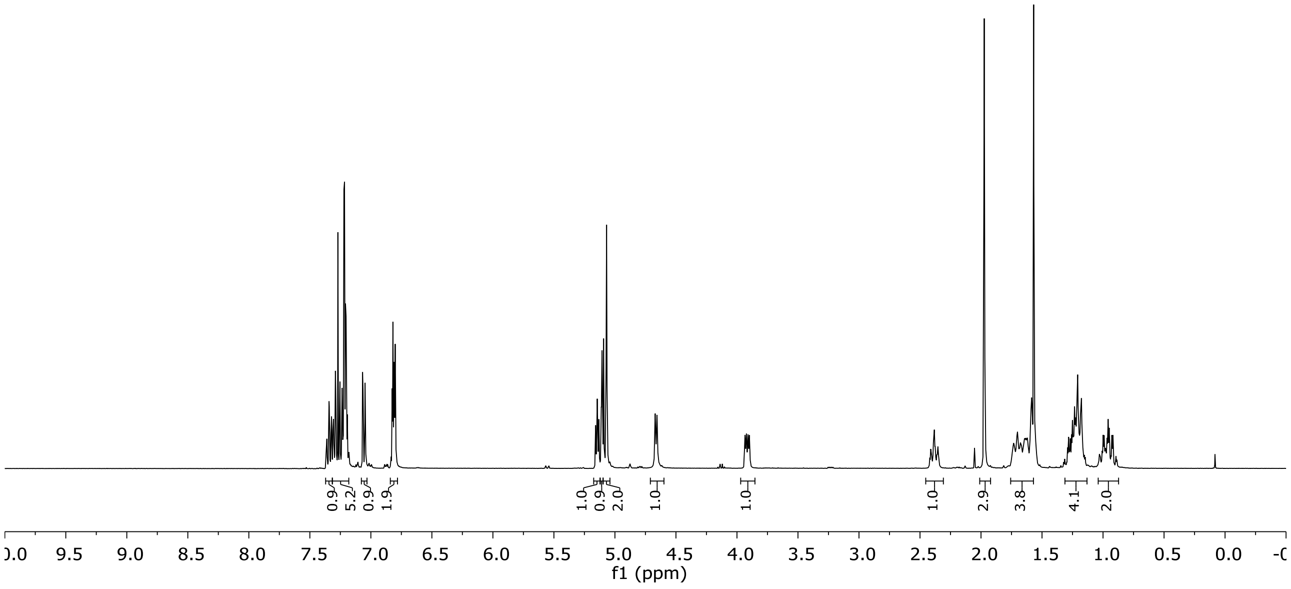

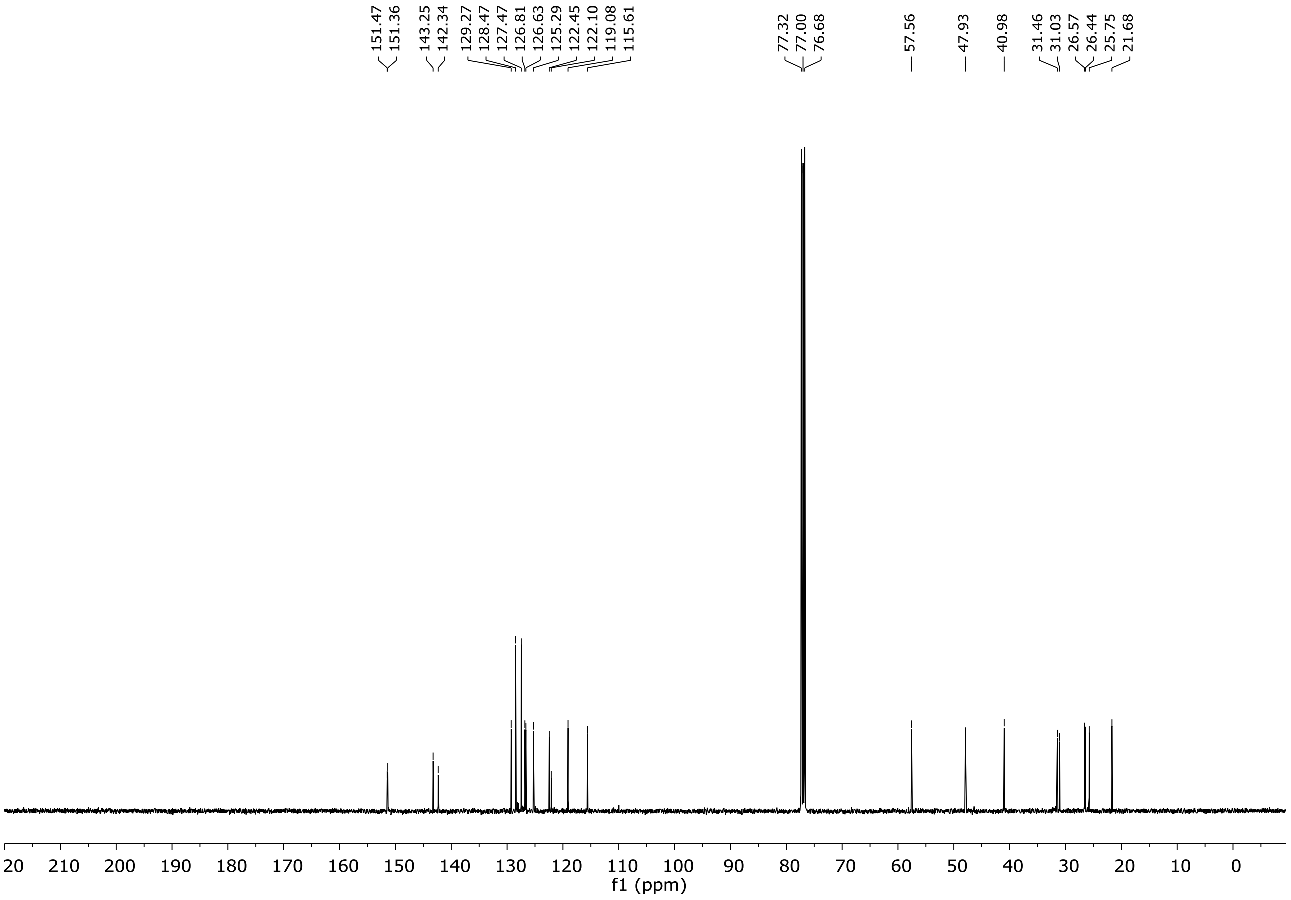

NOESY

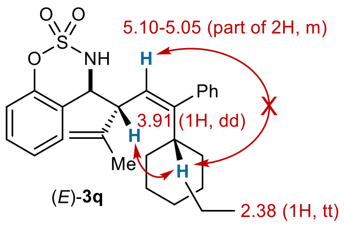

(*E*)-**3q**

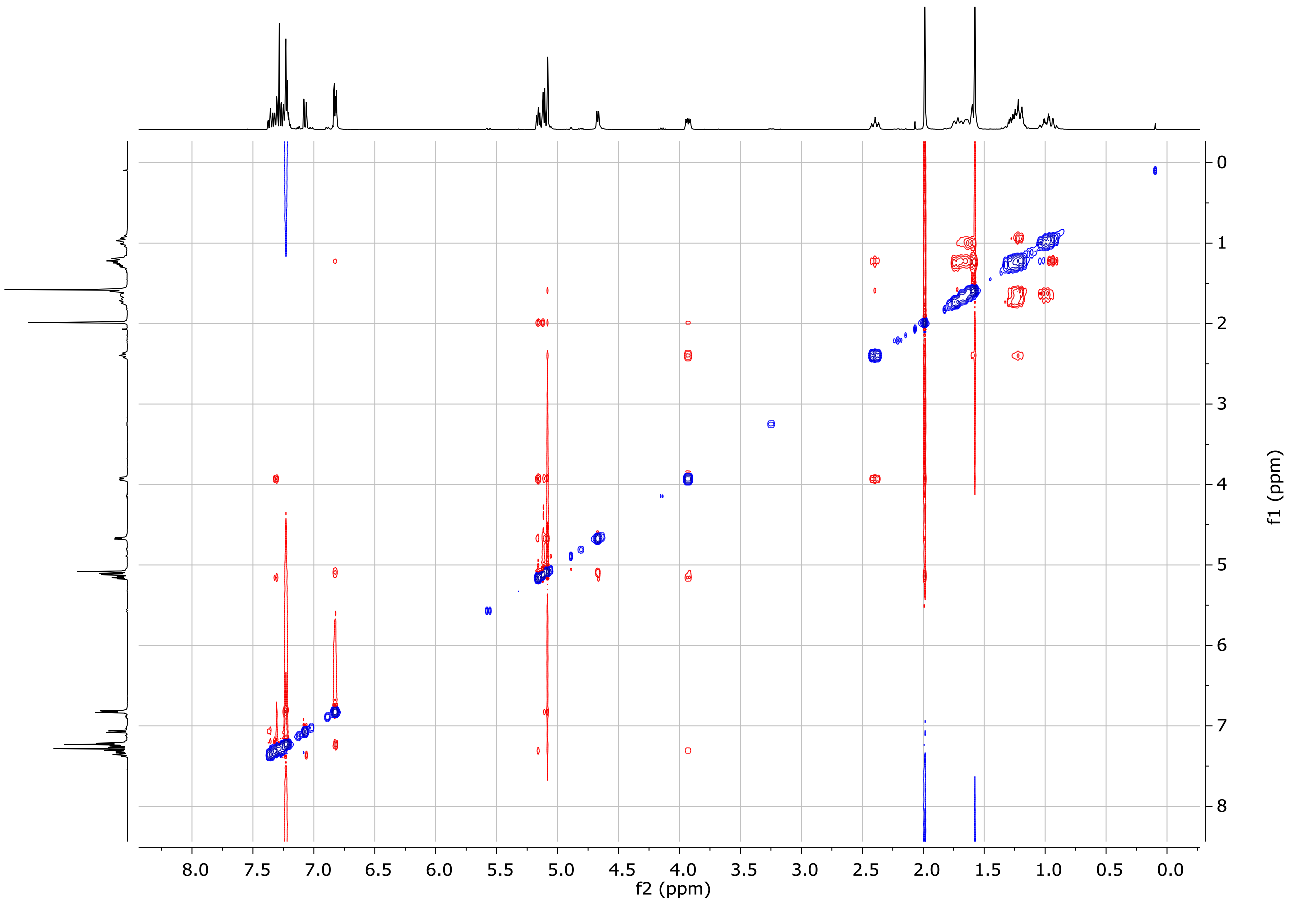

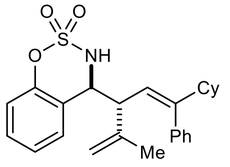
O
O
S
NH
O
Cy
Ph
Me

(*Z*)-**3q**

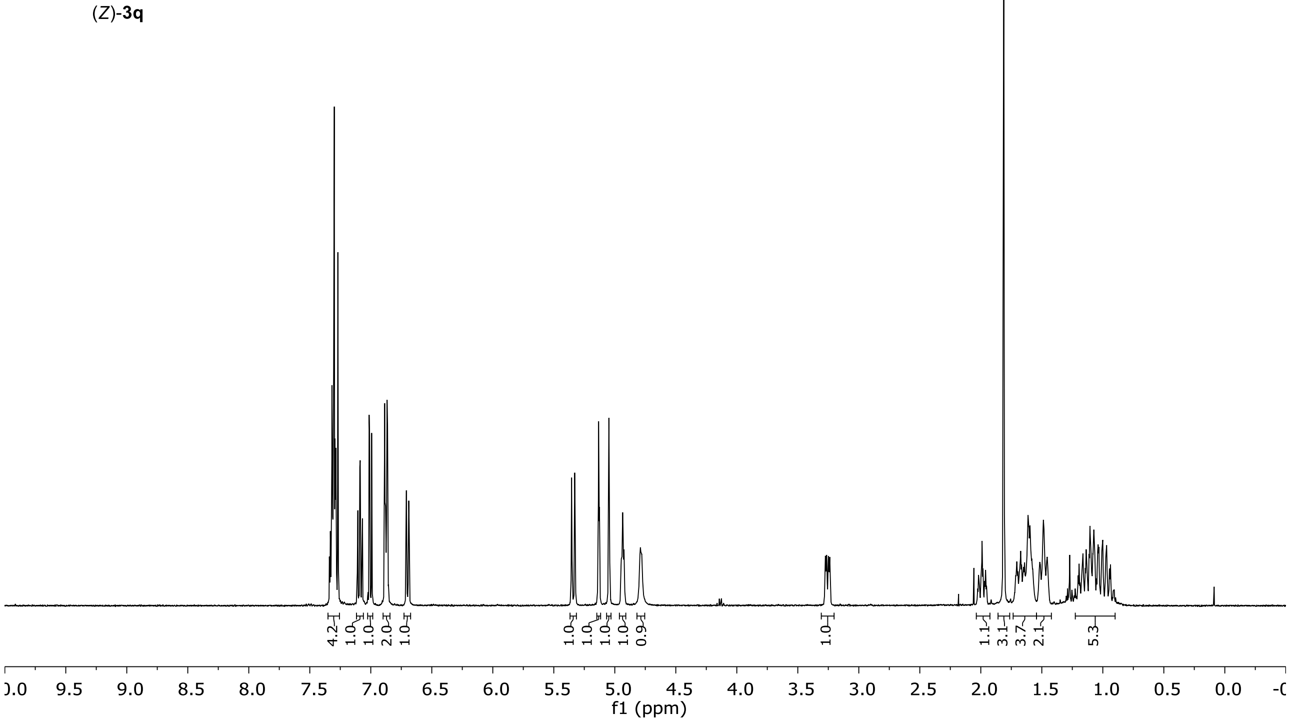
4.2
1.0
1.0
2.0
1.0
1.0
1.0
1.0
1.0
0.9
1.0
1.1
3.1
3.7
2.1
5.3
f1 (ppm)

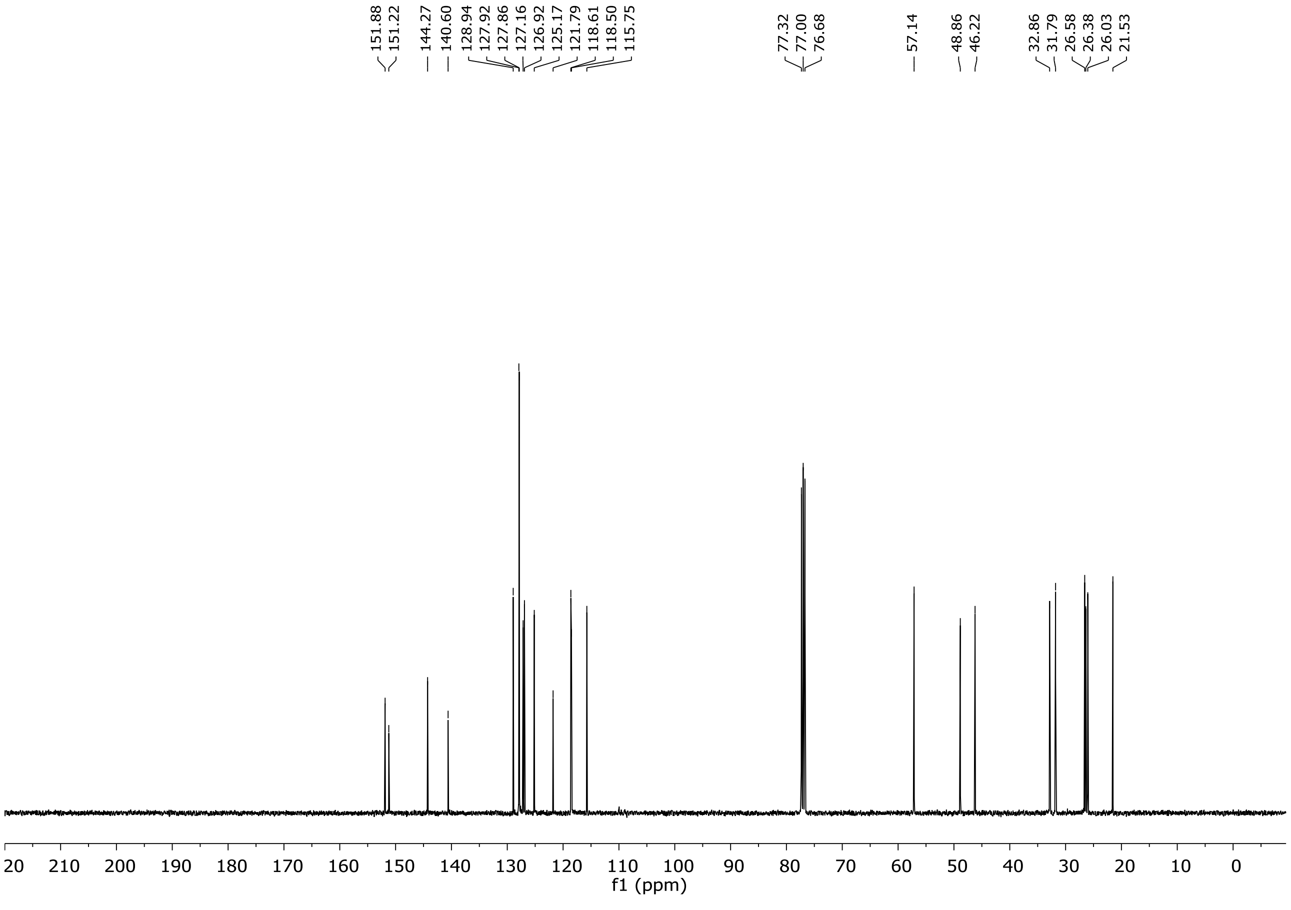
151.88
151.22
144.27
140.60
128.94
127.92
127.86
127.16
126.92
125.17
121.79
118.61
118.50
115.75
77.32
77.00
76.68
57.14
48.86
46.22
32.86
31.79
26.58
26.38
26.03
21.53
f1 (ppm)

NOESY

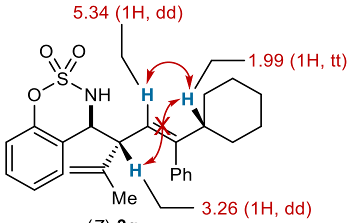

(*Z*)-**3q**

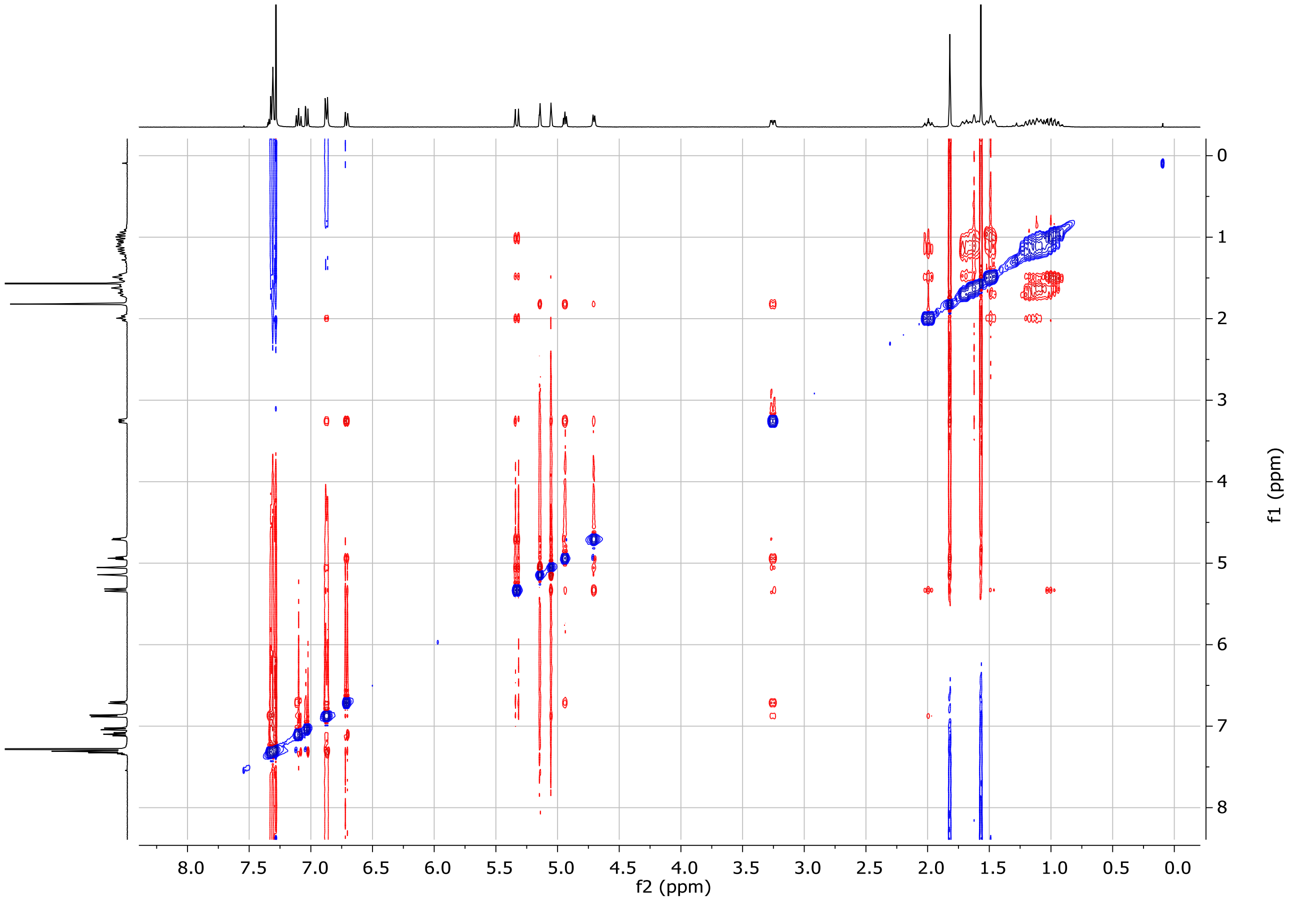

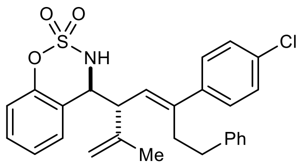
O O
S
O
NH
Cl
Me
Ph

3r

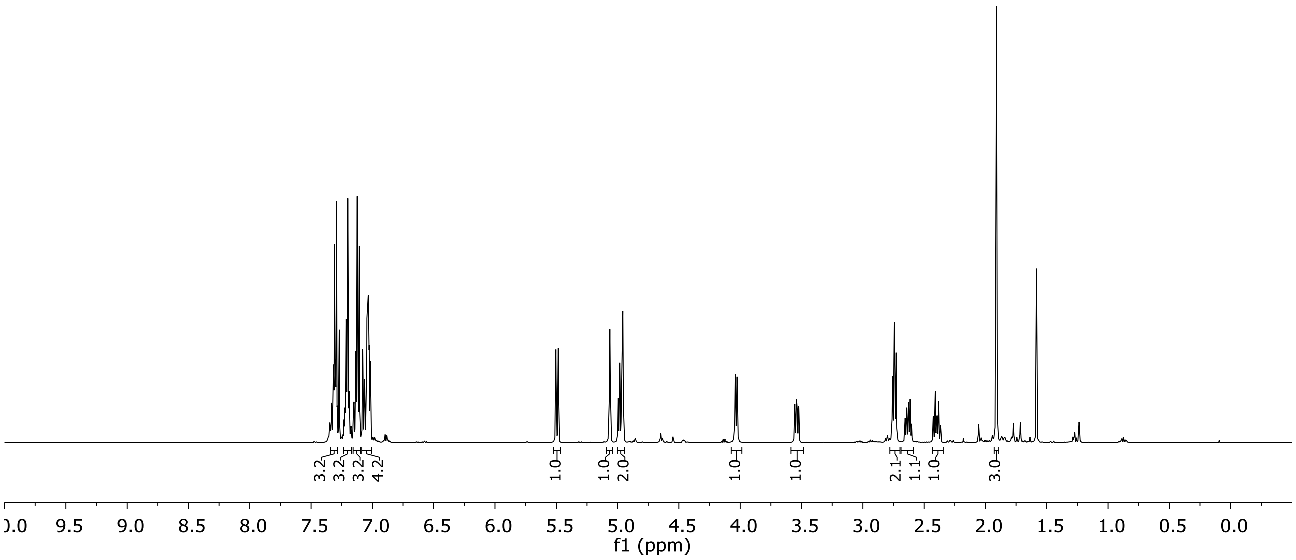
3.2
3.2
3.2
3.2
4.2
1.0
1.0
2.0
1.0
1.0
2.1
1.1
1.0
3.0
f1 (ppm)

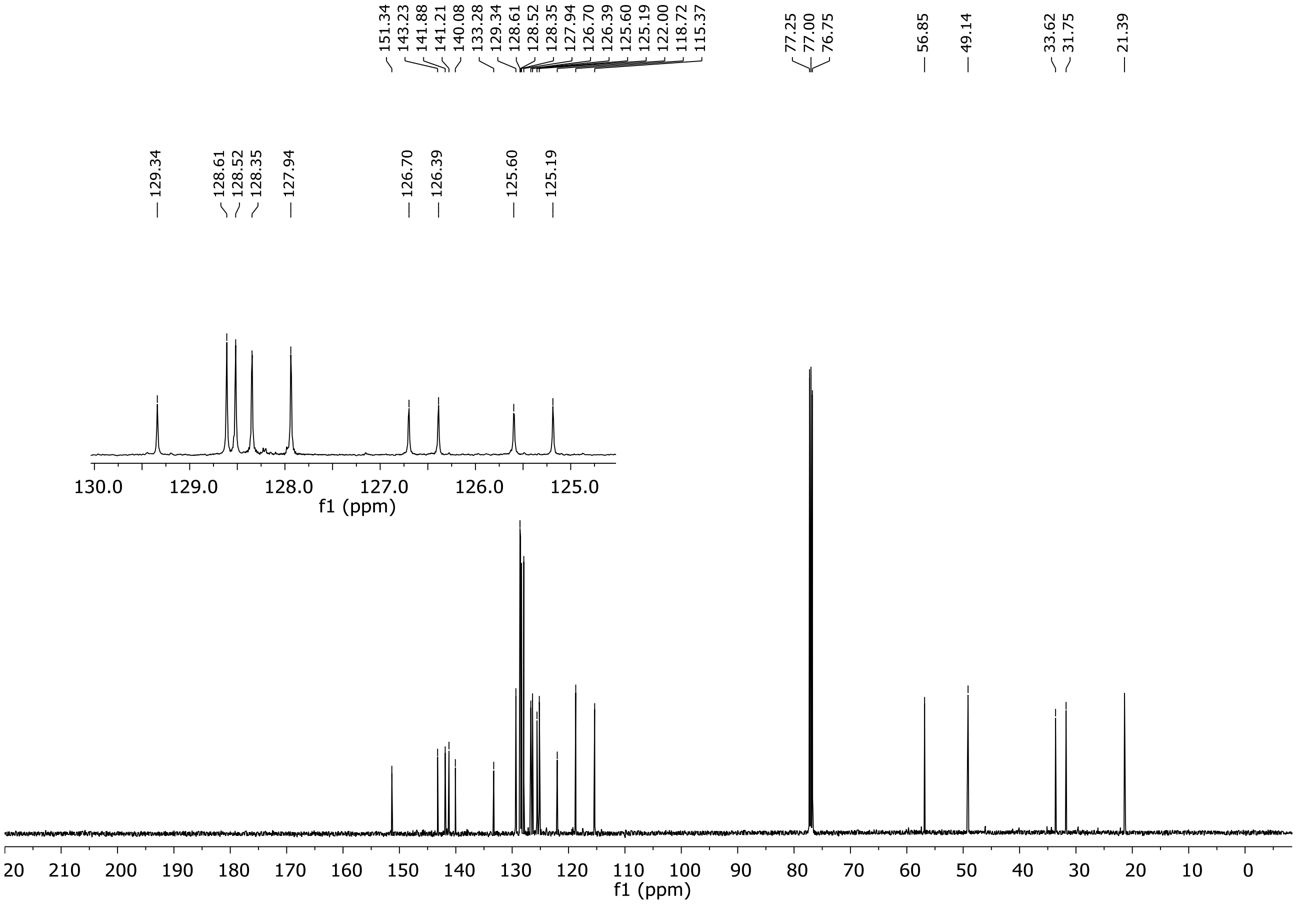
151.34
143.23
141.88
141.21
140.08
133.28
129.34
128.61
128.52
128.35
127.94
126.70
126.39
125.60
125.19
122.00
118.72
115.37
77.25
77.00
76.75
56.85
49.14
33.62
31.75
21.39
129.34
128.61
128.52
128.35
127.94
126.70
126.39
125.60
125.19
f1 (ppm)

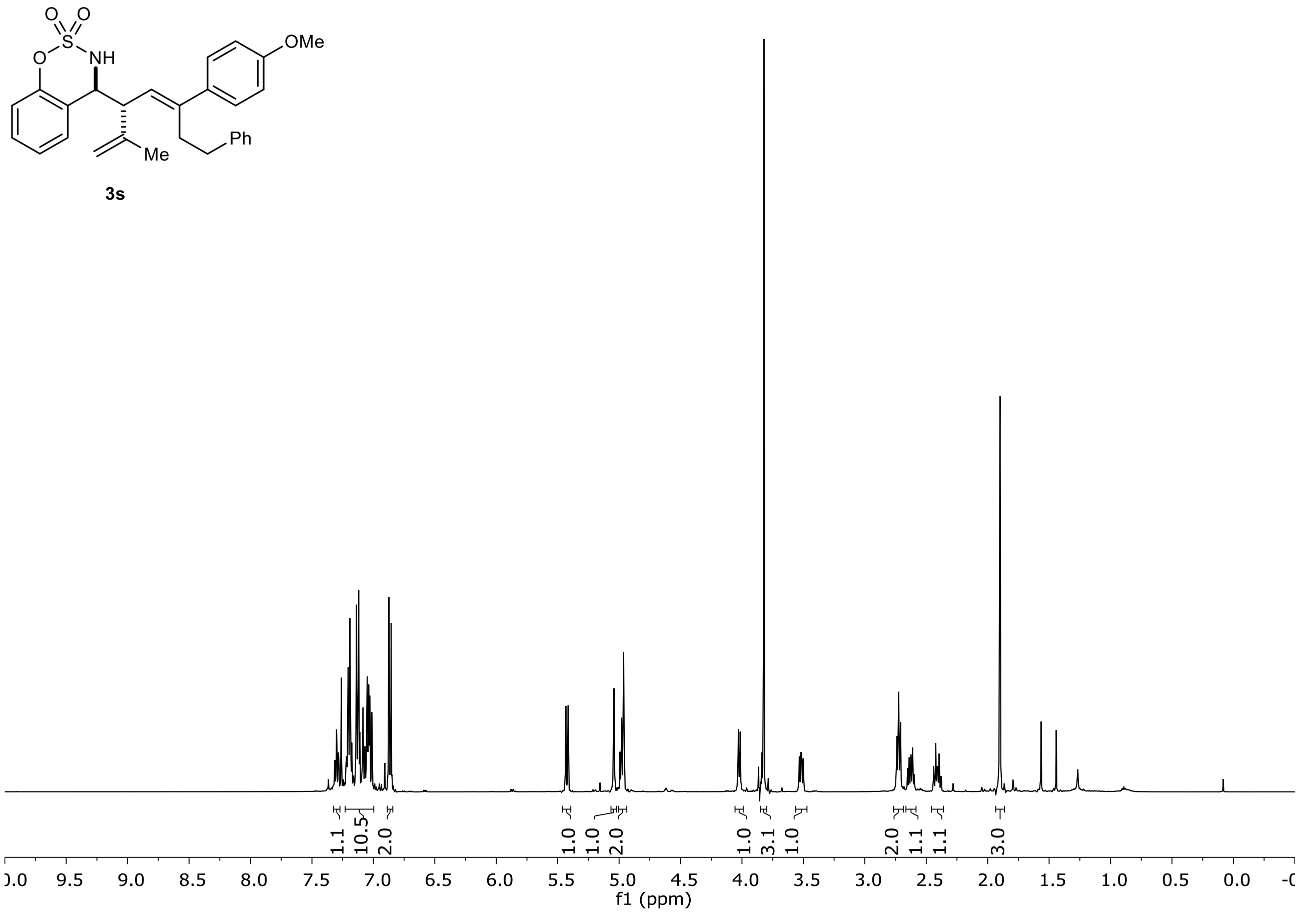
OMe
NH
Me
Ph

3s

1.1
10.5
2.0
1.0
1.0
2.0
1.0
3.1
1.0
2.0
1.1
1.1
3.0
f1 (ppm)

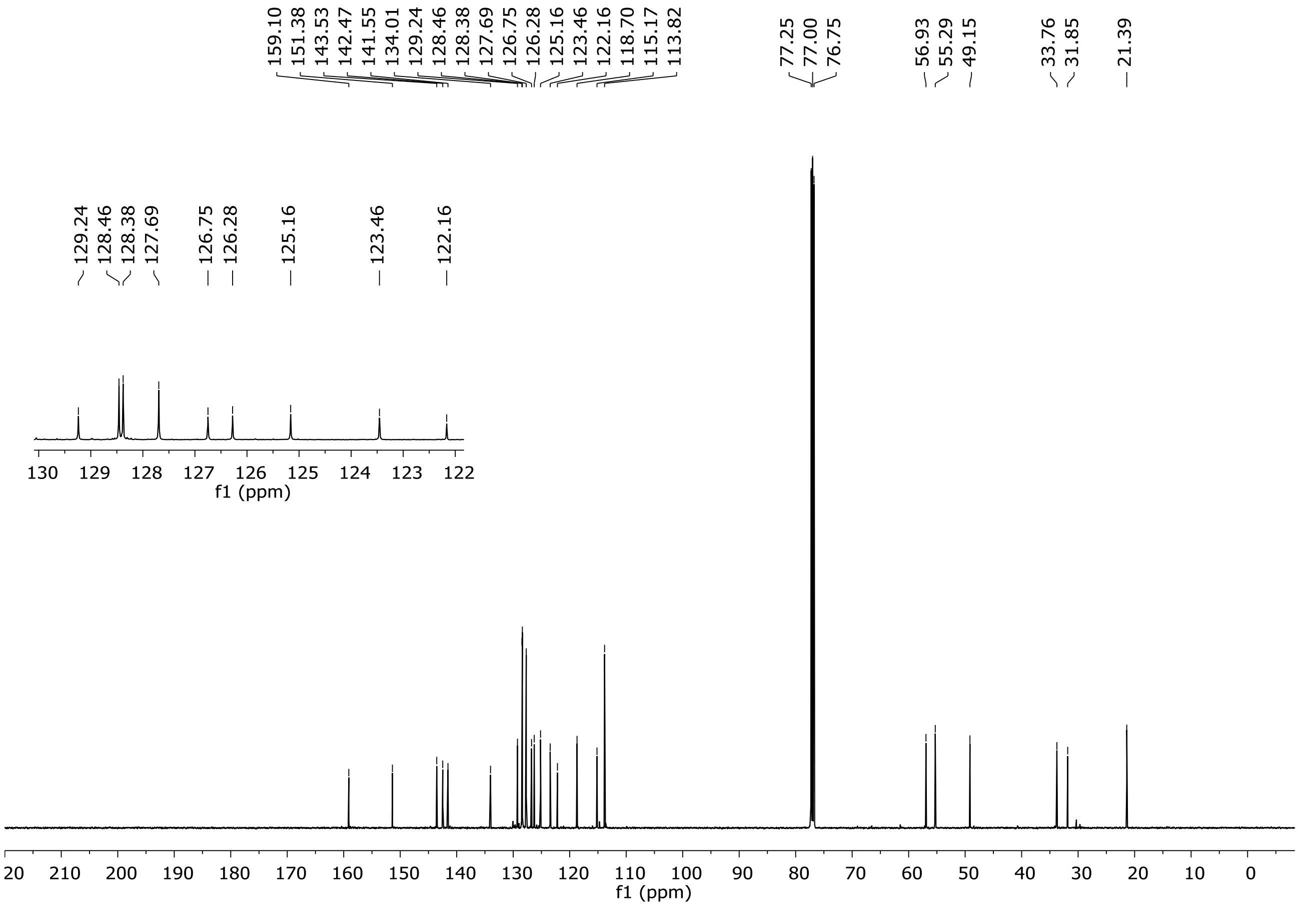
159.10
151.38
143.53
142.47
141.55
134.01
129.24
128.46
128.38
127.69
126.75
126.28
125.16
123.46
122.16
118.70
115.17
113.82
77.25
77.00
76.75
56.93
55.29
49.15
33.76
31.85
21.39
f1 (ppm)

129.24
128.46
128.38
127.69
126.75
126.28
125.16
123.46
122.16
f1 (ppm)

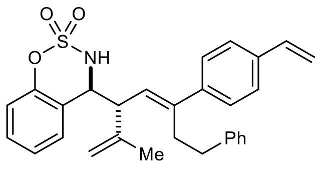
O
O
S
O
NH
Me
Ph

3t

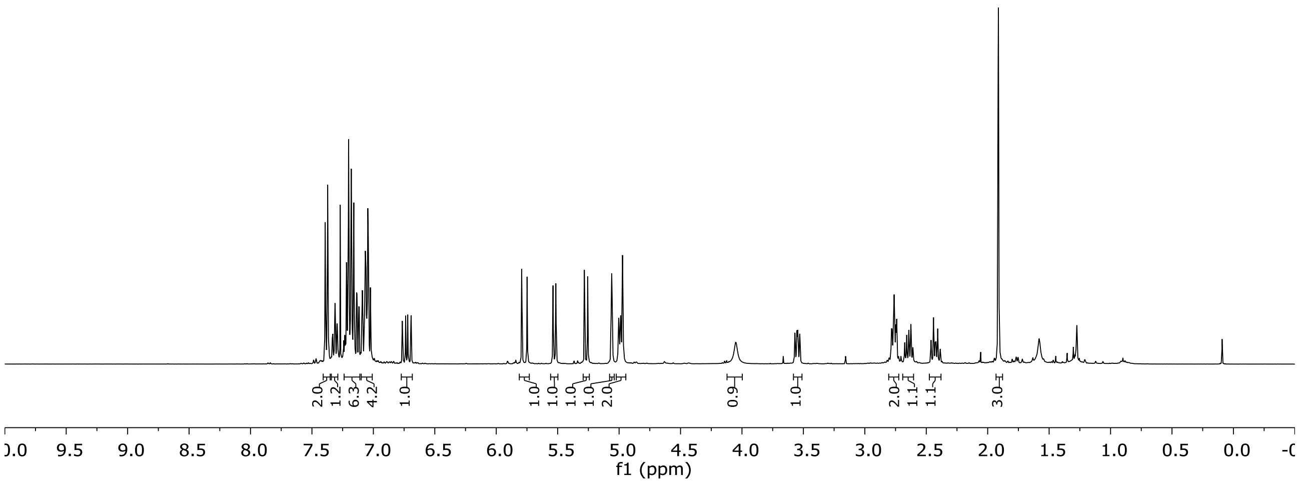
2.0
1.2
6.3
4.2
1.0
1.0
1.0
1.0
2.0
0.9
1.0
2.0
1.1
1.1
3.0
f1 (ppm)

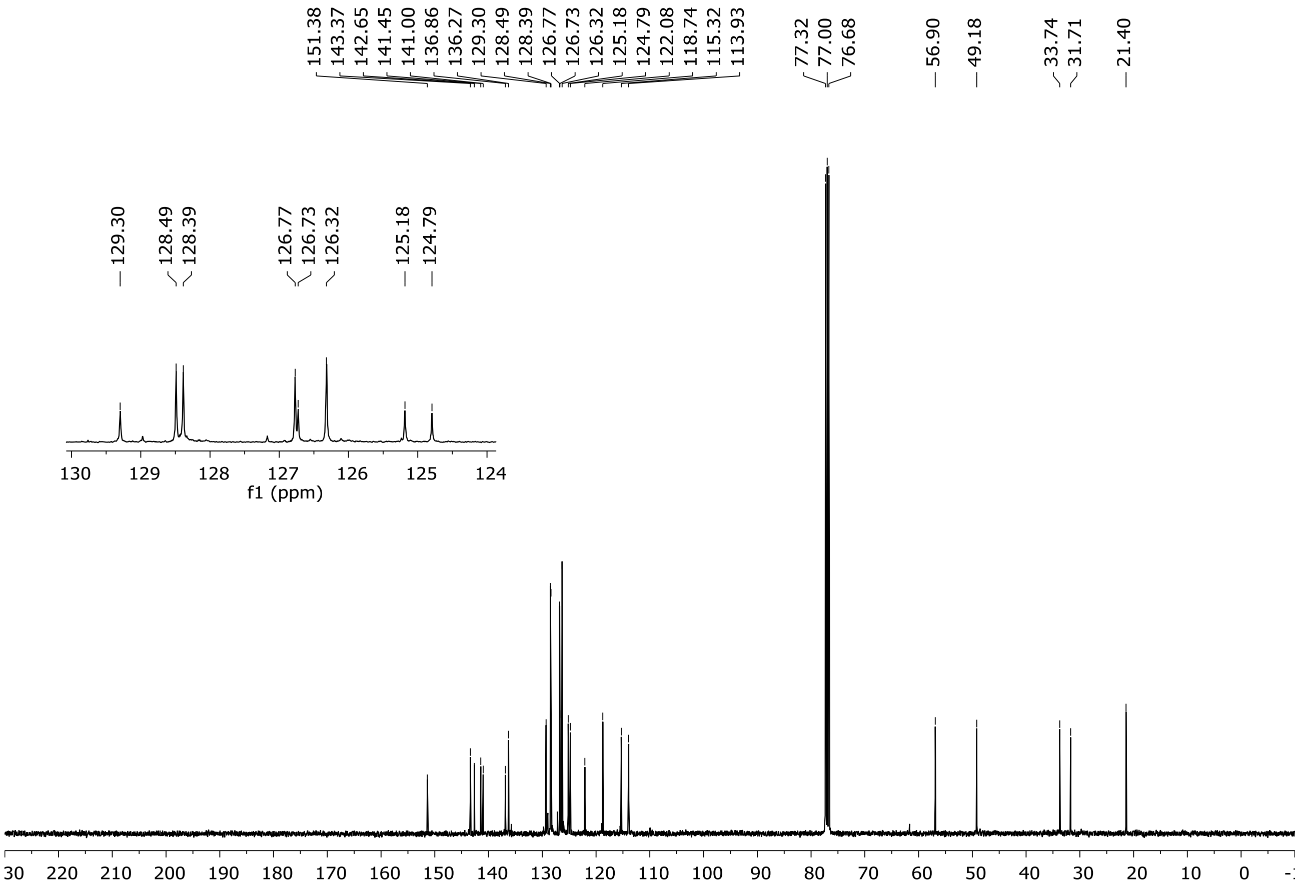
151.38
143.37
142.65
141.45
141.00
136.86
136.27
129.30
128.49
128.39
126.77
126.73
126.32
125.18
124.79
122.08
118.74
115.32
113.93
77.32
77.00
76.68
56.90
49.18
33.74
31.71
21.40
129.30
128.49
128.39
126.77
126.73
126.32
125.18
124.79
f1 (ppm)
f1 (ppm)

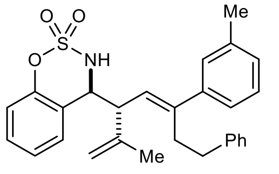
O
O
S
NH
O
Me
Ph
Me

3u

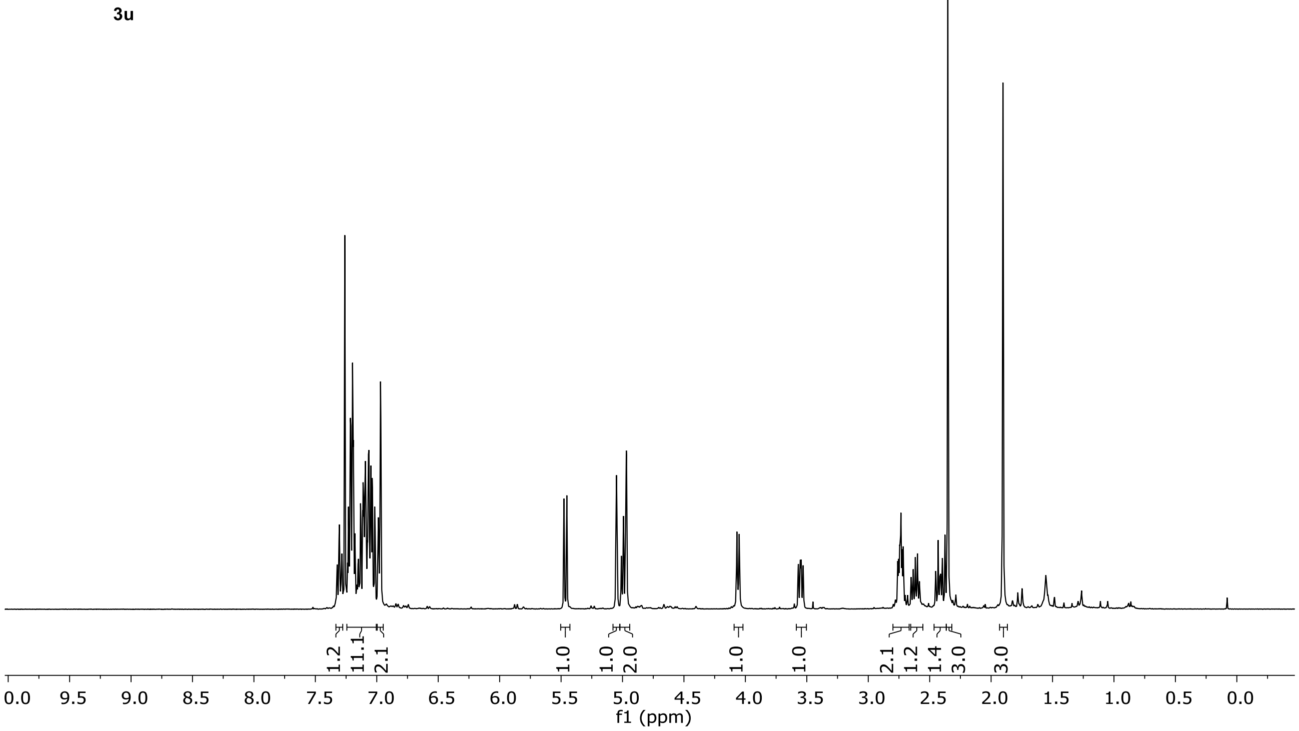
1.2
11.1
2.1
1.0
1.0
2.0
1.0
1.0
2.1
1.2
1.4
3.0
3.0
0.0 9.5 9.0 8.5 8.0 7.5 7.0 6.5 6.0 5.5 5.0 4.5 4.0 3.5 3.0 2.5 2.0 1.5 1.0 0.5 0.0
f1 (ppm)

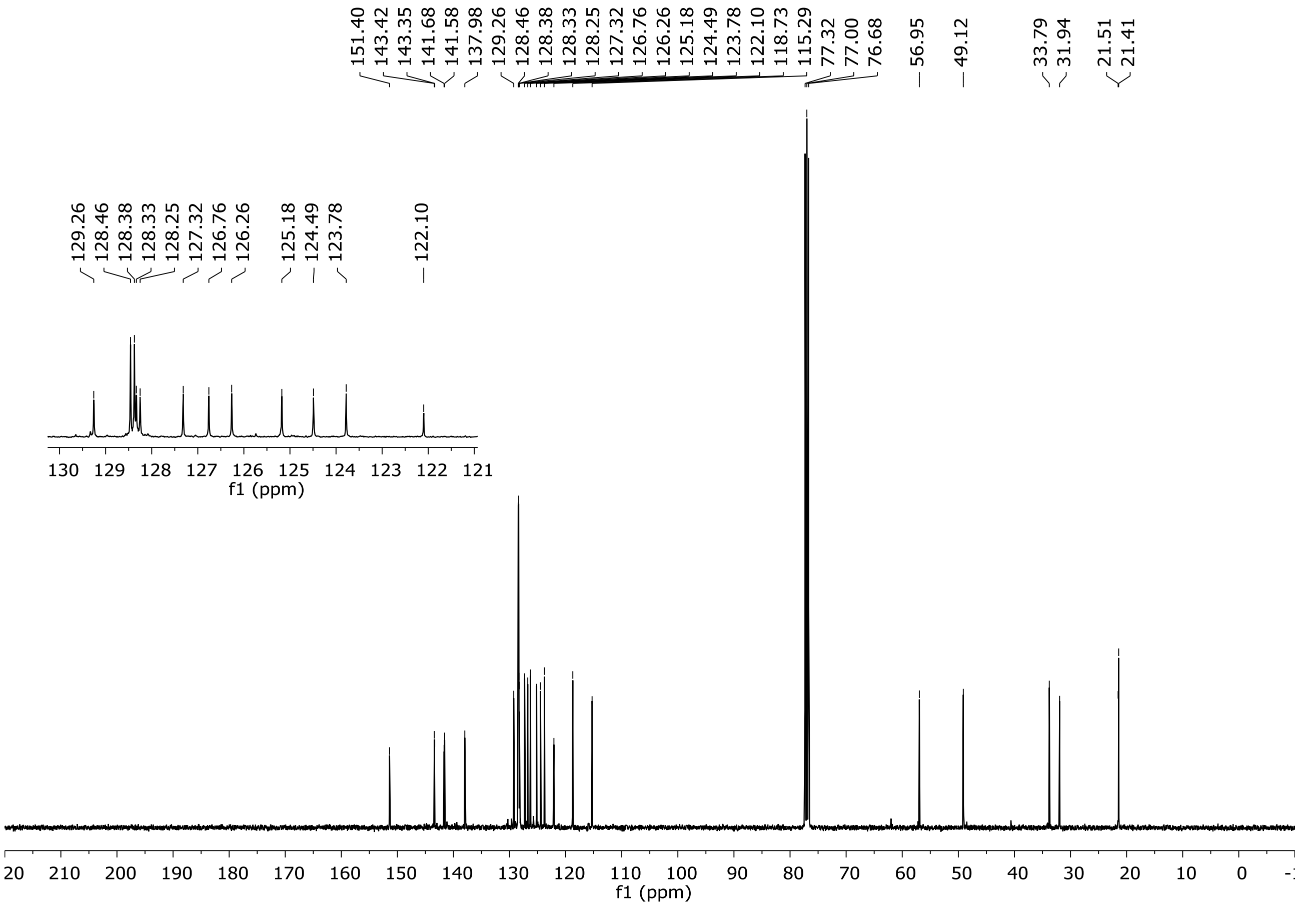
151.40
143.42
143.35
141.68
141.58
137.98
129.26
128.46
128.38
128.33
128.25
127.32
126.76
126.26
125.18
124.49
123.78
122.10
118.73
115.29
77.32
77.00
76.68
56.95
49.12
33.79
31.94
21.51
21.41
129.26
128.46
128.38
128.33
128.25
127.32
126.76
126.26
125.18
124.49
123.78
122.10
130 129 128 127 126 125 124 123 122 121
f1 (ppm)
20 210 200 190 180 170 160 150 140 130 120 110 100 90 80 70 60 50 40 30 20 10 0
f1 (ppm)

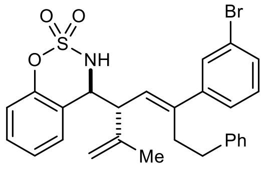
O O
S
O
NH
Br
Me
Ph

3v

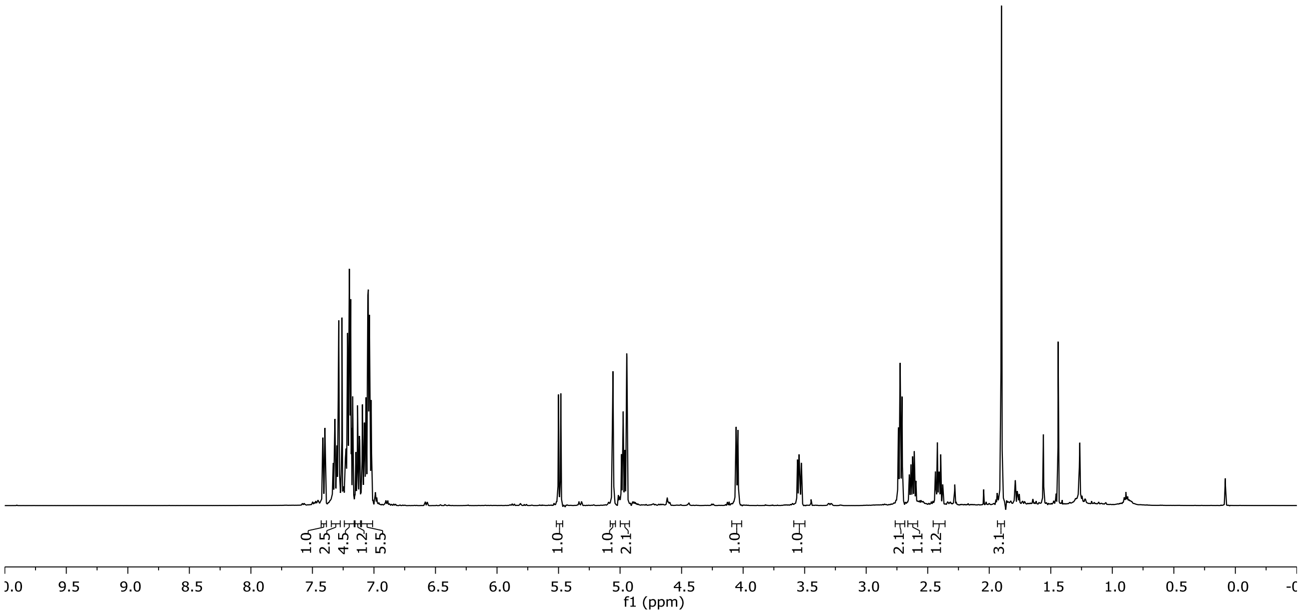
1.0
2.5
4.5
1.2
5.5
1.0
1.0
2.1
1.0
1.0
2.1
1.1
1.2
3.1
9.5 9.0 8.5 8.0 7.5 7.0 6.5 6.0 5.5 5.0 4.5 4.0 3.5 3.0 2.5 2.0 1.5 1.0 0.5 0.0
f1 (ppm)

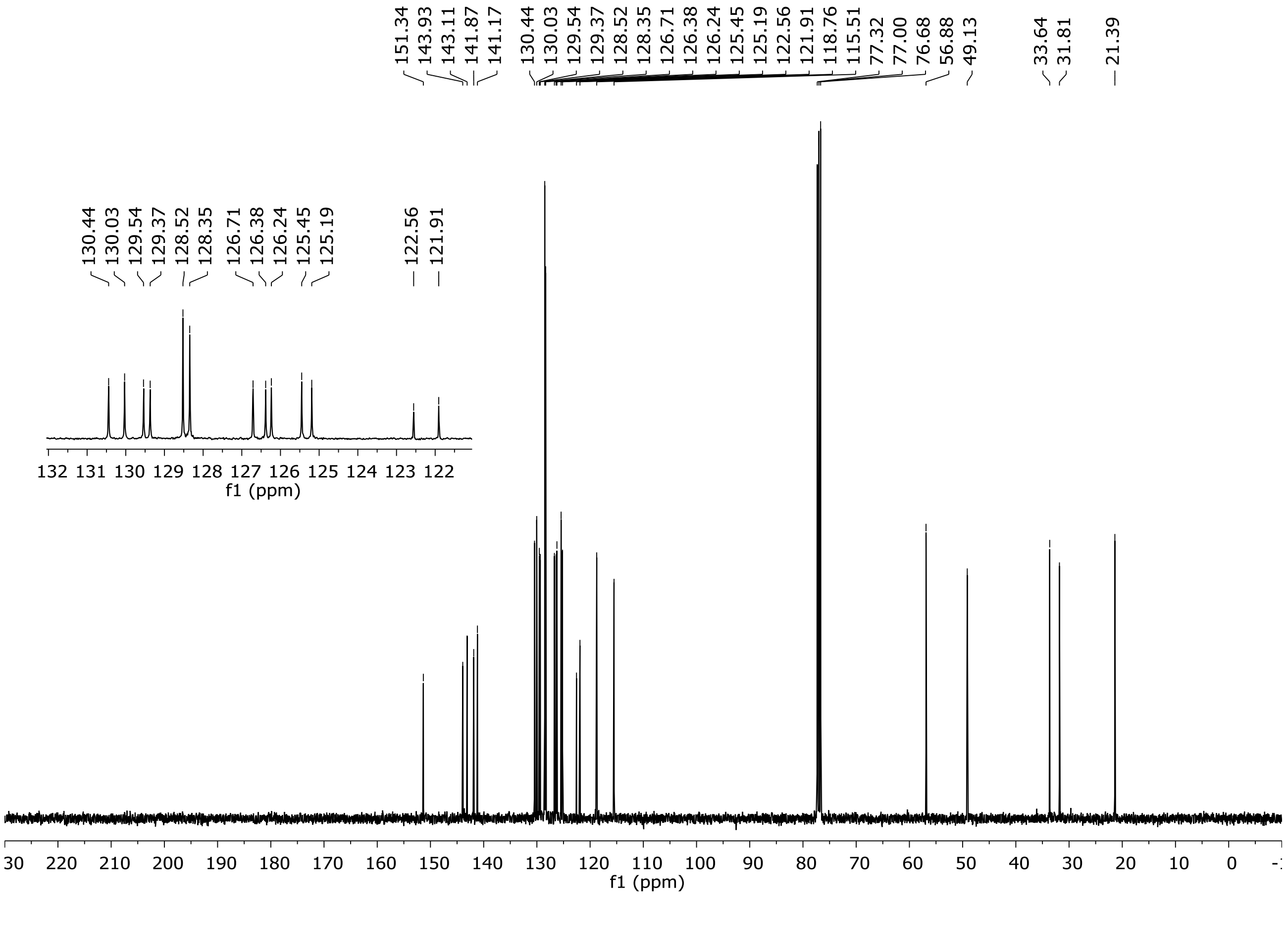
151.34
143.93
143.11
141.87
141.17
130.44
130.03
129.54
129.37
128.52
128.35
126.71
126.38
126.24
125.45
125.19
122.56
121.91
118.76
115.51
77.32
77.00
76.68
56.88
49.13
33.64
31.81
21.39
132 131 130 129 128 127 126 125 124 123 122
f1 (ppm)
220 210 200 190 180 170 160 150 140 130 120 110 100 90 80 70 60 50 40 30 20 10 0
f1 (ppm)

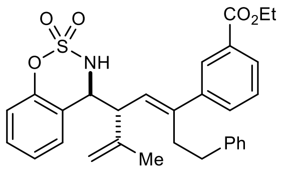

CO2Et
O
O
S
O
NH
Me
Ph

3w

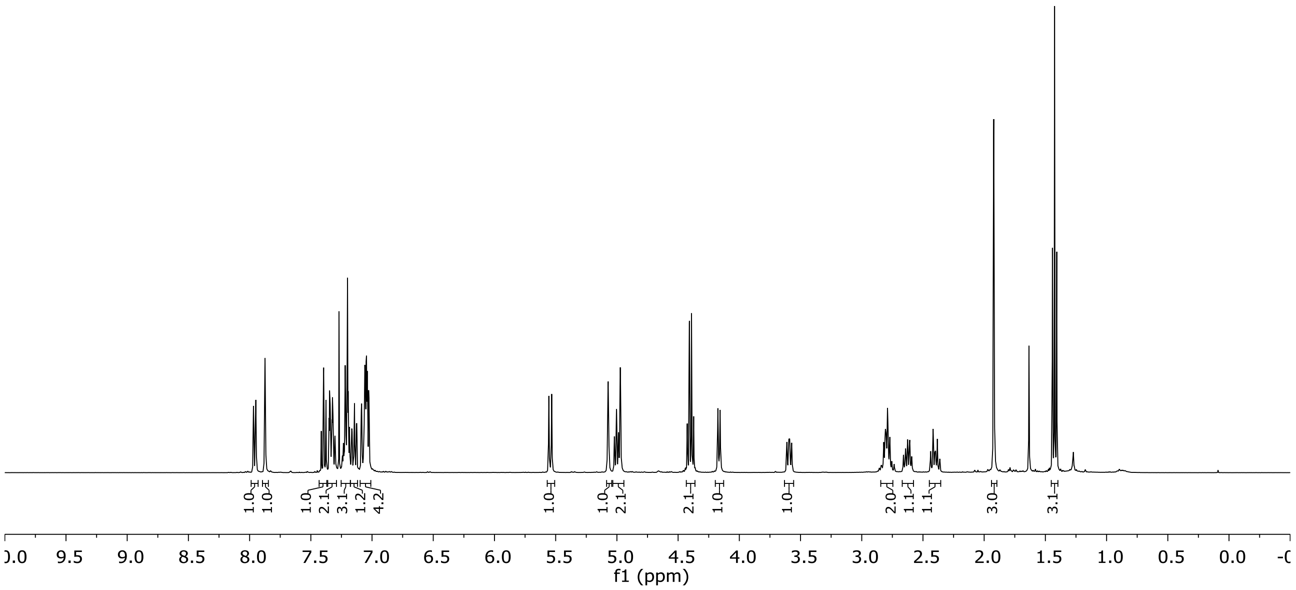

1.0
1.0
1.0
2.1
3.1
1.2
4.2
1.0
1.0
2.1
2.1
1.0
1.0
2.0
1.1
1.1
3.0
3.1
f1 (ppm)

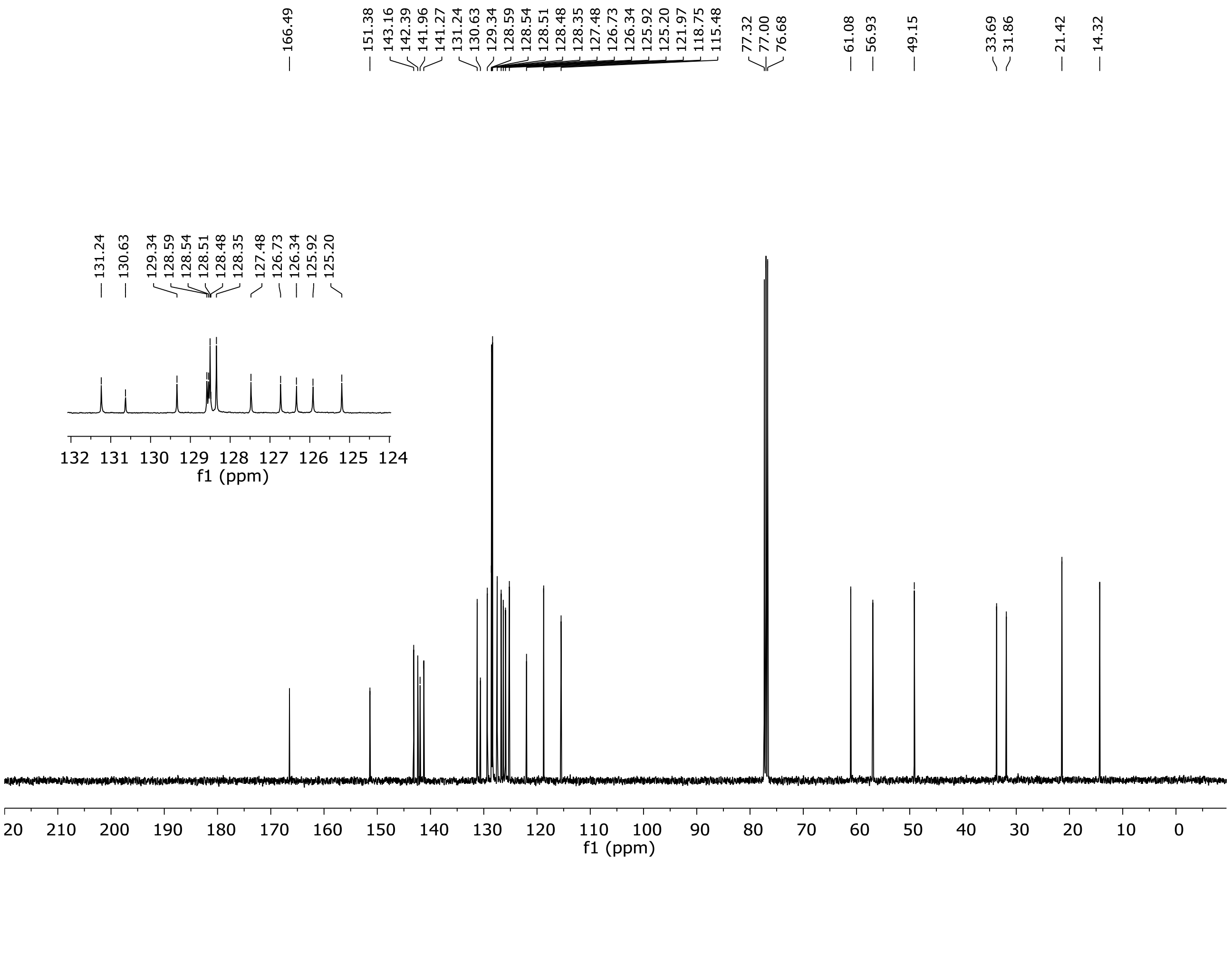

166.49
151.38
143.16
142.39
141.96
141.27
131.24
130.63
129.34
128.59
128.54
128.51
128.48
128.35
127.48
126.73
126.34
125.92
125.20
121.97
118.75
115.48
77.32
77.00
76.68
61.08
56.93
49.15
33.69
31.86
21.42
14.32
f1 (ppm)

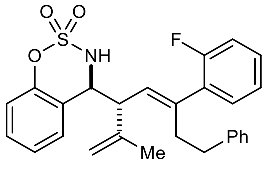
O
O
S
O
NH
F
Me
Ph

3x

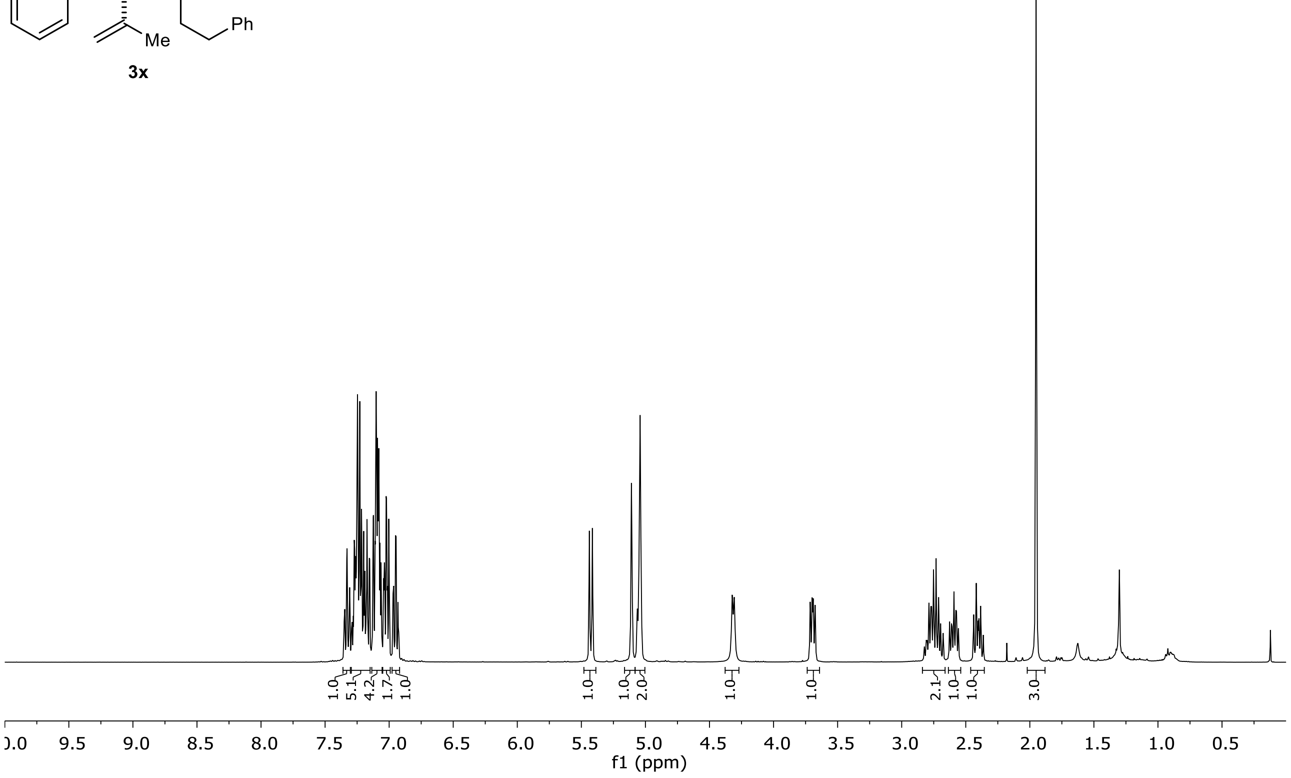
1.0
5.1
4.2
1.7
1.0
1.0
1.0
2.0
1.0
1.0
2.1
1.0
1.0
3.0
f1 (ppm)

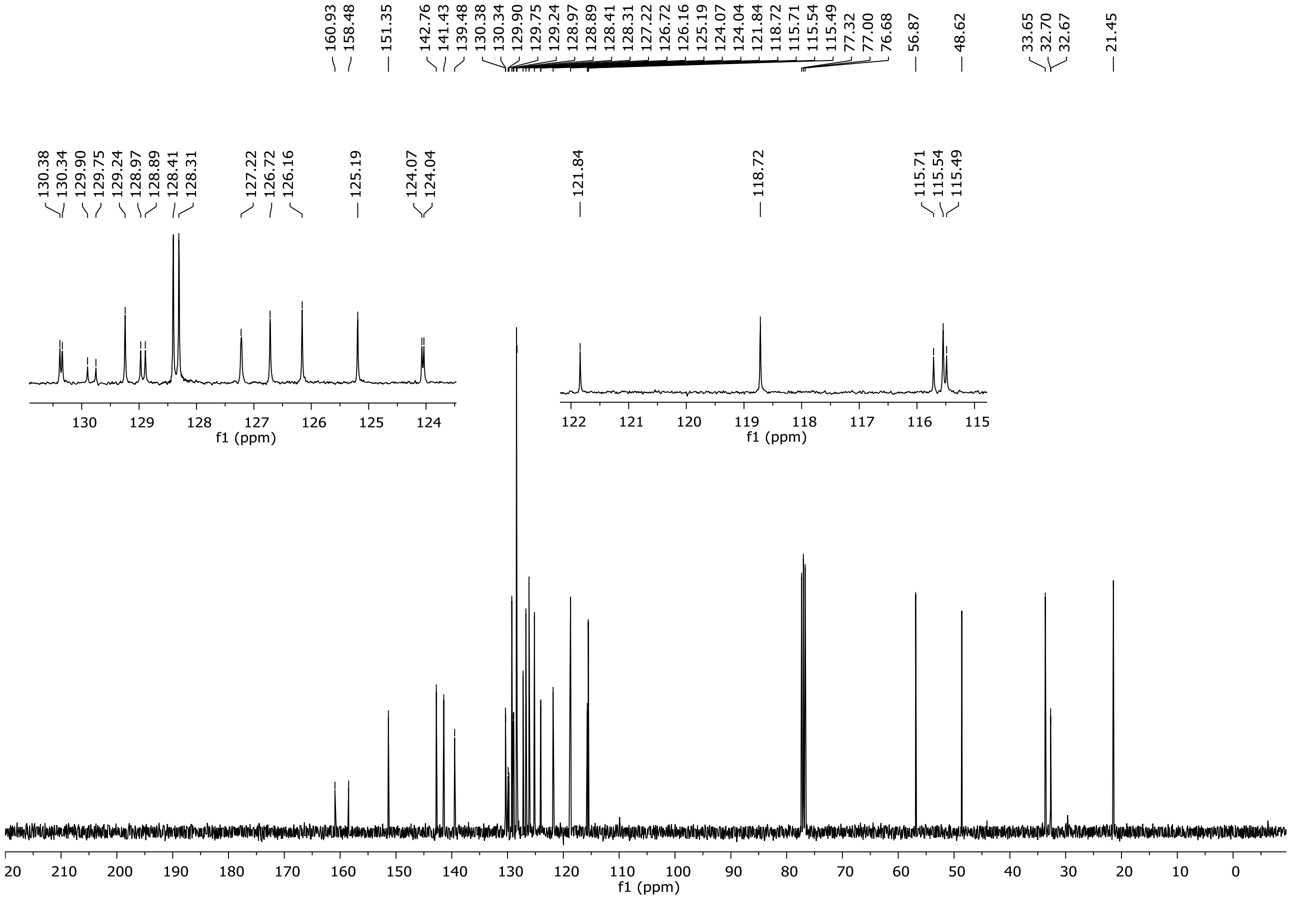
160.93
158.48
151.35
142.76
141.43
139.48
130.38
130.34
129.90
129.75
129.24
128.97
128.89
128.41
128.31
127.22
126.72
126.16
125.19
124.07
124.04
121.84
118.72
115.71
115.54
115.49
77.32
77.00
76.68
56.87
48.62
33.65
32.70
32.67
21.45
f1 (ppm)

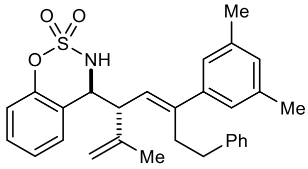

O
O
S
O
NH
Me
Me
Me
Ph

3y

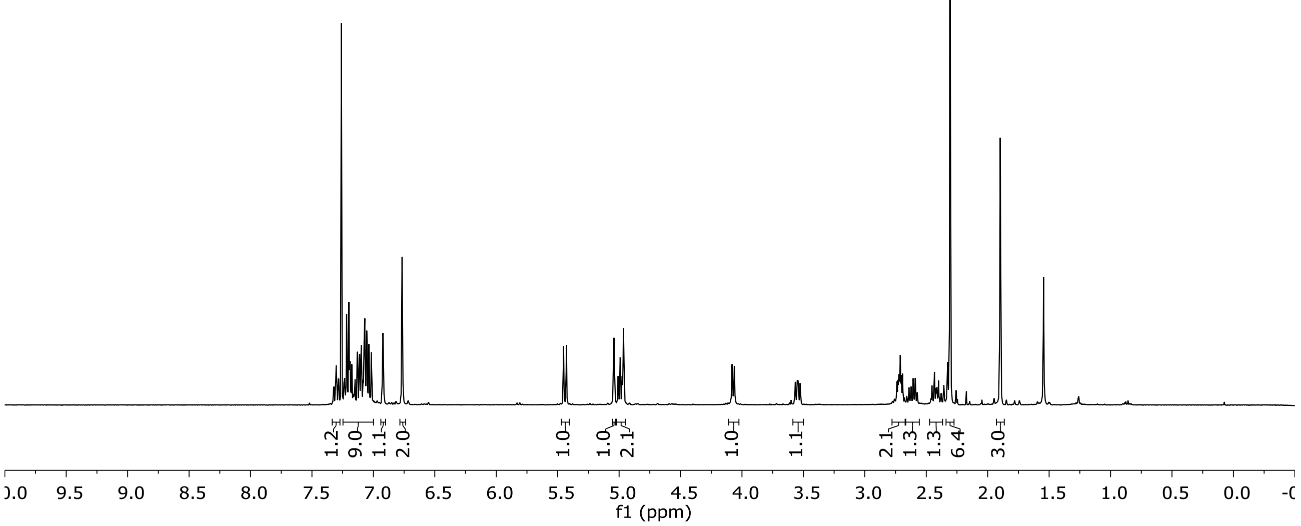

1.2
9.0
1.1
2.0
1.0
1.0
2.1
1.0
1.1
2.1
1.3
1.3
6.4
3.0
f1 (ppm)

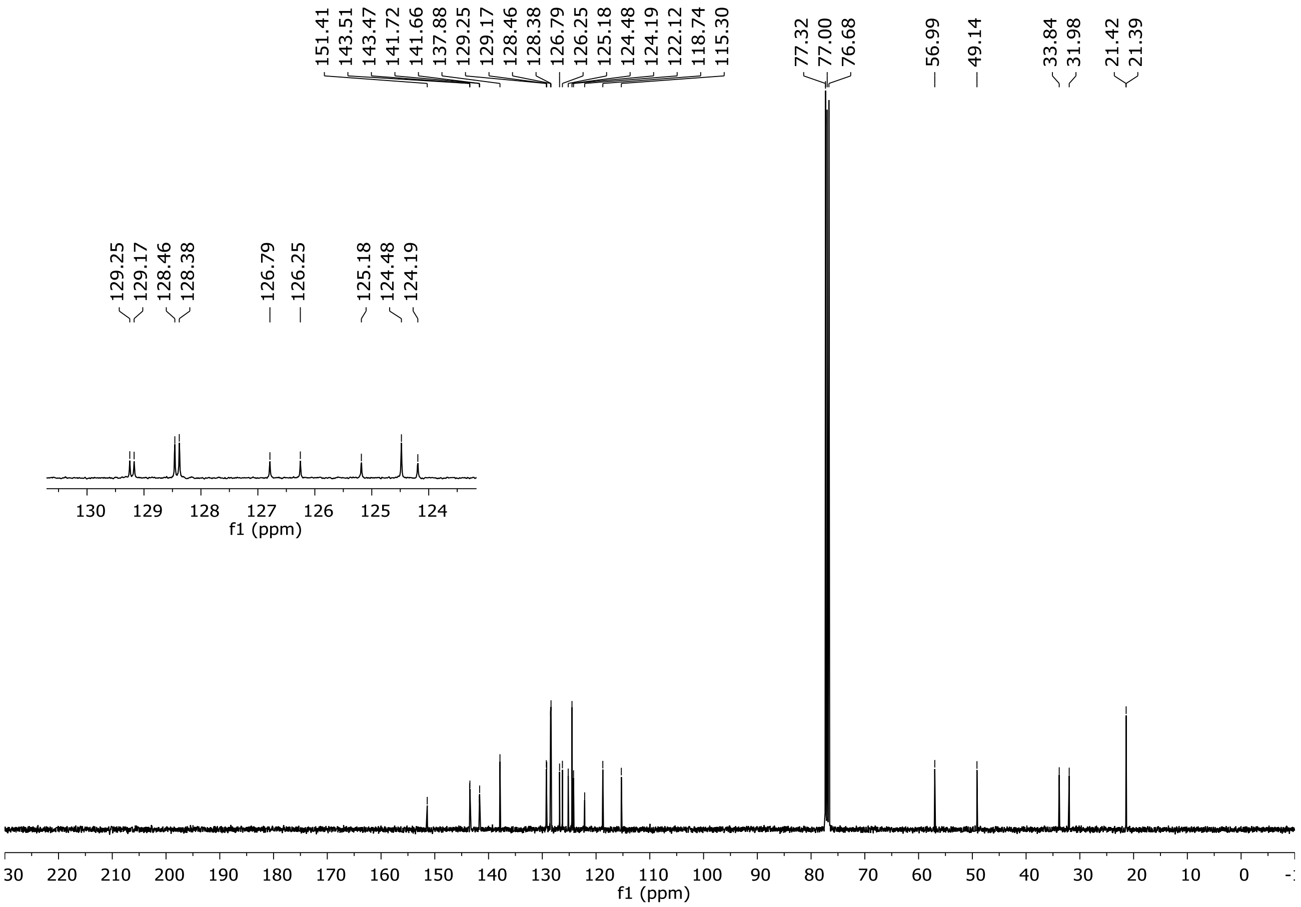

151.41
143.51
143.47
141.72
141.66
137.88
129.25
129.17
128.46
128.38
126.79
126.25
125.18
124.48
124.19
122.12
118.74
115.30
77.32
77.00
76.68
56.99
49.14
33.84
31.98
21.42
21.39
f1 (ppm)

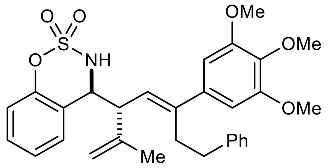

3z

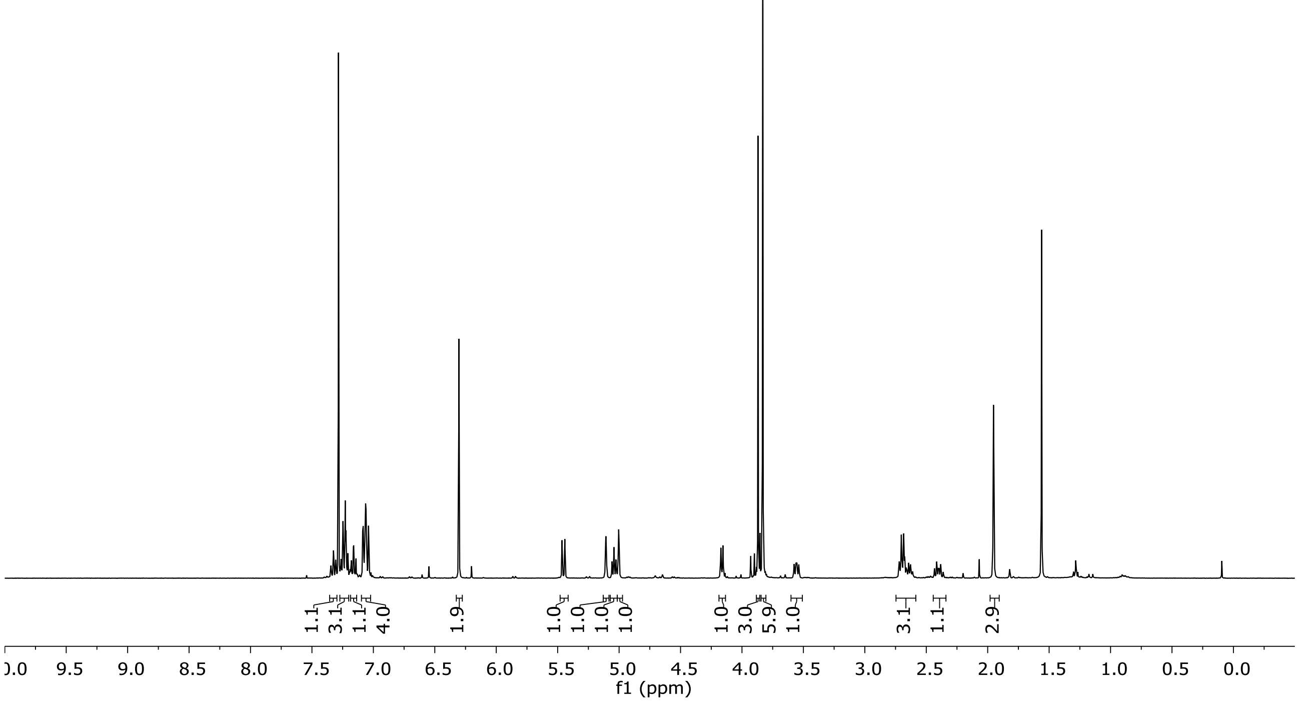

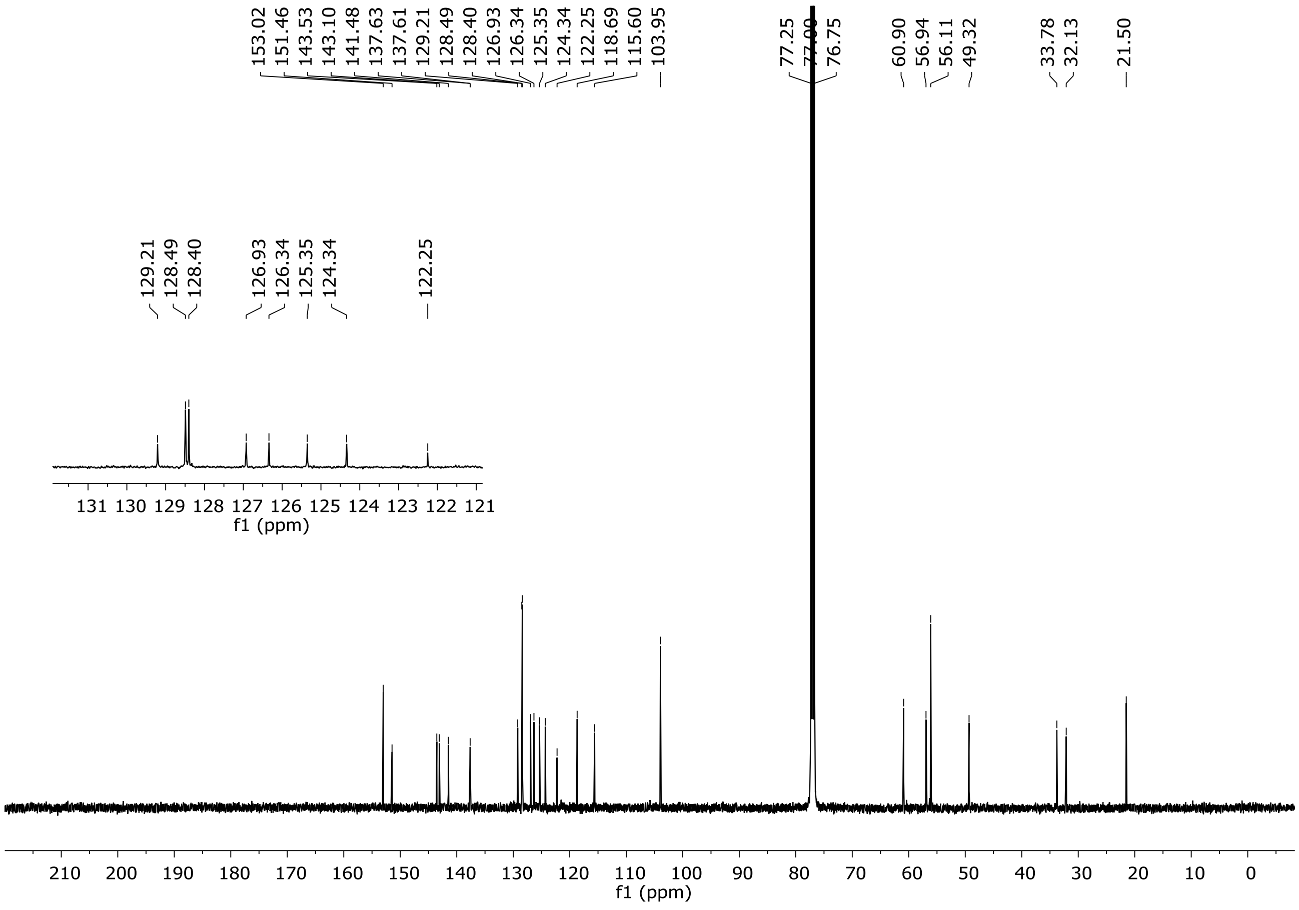

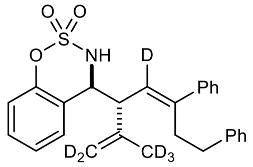
O
O
S
O
NH
D
Ph
Ph
D_2C
CD_3

$[D]_6$-**3a**

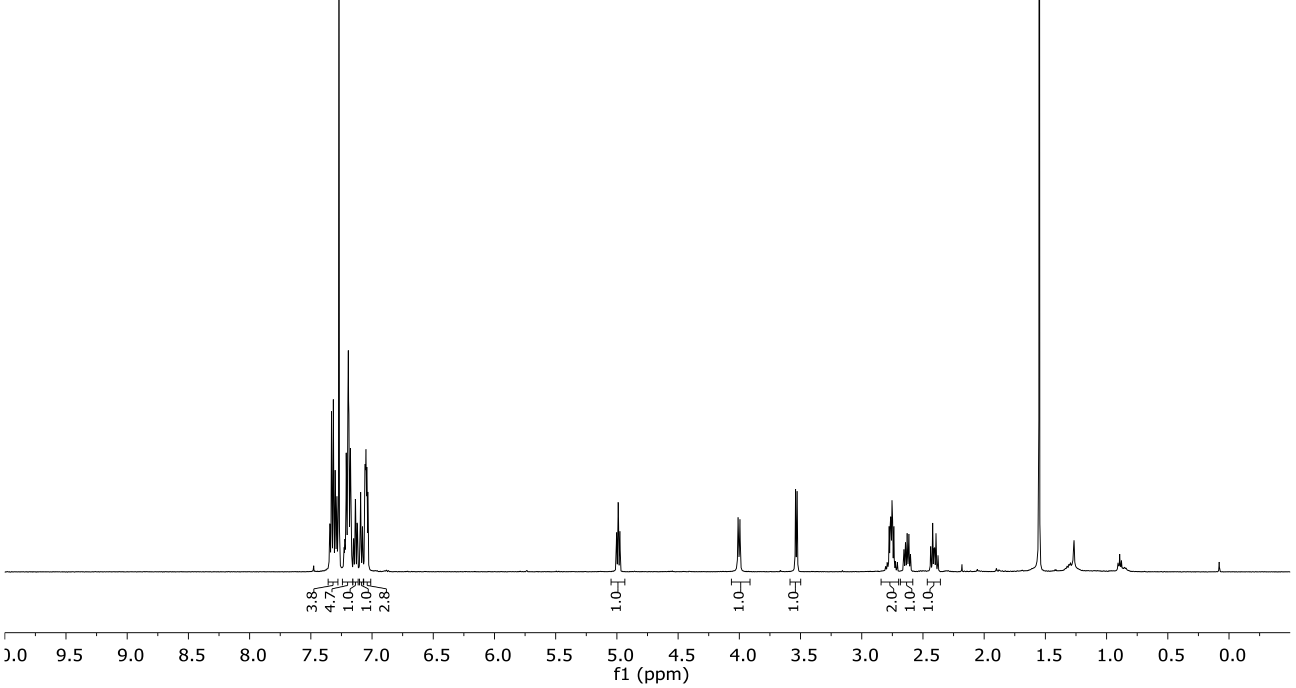
3.8
4.7
1.0
1.0
2.8
1.0
1.0
1.0
2.0
1.0
1.0
f1 (ppm)

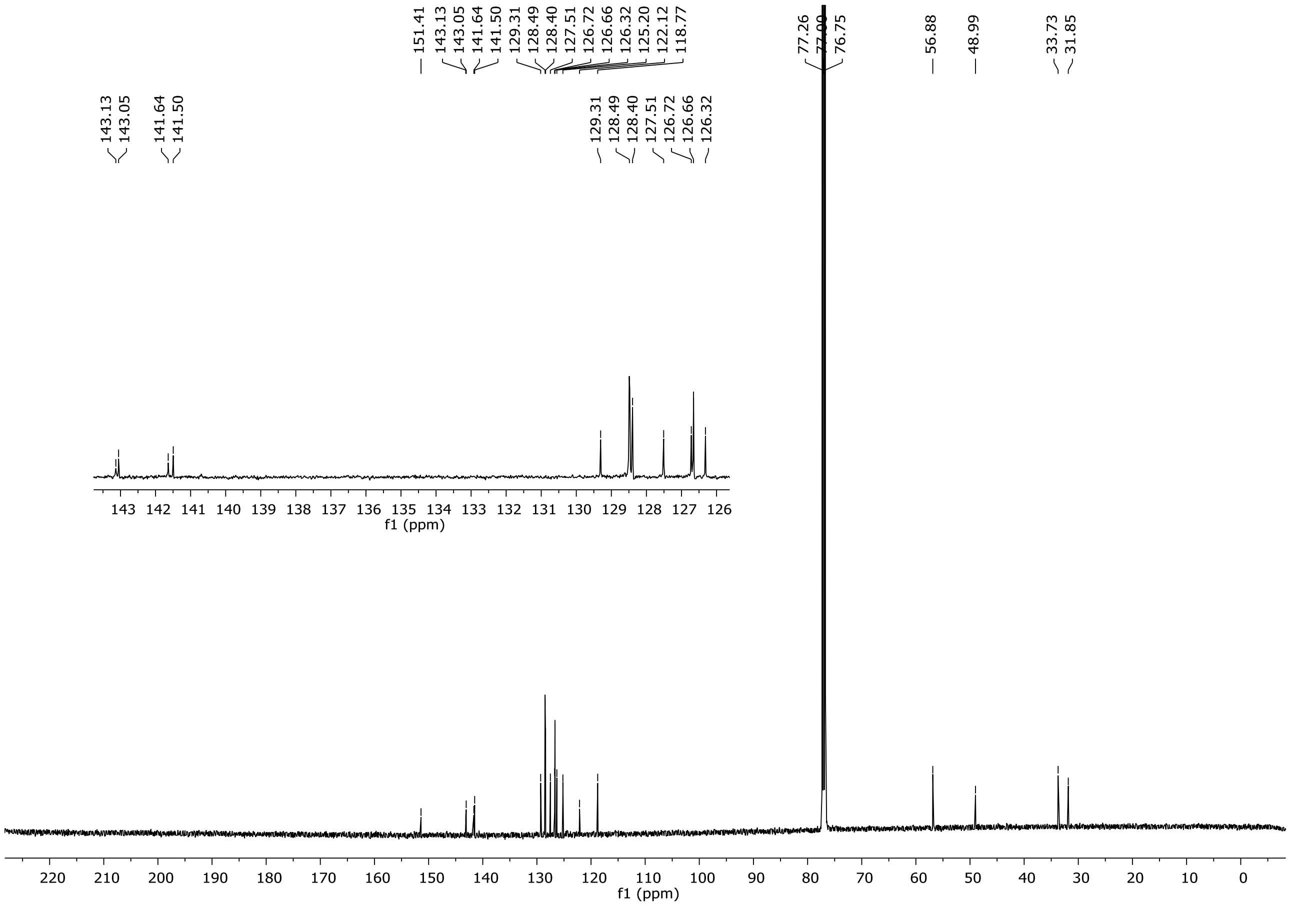
151.41
143.13
143.05
141.64
141.50
129.31
128.49
128.40
127.51
126.72
126.66
126.32
125.20
122.12
118.77
77.26
77.00
76.75
56.88
48.99
33.73
31.85
f1 (ppm)

7. HPLC Traces

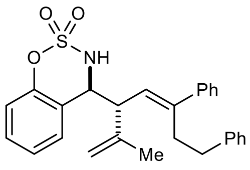

3a

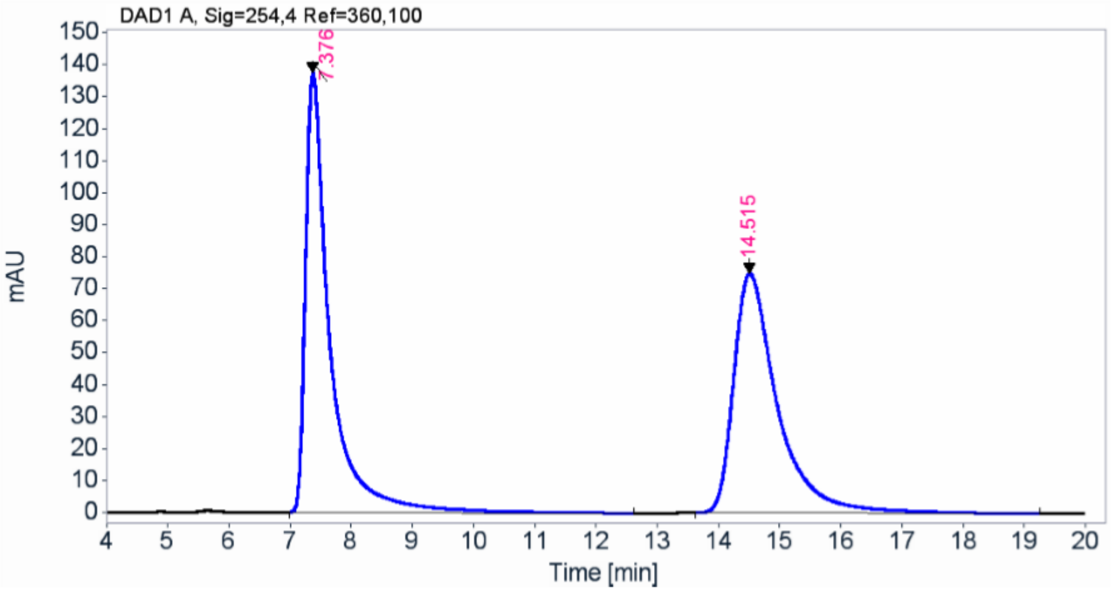

Signal: DAD1 A, Sig=254,4 Ref=360,100

RT [min]	Type	Width [min]	Area	Height	Area%
7.376	BB	0.3964	3795.119	137.3302	50.51
14.515	BB	0.7362	3717.759	74.7578	49.49

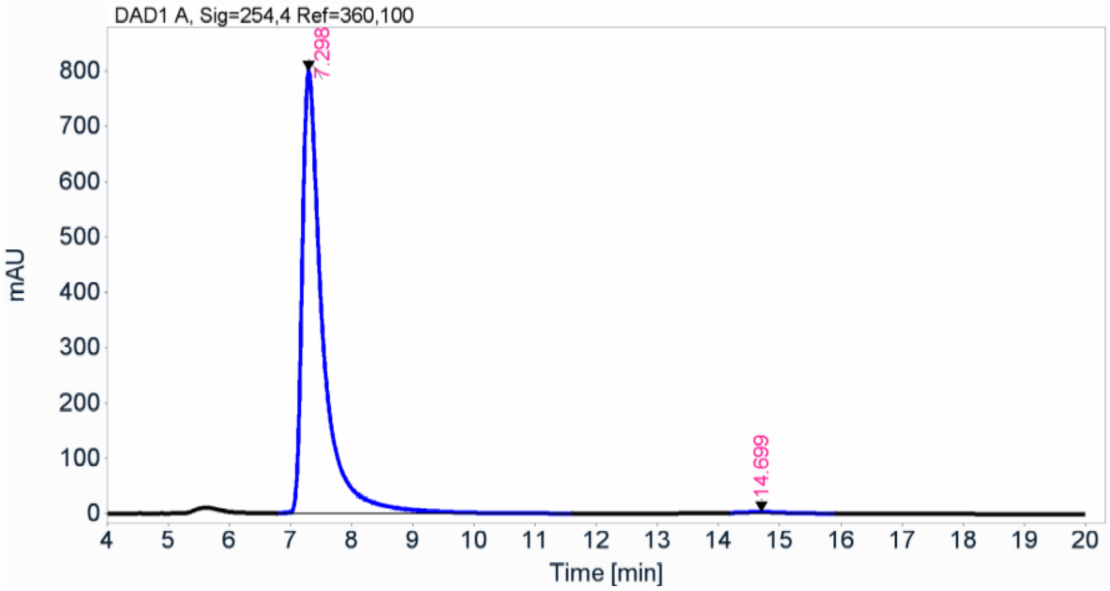

Signal: DAD1 A, Sig=254,4 Ref=360,100

RT [min]	Type	Width [min]	Area	Height	Area%
7.298	MM	0.3887	18621.953	798.5695	99.32
14.699	MM	0.8753	127.875	2.4350	0.68

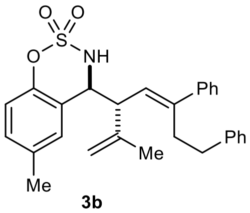

3b

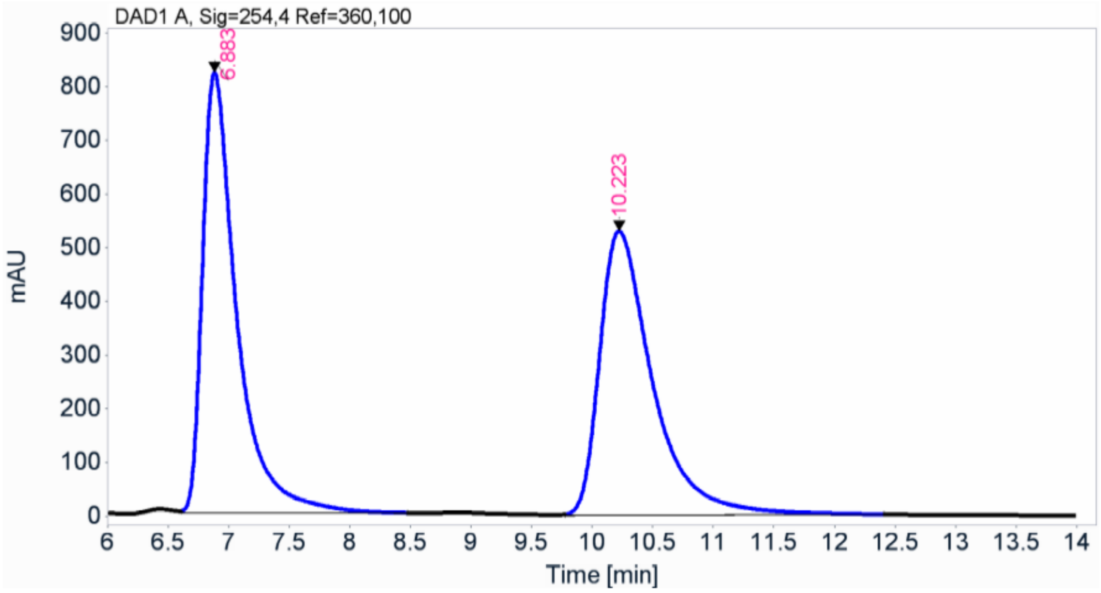

Signal:	DAD1 A, Sig=254,4 Ref=360,100				
**RT [min]**	**Type**	**Width [min]**	**Area**	**Height**	**Area%**
6.883	MM	0.3267	16077.415	820.2599	49.86
10.223	MM	0.5079	16168.633	530.5683	50.14

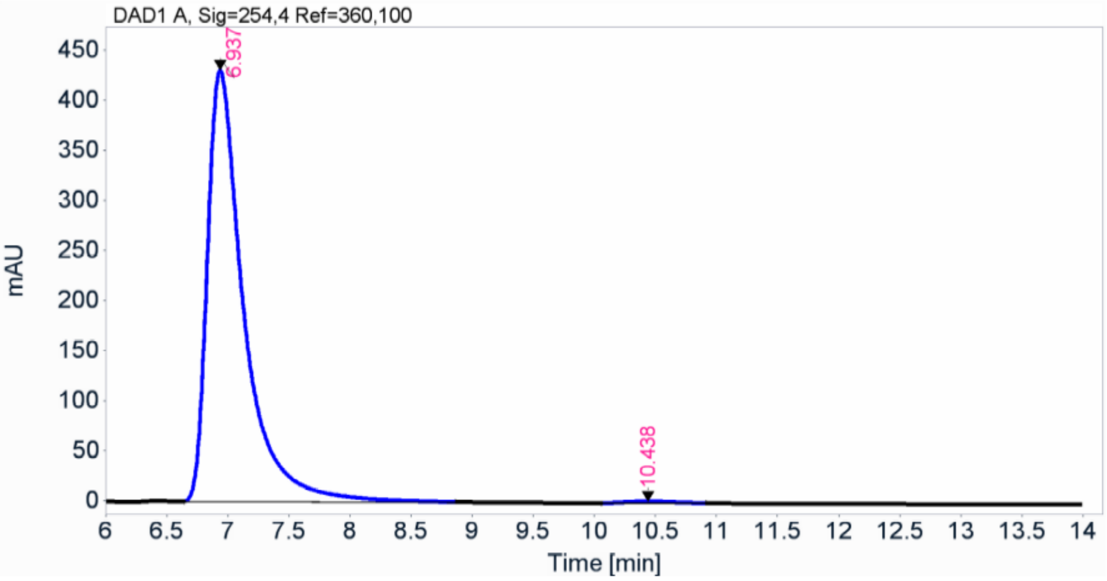

Signal:	DAD1 A, Sig=254,4 Ref=360,100				
**RT [min]**	**Type**	**Width [min]**	**Area**	**Height**	**Area%**
6.937	MM	0.3461	8951.959	431.1123	99.41
10.438	MM	0.4497	53.148	1.9698	0.59

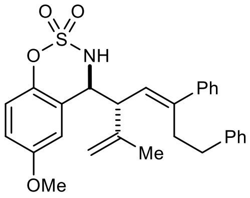

3c

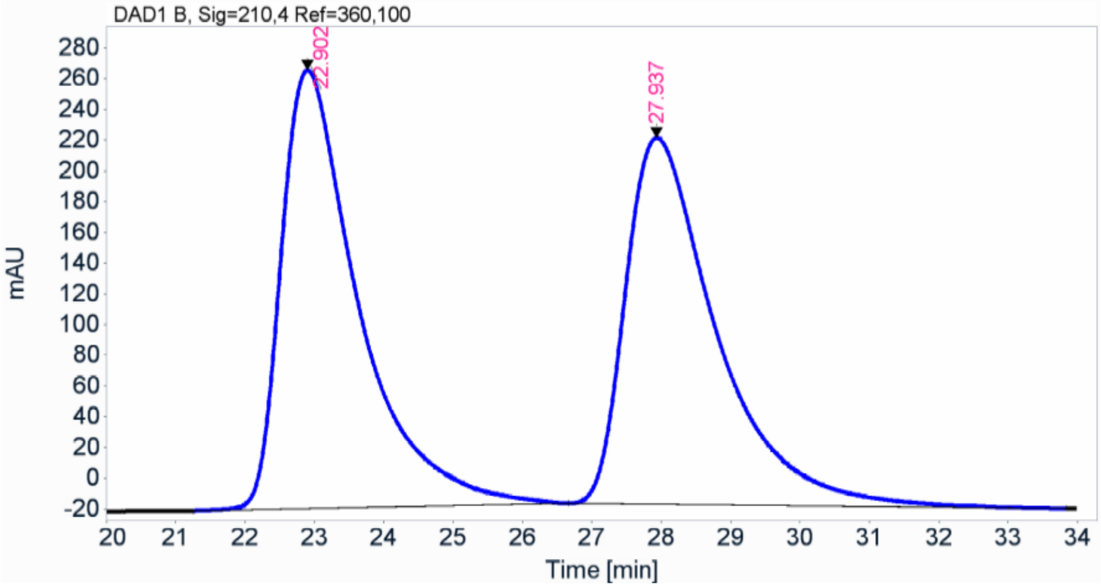

Signal: DAD1 B, Sig=210,4 Ref=360,100

RT [min]	Type	Width [min]	Area	Height	Area%
22.902	BB	1.1380	22051.520	285.2167	50.32
27.937	BB	1.3561	21767.307	238.6632	49.68

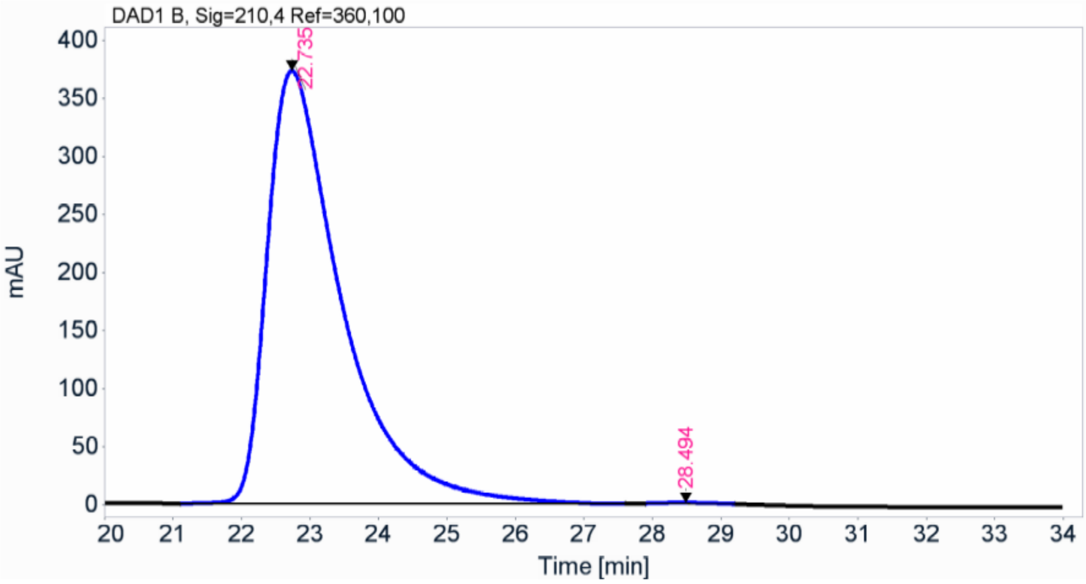

Signal: DAD1 B, Sig=210,4 Ref=360,100

RT [min]	Type	Width [min]	Area	Height	Area%
22.735	BB	1.1205	28191.561	372.6997	99.82
28.494	MM	0.7862	50.252	1.0652	0.18

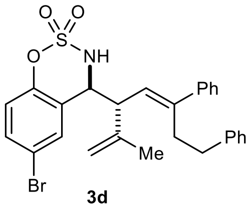

3d

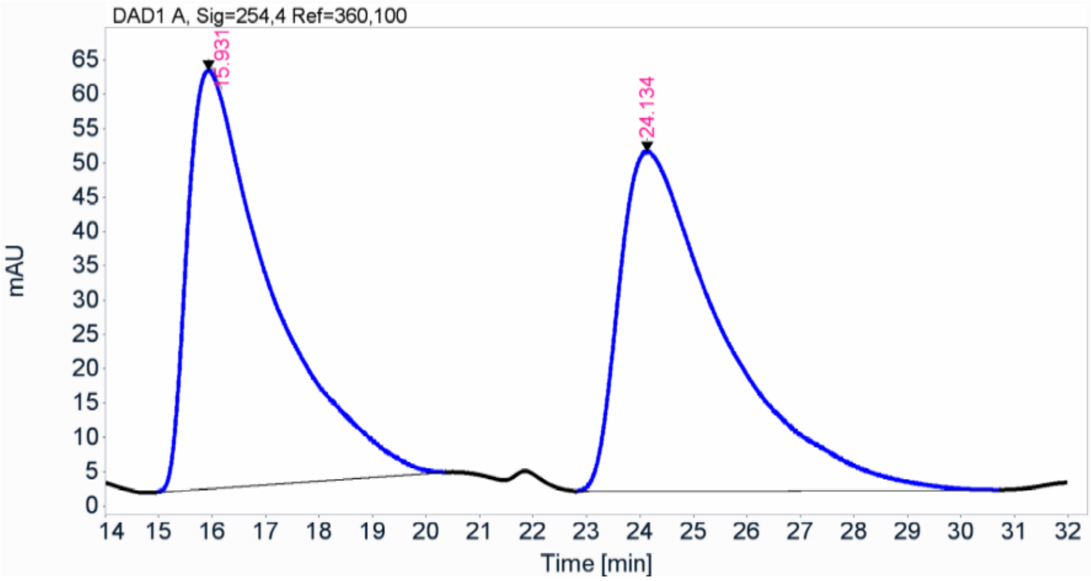

Signal:	DAD1 A, Sig=254,4 Ref=360,100				
RT [min]	Type	Width [min]	Area	Height	Area%
15.931	BB	1.5387	6588.873	60.8775	49.62
24.134	BB	1.9055	6688.719	49.4041	50.38

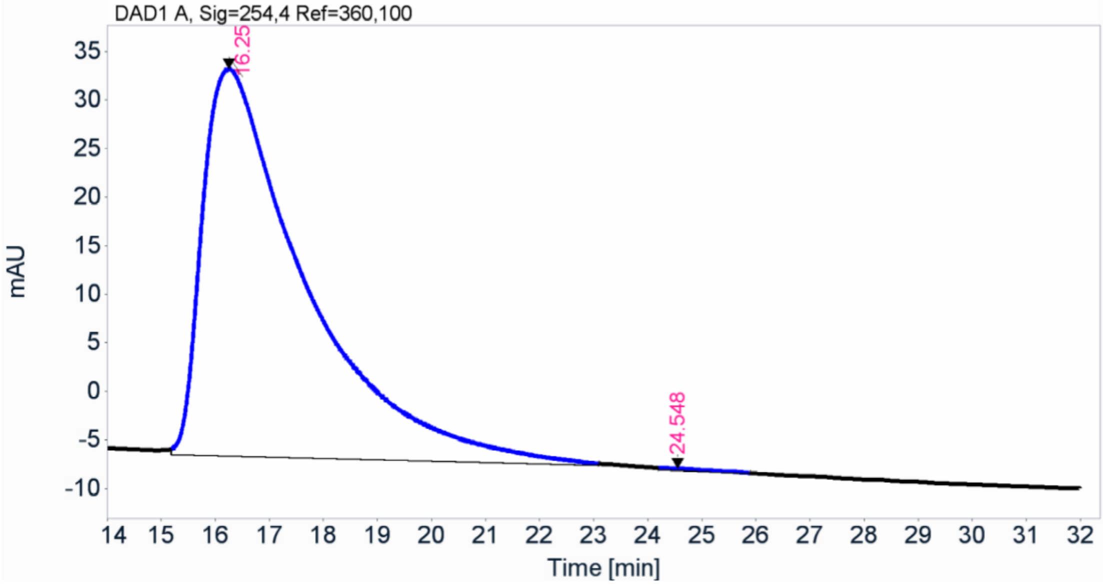

Signal:	DAD1 A, Sig=254,4 Ref=360,100				
RT [min]	Type	Width [min]	Area	Height	Area%
16.250	MM	2.2145	5294.606	39.8476	99.65
24.548	MM	1.2584	18.711	0.2478	0.35

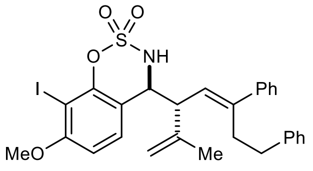

3e

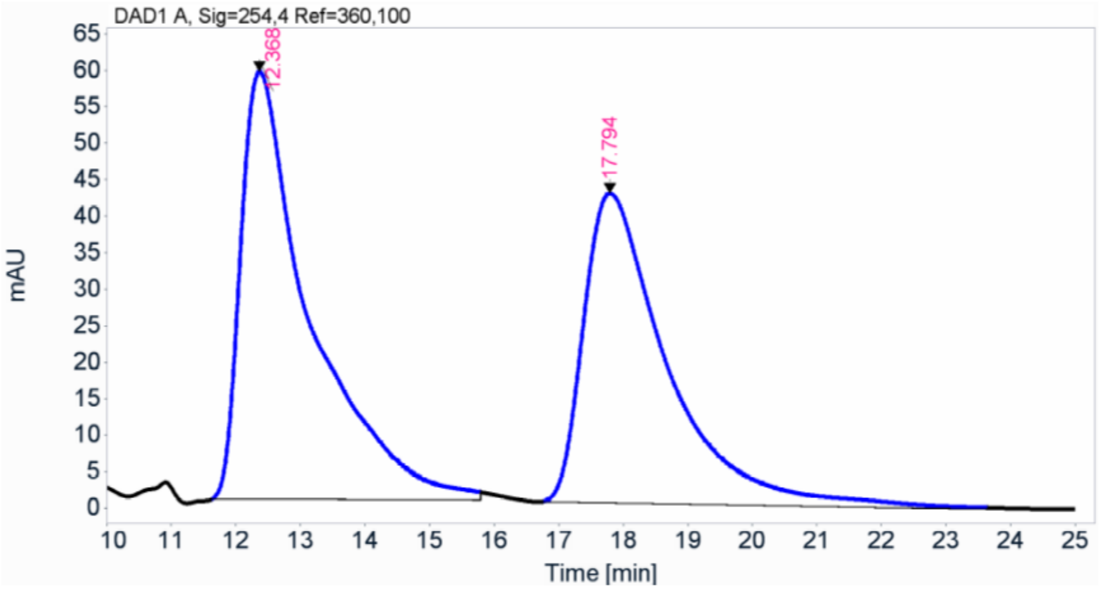

Signal:	DAD1 A, Sig=254,4 Ref=360,100				
**RT [min]**	**Type**	**Width [min]**	**Area**	**Height**	**Area%**
12.368	PM	1.2412	4363.138	58.5870	53.25
17.794	MM	1.5047	3830.029	42.4224	46.75

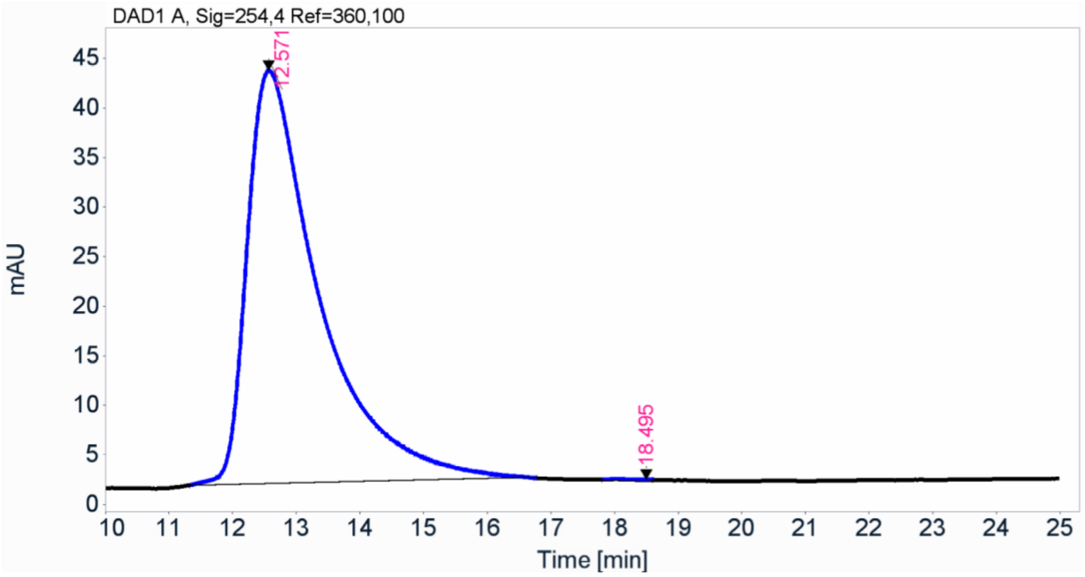

Signal:	DAD1 A, Sig=254,4 Ref=360,100				
**RT [min]**	**Type**	**Width [min]**	**Area**	**Height**	**Area%**
12.571	MM	1.3076	3266.577	41.6370	99.76
18.495	MM	0.6678	7.764	0.1938	0.24

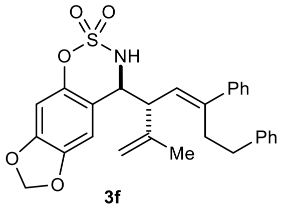

3f

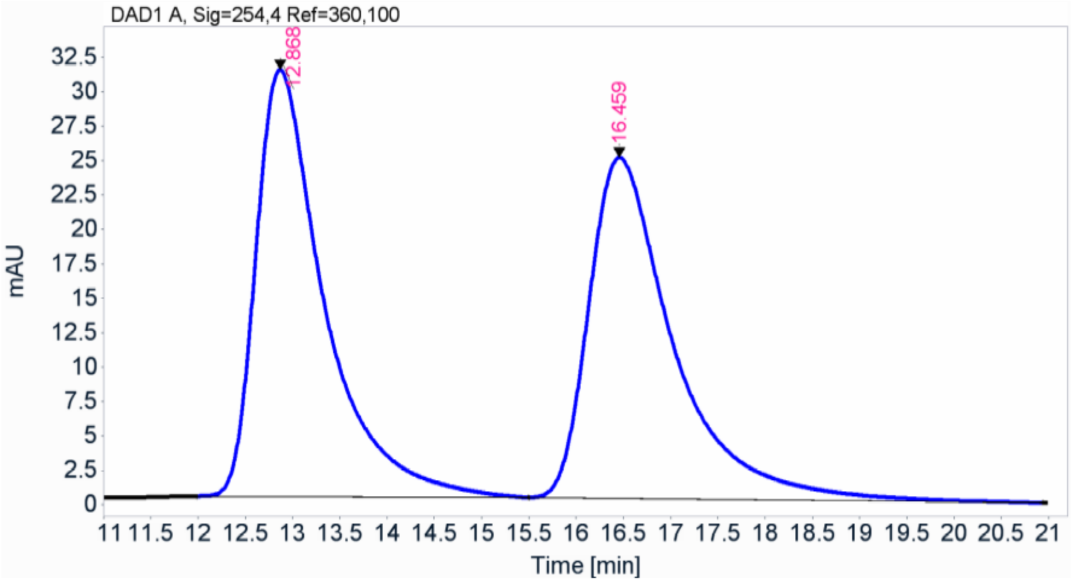

Signal: DAD1 A, Sig=254,4 Ref=360,100

RT [min]	Type	Width [min]	Area	Height	Area%
12.868	BB	0.7418	1556.134	30.9900	50.13
16.459	BB	0.9191	1548.326	24.7750	49.87

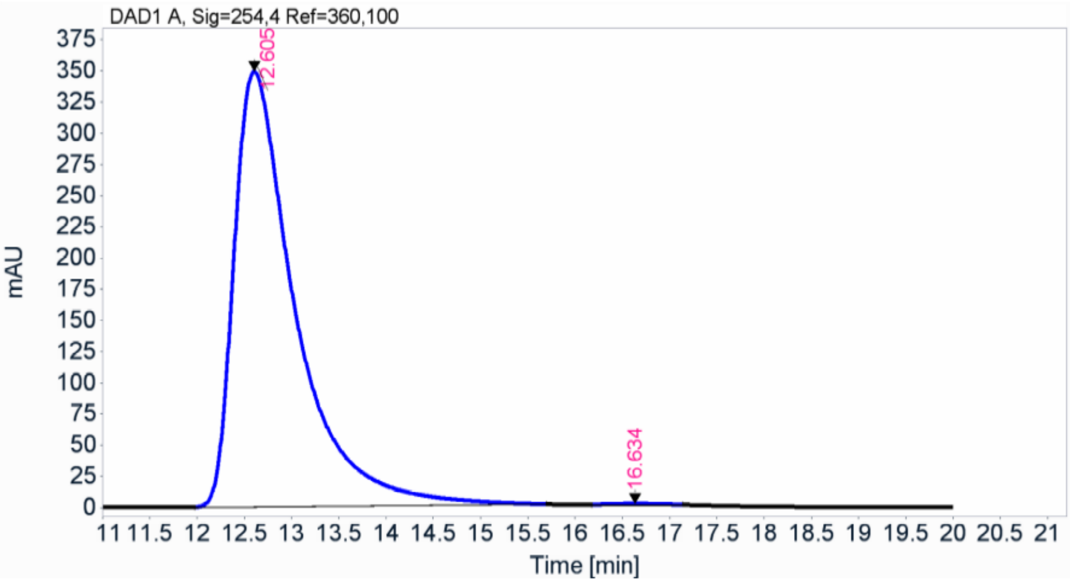

Signal: DAD1 A, Sig=254,4 Ref=360,100

RT [min]	Type	Width [min]	Area	Height	Area%
12.605	MM	0.7523	15763.051	349.2208	99.60
16.634	MM	0.7464	64.052	1.4303	0.40

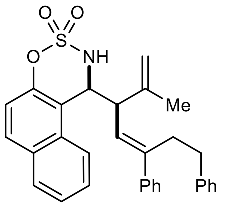

3g

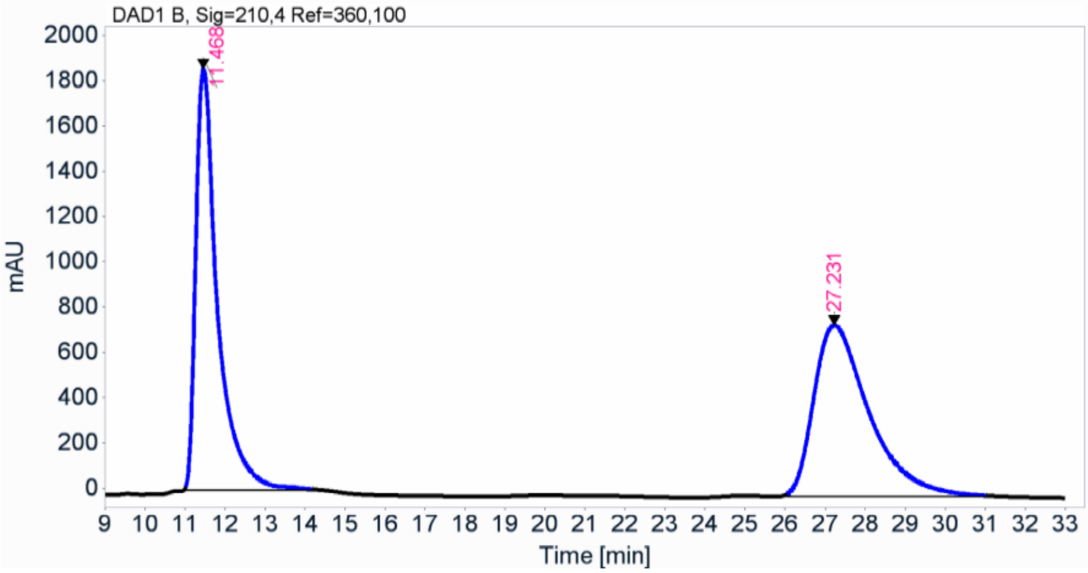

Signal:	DAD1 B, Sig=210,4 Ref=360,100				
RT [min]	Type	Width [min]	Area	Height	Area%
11.468	MM	0.6294	70169.500	1858.1954	49.18
27.231	MM	1.5996	72516.680	755.5559	50.82

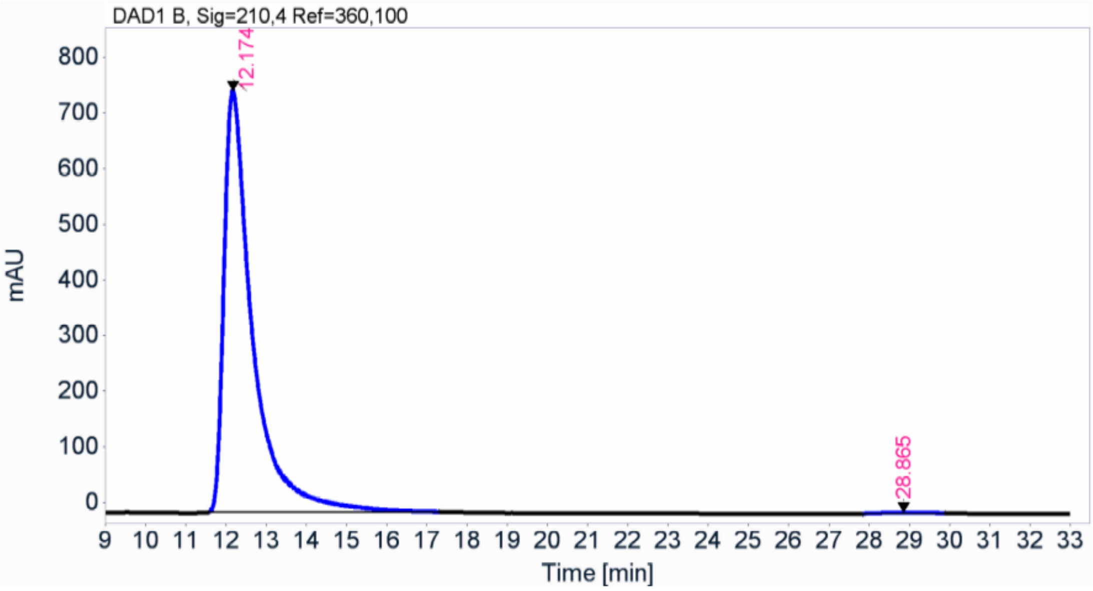

Signal:	DAD1 B, Sig=210,4 Ref=360,100				
RT [min]	Type	Width [min]	Area	Height	Area%
12.174	PM	0.8223	37311.711	756.2664	99.60
28.865	PM	1.2592	148.657	1.9677	0.40

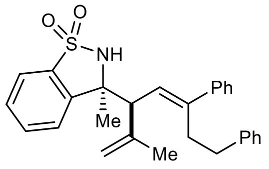

6

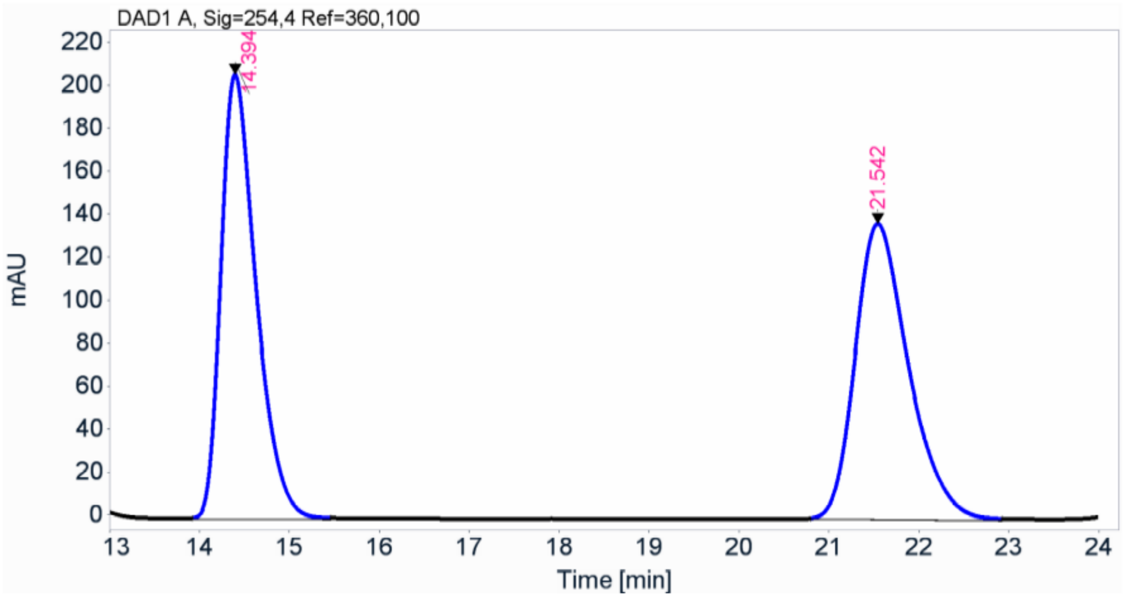

Signal:	DAD1 A, Sig=254,4 Ref=360,100				
RT [min]	Type	Width [min]	Area	Height	Area%
14.394	MM	0.4517	5607.477	206.8963	49.66
21.542	MM	0.6887	5685.180	137.5921	50.34

Before trituration

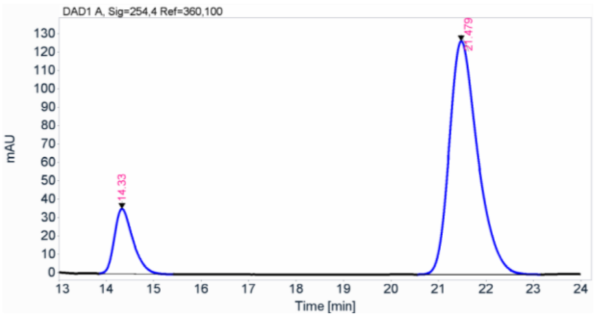

Signal:	DAD1 A, Sig=254,4 Ref=360,100				
RT [min]	Type	Width [min]	Area	Height	Area%
14.330	BB	0.4075	955.390	35.3273	15.40
21.479	BB	0.6268	5248.798	126.8342	84.60

After trituration

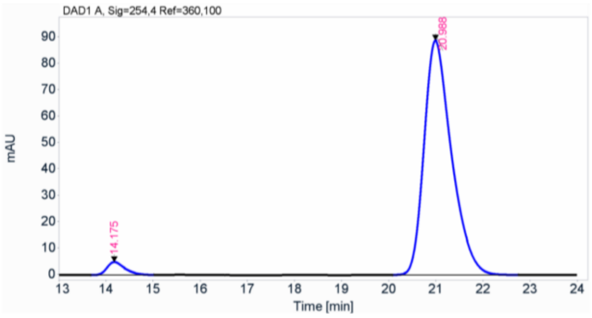

Signal:	DAD1 A, Sig=254,4 Ref=360,100				
RT [min]	Type	Width [min]	Area	Height	Area%
14.175	BB	0.3953	130.528	4.9560	3.51
20.988	BB	0.6087	3584.535	88.8519	96.49

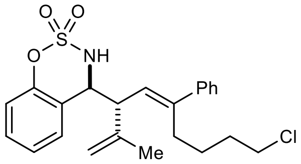

3h

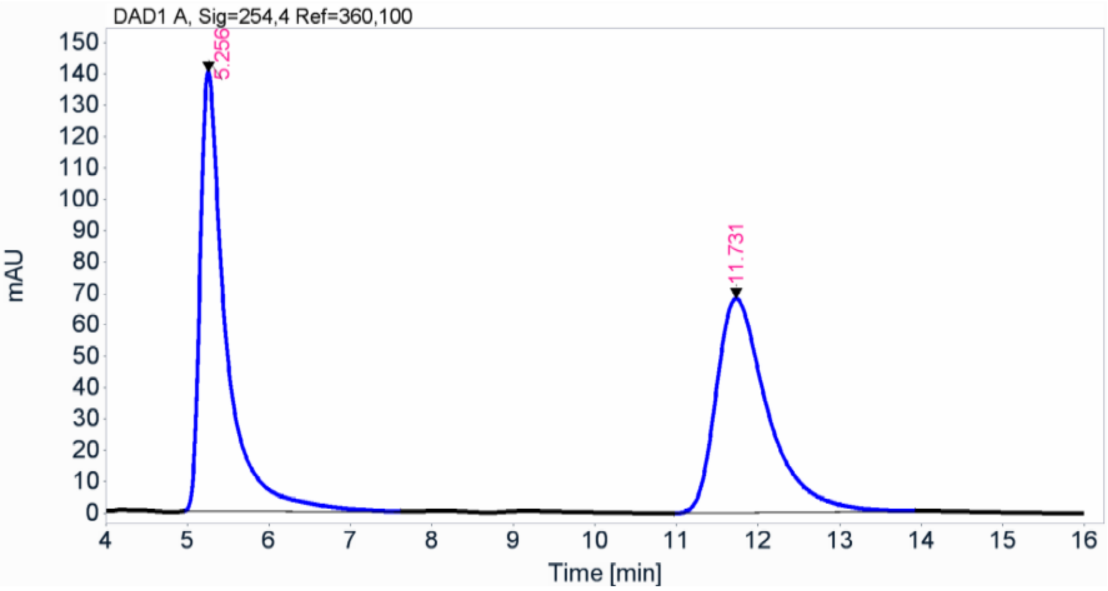

Signal:	DAD1 A, Sig=254,4 Ref=360,100				
**RT [min]**	**Type**	**Width [min]**	**Area**	**Height**	**Area%**
5.256	MM	0.3668	3082.023	140.0397	50.66
11.731	MM	0.7317	3001.171	68.3617	49.34

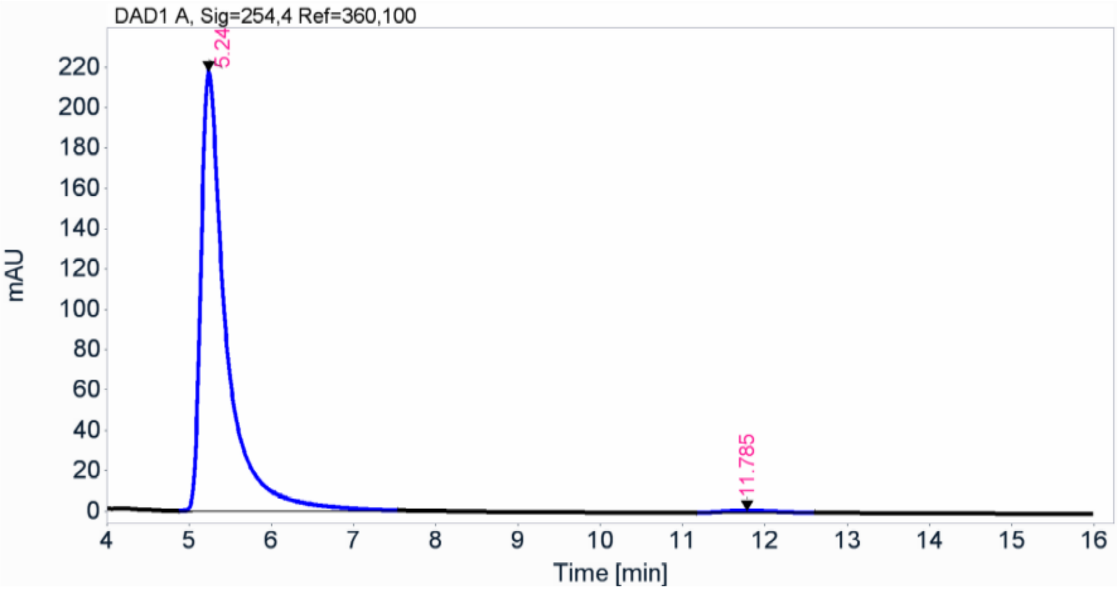

Signal:	DAD1 A, Sig=254,4 Ref=360,100				
**RT [min]**	**Type**	**Width [min]**	**Area**	**Height**	**Area%**
5.240	MM	0.3544	4629.639	217.7070	99.03
11.785	MM	0.7131	45.496	1.0633	0.97

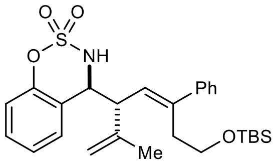

3i

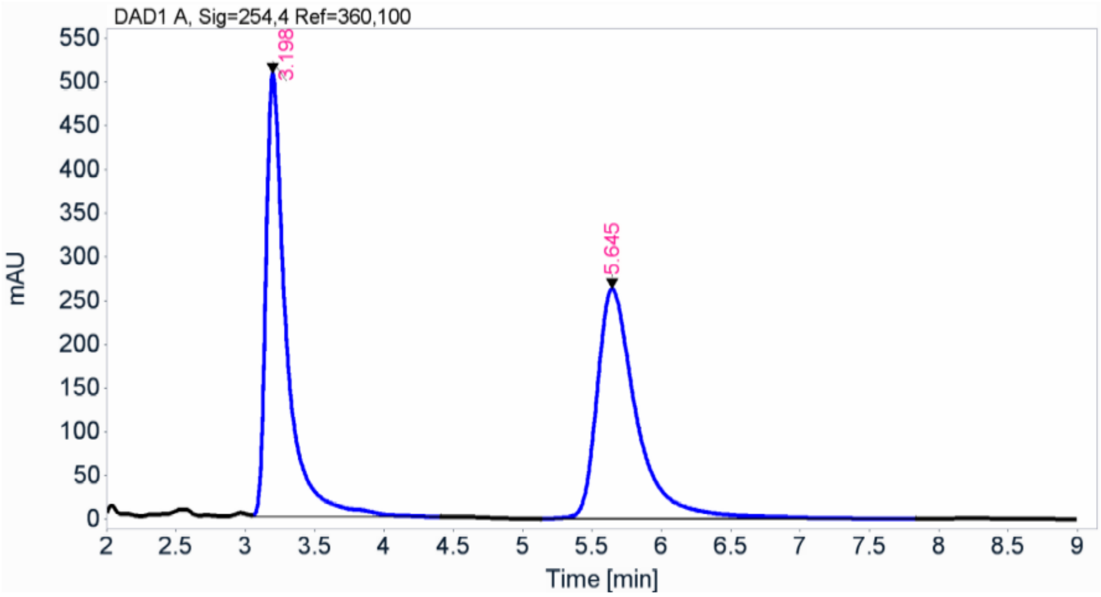

Signal:	DAD1 A, Sig=254,4 Ref=360,100				
RT [min]	Type	Width [min]	Area	Height	Area%
3.198	VB	0.1491	5175.195	507.0271	49.87
5.645	BB	0.2949	5202.046	262.8333	50.13

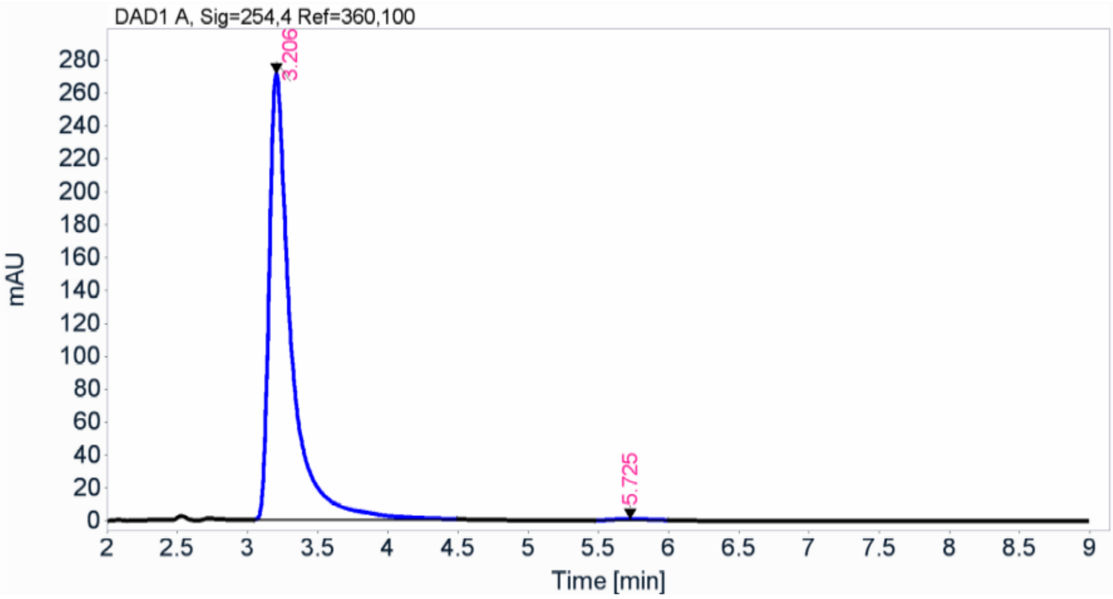

Signal:	DAD1 A, Sig=254,4 Ref=360,100				
RT [min]	Type	Width [min]	Area	Height	Area%
3.206	MM	0.1796	2924.771	271.3787	99.43
5.725	MM	0.3102	16.623	0.8932	0.57

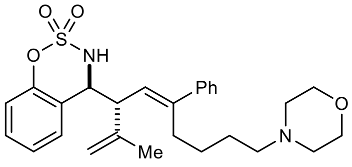

3j

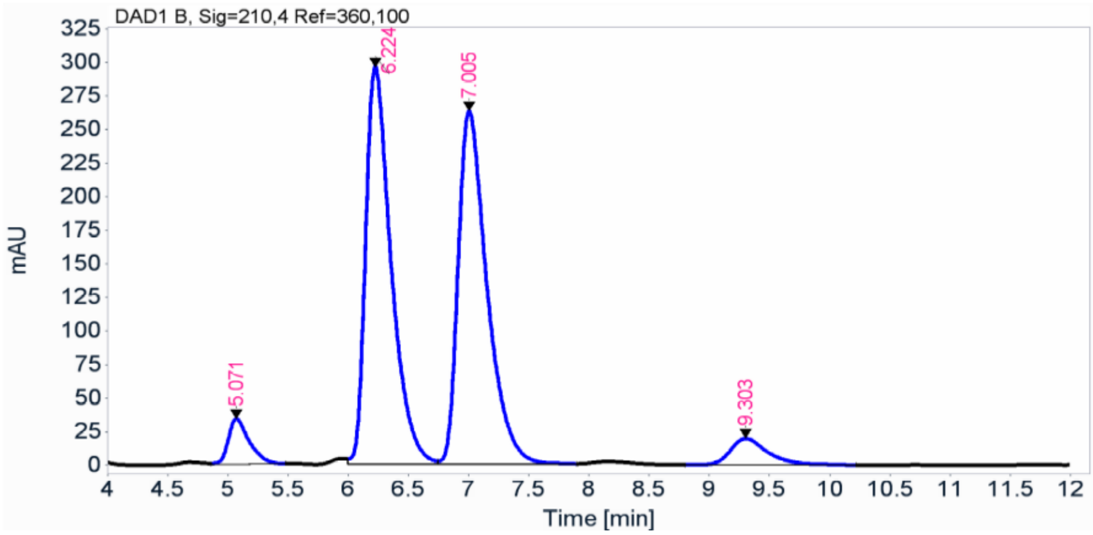

Signal:	DAD1 B, Sig=210,4 Ref=360,100				
RT [min]	Type	Width [min]	Area	Height	Area%
5.071	VB	0.1790	409.171	33.8446	4.28
6.224	VV	0.2213	4348.305	296.1090	45.51
7.005	VB	0.2498	4368.406	263.0285	45.72
9.303	BB	0.3237	428.724	19.7162	4.49

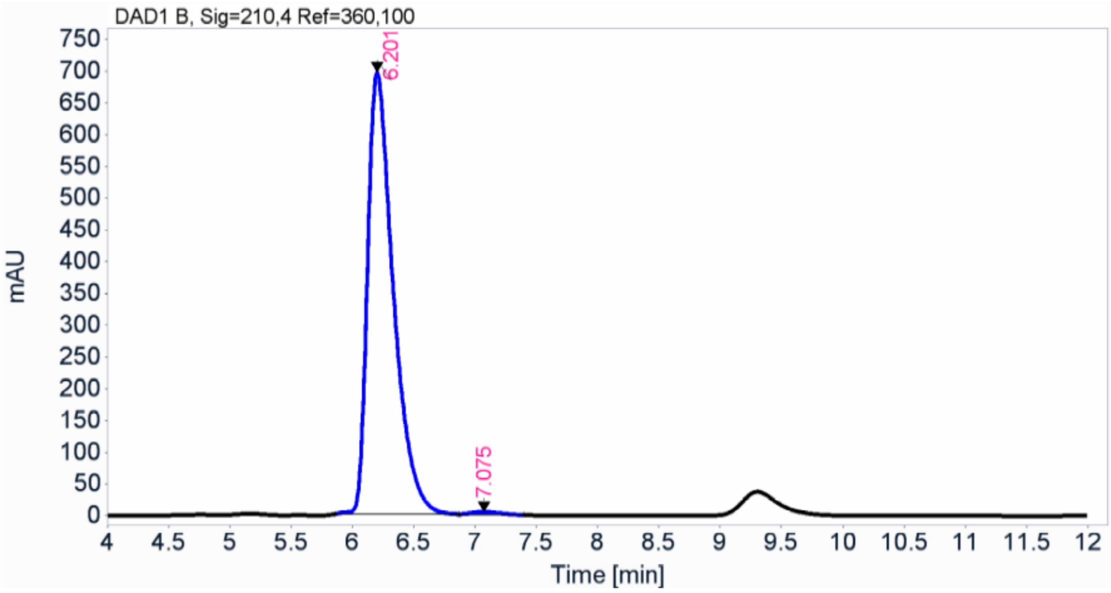

Signal:	DAD1 B, Sig=210,4 Ref=360,100				
RT [min]	Type	Width [min]	Area	Height	Area%
6.201	MM	0.2425	10116.198	695.2485	99.38
7.075	MM	0.2545	63.407	4.1527	0.62

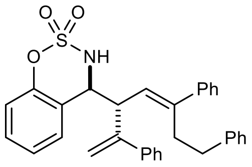

3k

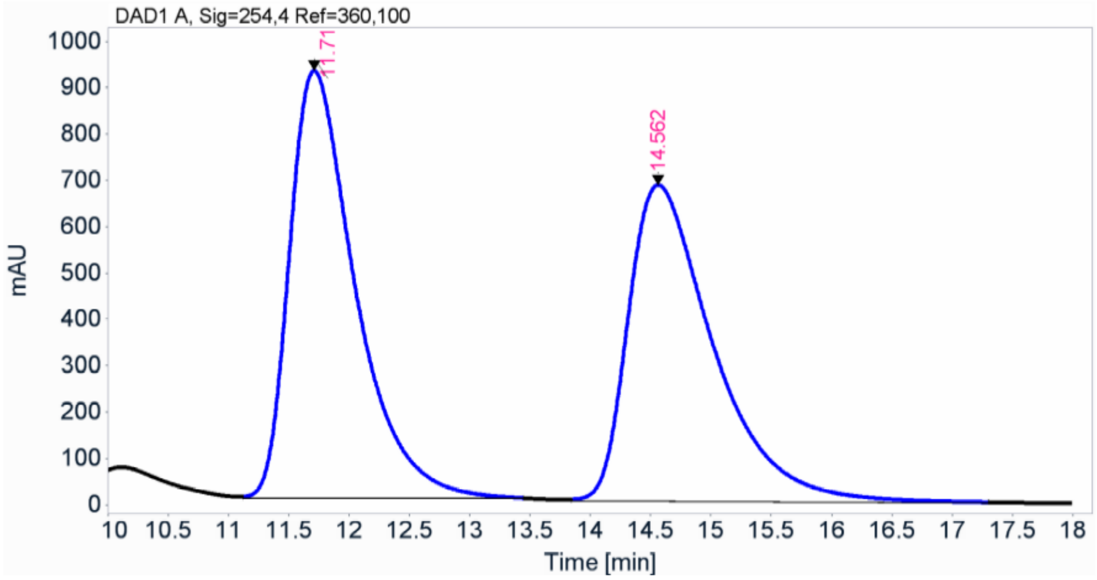

Signal:	DAD1 A, Sig=254,4 Ref=360,100				
**RT [min]**	**Type**	**Width [min]**	**Area**	**Height**	**Area%**
11.710	MP	0.6315	34973.977	923.0099	50.97
14.562	MM	0.8207	33648.758	683.3572	49.03

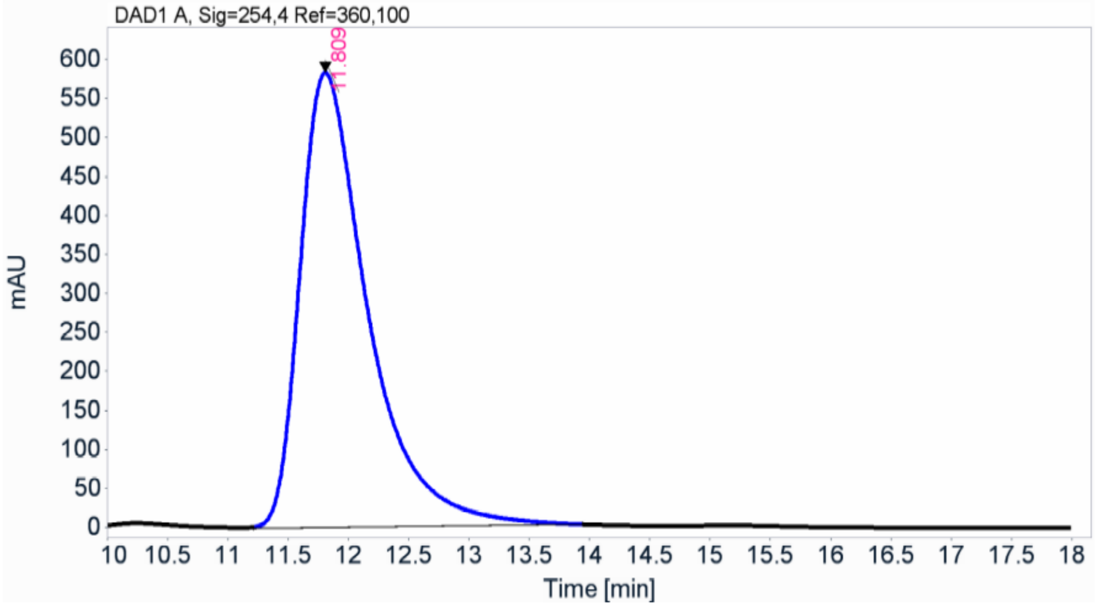

Signal:	DAD1 A, Sig=254,4 Ref=360,100				
**RT [min]**	**Type**	**Width [min]**	**Area**	**Height**	**Area%**
11.809	MM	0.6507	22775.510	583.3912	100.00

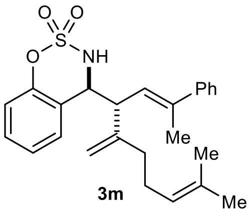

3m

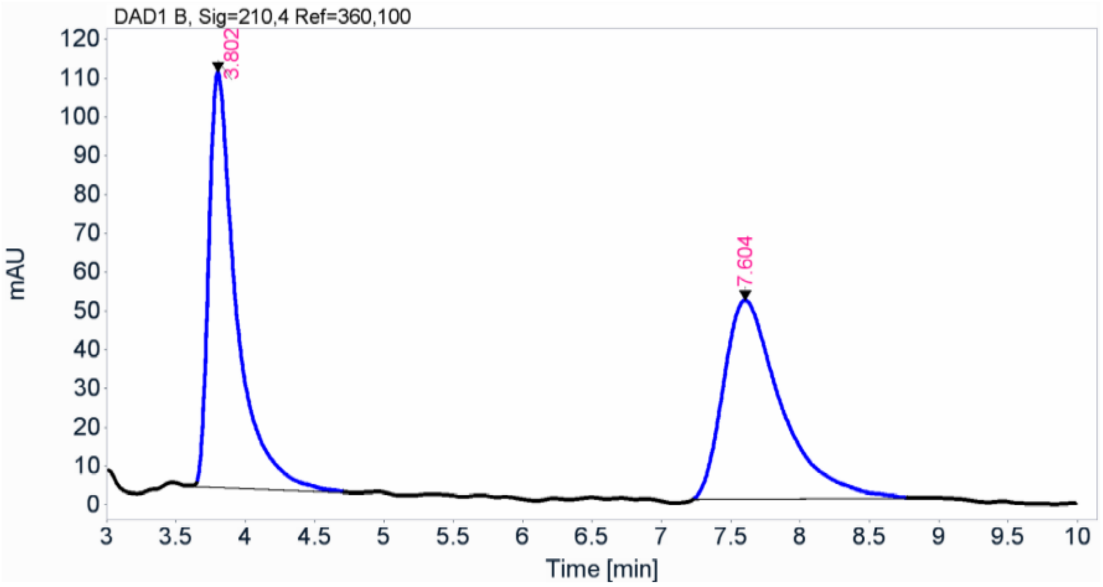

Signal:	DAD1 B, Sig=210,4 Ref=360,100				
**RT [min]**	**Type**	**Width [min]**	**Area**	**Height**	**Area%**
3.802	MM	0.2310	1485.679	107.1965	49.94
7.604	MM	0.4841	1489.064	51.2676	50.06

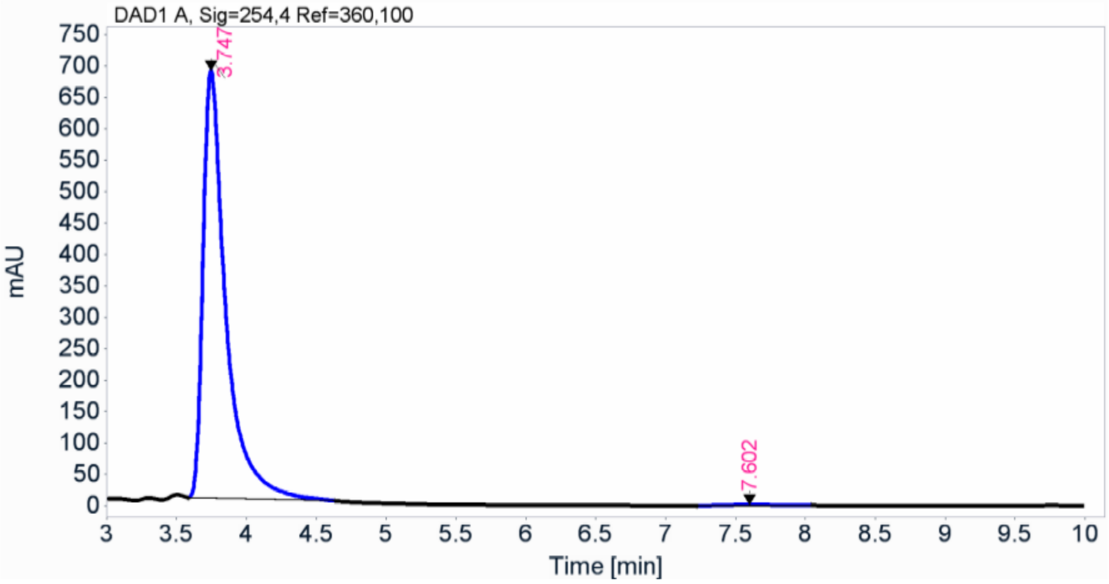

Signal:	DAD1 A, Sig=254,4 Ref=360,100				
**RT [min]**	**Type**	**Width [min]**	**Area**	**Height**	**Area%**
3.747	MM	0.1891	7733.747	681.5695	99.46
7.602	MM	0.4548	41.846	1.5335	0.54

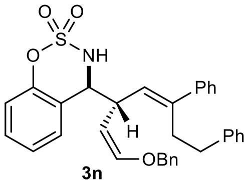

3n

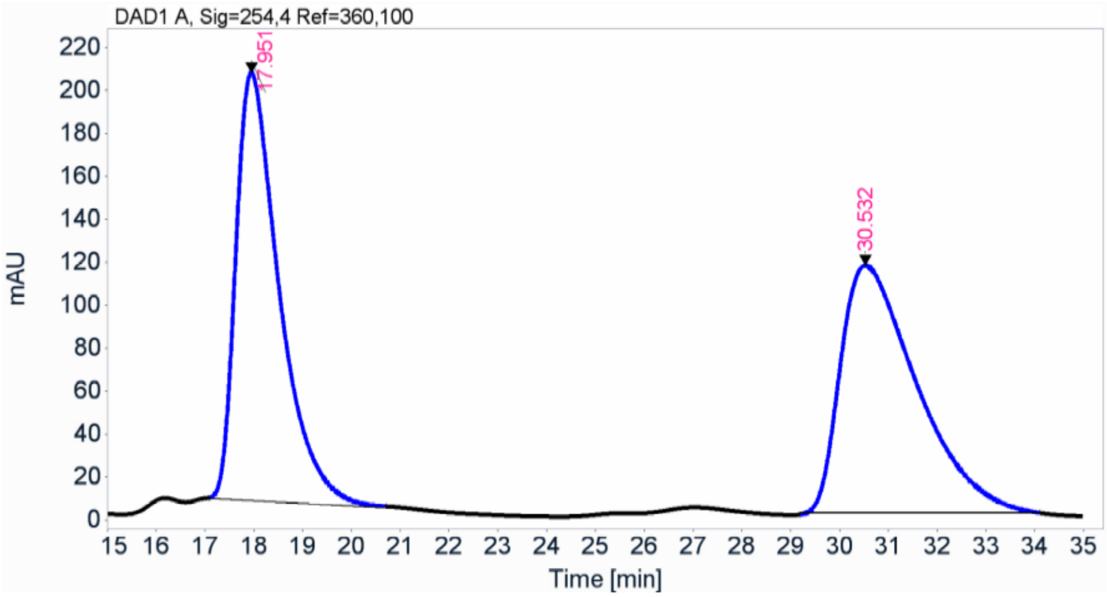

Signal: DAD1 A, Sig=254,4 Ref=360,100

RT [min]	Type	Width [min]	Area	Height	Area%
17.951	MM	1.0274	12271.299	199.0669	49.53
30.532	MM	1.8062	12502.054	115.3619	50.47

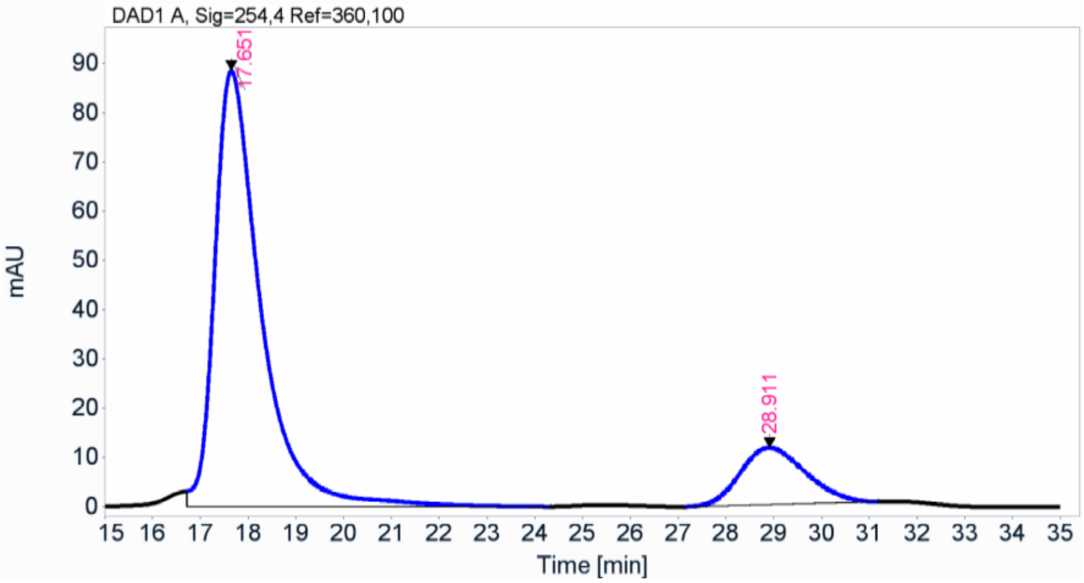

Signal: DAD1 A, Sig=254,4 Ref=360,100

RT [min]	Type	Width [min]	Area	Height	Area%
17.651	FM	1.1146	5916.652	88.4745	84.40
28.911	BB	1.3629	1093.665	11.5482	15.60

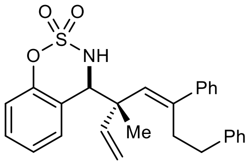

3o

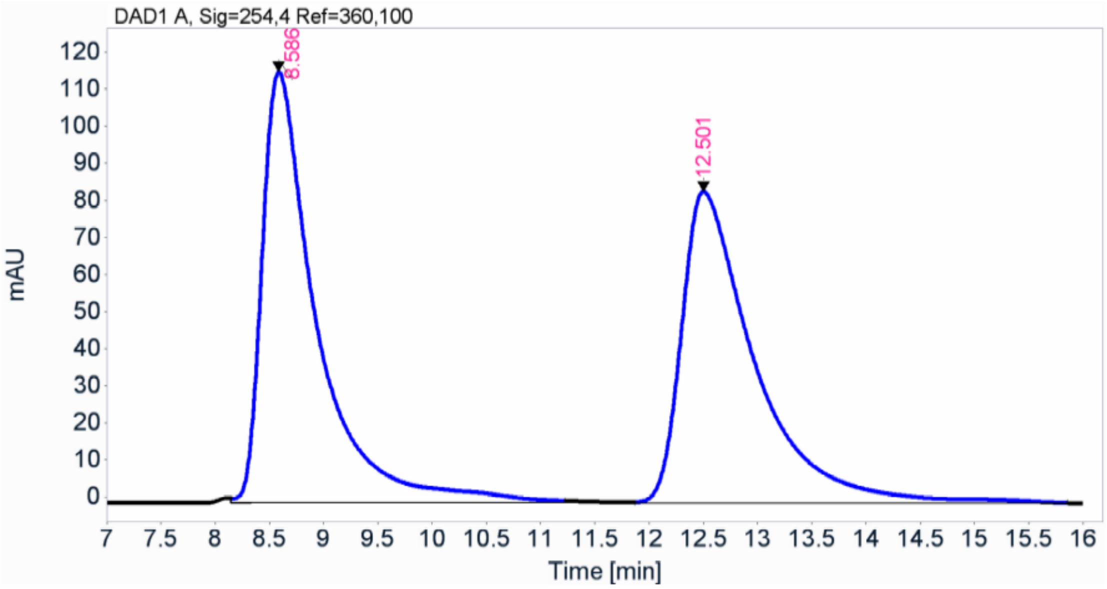

Signal:	DAD1 A, Sig=254,4 Ref=360,100				
**RT [min]**	**Type**	**Width [min]**	**Area**	**Height**	**Area%**
8.586	MM	0.5805	4047.693	116.2143	50.56
12.501	MM	0.7849	3958.318	84.0499	49.44

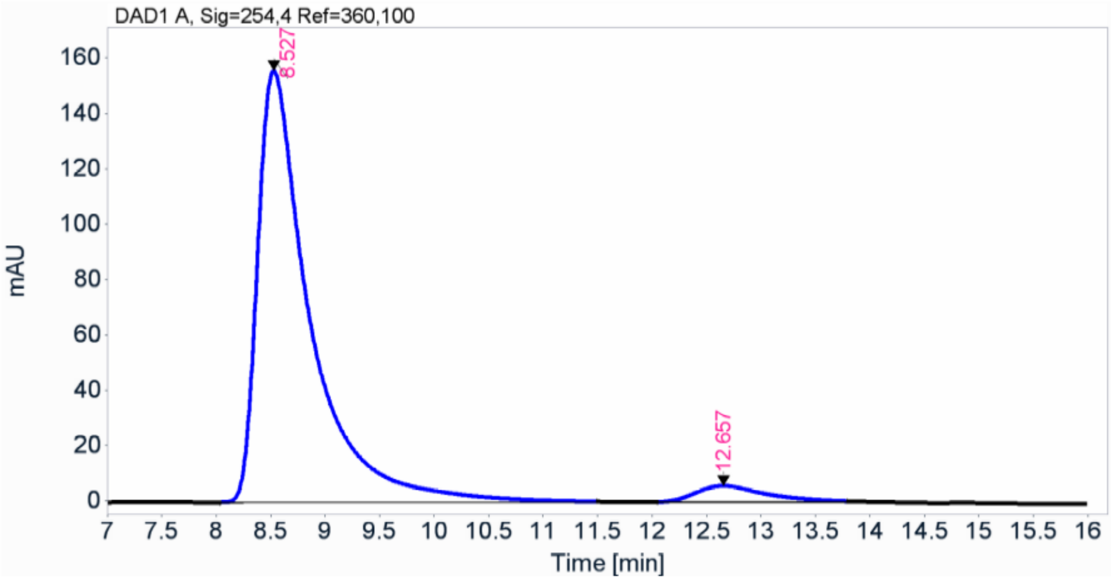

Signal:	DAD1 A, Sig=254,4 Ref=360,100				
**RT [min]**	**Type**	**Width [min]**	**Area**	**Height**	**Area%**
8.527	MM	0.5597	5236.211	155.9333	95.17
12.657	MM	0.7426	265.738	5.9639	4.83

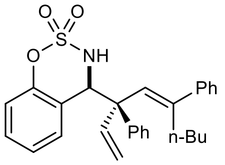

3p

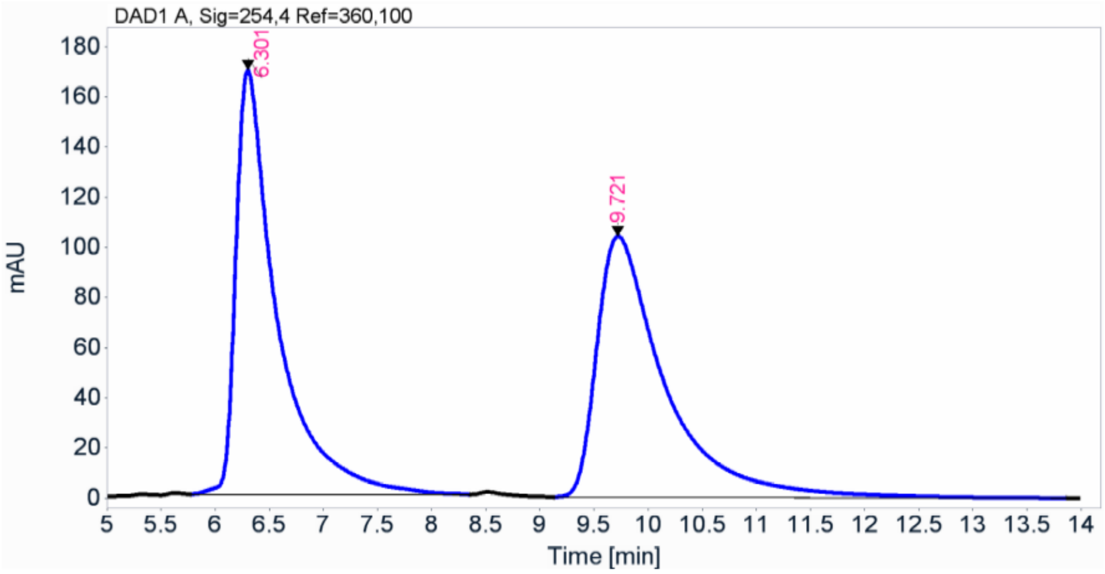

Signal:	DAD1 A, Sig=254,4 Ref=360,100				
RT [min]	Type	Width [min]	Area	Height	Area%
6.301	BB	0.3757	4486.752	169.0216	49.68
9.721	BB	0.6362	4545.168	103.9391	50.32

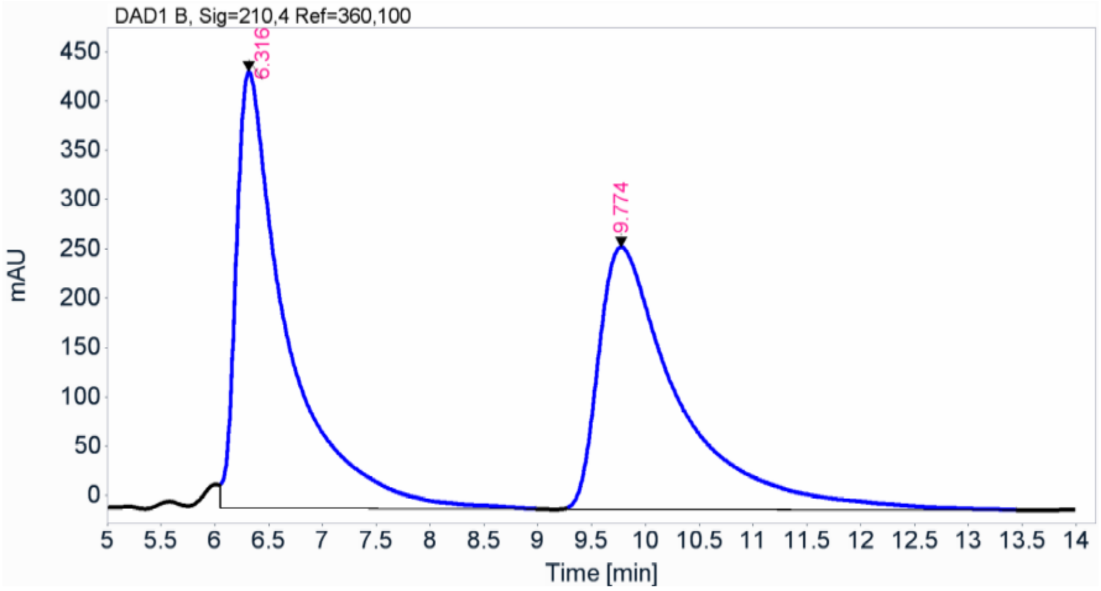

Signal:	DAD1 B, Sig=210,4 Ref=360,100				
RT [min]	Type	Width [min]	Area	Height	Area%
6.316	FM	0.5443	14444.783	442.3214	51.20
9.774	MM	0.8637	13768.820	265.7025	48.80

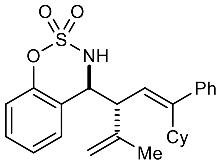

(*E*)-**3q**

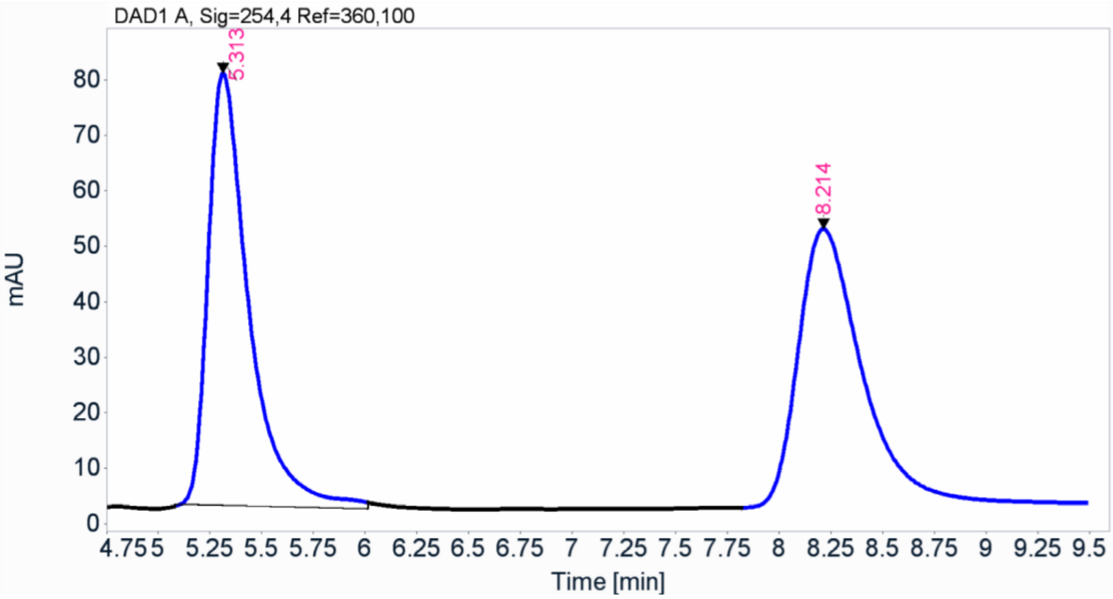

Signal: DAD1 A, Sig=254,4 Ref=360,100

RT [min]	Type	Width [min]	Area	Height	Area%
5.313	MM	0.2288	1069.974	77.9455	49.76
8.214	BB	0.3259	1080.485	50.0349	50.24

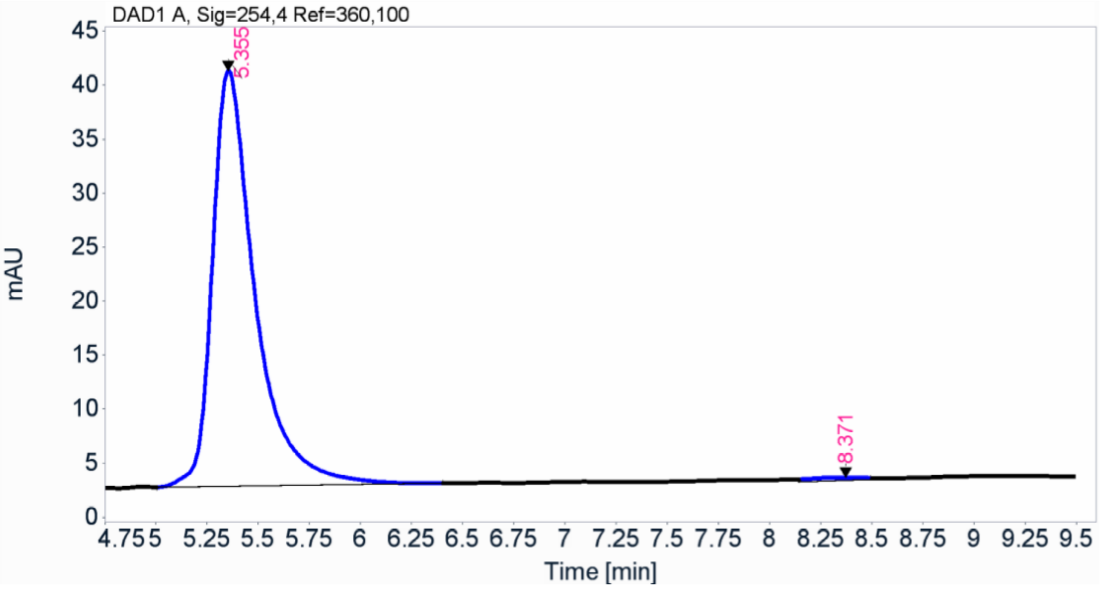

Signal: DAD1 A, Sig=254,4 Ref=360,100

RT [min]	Type	Width [min]	Area	Height	Area%
5.355	BB	0.2132	558.257	38.4866	99.09
8.371	MM	0.3263	5.124	0.2617	0.91

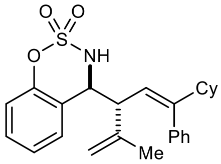

(*Z*)-**3q**

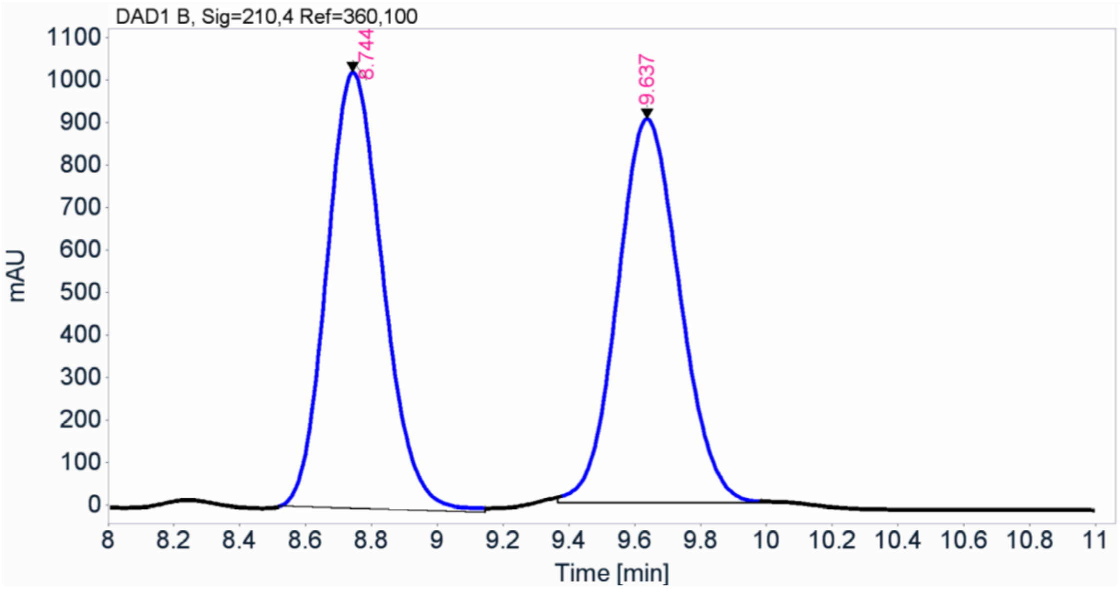

Signal: DAD1 B, Sig=210,4 Ref=360,100

RT [min]	Type	Width [min]	Area	Height	Area%
8.744	MM	0.1969	12120.783	1026.2179	50.49
9.637	MM	0.2192	11885.754	903.9160	49.51

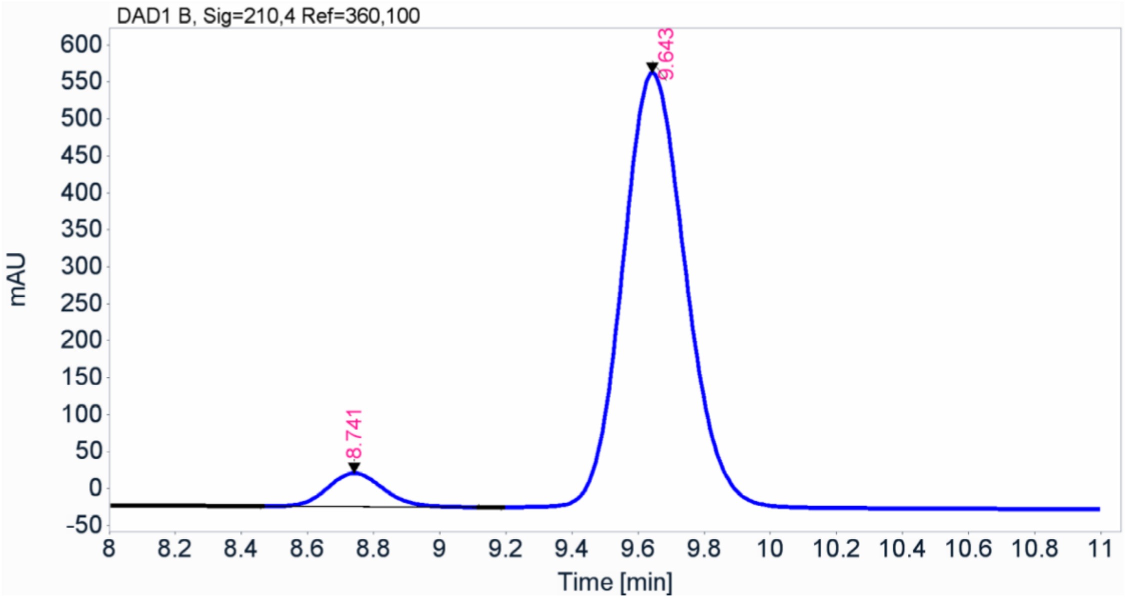

Signal: DAD1 B, Sig=210,4 Ref=360,100

RT [min]	Type	Width [min]	Area	Height	Area%
8.741	BB	0.1805	533.473	45.5823	6.38
9.643	BB	0.2072	7833.008	588.7488	93.62

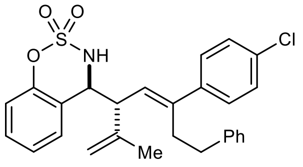

3r

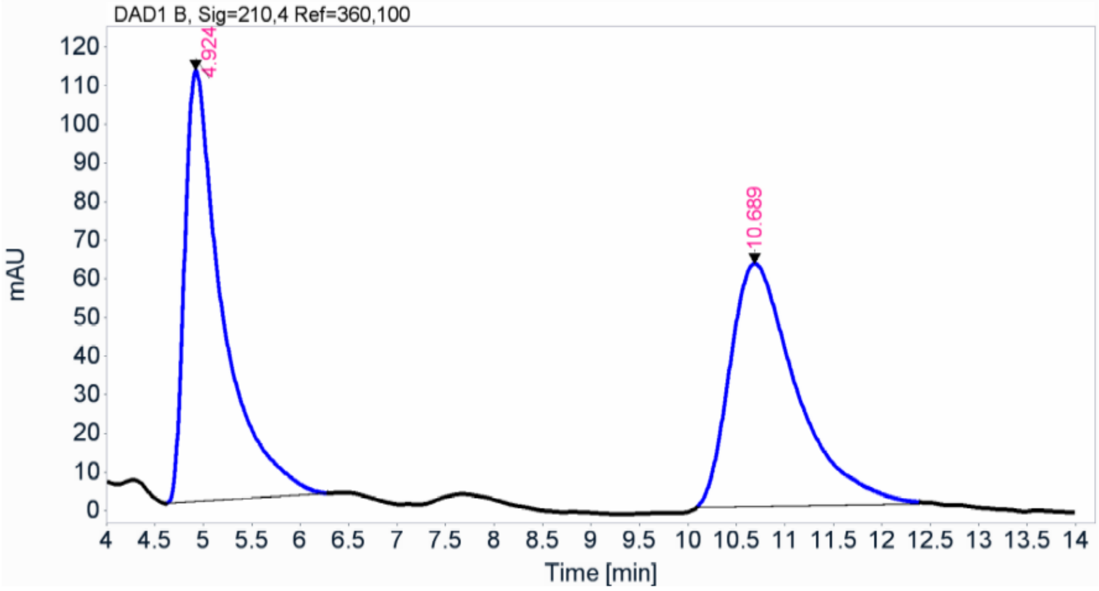

Signal:	DAD1 B, Sig=210,4 Ref=360,100				
RT [min]	Type	Width [min]	Area	Height	Area%
4.924	BB	0.3869	3032.641	111.6421	50.24
10.689	MM	0.7960	3003.804	62.8964	49.76

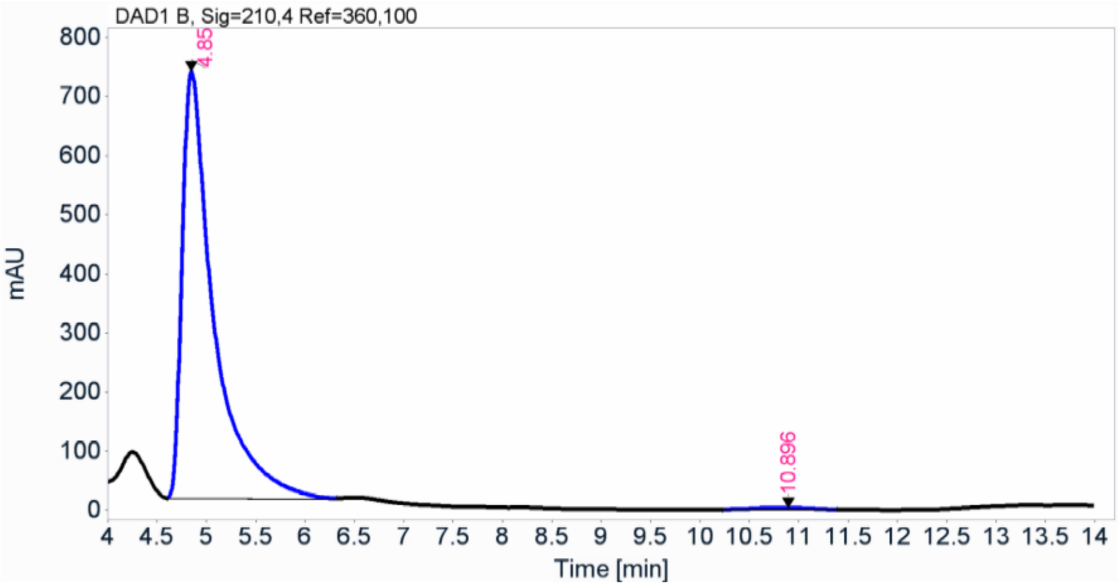

Signal:	DAD1 B, Sig=210,4 Ref=360,100				
RT [min]	Type	Width [min]	Area	Height	Area%
4.850	MM	0.3724	16151.250	722.7874	99.07
10.896	MM	0.6917	151.727	3.6560	0.93

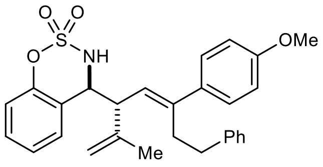

3s

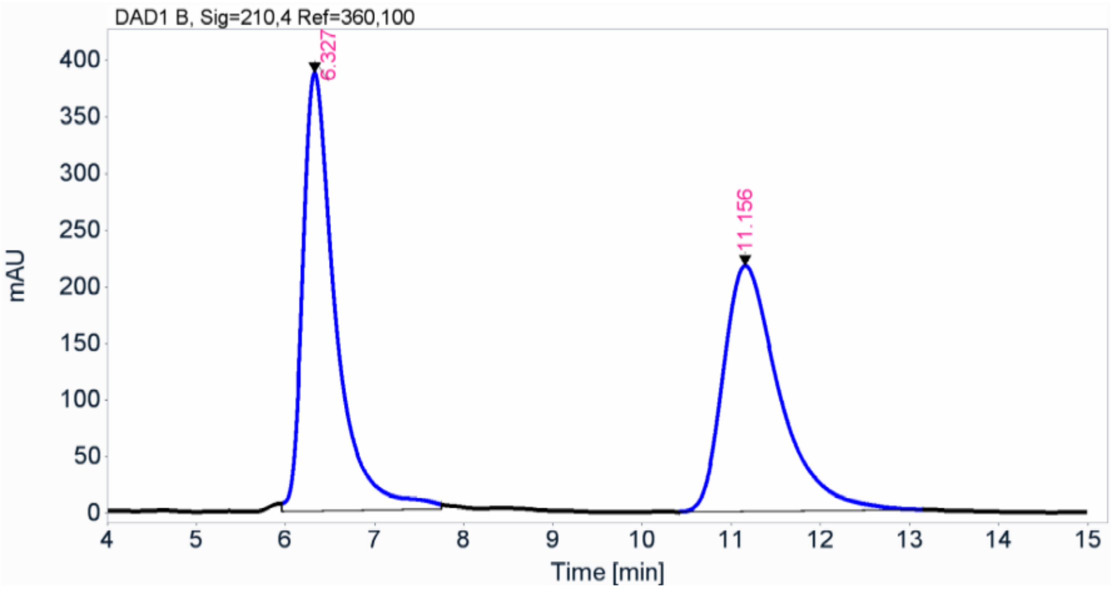

Signal: DAD1 B, Sig=210,4 Ref=360,100

RT [min]	Type	Width [min]	Area	Height	Area%
6.327	MF	0.4233	9811.697	386.3154	50.95
11.156	BB	0.6609	9447.290	216.5872	49.05

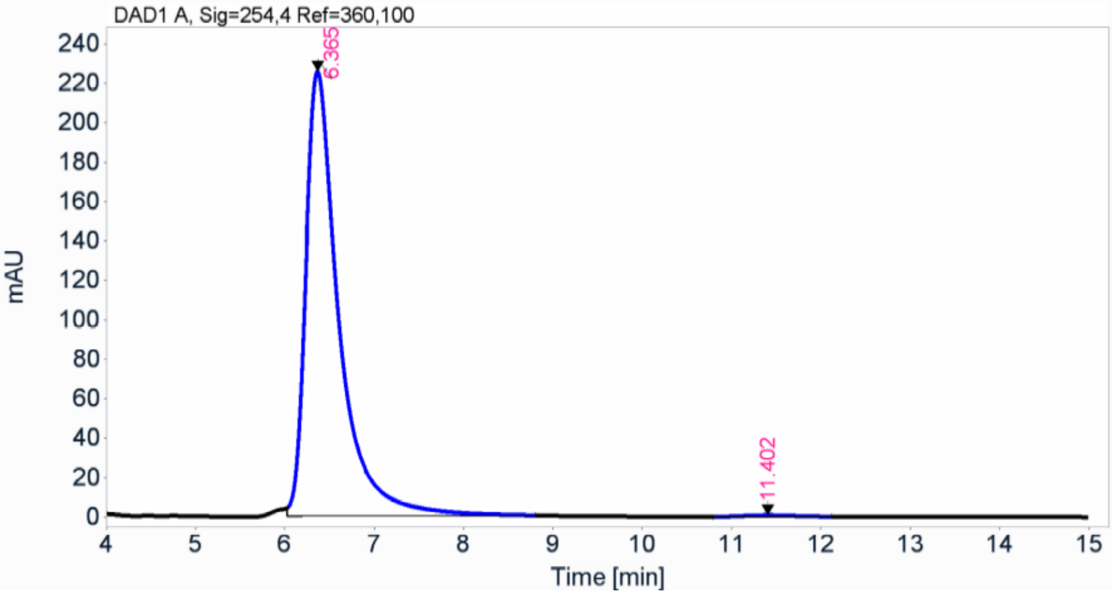

Signal: DAD1 A, Sig=254,4 Ref=360,100

RT [min]	Type	Width [min]	Area	Height	Area%
6.365	FM	0.4304	5823.984	225.5473	99.47
11.402	MM	0.6646	30.971	0.7767	0.53

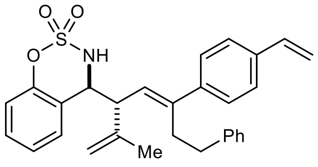

3t

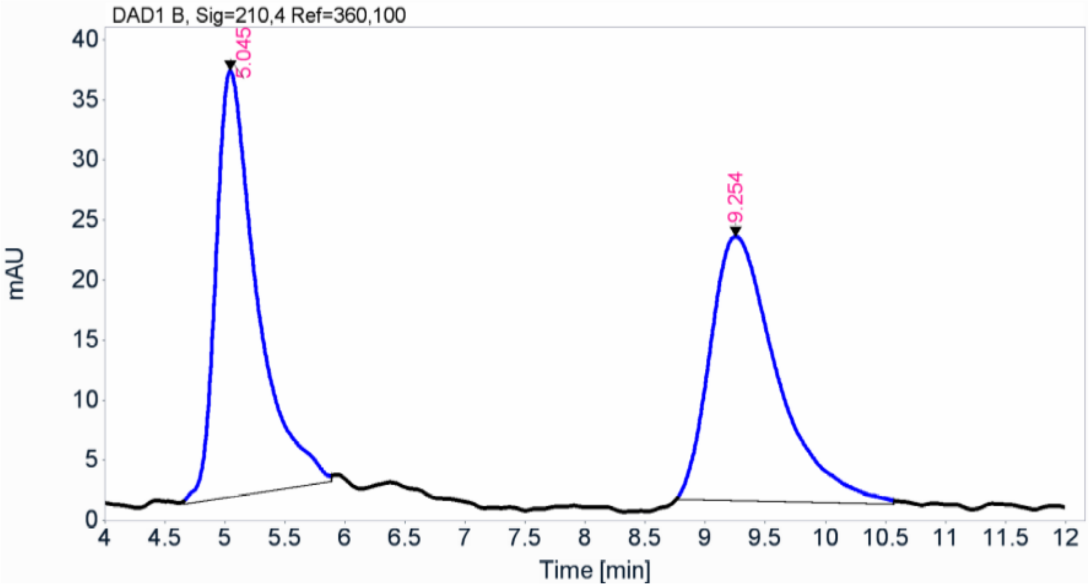

Signal:	DAD1 B, Sig=210,4 Ref=360,100				
RT [min]	Type	Width [min]	Area	Height	Area%
5.045	MM	0.3971	845.354	35.4769	49.78
9.254	MM	0.6463	852.822	21.9920	50.22

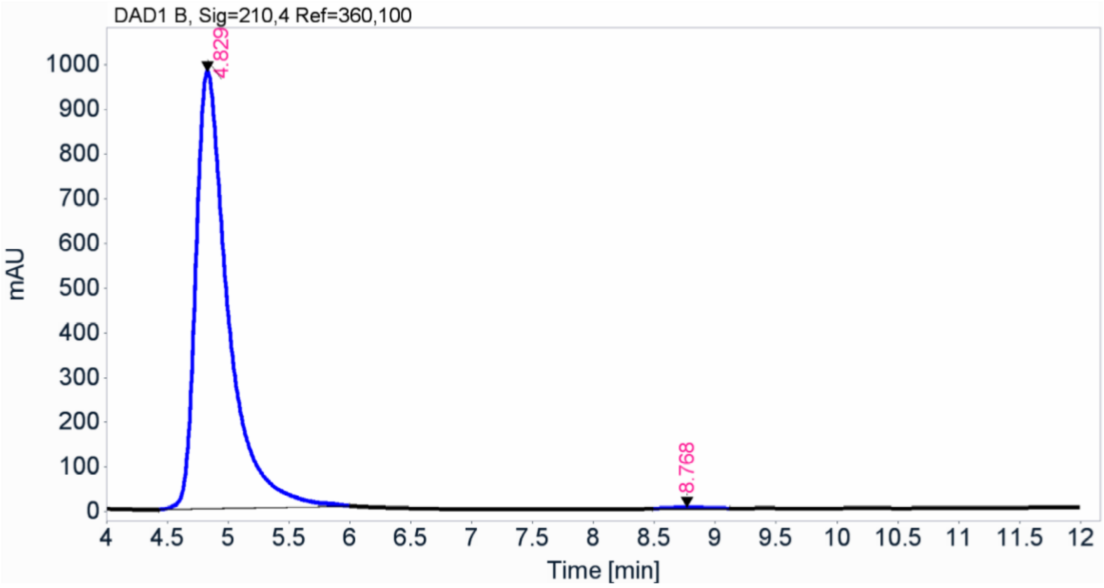

Signal:	DAD1 B, Sig=210,4 Ref=360,100				
RT [min]	Type	Width [min]	Area	Height	Area%
4.829	MP	0.3074	18053.977	978.9537	99.36
8.768	MM	0.4838	116.457	4.0119	0.64

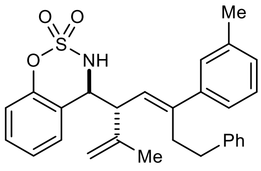

3u

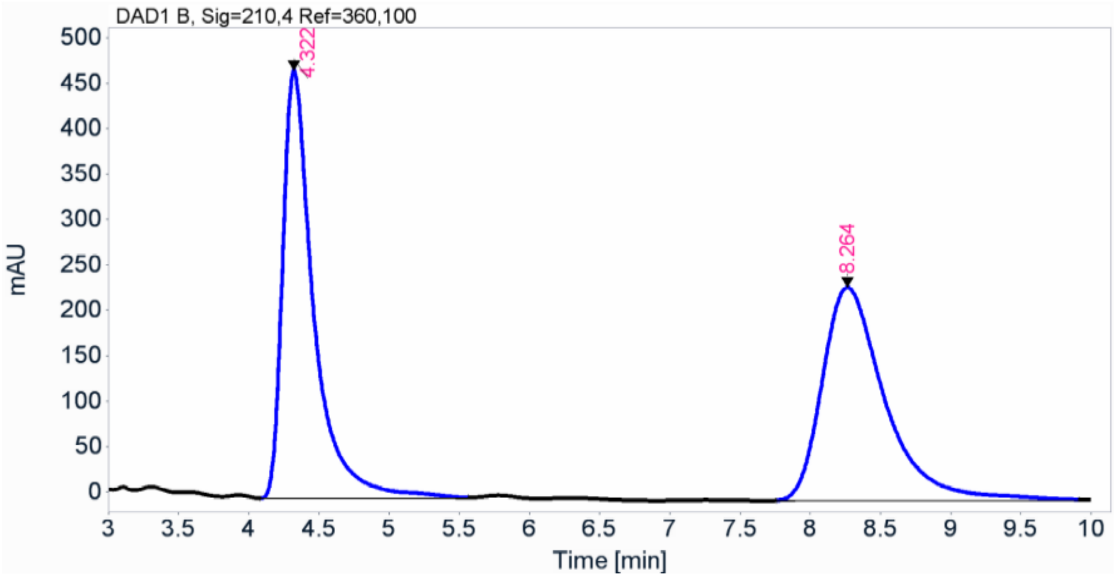

**Signal:**	DAD1 B, Sig=210,4 Ref=360,100				
**RT [min]**	**Type**	**Width [min]**	**Area**	**Height**	**Area%**
4.322	BV	0.2281	7274.634	471.2281	49.77
8.264	MM	0.5199	7341.445	235.3517	50.23

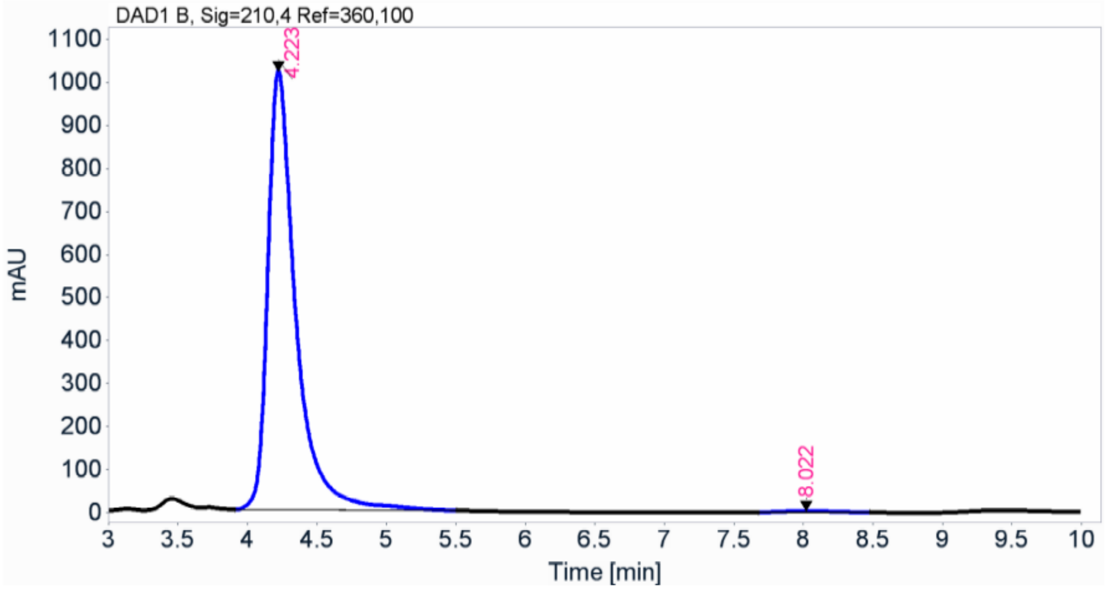

**Signal:**	DAD1 B, Sig=210,4 Ref=360,100				
**RT [min]**	**Type**	**Width [min]**	**Area**	**Height**	**Area%**
4.223	MM	0.2333	14257.078	1018.6358	99.43
8.022	MM	0.4022	82.380	3.4134	0.57

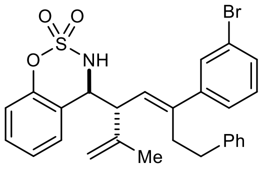

3v

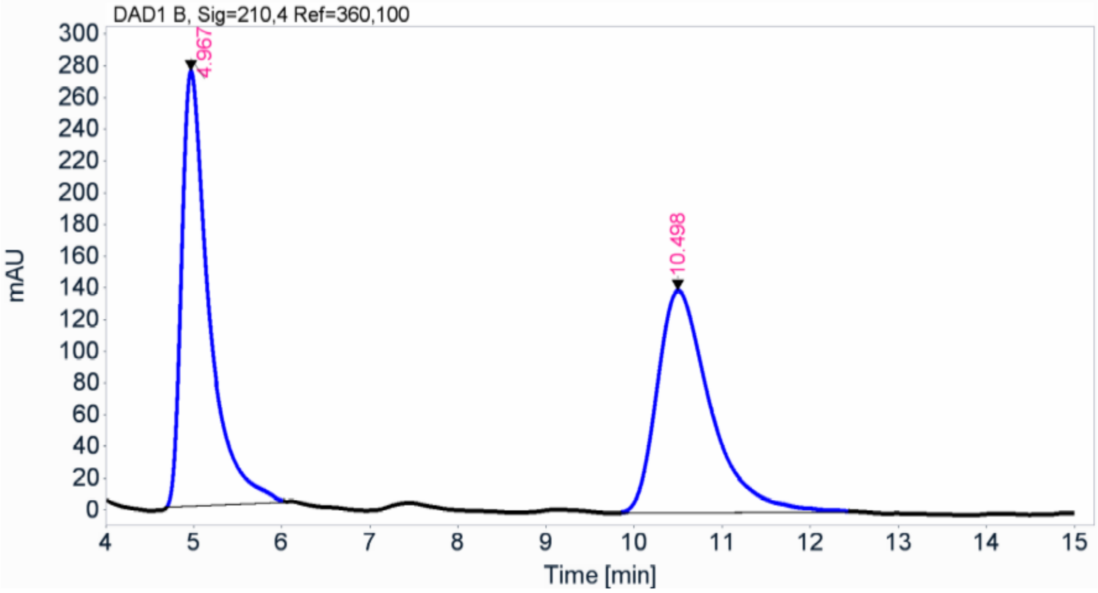

Signal:	DAD1 B, Sig=210,4 Ref=360,100				
RT [min]	Type	Width [min]	Area	Height	Area%
4.967	MM	0.3587	5908.794	274.5458	50.02
10.498	MM	0.7014	5904.677	140.3016	49.98

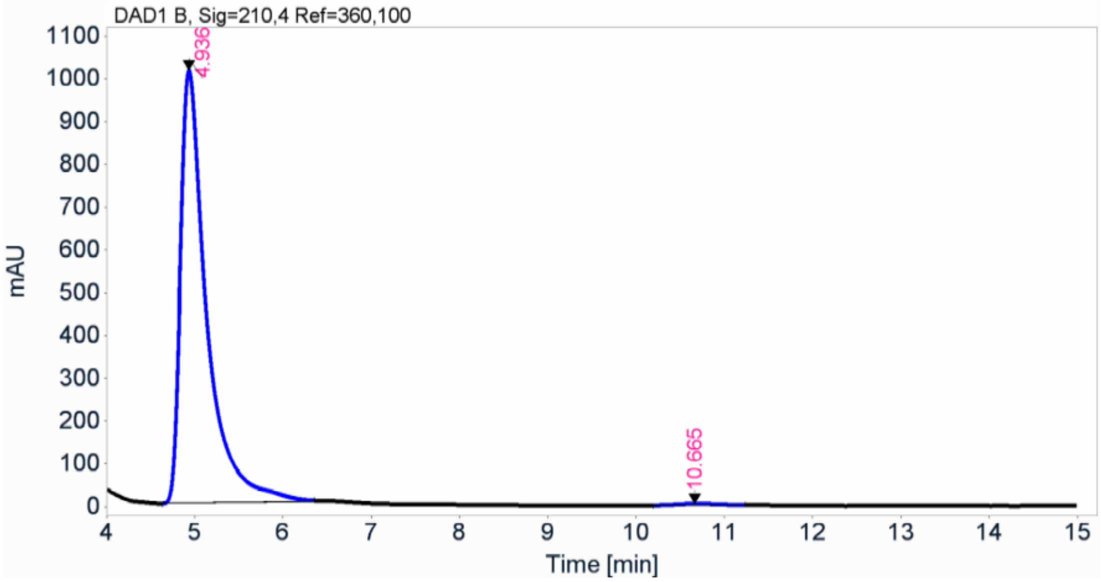

Signal:	DAD1 B, Sig=210,4 Ref=360,100				
RT [min]	Type	Width [min]	Area	Height	Area%
4.936	MM	0.3428	20802.799	1011.3995	99.39
10.665	MM	0.5948	127.803	3.5814	0.61

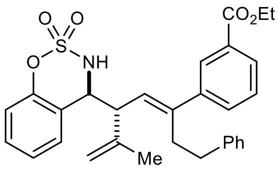

3w

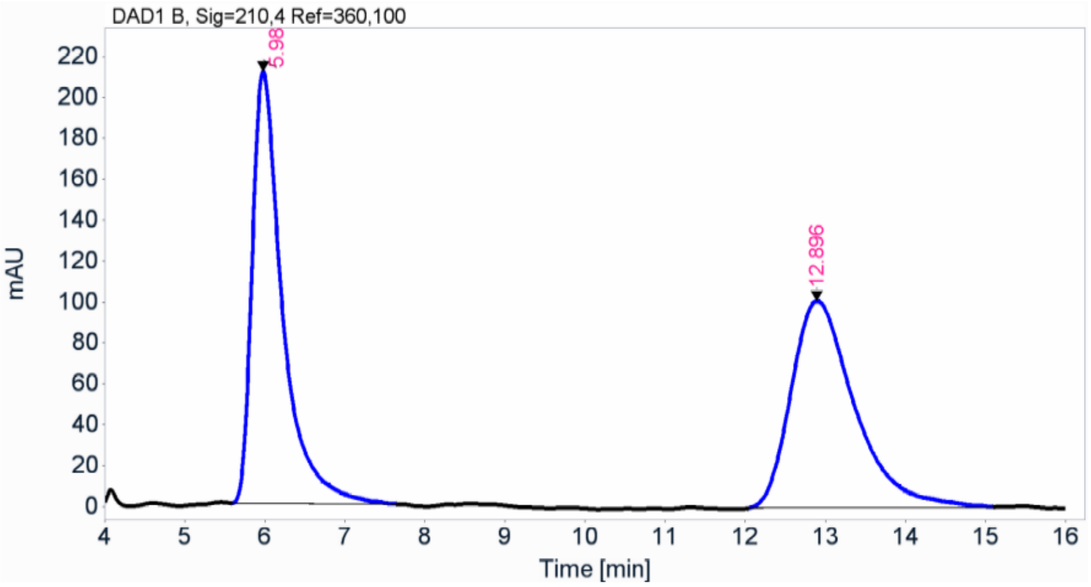

Signal:	DAD1 B, Sig=210,4 Ref=360,100				
**RT [min]**	**Type**	**Width [min]**	**Area**	**Height**	**Area%**
5.980	MM	0.4393	5562.564	211.0285	50.09
12.896	MM	0.9151	5543.536	100.9619	49.91

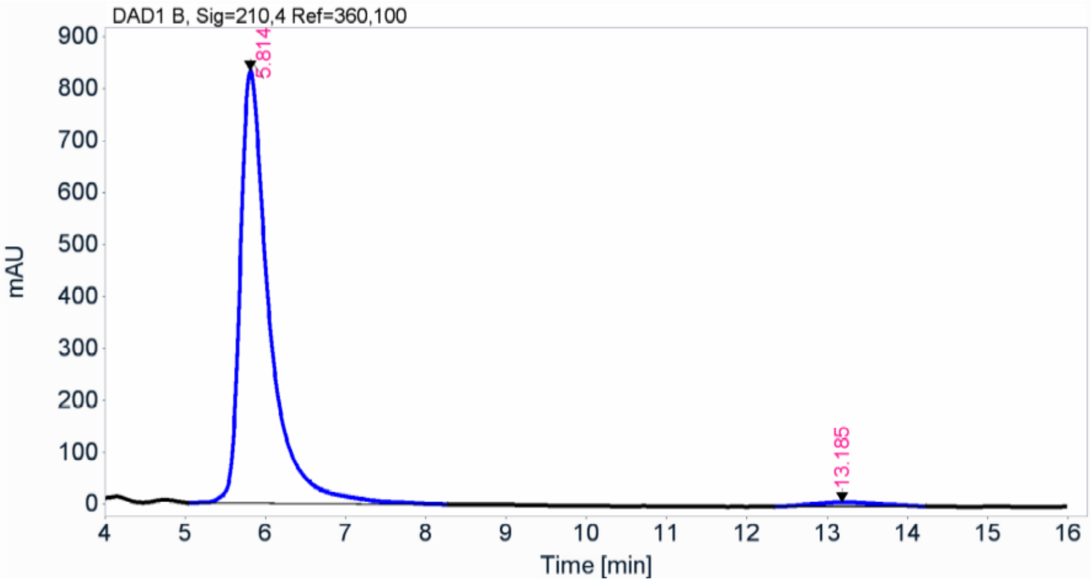

Signal:	DAD1 B, Sig=210,4 Ref=360,100				
**RT [min]**	**Type**	**Width [min]**	**Area**	**Height**	**Area%**
5.814	MM	0.4215	21030.586	831.5473	97.84
13.185	MM	0.9358	463.986	8.2638	2.16

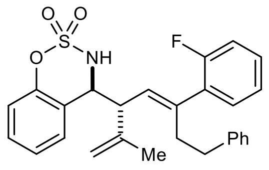

3x

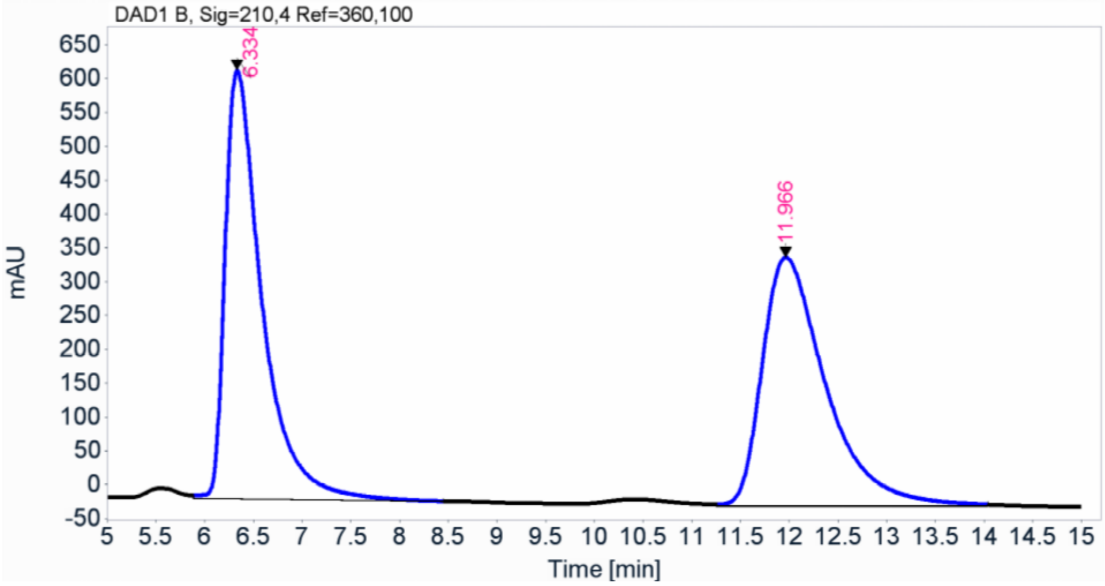

**Signal:**	DAD1 B, Sig=210,4 Ref=360,100				
**RT [min]**	**Type**	**Width [min]**	**Area**	**Height**	**Area%**
6.334	VB	0.3911	16673.391	633.3621	50.00
11.966	MM	0.7565	16671.232	367.2766	50.00

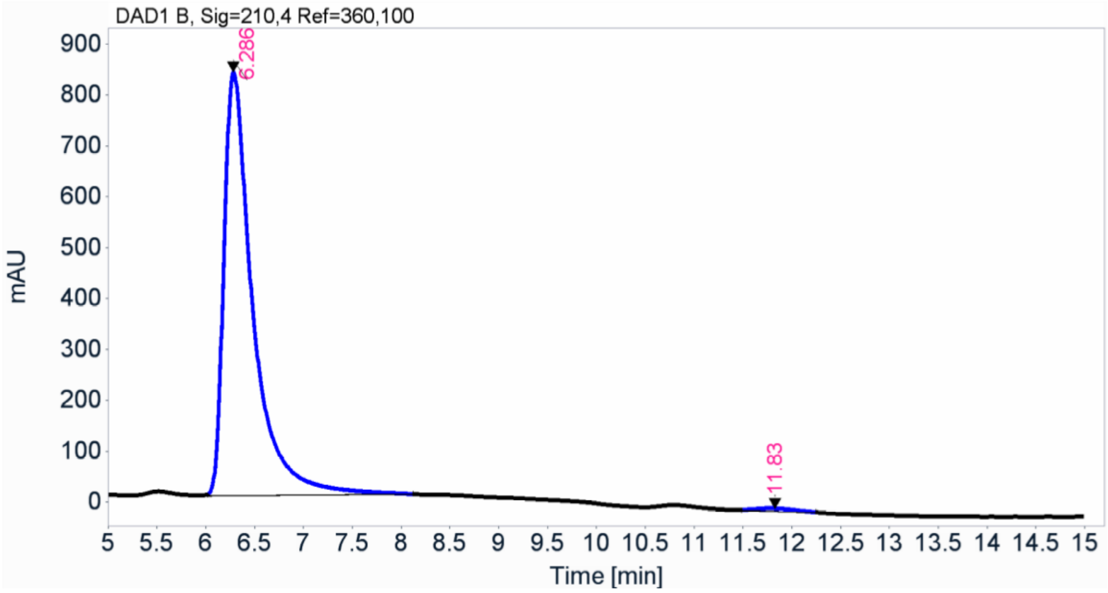

**Signal:**	DAD1 B, Sig=210,4 Ref=360,100				
**RT [min]**	**Type**	**Width [min]**	**Area**	**Height**	**Area%**
6.286	MM	0.3446	17203.555	832.1677	98.95
11.830	MM	0.4877	183.269	6.2631	1.05

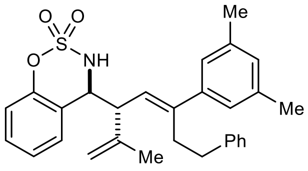

3y

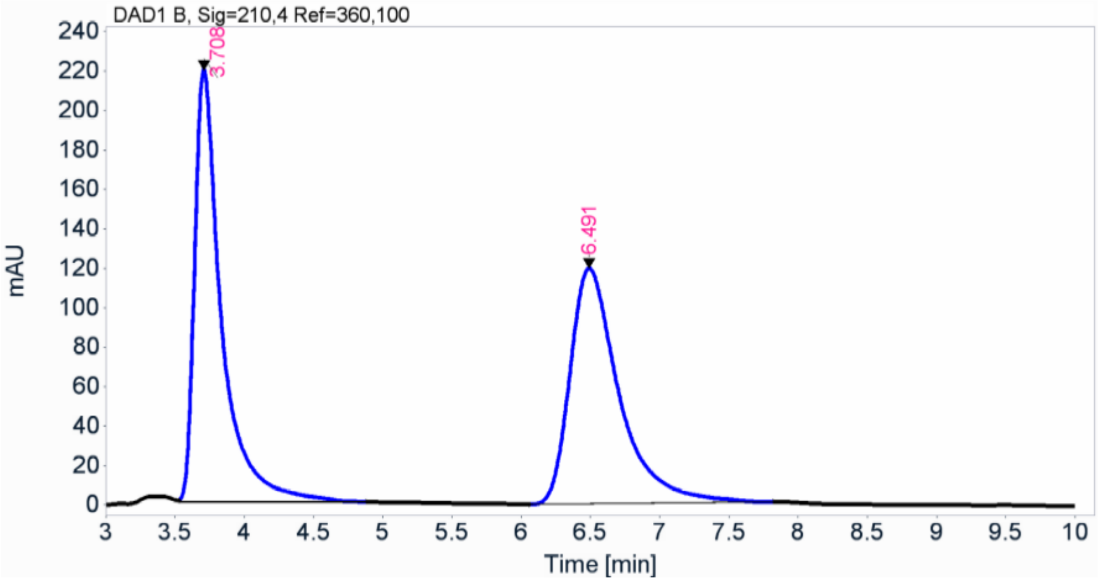

Signal:	DAD1 B, Sig=210,4 Ref=360,100				
**RT [min]**	**Type**	**Width [min]**	**Area**	**Height**	**Area%**
3.708	MM	0.2246	2952.960	219.1051	50.04
6.491	BB	0.3700	2948.601	119.4483	49.96

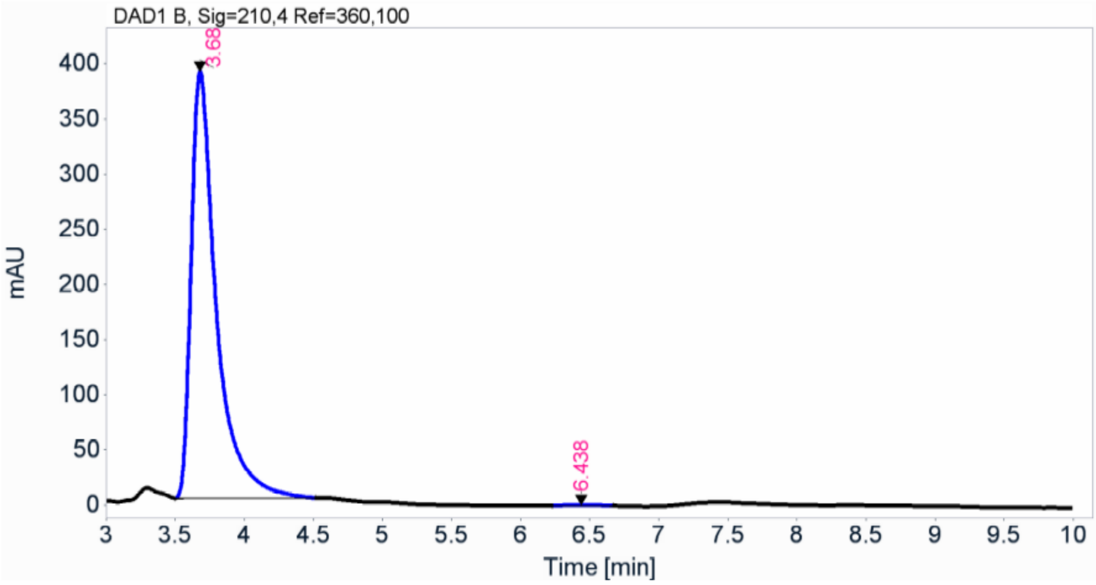

Signal:	DAD1 B, Sig=210,4 Ref=360,100				
**RT [min]**	**Type**	**Width [min]**	**Area**	**Height**	**Area%**
3.680	MM	0.2106	4894.479	387.3864	99.67
6.438	MM	0.2832	16.379	0.9639	0.33

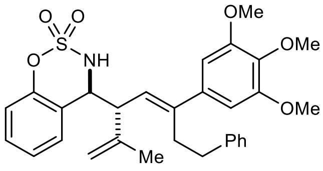

3z

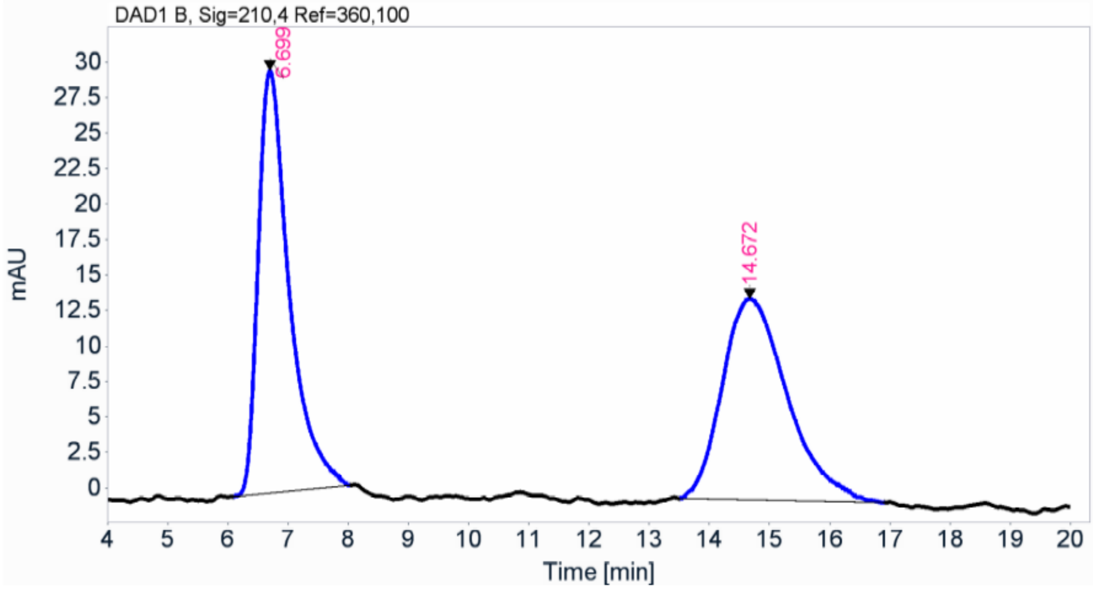

Signal: DAD1 B, Sig=210,4 Ref=360,100

RT [min]	Type	Width [min]	Area	Height	Area%
6.699	BB	0.5384	1066.538	29.7410	50.06
14.672	BB	1.0914	1064.014	14.1488	49.94

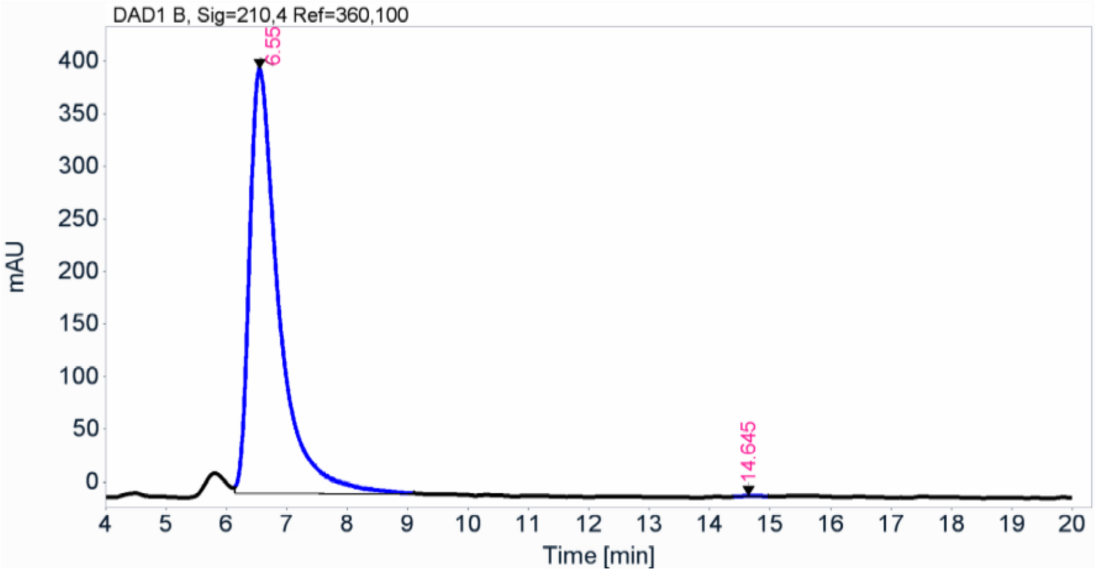

Signal: DAD1 B, Sig=210,4 Ref=360,100

RT [min]	Type	Width [min]	Area	Height	Area%
6.550	MM	0.5632	13621.280	403.0844	99.87
14.645	MM	0.3061	17.177	0.9352	0.13

f
